# The Mechanism of Visible‐Light Photochemical Rearrangements of Conjugated Amide Enolates

**DOI:** 10.1002/chem.71427

**Published:** 2026-07-12

**Authors:** James Mortimer, William Terry‐Wright, Basile F. E. Curchod, Jonathan Clayden

**Affiliations:** ^1^ School of Chemistry University of Bristol Bristol UK

**Keywords:** biradical, DFT, enolate, photochemistry, rearrangement

## Abstract

Deprotonation of conjugated amides forms colored solutions of lithium enolates (trienolates) that absorb light in the visible range of the electromagnetic spectrum. Irradiation of these extended enolates at a suitable wavelength induces a family of skeletal rearrangements, including bond migrations and ring expansions, that proceed through a biradical intermediate. Combined experimental and computational studies reveal that excitation of the extended enolate chromophore promotes homolysis of the C─N bond of the amide via an intersection seam between the S_1_ and S_0_ potential energy surfaces. The resulting singlet biradical undergoes rapid recombination to generate rearranged amide products, with regioselectivity governed by the molecular framework. Acyclic trienolates undergo competing [1,3]‐, [1,5]‐, and [1,7]‐migrations, but incorporation of a ring constrains radical recombination, resulting in regioselective re‐cyclizations. Lack of stereospecificity during the rearrangements and cyclopropylbenzyl ring openings, coupled with a lack of cross‐reactivity in TEMPO trapping and a crossover experiment, indicates the formation of short‐lived, solvent‐caged radical pairs rather than freely diffusing radicals. These findings establish a general mechanistic rationalization of visible‐light‐driven rearrangements of amide enolates, and suggest that ring topology can be used to control the outcome of the biradical recombination.

## Graphical abstract

Visible light irradiation of extended enolates of *N*‐benzylamides leads to homolytic C─N Bond cleavage, generating a biradical. Computational and mechanistic studies suggest rapid radical recombination within a solvent cage to give either ring‐expanded or rearranged products.

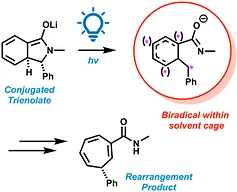

## 1 Introduction

The formation of coloured solutions is a common feature of the chemistry of stabilised anions such as organolithiums or enolates, especially those conjugated with one or more additional C═C bonds (allyllithiums, benzyllithiums, dienolates or trienolates) [1]. However, photochemically‐induced transformations of these chemically reactive chromophores have rarely been exploited, partly due to the practicalities of using standard laboratory equipment to irradiate solutions that must simultaneously be maintained at low temperature.

The increased availability of light‐emitting diodes (LEDs) capable of producing light at a range of wavelengths, including in the UV region, has fuelled a renaissance in the field of synthetic photochemistry and photocatalysis [2]. Most of the recent developments in the area have involved photoredox reactions, [3] but the development of other bond‐breaking, bond‐forming, and rearrangement reactions has also benefited from the use of LEDs to allow mild, non‐thermal irradiation [4]. Whereas photoredox catalysis exploits redox intermediates, previous work from our group demonstrated direct chromoselective excitation of anionic enolates, uncovering a distinct photochemical regime. Some years ago, we discovered serendipitously that irradiation of a range of coloured bicyclic amide trienolate chromophores with an incandescent lamp at low temperature promoted a remarkable family of skeletal rearrangements [5]. In a more recent asymmetric version of this reaction, we used LED irradiation at a suitable wavelength to ring‐expand benzamides, leading to the enantioselective one‐carbon insertion in a benzene ring, yielding enantioenriched cycloheptatrienes as shown in Figure 1 [6, 7].

**FIGURE 1:**
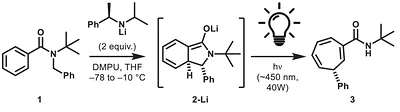
*N*‐Benzylbenzamide 1 undergoes dearomative cyclisation upon treatment with strong base to give a highly colored anion 2‐Li. Irradiation at 450 nm initiates a photochemical ring expansion to cycloheptatriene 3.

Dearomative cyclisation [8, 9] of **1** yields the bicyclic extended amide enolate **2‐Li**. This highly coloured anionic intermediate bears a chromophore with λ_max_ = 449 nm, and in situ irradiation of the reaction mixture as the enolate is formed induces ring‐expanding rearrangement to the cycloheptatriene **3‐Li** and hence, after protonation, **3**. Low temperature LED irradiation of a reactive intermediate using visible wavelength light avoided the lack of selectivity often associated with direct photochemistry (as opposed to photocatalysis), and careful choice of wavelength allowed the irradiation to excite only one reactive intermediate along the reaction pathway, the trienolate, despite the presence of starting material, products, and other intermediates, all of which also contain chromophores. We use the term ‘chromoselectivity’ [6] to refer to this selective excitation of a single reactive intermediate in the presence of other chromophores through choice of irradiation wavelength. In this paper, we elucidate the mechanism of the rearrangement in Figure 1 [6], and explore the reactivity that emerges from visible‐light photochemical rearrangements of the lithium enolates of conjugated amides as a general class of reactants. We use state‐of‐the‐art computational photochemistry to confirm that the photochemical rearrangement shown in Figure 1 [6] proceeds through a singlet biradical pathway, and we use experimental methods to explore the mechanism of similar rearrangements of cyclic and acyclic amide enolates suitable for visible‐light LED irradiation.

## 2 Results and Discussion

### 2.1 Computational Study of the Photochemical Reactivity of Cyclic Trienolates

The thermal reactivity of enolate derivatives of carbonyl compounds is a cornerstone of textbook synthetic chemistry [10]; likewise, the carbonyl group is the prototypical chromophore investigated computationally [11, 12, 13]. The photochemical reactivity of enolates, on the other hand, has received much less attention, although isolated examples of the action of light on enolates have been reported [14, 15].

In the cyclisation of **1** to **2‐Li** (Figure 1), trienolate **2‐Li** undergoes the primary photochemical processes [6]. In the following, we investigate the photochemical pathway of trienolates using a combination of density functional theory (DFT), linear‐response time‐dependent DFT (LR‐TDDFT), and second‐order approximate coupled cluster singles and doubles (CC2) calculations. For computational efficiency, this study focuses on the anion **4** (Figure 2a), chosen as a suitable analogue for enolate **2‐Li**. The Computational Details section (SI) provides all the details of the calculations performed in this work, a benchmark of the level of electronic‐structure theory, and justifications for the use of **4** as a proxy for **2‐Li**.

**FIGURE 2:**
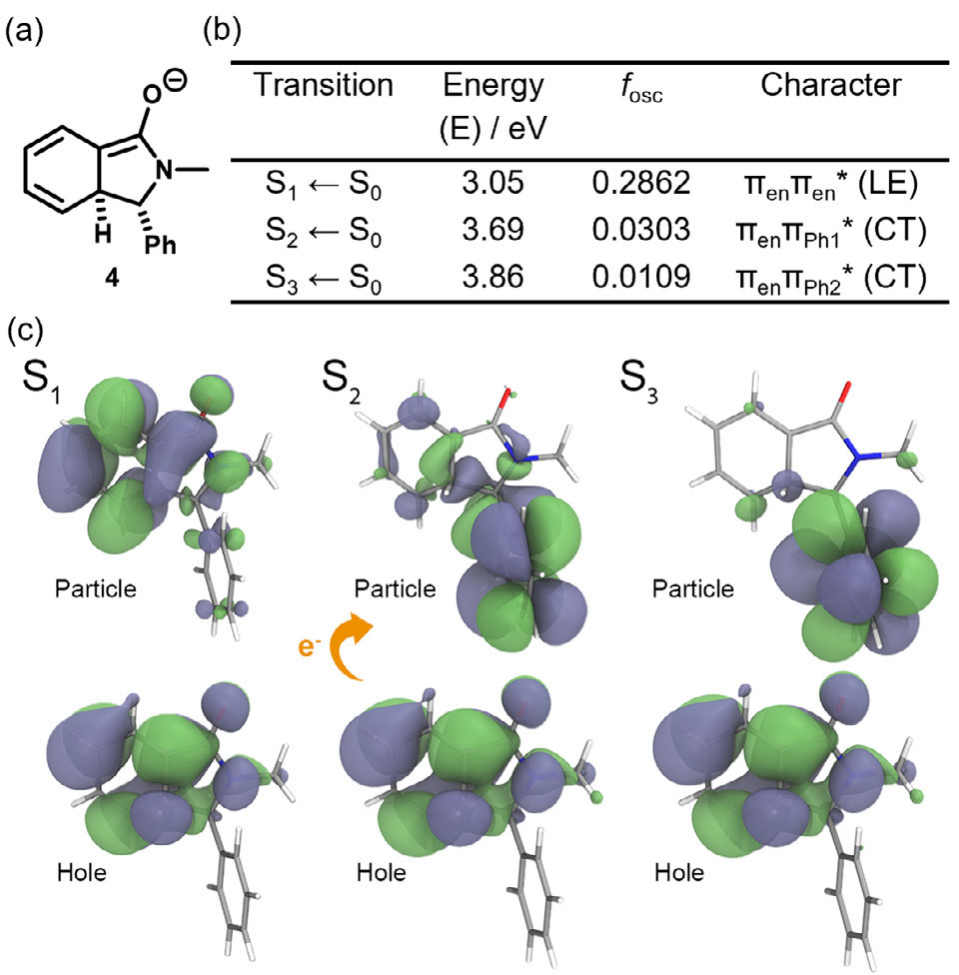
(a) Structure of the simplified anionic analogue 4. (b) Lowest three vertical excitations of 4 at its ground state equilibrium geometry (DFT/ωB97X‐D4/cc‐pVDZ/C‐PCM) calculated at the CC2/cc‐pVDZ level of theory with implicit solvent (THF) modelled as a continuum (COSMO). The table reports transition energies (E), oscillator strengths (f_osc_), and the character of each transition; LE denotes a local excitation, and CT denotes charge transfer (en = enolate, Ph = phenyl). (c) Natural transition orbitals (NTOs) for the first three vertical transitions of **4** at its ground state equilibrium geometry (DFT/ωB97X‐D4/cc‐pVDZ/C‐PCM) calculated at the CC2/cc‐pVDZ/COSMO(THF).

Our investigation focused first on understanding the electronic character of the different excited electronic states of **4** at its ground‐state equilibrium geometry, also known as the Franck‐Condon (FC) geometry. The transition to the first excited electronic state (S_1_) has the highest oscillator strength and shows a ππ* character localised on the enolate moiety (Figure 2b,c) with an energy of 3.05 eV representing an excitation wavelength of 366 nm [CC2/cc‐pVDZ/COSMO(THF)]. Transitions to the S_2_ and S_3_ electronic states, on the contrary, exhibit charge transfer character leading to lower oscillator strengths. The transition energy and higher oscillator strength suggest the S_1_←S_0_ transition is likely to be the electronic state of interest in the photochemistry of **4**.

To confirm this assignment, the (UV–Vis) absorption spectrum of **4** was calculated at the CC2/cc‐pVDZ/COSMO(THF) level of theory using the nuclear ensemble approach (NEA) and compared to the experimental spectrum (Figure 3). The NEA is a strategy allowing to reproduce the width and intensity of a UV/Vis absorption spectrum by sampling nuclear geometries (here, 500) to approximate the ground‐state nuclear probability density of a molecule [16]. While this method incorporates non‐Condon effects, it neglects the role of vibronic transitions [17]. The CC2 simulated absorption spectrum matches closely with the experimental spectrum, with a λ_max_ at 439 and 438 nm, respectively. The calculated molar extinction coefficient is, however, overpredicted [ε = 14487 M^−1^ cm^−1^ calculated (NEA//CC2/cc‐pVDZ/COSMO(THF)) versus ε = 7531.87 ± 3.14 M^−1^ cm^−1^ experimentally]—an expected feature of CC2 calculations [13]. The S_1_←S_0_ contribution to the absorption spectrum (purple shaded area in Figure 3) dominates the total absorption spectrum in the 450 nm region of the spectrum, overlapping well with the emission spectrum of the Kessil Blue lamp (orange curve in Figure 3) used experimentally to trigger the photochemical reaction. This assignment confirms that photoexcitation of **4** with a Kessil Blue lamp will target its first excited electronic state, and this electronic state will be the primary focus of our subsequent photochemical study.

**FIGURE 3:**
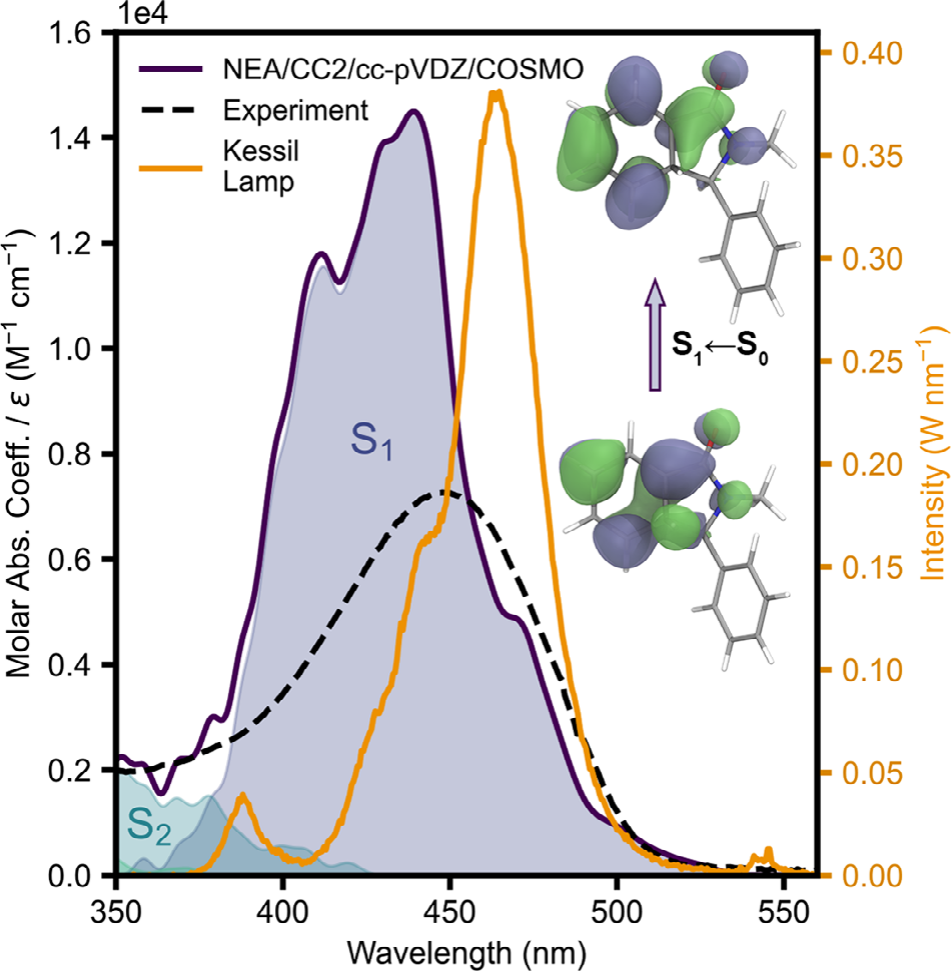
Experimental and theoretical absorption spectra of **4**. The theoretical absorption cross‐section (solid purple line) was obtained with the NEA using CC2/cc‐pVDZ/COSMO(THF), based on 500 sampled geometries and a gaussian line broadening of 0.05 eV. The theoretical absorption spectrum was further decomposed into contributions from individual transitions (shaded regions). The experimental UV–Vis absorption spectrum of **2‐Li** (dashed black line) and the emission spectrum of the Kessil blue lamp used in the photochemical ring expansion of **1** are also shown.

To unravel the photochemical reaction of **4** following photoexcitation to its first excited electronic state, we isolated three critical geometries characterising its S_1_ potential energy surface—a minimum energy geometry (min), a transition state (TS), and an intersection seam (IS)—using LR‐TDDFT/TDA/ωB97X‐D4/cc‐pVDZ/C‐PCM(THF) (see SI for additional details). We then built a path connecting these critical geometries, together with the ground‐state geometry, by using geodesic interpolations and CC2/cc‐pVDZ/COSMO(THF) for the electronic energies [18]. This interpolation path (Figure 4a) is not a minimum‐energy path per se, but it provides information on the potential nonradiative pathways undergone by a photoexcited molecule [19].

**FIGURE 4:**
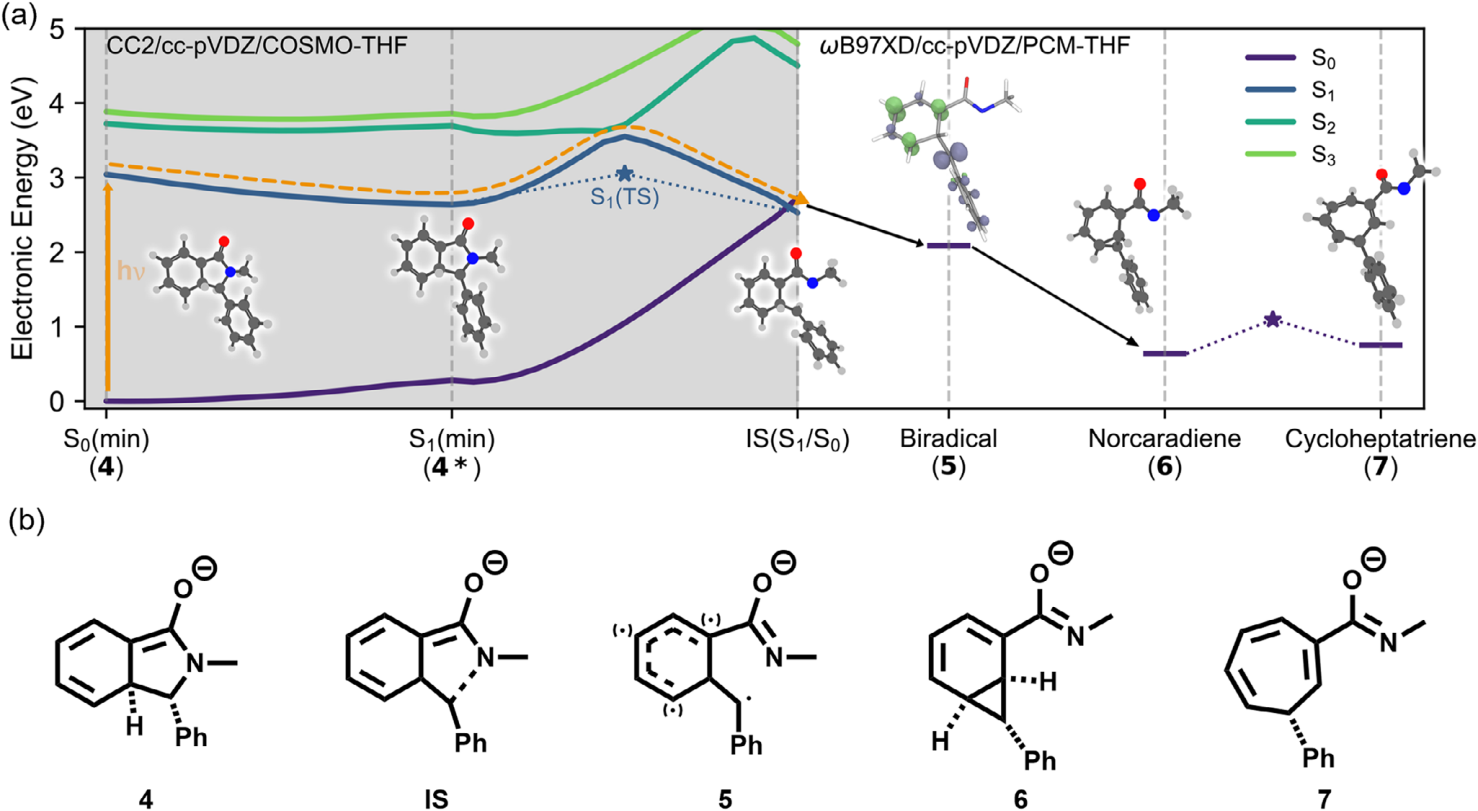
(a) Photochemical and chemical pathway for **4** calculated with CC2/cc‐pVDZ/COSMO(THF) (from the S_0_ minimum to the intersection seam (IS) and with (U)DFT/ωB97X‐D4/C‐PCM(THF) (biradical onwards). Stars represent transition states. See Computational Details in the Supporting Information. (b) Schematic representation of the various critical geometries along the photochemical pathway.

The interpolation path indicates that **4** can relax towards an S_1_ minimum following photoexcitation. This S_1_ minimum is connected to an intersection seam between S_1_ and S_0_ via a transition state (star in Figure 4a), with an energy lower than that of the photoexcited **4** at the FC point (S_0_(min)). Reaching the intersection seam requires a significant elongation of the C─N bond of the enolate moiety (IS structure in Figure 4a,b), leading to the nonradiative transfer of **4** from S_1_ to S_0_ and the formation of a biradical‐like structure, **5**, in the ground electronic state. This biradical structure exhibits positive spin density around the conjugated enolate and negative spin density on the pendant phenyl ring (see Figure 4a).

The reaction proceeds in the ground electronic state with the recombination of the biradical to form a norcaradiene intermediate. The norcaradiene is not the isolated species experimentally (although alternatively substituted benzamides do give norcaradiene products) [6], but instead rearranges to a cycloheptatriene. Although this transformation appears unfavorable according to DFT (both the enolate intermediates, **6** and **7,** as well as their protonated products, exhibit this trend), high‐level CCSD(T) calculations prove that the cycloheptatriene is indeed the favoured product by 0.38 eV (see Computational Details).

These computational results establish a mechanistic hypothesis for the formation of **3** (Figure 1) in which photoexcitation of the extended amide enolate chromophore **2‐Li** leads, via a conical intersection between the first‐excited and ground electronic states, to a C─N bond homolysis and formation of a short‐lived singlet biradical. Recombination of the biradical forms a norcaradiene that undergoes a 6π‐electrocyclic ring opening (either as the anion or as a neutral species after protonation) to give the final product **3**.

### 2.2 Experimental Study of the Photochemical Reactivity of Acyclic Enolates

Having identified a potential pathway for the photochemical transformation of **4**, we next sought experimental evidence for intermediate biradicals in the photochemical rearrangements of enolates. To this end, we examined the photochemistry of structurally simplified and systematically modified enolates designed to probe how substitution and ring incorporation influence the outcome of the rearrangement. We evaluated acyclic trienolate systems to determine whether the biradical mechanism proposed for the bicyclic chromophore could be generalised to open‐chain analogues.

The photochemically induced formation of a biradical species during the rearrangement of **2‐Li** suggests that similar chromophores embedded in different structures might undergo similar photochemical rearrangements. The most obvious simplification to the structure of **1** entails the removal of the bicyclic bridge (Figure 5a), to give a potential trienolate intermediate **8a‐Li** that would be formed from the hexa‐3,5‐dienamide **8a**. *N*‐Benzyl hexadienamide **8a** was synthesised (see Supporting Information) and deprotonated with LDA to give a green coloured solution, with an experimental UV–Vis absorption maxima (λ_max_) between 350 and 390 nm. Single point CC2/cc‐pVDZ/COSMO(THF) calculations on the optimised ground state structure of **8a‐Li** suggested a λ_max_ of 350 nm. Irradiation of the enolate at 385 nm triggered a photochemical rearrangement that yielded three regioisomeric products **9a**, **10a**, and **11a** corresponding to [1,3], [1,5], or [1,7] shifts of the *N*‐benzyl group (Figure 5b), an outcome consistent with the operation of a radical pathway. Similar outcomes, with moderate yields and minor variation in selectivities, were obtained from alternatively substituted *N*‐benzylhexadienamides **8b‐8d**.

**FIGURE 5:**
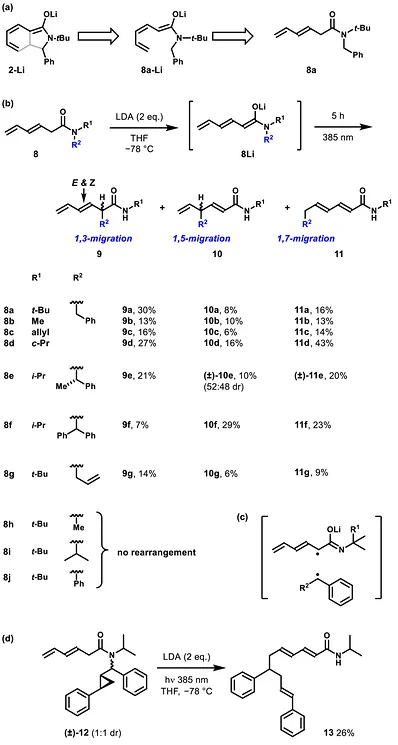
(a) Conceptual simplification of **2‐Li** to **8a‐Li**; (b) rearrangements of acyclic extended enolates **8‐Li** give regioisomeric mixtures **9–11**; (c) the intermediate radical pair; (d) diagnostic ring opening and rearrangement of a cyclopropylbenzyl radical intermediate.

A similar amide derivative of enantiopure a‐methylbenzylamine **8e** was subjected to the same reaction conditions. Deprotonating and irradiation at 385 nm likewise led to a mixture of three regioisomers **9e**, **10e**, and **11e**, two of them as mixtures of diastereoisomers. Crucially, the ee of **11e** and of the diastereoisomers of **10e** could be determined: the rearranged products were formed as racemic mixtures. This complete loss of stereochemical integrity rules out the operation of concerted suprafacial or antarafacial sigmatropic shifts and is instead consistent with the formation of a discrete, planar benzylic radical intermediate formed by homolysis of the C─N bond (Figure 5c). Deprotonation and irradiation of *N*‐benzhydryl‐substituted **8f** and *N*‐allyl‐substituted **8g**, from which a stabilised migrating allyl or benzhydryl radical may be formed, likewise returned rearranged products (with some migration of the allyl group noted from **8g**). Other amide derivatives **8h**, **8i**, and **8j**, from which homolysis cannot give a stabilized radical, gave no products, consistent with the lack of an available radical pathway.

The instability of cyclopropylalkyl radicals provides a further probe of the intermediacy of a biradical [20], thus phenylcyclopropyl‐containing dienamide **12** was synthesised as a diagnostic substrate. Deprotonation and irradiation of **12** yielded the linear styrene derivative **13**, a product of cyclopropylcarbinyl radical ring‐opening. The absence of products retaining the cyclopropane ring implies that the rate of ring opening (*k* ≈ 10^8^ s^−1^) [21] exceeds the rate of radical recombination, indicating a finite lifetime for the radical species.

These experiments are supportive of a mechanism involving a biradical intermediate, as computed for the bicyclic analogue. Nonetheless, mechanistic interrogation using the persistent radical TEMPO revealed no trapped adducts, and crossover experiments using a mixture of the doubly differentiated amides **8a** and **8e** showed no intermolecular exchange of migrating groups. Collectively, therefore, these data indicate that C─N bond homolysis of the enolate chromophore generates a singlet radical pair that is confined within a solvent cage. Rapid geminate recombination outcompetes both diffusion into the bulk medium and intermolecular trapping.

### 2.3 Experimental Study of the Photochemical Reactivity of Lactam Trienolates

Rearrangements of the acyclic trienolates **8‐Li** proved that C─N homolysis of extended amide enolates with visible light yields radical pairs, but the availability of three alternative recombination pathways resulted in poor regiocontrol. Embedding the chromophore within a lactam would tether the radical pair, imposing geometric constraints that may limit the reaction pathways available and restore the selectivity noted in the rearrangement of **2‐Li**.

Six‐membered lactam **14a** was synthesised using an aldol condensation, as described in the Supporting Information. Calculations (CC2/cc‐pVDZ/COSMO) suggested that its enolate would have an absorbance maximum of 352 nm, and indeed deprotonation of **14a** with LiHMDS (to avoid a competing conjugate addition of the diisopropylamide anion in LDA) and irradiation at 385 nm triggered homolysis and rearrangement (Figure 6). Two products were formed. One pathway entailed a [1,5]‐radical migration, yielding a substituted cyclohexenamide **16a** (17%, dr 2:1). This was accompanied by a competing [1,3]‐migration leading to ring‐contraction, affording the strained cyclobutyl amide **15a** (14%) with some degree of diastereoselectivity.

**FIGURE 6:**
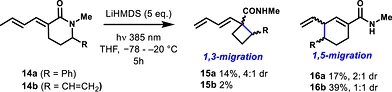
Photochemical rearrangements of lactam trienolates.

The poor yields of these reactions are likely due to competing oligomerisation, but some improvement in both yield and selectivity was noted with the related vinyl‐substituted lactam **14b**. Rearrangement of the enolate of **14b** by irradiation at 385 nm gave almost complete selectivity for [1,5]‐migration, giving the cyclohexenyl products **16b** (39%), accompanied by 2% ring‐contracted **15a**, possibly because **15b** is converted into **16b** by [3,3]‐sigmatropic rearrangement.

## 3 Conclusion

Visible‐light irradiation of conjugated amide enolates promotes a distinctive class of skeletal rearrangements that proceed through a short‐lived biradical intermediate. Combined computational and experimental studies identify an intersection seam linking the S_1_ and S_0_ potential‐energy surfaces as the key step leading to C─N bond homolysis and formation of a singlet biradical. The subsequent intramolecular radical recombination defines the product distribution, with incorporation of a ring constraining the geometry of the intermediate and restoring regio‐ and diastereocontrol. The absence of TEMPO‐trapped products and the outcome of crossover experiments confirm that recombination occurs within a solvent cage rather than through free radical diffusion. These results provide a unified mechanistic framework for understanding visible‐light‐induced rearrangements of amide enolates and establish design principles for controlling selectivity in photochemical transformations of reactive anionic chromophores.

## 4 Experimental Section

### 4.1 Computational Details

The calculations reported in this work employed density functional theory (DFT) and linear‐response time‐dependent density functional theory (LR–TDDFT) within the Tamm–Dancoff approximation (TDA) or second‐order approximate coupled‐cluster singles and doubles (CC2) with the cc‐pVDZ basis set [22] unless otherwise stated. For DFT and LR‐TDDFT/TDA, the long‐range corrected exchange‐correlation functional ωB97X‐D4 with Grimme's D4 dispersion correction was used [23]. Stationary points were located by standard geometry optimisations and confirmed by frequency calculations showing the lack of imaginary frequencies (for minima) or a single imaginary frequency (for transition states).

All single‐point calculations and geometry optimisations using DFT, LR–TDDFT/TDA, and EOM–CCSD were conducted with the ORCA electronic structure program (version 6.1.0) [24, 25]. All CC2 and ADC(2) calculations utilized the TURBOMOLE (version 7.8) [26] software package with the resolution of the identity approximation (RI‐CC2 or RI‐ADC(2)). For the nuclear ensemble approach (NEA), NewtonX (Version 2.6) [27] was used and combined with TURBOMOLE (for harmonic Wigner distribution, excitation energies, and oscillator strengths).

Solvation was accounted for using the Conductor‐like Screening Model (COSMO) for CC2 calculations and Conductor‐like Polarizable Continuum Model (C‐PCM) in the case of DFT and LR–TDDFT/TDA calculations. The solvent chosen was THF to match that used in the reaction.

Additional exploratory calculations were also performed and are detailed in the Supporting Information.

### 4.2 Representative Procedure for Photochemical Rearrangement of Acyclic Enolates

Diisopropylamine (2.0 eq.) was dissolved in anhydrous THF (5 mL) before dropwise addition of *n*‐BuLi (2.1 eq., 2.5 M in hexanes) at −78°C. A solution of the amide (1.0 eq.) in anhydrous THF (0.5 M) was added to the basic solution at −78°C to give a colored enolate solution. Irradiation was started at 385 nm (Thorlabs M385LP1) for 5 h before quenching with NH_4_Cl(aq.) (10 mL). The layers were separated at room temperature, and the aqueous layer was extracted with EtOAc (3 × 10 mL). The combined organic extracts were washed with brine (10 mL), dried with Na_2_SO_4_, filtered, and concentrated under reduced pressure. Purification by flash column chromatography afforded the photochemical products, as described in detail in the Supporting Information.

## Conflicts of Interest

The authors declare no conflicts of interest.

## Supporting Information

The authors have cited additional references within the Supporting Information [18, 28, 29, 30, 31, 32, 33, 34, 35].

## Supporting information




**Supporting File 1**: chem71427‐sup‐0001‐SuppMat.pdf

## Data Availability

The data that support the findings of this study are available in the supplementary material of this article.
