# Supplementary material for: The Mechanism of Visible‐Light Photochemical Rearrangements of Conjugated Amide Enolates
Source: Chemistry. 2026 Jul 12;32(39):e71427. doi: 10.1002/chem.71427 (PMC13715451; doi:10.1002/chem.71427)
Supplement: Supplementary file 1 [file CHEM-32-e71427-s001.pdf]

The mechanism of visible-light photochemical rearrangements of conjugated amide enolates

James Mortimer[a], William Terry-Wright[a], Basile F. E. Curchod[a*] and Jonathan Clayden[a*]

1. COMPUTATIONAL DETAILS AND FURTHER CALCULATIONS ... 2

1.1. Computational details ... 2
1.2. Simplified molecular system for calculations ... 2
1.3. Benchmarking electronic-structure methods at the Franck-Condon point ... 3
1.4. Geodesic interpolation pathways ... 4
1.5. Impact of the simplification of 2-Li on electronic energies ... 5
1.6. Comparing RI-CC2 and LR-TDDFT/TDA/ΩB97X-D4 beyond the Franck-Condon point ... 6
1.7. Monitoring the potential importance of triplet states ... 7
1.8. Probing the intersection seam with mixed-reference spin-flip TDDFT ... 8
1.9. Photoabsorption (UV-Vis) spectra ... 8
1.10. Norcaradiene vs cycloheptatriene [CCSD(T)] ... 9

2. EXPERIMENTAL ... 11

2.1. Materials ... 11
2.2. Methods ... 11
2.3. Instrumentation ... 11
2.4. UV-Visible Spectroscopy Measurements ... 12
2.5. Photochemistry set-up ... 15
2.6. LED Emission Data ... 16
2.7. General Procedures ... 19
2.8. Experimental Procedures ... 22
2.9. Mechanistic Experimentation (TEMPO and crossover experiments) ... 71
2.10. NMR Spectra ... 72

3. REFERENCES ... 165

1. Computational Details and Further Calculations

1.1. Computational details

All the calculations reported in this work employed density functional theory (DFT) and linear-response time-dependent density functional theory (LR-TDDFT) within the Tamm-Dancoff approximation (TDA) or second-order approximate coupled-cluster singles and doubles (RI-CC2) with the cc-pVDZ basis set[1] unless otherwise stated. For DFT and LR-TDDFT/TDA, the long-range corrected exchange-correlation functional ωB97X-D4 with Grimme's D4 dispersion correction was used.[2] Stationary points were located by standard geometry optimisations and confirmed by frequency calculations showing the lack of imaginary frequencies (for minima) or a single imaginary frequency (for transition states).

All single-point calculations and geometry optimisations using DFT, LR-TDDFT/TDA and EOM-CCSD were conducted with the ORCA electronic structure program (version 6.1.0).[3, 4] All CC2 and ADC(2) calculations utilised the TURBOMOLE (version 7.8)[5] software package with the resolution of the identity approximation (RI-CC2 or RI-ADC(2)). For the nuclear ensemble approach (NEA), NewtonX (Version 2.6)[6] was used and combined with TURBOMOLE (for harmonic Wigner distribution, excitation energies and oscillator strengths).

Solvation was accounted for using the Conductor-like Screening Model (COSMO) for CC2 calculations and Conductor-like Polarizable Continuum Model (C-PCM) in the case of DFT and LR-TDDFT/TDA calculations. The solvent chosen was THF to match that used in the reaction.

Additional exploratory calculations were also performed and are detailed below.

1.2. Simplified molecular system for calculations

Two key modifications were made to the enolate **2-Li** to improve computational efficiency to give anion **4**. Firstly, we replaced the *tert*-butyl protecting group with a methyl group to reduce the computational cost of our calculations. It is known that systems with a methyl group in this position can undergo enolate photochemistry – for example, see compound **8b** in the main text.

Secondly, the free anion was chosen as opposed to the ion pair (considering the reaction involves a lithium counter-ion). Further investigation into the effect of these modifications is discussed in Section 1.5 below.

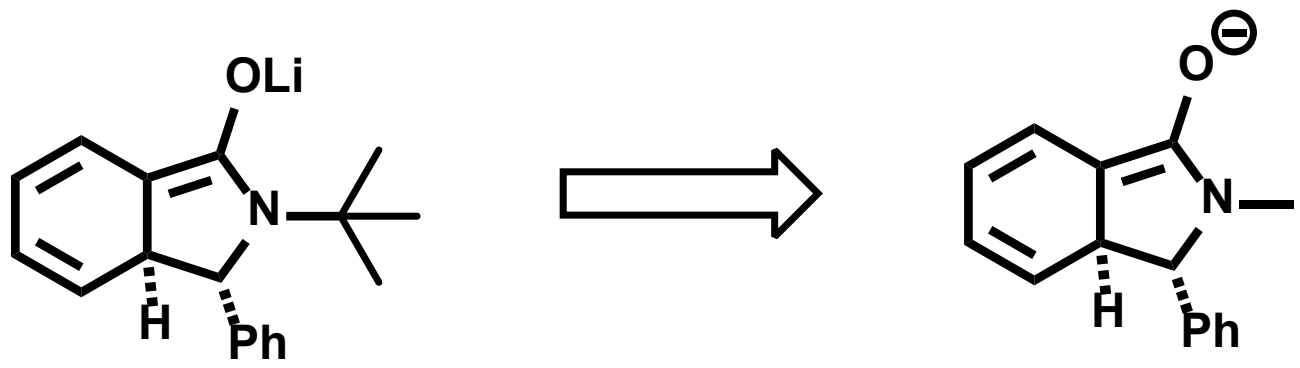

4

Figure S1. Modifications made to the lithium enolate 2-Li, giving 4 as a suitable derivative for computational studies.

1.3. Benchmarking electronic-structure methods at the Franck-Condon point

We first conducted a benchmark of a series of electronic structure methods to probe the excitation energies and oscillator strengths of **A**. Both LR-TDDFT/TDA (with the ωB97X-D4 & PBE0 functional) and *wavefunction-based* methods (ADC2, RI-CC2) were investigated using the cc-pVDZ basis set and the high-level EOM-CCSD method with the same basis set was used as a benchmark. The results are summarised in Table S1.

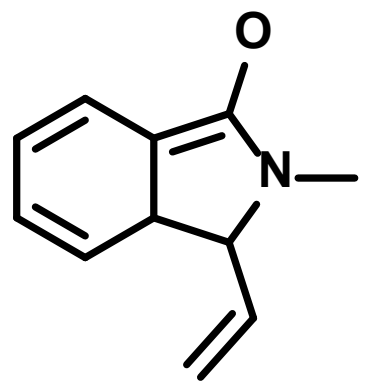

A

Table S1. Excitation energies (ΔE) and oscillator strengths (f) of A at its ground state geometry (optimised at the ωB97X-D4/cc-pVDZ/gas level of theory) obtained at various levels of theory using the cc-pVDZ basis set in the gas phase.

	EOM-CCSD		RI-CC2		ADC(2)		LR-TDDFT/TDA ωB97X-D4		LR-TDDFT/TDA PBE0	
**Transition**	ΔE(eV)	*f*	ΔE(eV)	*f*	ΔE(eV)	*f*	ΔE(eV)	*f*	ΔE(eV)	*f*
$S_1 \leftarrow S_0$	3.23	0.2035	3.17	0.1993	3.04	0.1774	3.51	0.2423	2.55	0.0087
$S_2 \leftarrow S_0$	4.03	0.0109	3.51	0.0208	3.53	0.0106	4.18	0.0159	3.48	0.2448
$S_3 \leftarrow S_0$	5.05	0.0039	4.86	0.0215	4.88	0.0143	5.41	0.0028	4.46	0.0202

In general, the wavefunction-based methods ADC2 and RI-CC2 are in good agreement, with RI-CC2 giving results closer to those of EOM-CCSD. The different functionals, used in combination with LR-TDDFT/TDA, deviate more significantly from the EOM-CCSD reference. As expected from earlier benchmarks,[7] ωB97X-D4 overestimates the transition energies. PBE0 yields vertical transitions that are too low in energy and alter the ordering of the states, possibly due to the charge-transfer nature of some of the low-lying transitions (see main text). Therefore, ωB97X-D4 was chosen as a suitable compromise for the geometry optimisations conducted in this work. The impact of solvent was investigated based on single point calculations using an implicit C-PCM model and combined with LR-TDDFT/TDA (Table S2). These calculations show that the bright S_1 state is further detached from the other electronic states.

Table S2. Excitation energies (ΔE) and oscillator strengths (f) of A at its ground state geometry (optimised at the ωB97X-D4/cc-pVDZ/gas level of theory) with ωB97X-D4/cc-pVDZ/C-PCM(THF).

		LR-TDDFT/TDA ωB97X-D4 C-PCM(THF)	
**Transition**	**Character**	ΔE(eV)	*f*
$S_1 \leftarrow S_0$	$\pi\pi_{en}^*$	3.40	0.3019
$S_2 \leftarrow S_0$	$\pi\pi_{Ph1}^*$	4.93	0.0226
$S_3 \leftarrow S_0$	$n\pi_{en}^*$	5.63	0.0053

Next, the full system **4** was considered and shows good agreement on the energies, oscillator strengths and characters for the first three electronic transitions of the molecule at its Franck-Condon point.

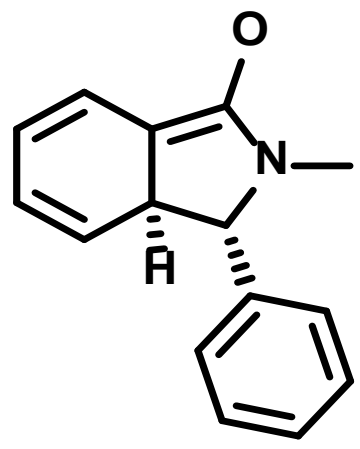

4

Table S3. Excitation energies (ΔE) and oscillator strengths (f) of 4 at its ground state geometry (optimised at the ωB97X-D4/cc-pVDZ/gas level of theory) obtained at two levels of theory using the cc-pVDZ basis set in the solvent phase. RI-CC2 D1 diagnostic = 0.0968. Both methods agree on the excitation character based on visualization of the natural transition orbitals.

		RI-CC2/COSMO		LR-TDDFT/TDA ωB97X-D4/C-PCM(THF)	
**Transition**	**Character**	ΔE(eV)	*f*	ΔE(eV)	*f*
$S_1 \leftarrow S_0$	$\pi\pi_{en}$*	3.04	0.2370	3.37	0.2842
$S_2 \leftarrow S_0$	$\pi\pi_{Ph1}$*	3.72	0.0268	4.60	0.0258
$S_3 \leftarrow S_0$	$\pi\pi_{Ph2}$*	3.88	0.0124	4.80	0.0139

This was repeated for the same molecule, this time optimised with an implicit solvent (THF) using ωB97X-D4/cc-pVDZ/C-PCM(THF). Comparing the vertical transitions between LR-TDDFT and RI-CC2 within an implicit solvent again shows good agreement between the two methods.

Table S4. Excitation energies (ΔE) and oscillator strengths (f) of 4 at its ground state geometry (optimised at the ωB97X-D4/cc-pVDZ/C-PCM(THF) level of theory) obtained at two levels of theory using the cc-pVDZ basis set in the solvent phase (C-PCM for LR-TDDFT/TDA and COSMO for RI-CC2). RI-CC2 D1 diagnostic = 0.0982.

		RI-CC2/COSMO		LR-TDDFT/TDA ωB97X-D4/C-PCM(THF)	
**Transition**	**Character**	ΔE(eV)	*f*	ΔE(eV)	*f*
$S_1 \leftarrow S_0$	$\pi\pi_{en}$*	3.05	0.2367	3.38	0.2862
$S_2 \leftarrow S_0$	$\pi\pi_{PhA}$*	3.69	0.0303	4.57	0.0277
$S_3 \leftarrow S_0$	$\pi\pi_{PhB}$*	3.86	0.0109	4.78	0.0117

1.4. Geodesic interpolation pathways

Linear interpolations between critical geometries (minima, transition states, intersection seam) were used to investigate the potential energy surfaces of enolates. The geodesic interpolation takes 17 representative geometries along a linear interpolation between two critical (optimised) geometries. These pathways are not a minimum energy pathway between critical points of the potential energy surfaces, but they offer a good indication of potential trajectories of a photoexcited molecule on a potential energy surface.

Interpolations were generated using geodesic interpolation between the two geometries of interest using the code described by Martínez *et al.*[8] From this, 17 geometries of interest were generated and single point calculations on each of these were run. The critical geometry for the intersection seam was obtained by running an exploratory Born-Oppenheimer molecular dynamics simulation of **4** in S_1 (using NewtonX and Gaussian 16 Rev C.01)[9] with LR-TDDFT/TDA/ωB97X-D/cc-pVDZ). During this dynamics simulation, the molecule reached an intersection seam (with a significant elongation of its C–N bond). We used the geometry at the last step of the dynamics (before reaching the intersection seam) to launch a geometry

optimisation in S_1 with ORCA and LR-TDDFT/TDA/ωB97X-D4/cc-pVDZ, leading to the isolation of a geometry near the seam of intersection between S_1 and S_0. We note that the limitations of the adiabatic approximation in LR-TDDFT do not allow us to locate a precise minimum-energy conical intersection.

1.5. Impact of the simplification of 2-Li on electronic energies

To investigate the similarities in electronic structure between **2-Li** and the simplified molecular structure **4**, we used geodesic interpolation pathways (from the S_0 minimum to the S_1 minimum of each system) to compare the electronic energies of limiting structures. Firstly, the effect of the N-protecting group (methyl [**4**] vs *tert*-butyl group [the anion of **2-Li**]) was shown to have no significant effect, as shown in Figure S2.

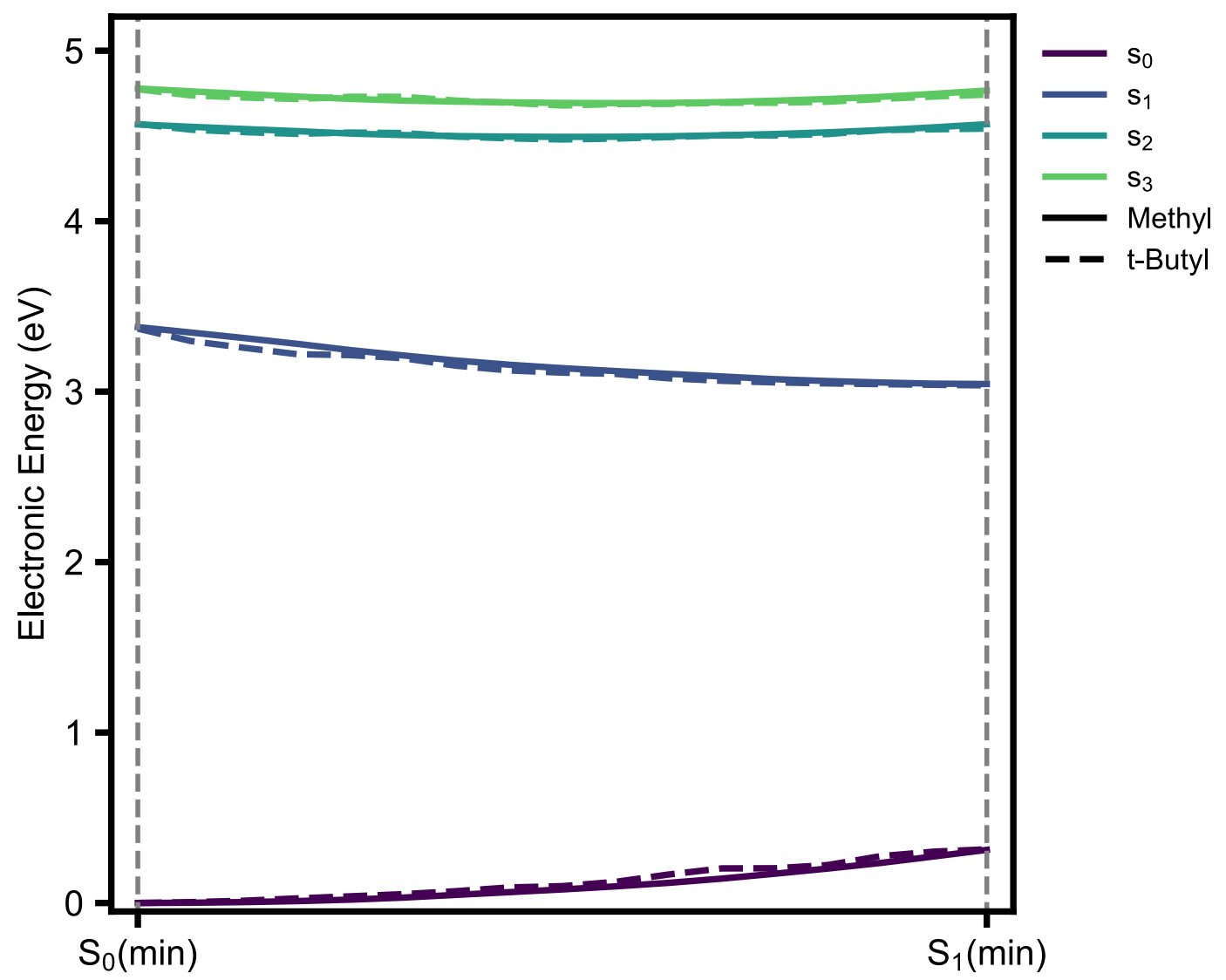

Figure S2. Geodesic interpolation from the ground state minimum energy geometry to the first excited state minimum energy geometry optimized with the ωB97X-D4/cc-pVDZ/C-PCM(THF) level of theory for both the anion 4 (solid line) and its *N-tert*-butyl analogue (dashed line), calculated at the ωB97X-D4/cc-pVDZ/C-PCM(THF) level of theory.

Secondly, the effect of the lithium counter ion (**4** vs **4-Li**) was similarly shown to have minimal effect (Figure S3).

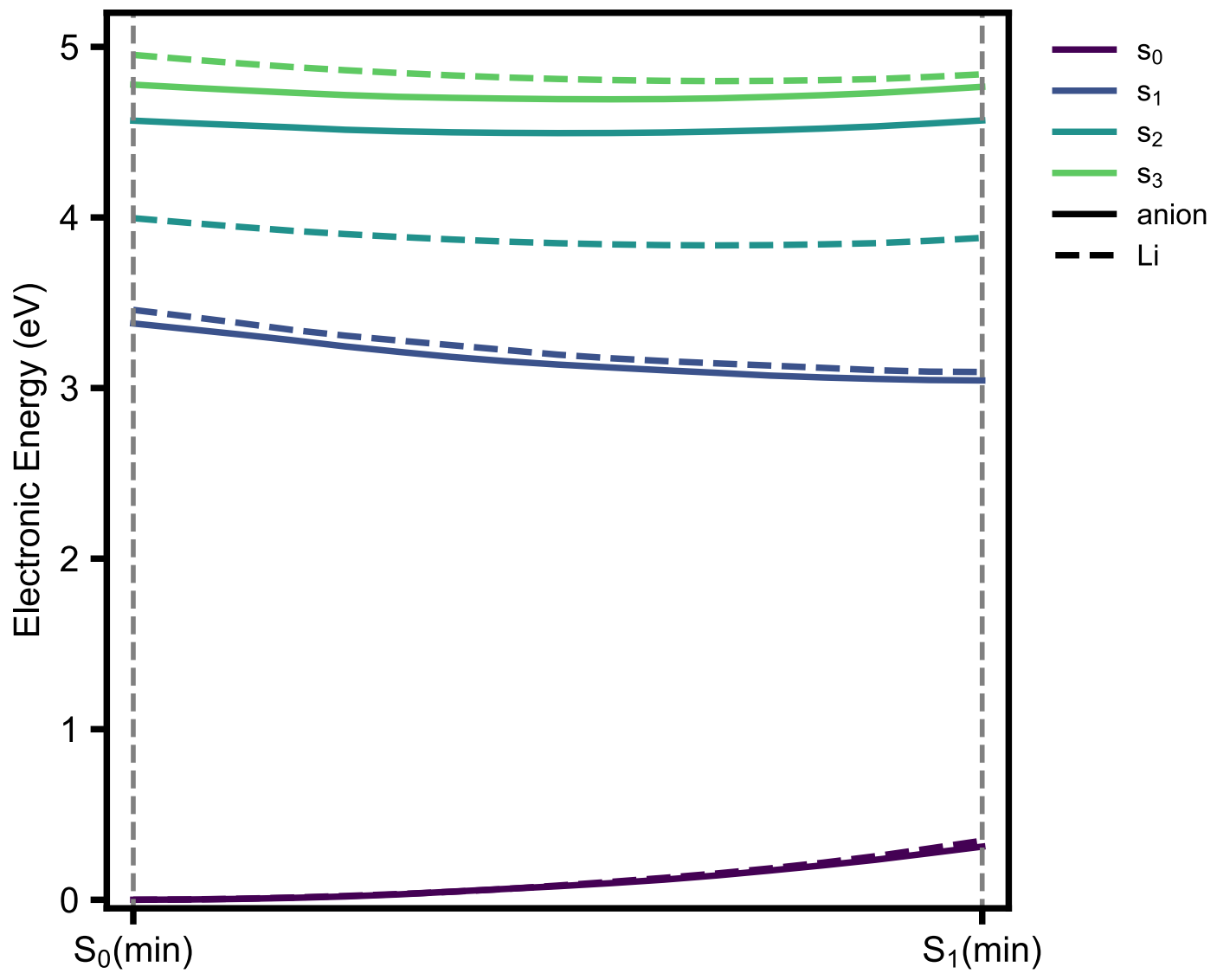

Figure S3. Geodesic interpolation from the ground state minimum energy geometry to the first excited state minimum energy geometry optimized with the ωB97X-D4/cc-pVDZ/C-PCM(THF) level of theory for both the anion 4 (solid line) and its lithium-ion pair (dashed line) calculated at the ωB97X-D4/cc-pVDZ/C-PCM(THF) level of theory.

1.6. Comparing RI-CC2 and LR-TDDFT/TDA/ωB97X-D4 beyond the Franck-Condon point

To compare the performance of both RI-CC2 and (LR-TD)DFT/TDA/ωB97X-D4 on the excited and ground state manifolds, electronic energies along paths were calculated based on the gas-phase optimised structures of **4**. For the excited-state region, geodesic interpolations were used and for the ground state, single optimized geometries were used. Figures S4 and S5 show that the overall behaviour of the first excited electronic state with RI-CC2 along the geodesic pathway is well mimicked by LR-TDDFT/TDA/ωB97X-D4 (up to a nearly rigid energy shift). RI-CC2 also reproduces the avoided crossing between S_1 and S_2 observed with LR-TDDFT/TDA/ωB97X-D4.

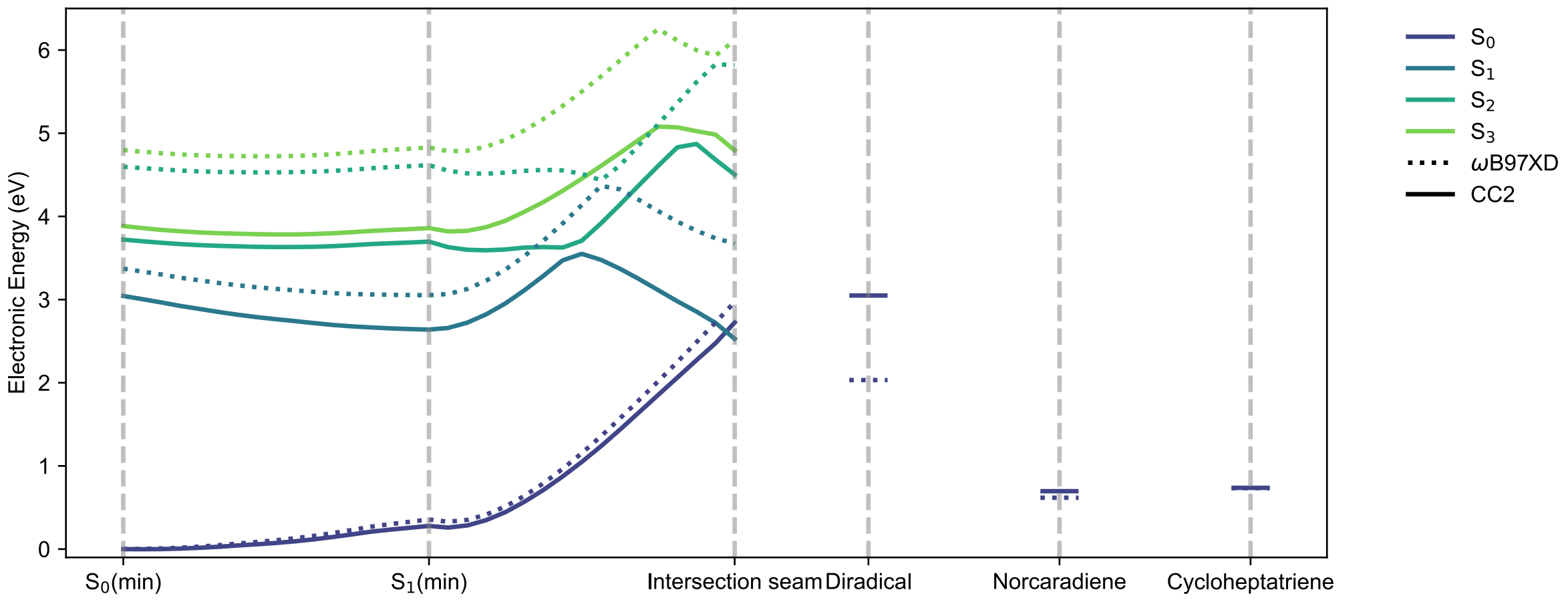

Figure S4. Geodesic interpolations of 4 at the ωB97X-D4/cc-pVDZ and RI-CC2/cc-pVDZ levels of theory with solvent effects (C-PCM for LR-TDDFT/TDA and COSMO for RI-CC2). All geometries were optimised with ωB97X-D4/cc-pVDZ in the gas phase.

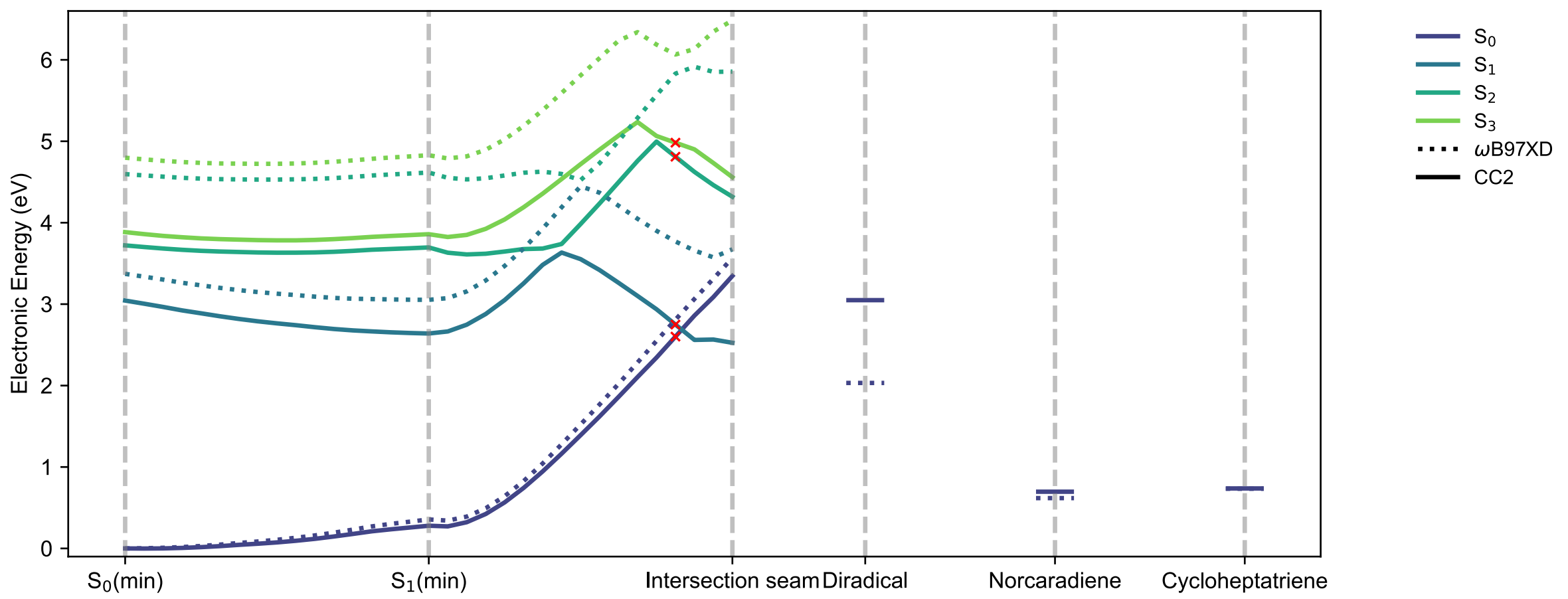

Figure S5. Geodesic interpolations of 4 at the ωB97X-D4/cc-pVDZ and RI-CC2/cc-pVDZ levels of theory with solvent effects (C-PCM for LR-TDDFT/TDA and COSMO for RI-CC2). All geometries were optimised with ωB97X-D4/cc-pVDZ in the gas phase, except for the intersection point, which was reoptimized with solvent effects (C-PCM[THF]). Red crosses indicate geometries along the geodesic path where an electronic energy could not be fully converged (energy shown is the mid-point between two neighbouring points).

1.7. Monitoring the potential importance of triplet states

To monitor the potential influence of triplet states (and intersystem crossing) in the photodynamics of **4**, we calculated electronic energies along the interpolation path from the S_0 to the S_1 minimum-energy geometries, including triplet states. The energy separation between S_1 and the low-lying triplet states along this pathway indicates that triplet states are unlikely to be involved in the photochemistry of these systems (Figure S6), in particular when considering the small magnitude of the spin-orbit coupling matrix elements (SOCME) at the S_1 minimum-energy geometry between S_1 and both the T_1 and T_2 states (Table S5).

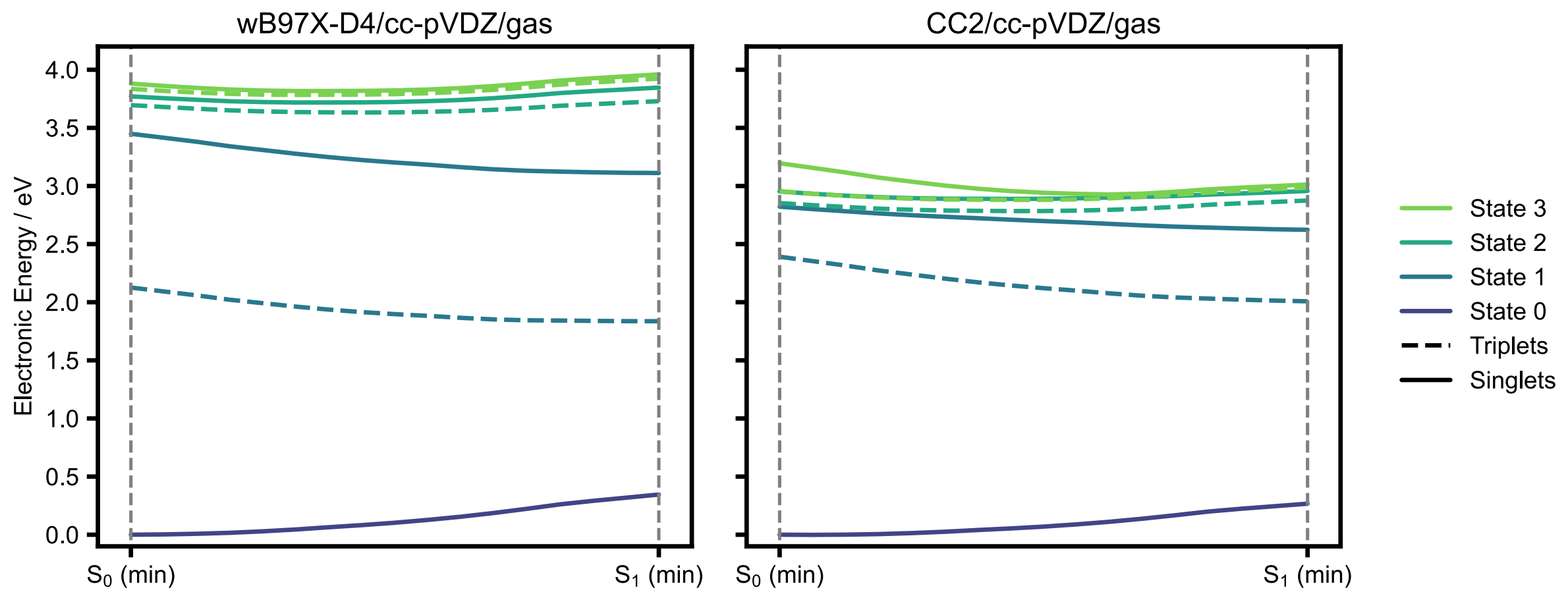

Figure S6. Geodesic interpolations from the S_0 minimum energy geometry to the S_1 minimum energy geometry of 4 (optimised at the ωB97X-D4/cc-pVDZ/gas level of theory), showing both the low-lying singlet and triplet electronic states, calculated at the ωB97X-D4/cc-pVDZ/gas (left) and RI-CC2/cc-pVDZ/gas (right) levels of theory.

Table S5. The magnitude of the spin-orbit coupling matrix elements (SOCME) between the S_1 and T_1/T_2 states of 4 at its S_1 minimum energy geometry (optimized with ωB97X-D4/cc-pVDZ/gas) calculated with ωB97X-D4/cc-pVDZ/gas.

| SOCME(S_1/T_1) | / cm^{-1}	0.13
| SOCME(S_1/T_2) | / cm^{-1}	0.80

1.8. Probing the intersection seam with mixed-reference spin-flip TDDFT

To probe the quality of the intersection seam provided by LR-TDDFT/TDA and RI-CC2, we used MRSF-TDDFT (mixed-reference spin-flip TDDFT) on the support of the LR-TDDFT/TDA geodesic interpolation pathways.[10] This analysis confirmed the presence of an intersection between S_1 and S_0, and the evolution of the character to a diradical in the ground electronic state following passage through the intersection.

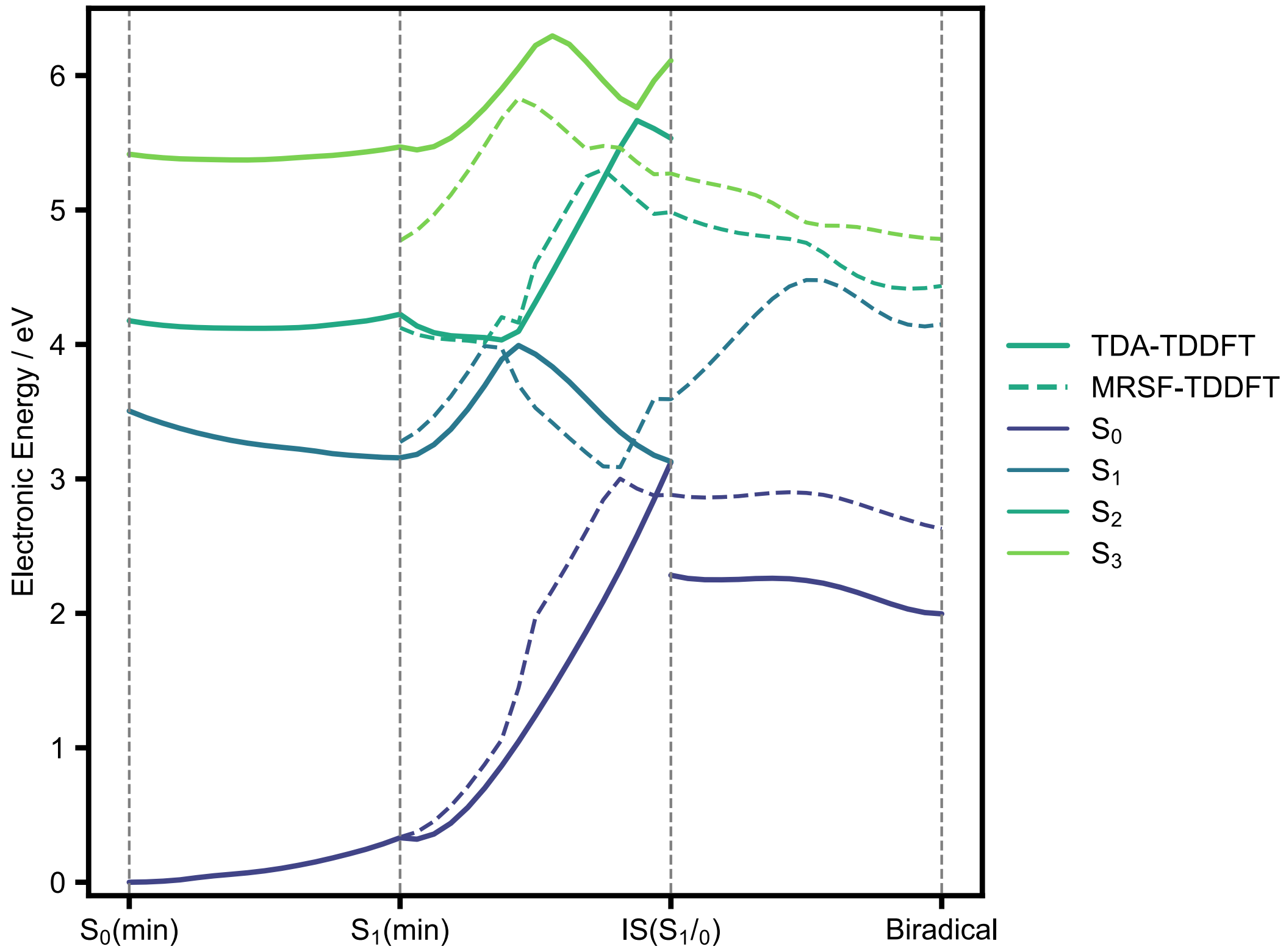

Figure S7. Geodesic interpolations of vinylbenzamide anion (A) from the ground state minimum energy geometry to the biradical modelled with LR-TDDFT/TDA (ωB97X-D4/cc-pVDZ/gas) and MRSF-TDDFT (ωB97X-D3/cc-pVDZ/gas). All geometries optimised at the gas phase with ωB97X-D4/cc-pVDZ/gas, biradical optimised using a UDFT basis.

1.9. Photoabsorption (UV-Vis) spectra

Theoretical photoabsorption cross sections were calculated with the Nuclear Ensemble Approach (NEA) as implemented in the Newton-X code.[6] 500 geometries were sampled from a harmonic Wigner distribution built using the ground-state optimised structure (and corresponding vibrational frequencies) obtained at the ωB97X-D3/cc-pVDZ level of theory with Gaussian 16. Based on these geometries, excitation energies and oscillator strengths were calculated at the ωB97X-D4/cc-pVDZ level of theory, both with and without implicit solvation (C-PCM), and RI-CC2/cc-pVDZ levels of theory. A Gaussian line broadening σ = 0.05 eV was applied to each vertical excitation.

The NEA can provide a better estimate of the photoabsorption cross-section in comparison to a vertical excitation from an optimised ground-state geometry. By sampling geometries from an approximate ground-state nuclear probability density, the NEA describes more accurately the width of each band of an absorption spectrum and includes non-Condon effects.[11, 12]

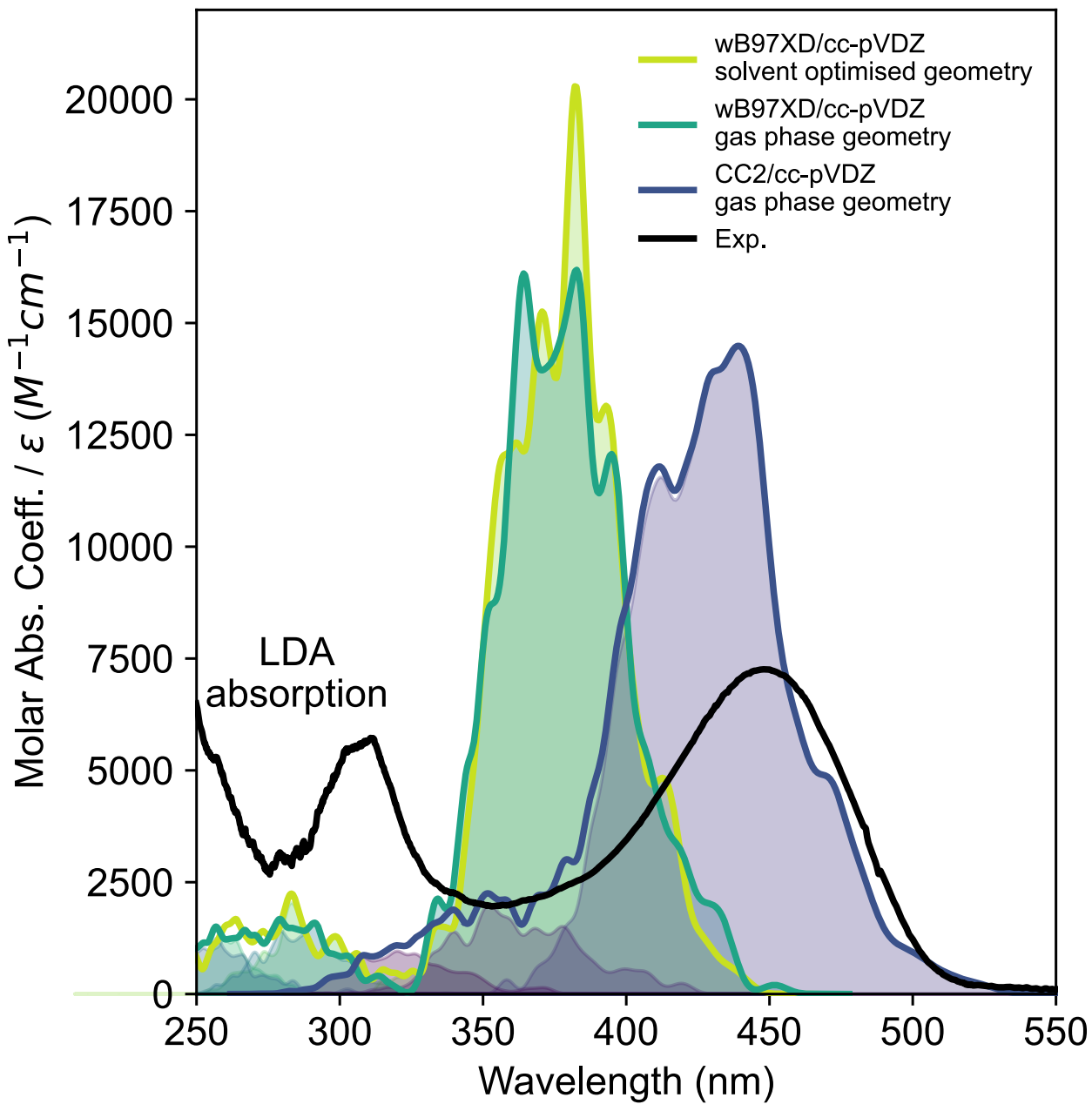

Figure S8. Simulated photoabsorption cross section calculated from 500 geometries sampled from a harmonic Wigner distribution constructed from the ground state optimized geometry obtained with ωB97X-D4/cc-pVDZ in both the gas phase (green) and solvent phase (yellow), vertical excitations are calculated with the same level of theory. Also plotted are the same theoretical spectra calculated at the RI-CC2/cc-pVDZ level of theory on the RI-CC2/cc-pVDZ optimized gas phase geometry (purple) and the experimental spectra (black) which includes absorptions in the 250-350 nm range due to the presence of excess base (LDA) in the sample. The theoretical spectra have been deconvoluted to show contributions from the first three excited state transitions.

1.10. Norcaradiene vs cycloheptatriene [CCSD(T)]

The final step in the photochemical reaction of **2-Li** is the formal 6π electrocyclic ring opening of the norcaradiene to form the cycloheptatriene.[13] According to ground state DFT calculations (ωB97X-D4/cc-pVDZ), both with and without implicit solvent, this transformation appears unfavourable. However, when performing the reaction, it is the latter structure that is isolated rather than the norcaradiene (except for a single example – see the main text). To investigate the cause of this discrepancy, we conducted high-level coupled cluster calculations on these systems. The result is shown in Figure S9 and clearly shows the CCSD(T)/cc-pVDZ/gas suggests a favourable transformation.

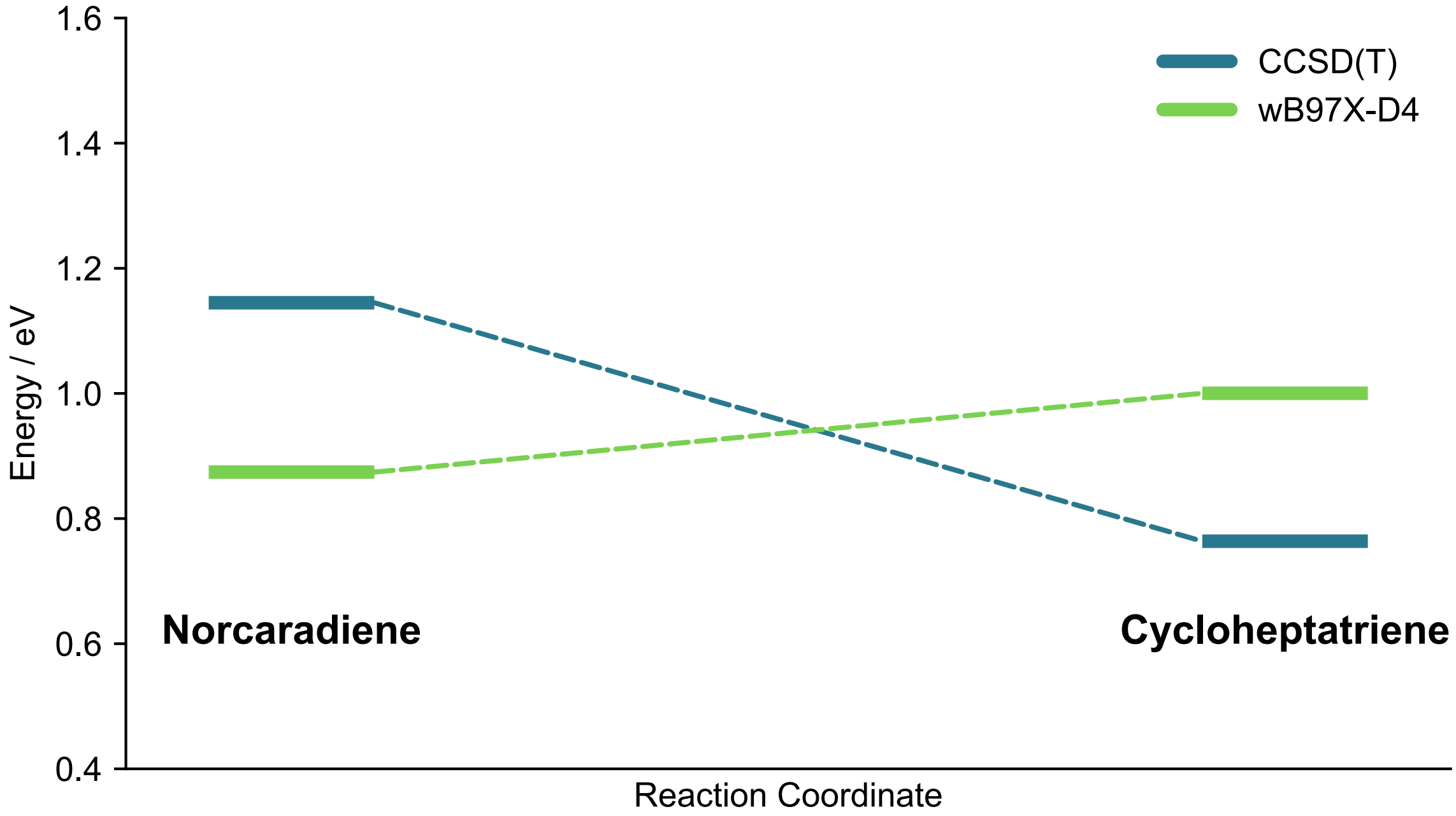

Figure S9. Energies of the final two species (anions) of the interpolation pathway presented in the main text, calculated at both the ωB97X-D4/cc-pVDZ/gas and CCSD(T)/cc-pVDZ/gas levels of theory. Geometries were optimized in the gas phase with ωB97X-D4/cc-pVDZ.

2. Experimental

2.1. Materials

All starting materials were obtained from commercial suppliers unless otherwise stated. Anhydrous THF and CH_2Cl_2 were dried on an Anhydrous Engineering activated alumina column before being stored in an atmosphere of nitrogen. Deuterated solvents, $CDCl_3$ and DMSO-d_6 were used directly as purchased.

2.2. Methods

For all reactions requiring anhydrous conditions (as specified) were executed using standard Schlenk line techniques under an atmosphere of nitrogen using oven-dried glassware. Air and moisture sensitive reagents and solutions were transferred *via* syringe. Where thin layer chromatography (TLC) was employed to monitor reaction progress, Merck TLC silica gel 60 F254 plates were used and were visualised using UV light (254 nm), often with staining either in prepared solutions of $KMnO_4$ (general) or I_2 (diene or trienes). Normal phase flash column chromatography was carried out using a Biotage Isolera Four system using Biotage Sfär Duo pre-packed silica gel columns, with solvent systems specified.

2.3. Instrumentation

NMR: ^{1}H NMR spectra were measured at 400 MHz on a Bruker Avance 400, Varian 400-MR, Jeol ECZ400 or Jeol ECS400 or at 500 MHz on a Bruker Avance 500. Corresponding ^{13}C frequencies are 101 MHz and 126 MHz. 1D NOE spectra were recorded on a Varian 500 MHz spectrometer. NMR samples were analysed as solutions with solvents specified below and, unless otherwise stated, at 298 K. ^{1}H and ^{13}C {^{1}H} NMR spectra were referenced to residual solvent peaks ($CDCl_3$ δ_H 7.26 and δ_C 77.2; DMSO-d_6 δ_H 2.50 and δ_C 39.5).

Chemical shifts (^{1}H and ^{13}C) are reported in parts per million (ppm) relative to residual solvent. Coupling constants (*J*) are reported to the nearest 0.1 Hz (^{1}H NMR) and multiplicities are defined as: s (singlet), d (doublet), t (triplet), q (quartet), m (multiplet) and combinations thereof. Assignments are stated throughout. However, some assignments are not possible where considerable overlap of spectral peaks exist (mainly with aromatic signals).

HRMS: High resolution mass spectra were recorded on a Bruker Daltronics MicrOTOF 2 mass spectrometer (ESI).

Melting Points: Expressed in °C, were measured on a Stuart SMP30 melting point apparatus and are uncorrected.

IR: IR spectra were recorded on a Perkin Elmer (Spectrum One) FT-IR spectrometer (ATR sampling accessory). Only strong and selected absorbances (expressed in cm^{-1}) are reported.

UV-Vis: UV-visible spectra were recorded on an Agilent Cary Series 300 UV-Vis Spectrophotometer using a baseline correction for the solvent used in the sample or using a custom UV-Vis probe as described below.

Cryostat: All low temperature reactions (below 0 °C) were carried out using either a Huber TC100E or a Thermo Scientific EK90 Immersion Cooler using a solvent bath of methanol unless otherwise stated.

Microwave: A Biotage® Initiator+ microwave system was used for microwave reactions using a sealed tube, with pre-stirring of the mixture for 30 seconds.

2.4. UV-Visible Spectroscopy Measurements

To an oven-dried 5 mm cuvette was attached a septum secured with Parafilm®. The cuvette was swing-purged using a Schlenk line. The cuvette was placed in an ice bath. In the ice bath, a solution of enolate (formed from the corresponding amide and deprotonation with two equivalents of LDA) in anhydrous THF was added by syringe. The cuvette was kept in an ice bath until running the spectrum.

A blank sample containing anhydrous THF was run before UV-vis measurements.

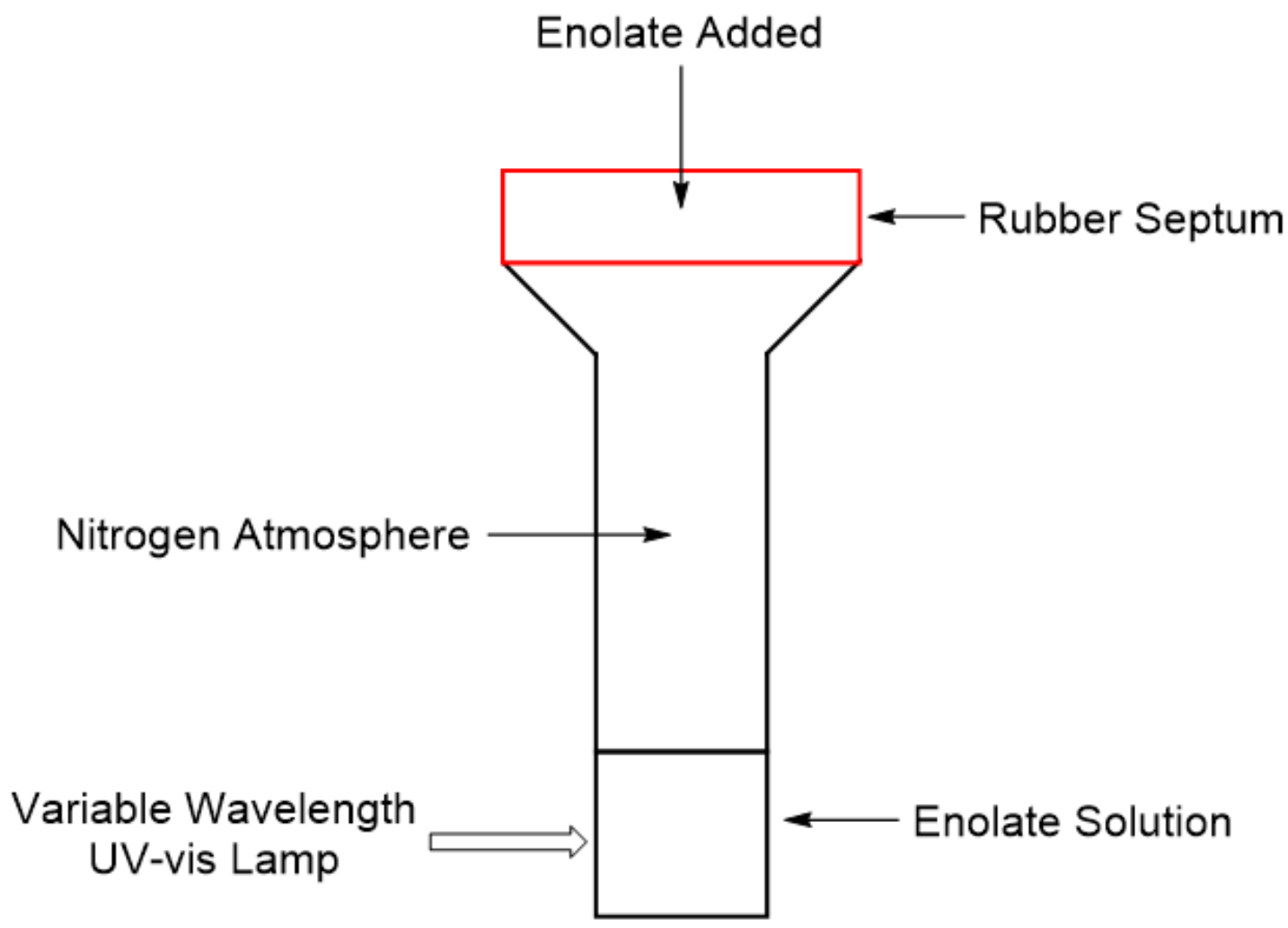

For the experimental spectrum of enolate **4** as shown in the main text (Figure 3), a custom-built probe was used to record UV-Vis measurements. Due to its highly moisture and air sensitivity, and to record accurate molar absorption coefficients (ξ), *in-situ* measurements at a range of concentrations were obtained.

The experimental setup consisted of a ThorLabs stabilised deuterium UV-Vis source (SLS204) connected to one side of an Ocean Optics 300-micron Transmission dip probe (TP300-UV-VIS) with Adjustable path (2-10 mm) tip (RTP-2-10), the other side was then connected to a ThorLabs compact CMOS spectrometer (CCT10). The transmission dip probe was then inserted into a flame dried, 3-necked round bottom flask and sealed with a rubber bung. To the flask was also connected a gas inlet and a rubber septum before being swing purged onto a Schlenk line *via* the gas inlet (complete setup shown in Figure S10).

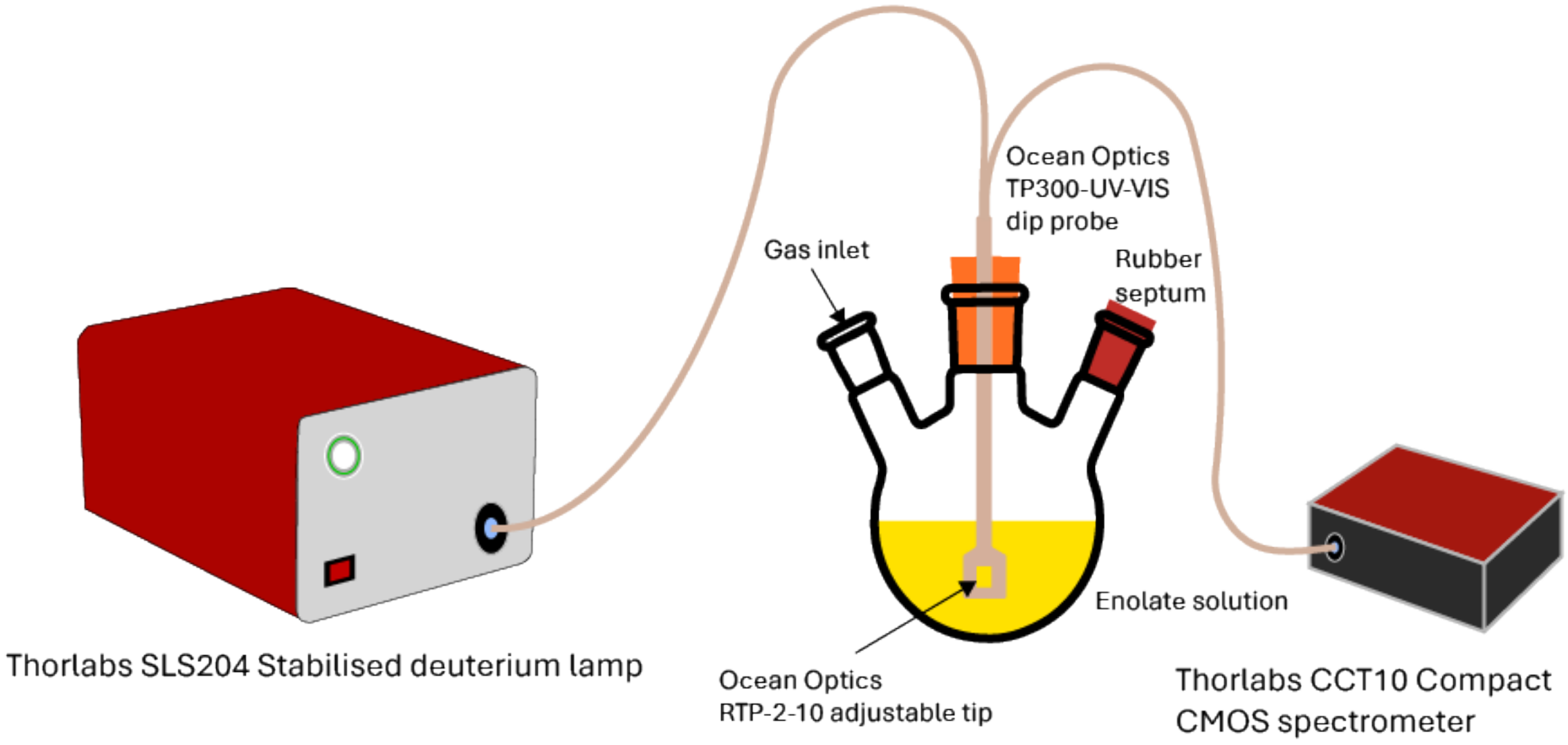

Figure S10. ***In-situ*** **UV-Vis measurement set-up consisting of a Thorlabs UV source, Ocean Optics transmission dip probe and a Thorlabs CMOS spectrometer. The probe is secured using an airtight rubber bung inside a three-necked flask, to this is also attached a gas inlet to cycle onto a Schlenk line and a rubber septum for addition of solutions.**

To the flask was added a suitable volume of THF to ensure the dip probe was fully submerged, a reference spectrum was obtained of the solvent. Diisopropylamine (5 equiv.) was dissolved in the solution which was then cooled to -78 °C before addition of n-BuLi (5.5 equiv.). The solution was warmed to room temperature before cooling again to -78°C. A solution of pre-cyclised benzamide **B** was then added to the solution and stirred for 15 minutes before recording a UV-Vis spectrum. Spectra were recorded with a 7 ms exposure time and averaged over 1000 scans. The molar absorption coefficient was thus obtained by plotting the concentration of the solution against the absorbance of the solution at 449.27 nm (Figure S9):

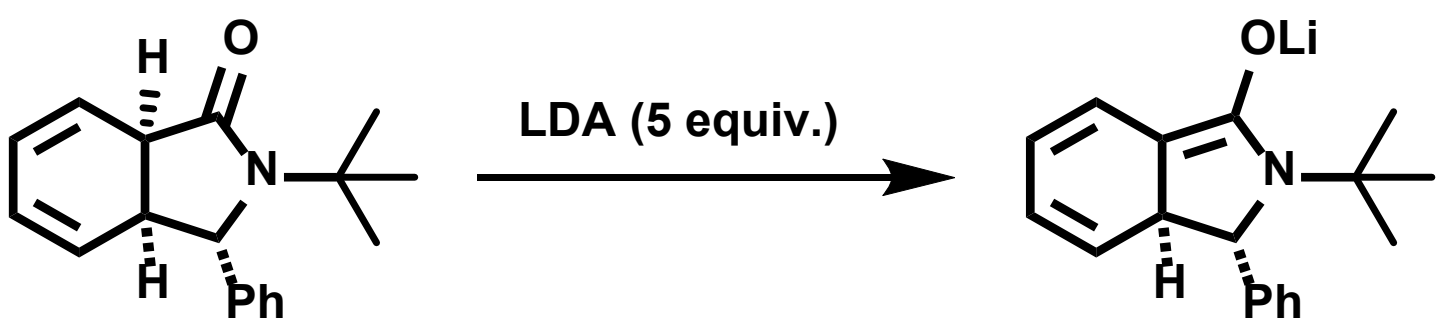

B **B-Li**

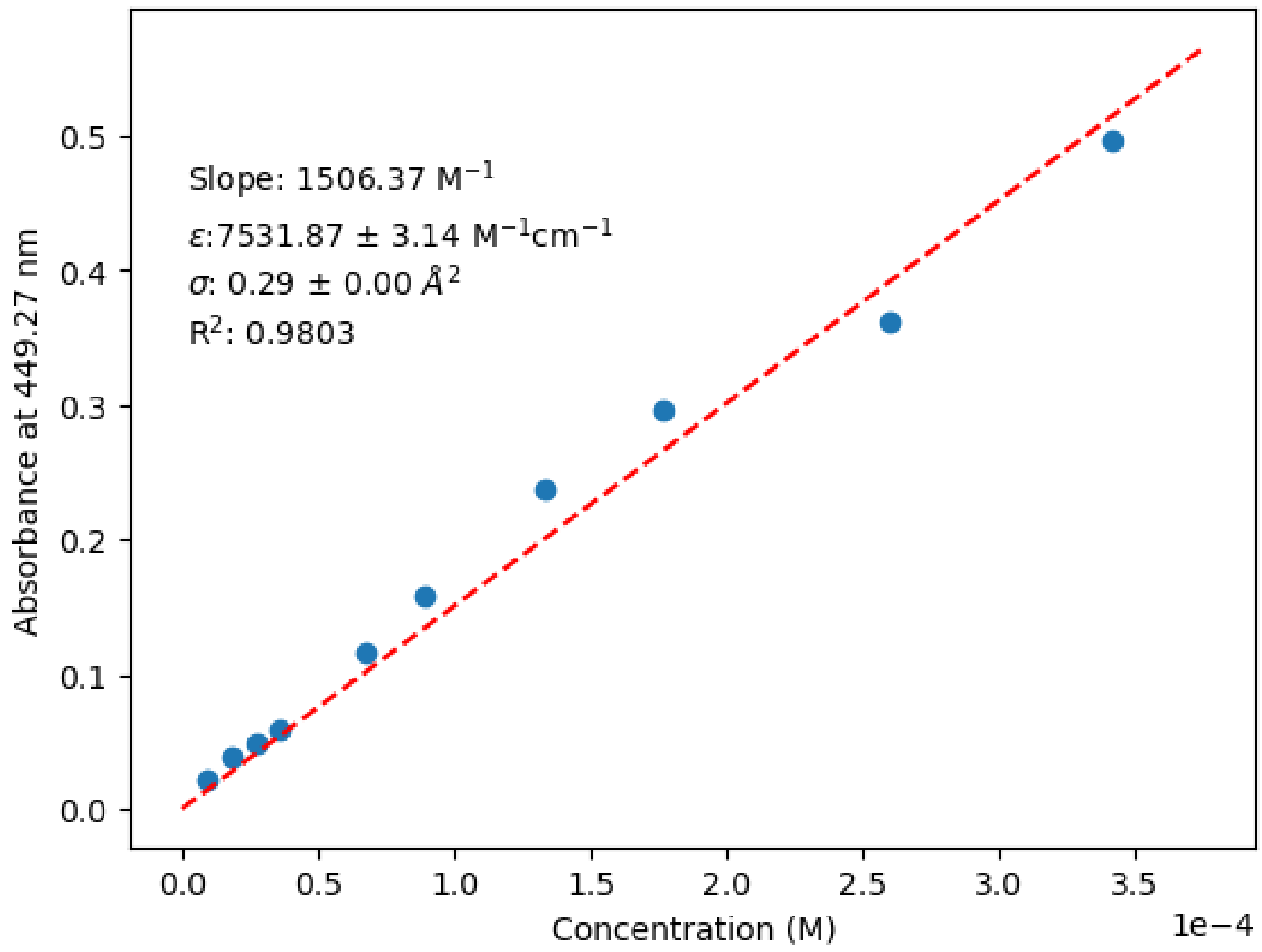

Figure S11: Absorbance (y-axis) against concentration (x-axis) for a solution of B-Li, prepared and measured in-situ *via* the deprotonation of B with 5 equivalents of LDA as described above.

2.5. Photochemistry set-up

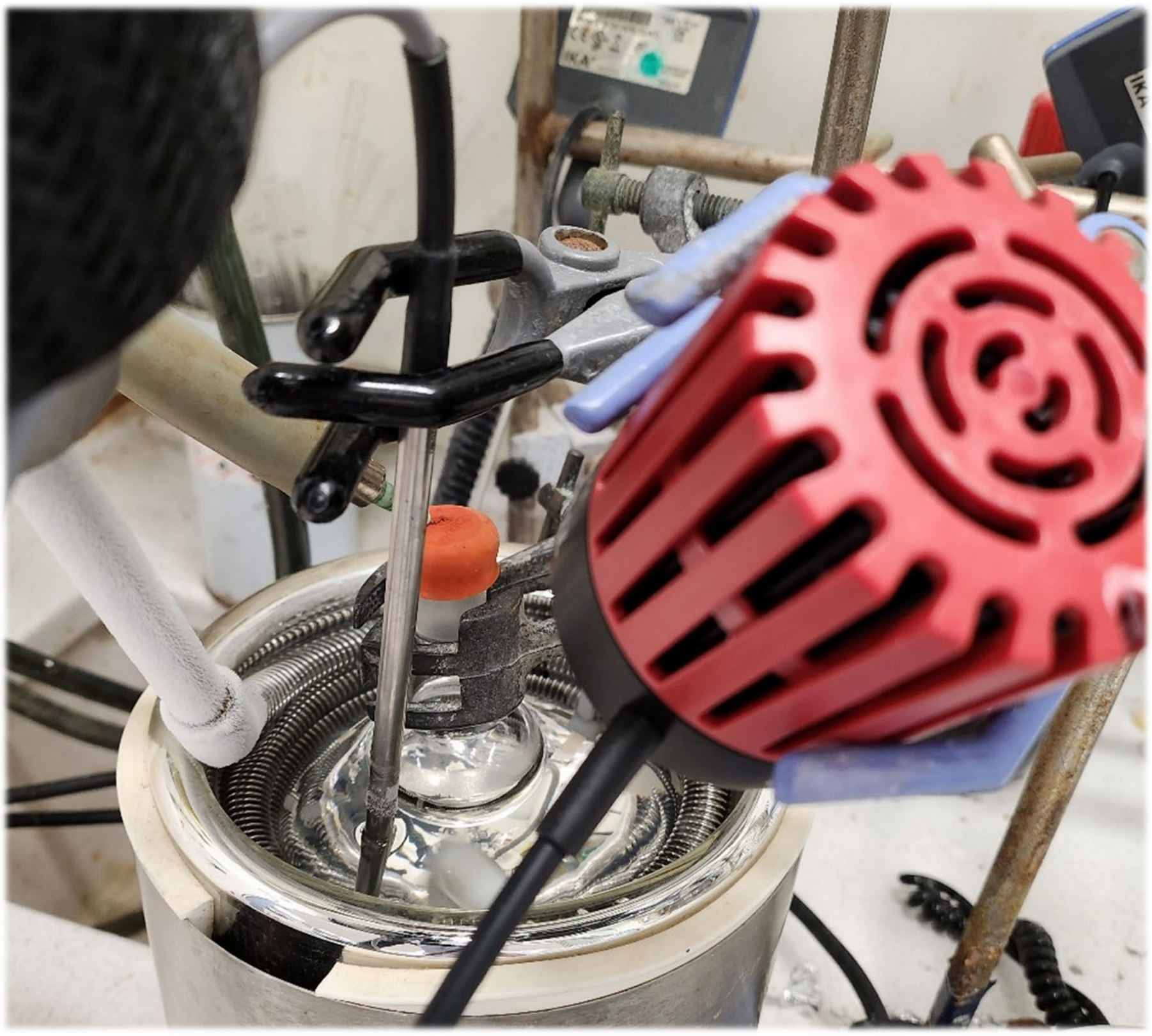

Figure S12. Showing the setup used for photochemical reactions in this work.

The Cryostat was attached to a Dewar containing methanol with a temperature probe in the solution adjacent to the reaction flask. The LED was clamped above the mixture, giving a clear line of consistent irradiation onto the mixture.

If the glassware of the reaction becomes cloudy with condensation, methanol was run down the sides to clear it, allowing for better light penetration. The reaction was carried out with consistent flow of nitrogen through a needle and septum.

The LED is powered by either a Thorlabs T-Cube^{TM} LED driver or a Thorlabs DC4100 four-channel LED driver (below) at the maximum LED current, where possible.

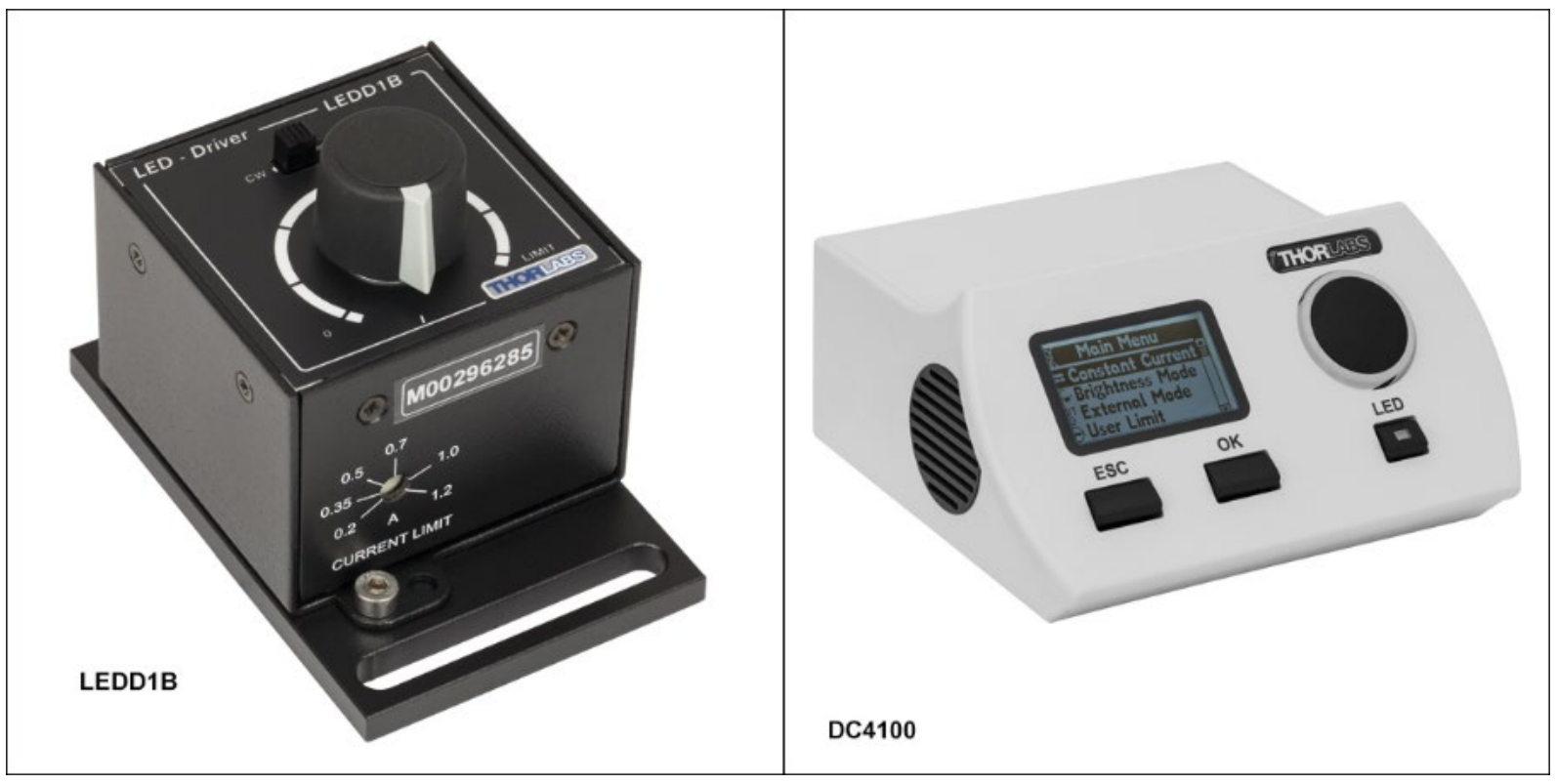

The Thorlabs LEDs were used with an attached aspheric condenser lens (Ø 25 mm, coated for 350-700 nm).

2.6. LED Emission Data

Kessil® A160WE Tuna Blue: Blue-coloured LED with spectral tuning, shifting measured maximum emission (shown below). The 'blue-end' consists of maximum emission of 465 nm whereas the 'white-end' consists of a greater proportion of emission at 441 nm. When irradiation at 450 nm is reported in this work, the 'blue-end' was utilised. This LED has a 40 W rating with an attached power supply.

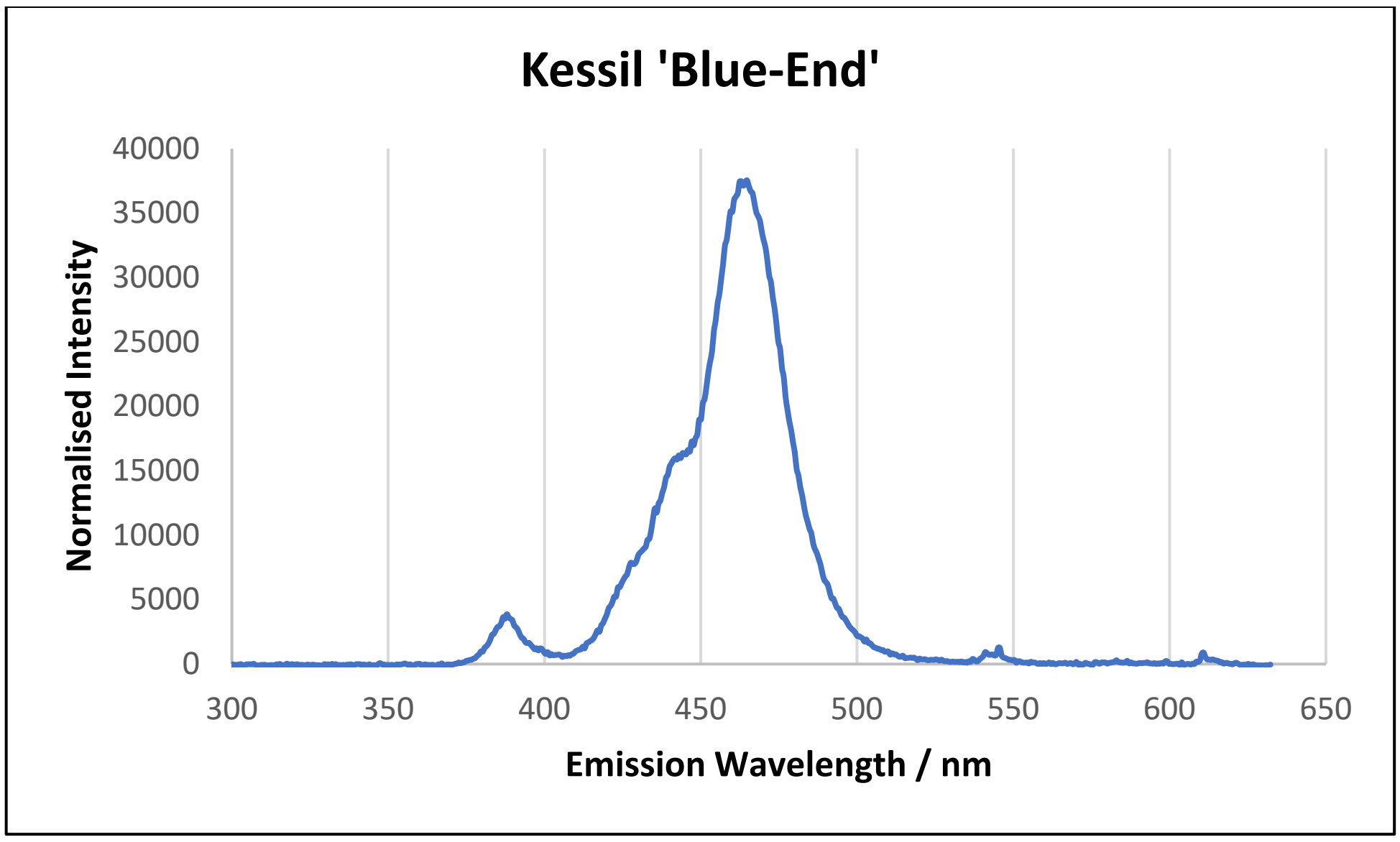

Figure S13. Emission spectrum of the Kessil lamp at it's 'blue-end'.

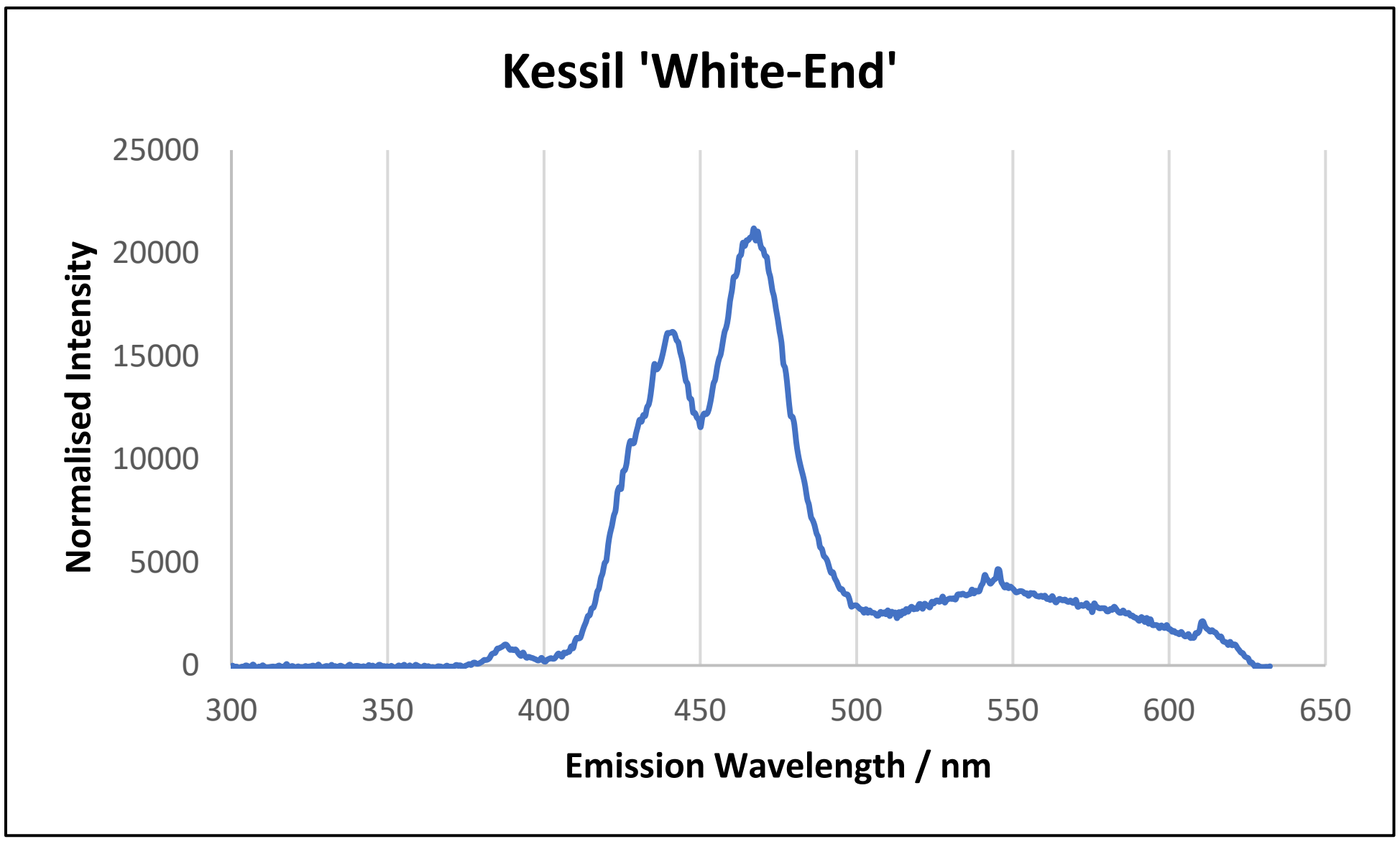

Figure S14. Emission spectrum of the Kessil lamp at it's 'white-end'

Thorlabs M365LP1: Mounted LED (UV-colour, 365 nm) with a power rating of 6.80 W. Measured emission spectra of this LED is shown below, with a maximum emission of 367 nm.

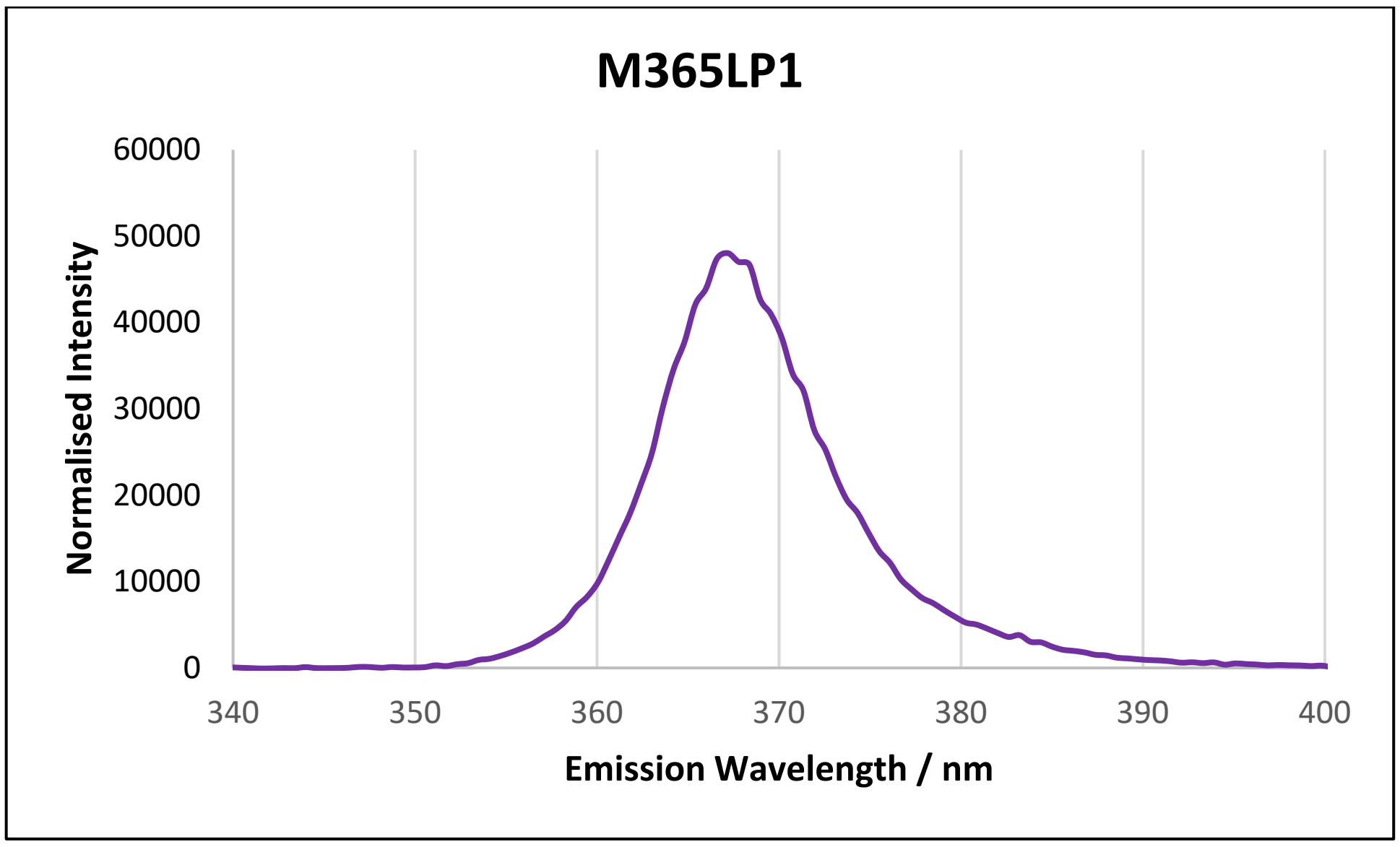

Figure S15. Emission spectrum of the Thorlabs M365LP1 lamp.

Thorlabs M385LP1: Mounted LED (UV-colour, 385 nm) with a power rating of 6.63 W. Measured emission spectra of this LED is shown below, with a maximum emission of 383 nm.

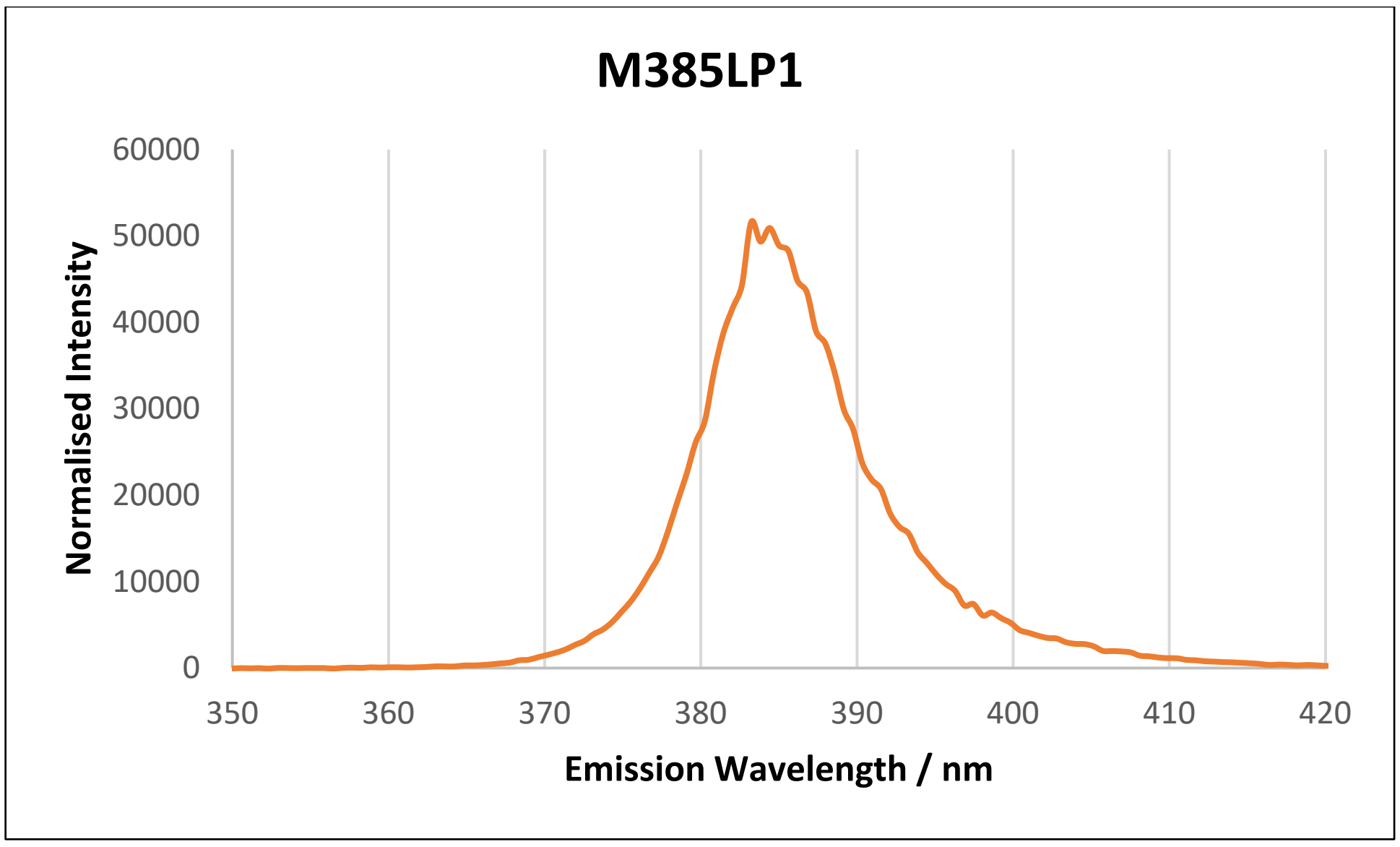

Figure S16. Emission spectrum of the Thorlabs M385LP1 lamp.

2.7. General Procedures

General Procedure 1: Amide Coupling Using HATU

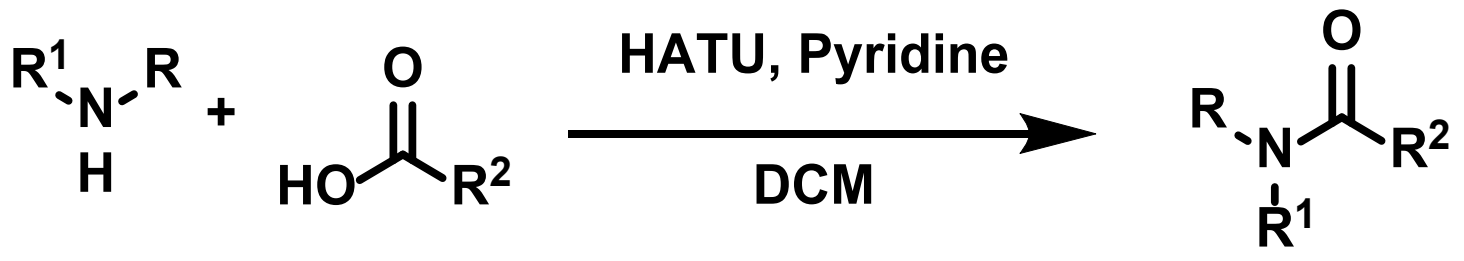

The corresponding carboxylic acid (1.0 eq.) was dissolved in CH_2Cl_2 (0.5 M) along with HATU (1.1 eq.) before addition of the respective amine (1.1 eq.). The mixture was stirred for 5 minutes before addition of pyridine (2.0 eq.) and the mixture stirred at room temperature for 16 hours. $NaHCO_{3(aq.)}$ (60 mL) was added and the layers separated. The aqueous layer was extracted with CH_2Cl_2 (2 × 40 mL) before the combined organics were washed with brine (60 mL), dried with $MgSO_4$, filtered and concentrated *in vacuo*. Purification by flash column chromatography afforded the amide product.

General Procedure 2: Photochemistry on Acyclic Enolates using LDA

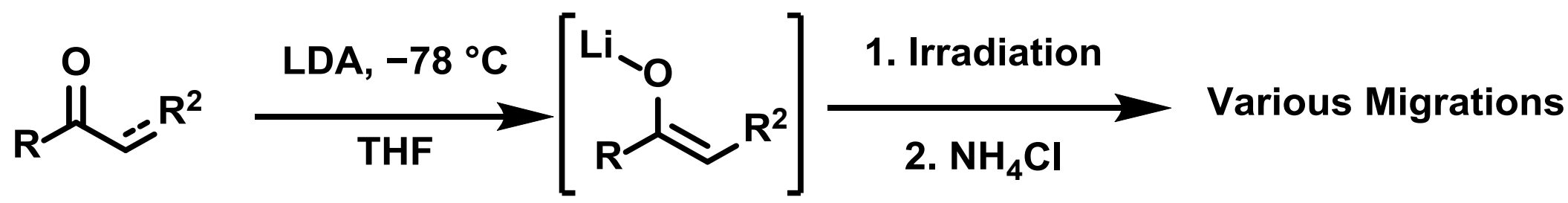

DIPA (2.0 eq.) was dissolved in anhydrous THF (5 mL) before dropwise addition of ^{n}BuLi (2.1 eq., 2.5 M in hexanes) at −78 °C. A solution of amide (1.0 eq.) in anhydrous THF (0.5 M) was added to the basic solution at −78 °C to give a coloured enolate solution. Irradiation was started at 385 nm (Thorlabs M385LP1) for 5 hours before quenching with $NH_4Cl_{(aq.)}$ (10 mL). The layers were separated at room temperature before the aqueous layer was extracted with EtOAc (3 × 10 mL) and the combined organics were washed with brine (10 mL), dried with Na_2SO_4, filtered and concentrated *in vacuo*. Purification by flash column chromatography afforded the respective photochemical products.

General Procedure 3: Lactam Cyclisation under Microwave Conditions

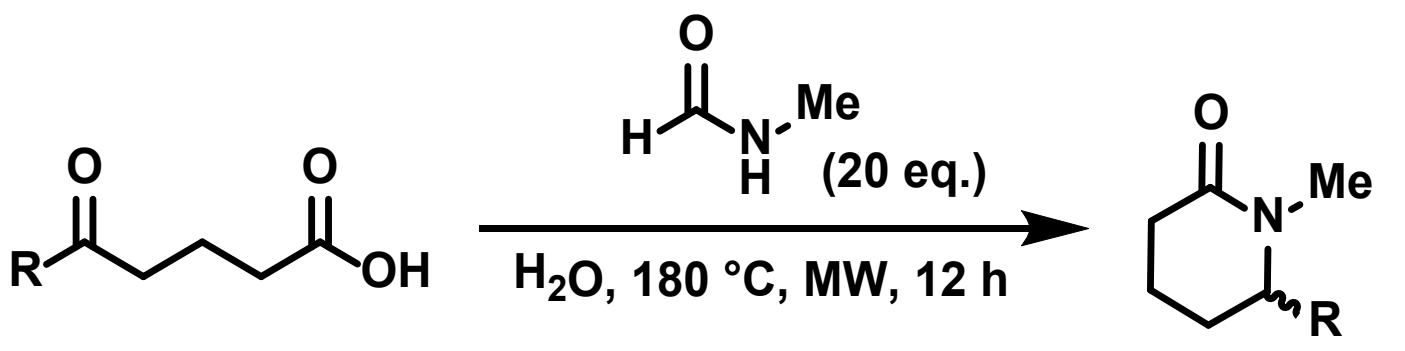

Synthesised according to a modified procedure by Li *et al.*[14]

To a 10-20 mL microwave vial was added the corresponding keto acid (1.0 eq.), H_2O (2 mL) and *N*-methyl formamide (20.0 eq.). A small amount of H_2O (1 mL) was used to wash the walls of the vial before the vial was placed under microwave conditions (30 s pre-stirring, 180 °C, 12 h, high absorption setting). After cooling to rt, H_2O (10 mL) was added and the aqueous layer extracted with CH_2Cl_2 (3 × 10 mL) before the combined organics washed with brine (10 mL), dried over $MgSO_4$, filtered and concentrated *in vacuo*. Purification by flash column chromatography afforded the lactam product.

General Procedure 4: Aldol Condensation for Dienyl Lactams

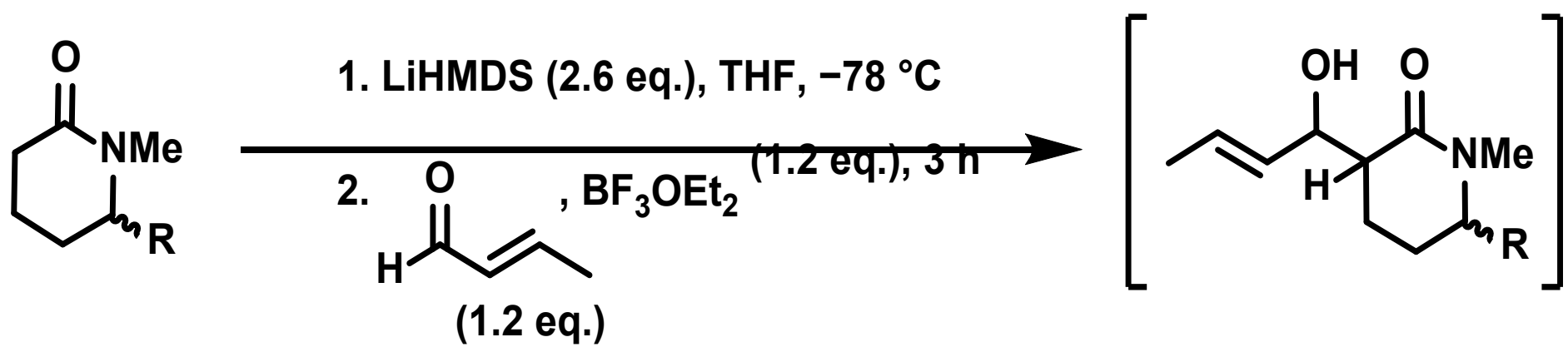

The respective lactam (1.0 eq.) was dissolved in anhydrous THF (0.25 M) before slow addition of LiHMDS (2.6 eq., 1 M in THF) at −78 °C. After stirring at this temperature for 1 h, a solution of crotonaldehyde (1.2 eq.), BF_3OEt_2 (1.2 eq.) and anhydrous THF (15 mL) was added slowly at −78 °C. The mixture was left to stir for 3 h at this temperature before being quenched with saturated $NH_4Cl_{(aq.)}$ (30 mL) and water (30 mL). The aqueous layer was extracted with EtOAc (3 × 40 mL) before the combined organics were washed with water (2 × 30 mL), brine (2 × 30 mL), dried over $MgSO_4$, filtered and concentrated *in vacuo*. The crude mixture was used as a mixture of diastereomers in the next step without further purification.

General Procedure 5: Elimination of Alcoxy Lactams to Alkenyl Lactams

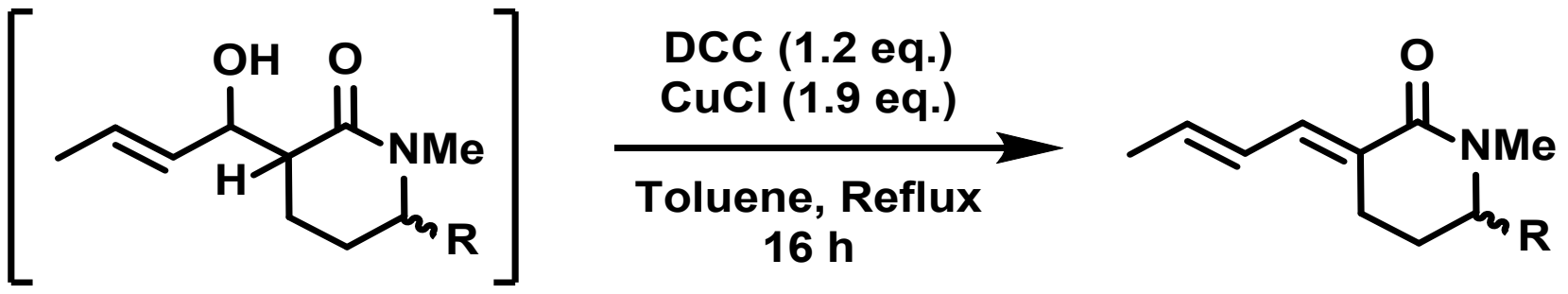

Synthetic procedure adapted from Santos *et al.*[15]

To an oven-dried flask was added a solution of α-alcoxy lactam (1.0 eq.) in anhydrous toluene (0.4 M) before addition of DCC (1.2 eq.) and CuCl (1.9 eq.). The mixture was heated to reflux for 16 h before slow addition of 30% $NH_{3(aq.)}$ (30 mL). The aqueous layer was extracted with EtOAc (5 × 30 mL) before the combined organics organics were washed with brine (2 × 50 mL), dried over $MgSO_4$, filtered and concentrated *in vacuo*. Purification by flash column chromatography afforded the product as a mixture of isomers.

General Procedure 6: Photochemical Ring-Contraction of Lactam Trienolates

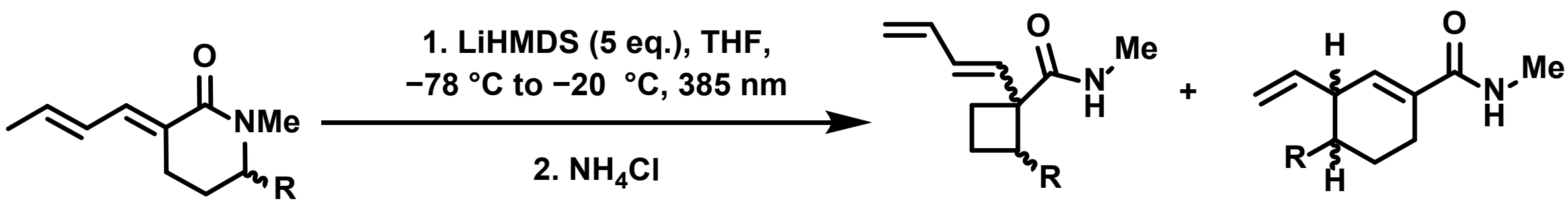

The respective lactam (1.0 eq.) was dissolved in anhydrous THF (0.05 M) before slow addition of LiHMDS (5.0 eq., 1 M in THF) at −78 °C with irradiation at 385 nm (Thorlabs M385LP1). After 1 h at this temperature, the mixture was allowed to slowly warm to −20 °C. After 5 h of total irradiation, the mixture was quenched with saturated $NH_4Cl_{(aq.)}$ (10 mL) before the aqueous layer was extracted with EtOAc (3 × 10 mL). The combined organics were washed with brine (10 mL) before being dried over Na_2SO_4, filtered and concentrated *in vacuo*. Purification by flash column chromatography gave the photochemical products. When n = 1, only the 1,3 ring-contraction product is seen (left product above). When n = 2, both photochemical products (1,3 and 1,5), shown above, were isolated.

2.8. Experimental Procedures

N-benzyl-2-methylpropan-2-amine

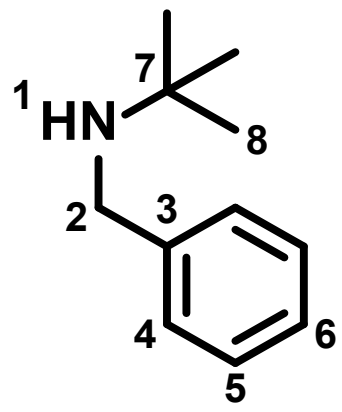

Synthesised according to the procedure by Clayden *et. al.*[13]

Tert-butylamine (0.2 mol, 1.0 eq.) and benzaldehyde (0.2 mol, 1.0 eq.) were dissolved in MeOH (500 mL) and the resulting solution was stirred for 4 hours. $NaBH_4$ (0.24 mol, 1.2 eq.) was added portion wise at 0 °C and the mixture stirred for 15 minutes before being allowed to warm to room temperature. After 2 hours, solvent was removed *in vacuo* before addition of water (300 mL) and CH_2Cl_2 (200 mL), and the layers separated. The aqueous layer was further extracted with CH_2Cl_2 (2 × 100 mL), and the combined organic phase was washed with brine (60 mL), dried with $MgSO_4$, filtered and concentrated *in vacuo* to give the title compound as a yellow oil (0.19 mol, 92%). No further purification was carried out.

^{1}H NMR (400 MHz, Chloroform-*d*): δ 7.41-7.32 (4H, m, H4-5), 7.29-7.24 (1H, m, H6), 3.78 (2H, s, H2), 1.23 (9H, s, H8).

^{13}C NMR (101 MHz, Chloroform-*d*): δ 141.5 (C3), 128.4 (C4), 128.2 (C5), 125.6 (C6), 51.5 (C7), 46.7 (C2), 29.1 (C8).

Data consistent with literature.[13]

(*E*)-hexa-3,5-dienoic acid

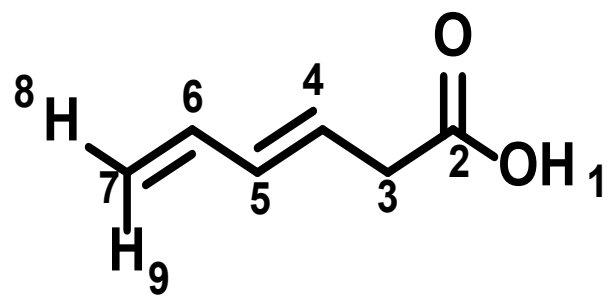

Synthesised according to the procedure by Mestres *et. al.*[16]

To a solution of DIPA (53.55 mmol, 2.0 eq.) in anhydrous THF (15 mL) was added ^{n}BuLi (56.22 mmol, 2.1 eq., 2.5 M in hexanes) at −78 °C. A solution of 2*E*,4*E*-hexadienoic acid (26.77 mmol, 1.0 eq.) in anhydrous THF (30 mL) was added to the base solution to give an orange cloudy mixture at −78 °C. The solution was allowed to warm to −20 °C gradually for 2.5 hours before being quenched with $NH_4Cl_{(aq.)}$ (50 mL). The layers were separated before the aqueous layer was acidified until pH ~ 2 using 3M $HCl_{(aq.)}$ and extracted with EtOAc (3 × 40 mL). The combined organics were dried with $MgSO_4$ before being filtered and concentrated *in vacuo* to give an orange oil as the title compound (26.77 mmol, quant.).

^{1}H NMR (400 MHz, Chloroform-*d*): δ 9.43 (1H, br s, H1), 6.33 (1H, dt, *J* = 17.1, 10.1 Hz, H6), 6.15 (1H, dd, *J* = 15.2, 10.7 Hz, H5), 5.77 (1H, dt, *J* = 15.2, 7.5 Hz, H4), 5.17 (1H, dd, *J* = 16.7, 1.1 Hz, H9), 5.07 (1H, dd, *J* = 10.0, 1.1 Hz, H8), 3.15 (2H, d, *J* = 7.3 Hz, H3).

^{13}C NMR (101 MHz, Chloroform-*d*): δ 177.7 (C2), 136.3 (C6), 134.8 (C5), 125.1 (C4), 117.3 (C7), 37.9 (C3).

HRMS (ESI^{-}) *m/z* calcd for $C_6H_7O_2$ [M−H]$^{-}$ 111.0446, found 111.0451.

IR: ν_{max} = 3087 (O-H), 3021 (C-H), 2974 (C-H), 1704 (C=O), 1652 (C=C), 1604 (C=C), 1410, 1273, 1219, 1002 cm^{-1}.

(*E*)-*N*-benzyl-*N*-(*tert*-butyl)hexa-3,5-dienamide (8a)

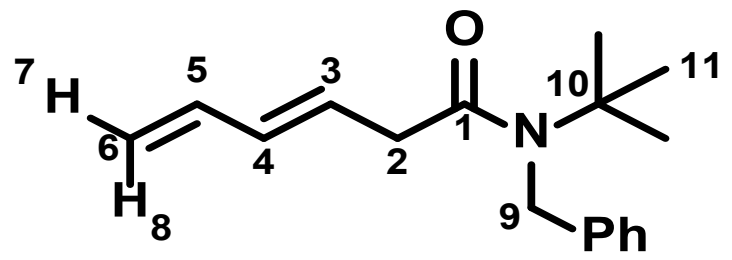

Synthesised according to General Procedure 1 on a 17.83 mmol scale to give a white solid (7.13 mmol, 40%), using (*E*)-hexa-3,5-dienoic acid and *N*-benzyl-2-methylpropan-2-amine as reagents. Purification was carried out with an eluent of 10% EtOAc / petroleum ether.

^{1}H NMR (400 MHz, Chloroform-*d*): δ 7.36 (2H, t, *J* = 6.9 Hz, Ar), 7.28-7.19 (3H, m, Ar), 6.33 (1H, dt, *J* = 17.0, 10.1 Hz, H5), 6.00 (1H, dd, *J* = 15.5, 10.5 Hz, H4), 5.86 (1H, dt, *J* = 15.7, 6.6 Hz, H3), 5.09 (1H, dd, *J* = 17.0, 0.8 Hz, H7), 5.00 (1H, dd, *J* = 10.4, 0.8 Hz, H8), 4.58 (2H, s, H9), 3.10 (2H, d, *J* = 6.4 Hz, H2), 1.43 (9H, s, H11).

^{13}C NMR (101 MHz, Chloroform-*d*): δ 174.5 (C1), 140.3 (Ar), 136.8 (C5), 134.7 (C4), 129.3 (Ar), 128.3 (Ar), 126.6 (C3), 125.6 (Ar), 116.3 (C6), 58.1 (C10), 49.2 (C9), 39.3 (C2), 27.2 (C11).

HRMS (ESI^{+}) *m/z* calcd for $C_{17}H_{24}NO$ $[M+H]^{+}$ 258.1852, found 258.1846.

MP: 49-50 °C.

IR: ν_{max} = 2967 (C-H), 1630 (C=O), 1603 (C=C), 1395, 1359, 1192, 703 cm^{-1}.

2-benzyl-*N*-(*tert*-butyl)hexa-3,5-dienamide (*E*/*Z*-9a)

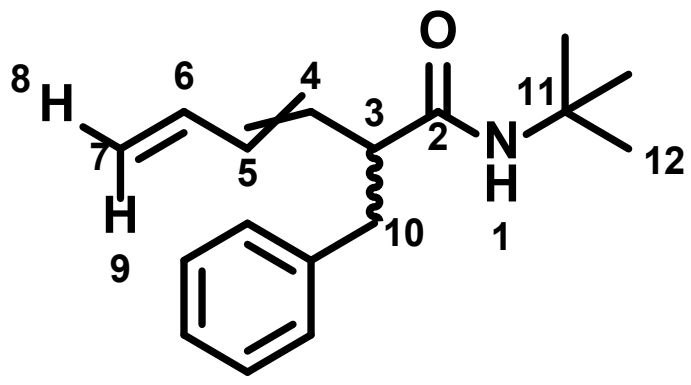

Synthesised according to General Procedure 2 on a 0.39 mmol scale using (*E*)-*N*-benzyl-*N*-(*tert*-butyl)hexa-3,5-dienamide **(8a)** (purification with an eluent of 5-10% EtOAc / petroleum ether) to give a mixture of *E* and *Z* isomers of the title compound (yellow solid) in a ratio of 1.2:1 respectively with an NMR yield of 37%, isolated yield of 30%. *E* and *Z* in assignments below relate to the stereoisomer of the molecule, and not the individual atom discussed.

^{1}**H NMR** (400 MHz, Chloroform-*d*): δ 7.29-7.24 (4H, m, Ar), 7.22-7.17 (6H, m, Ar), 6.46 (1H, dt, *J* = 16.9, 10.0 Hz, *Z*-H6), 6.32 (1H, dt, *J* = 16.9, 10.2 Hz, *E*-H6), 6.15-6.05 (2H, m, *E* & *Z*-H5), 5.78 (1H, dd, *J* = 15.2, 8.8 Hz, *E*-H4), 5.55 (1H, t, *J* = 10.3 Hz, *Z*-H4), 5.27-5.05 (6H, m, *E* & *Z*-H1, H8 & H9), 3.42 (1H, dt, *J* = 9.8, 7.0 Hz, *Z*-H3), 3.15 (2H, 2 x overlapping dd, *J* = 13.6, 7.2 Hz & 10.8, 7.1 Hz, *E* & *Z*-H10), 2.94 (1H, td, *J* = 10.2, 7.1 Hz, *E*-H3), 2.80 (2H, 2 x overlapping dd, *J* = 13.3, 7.3 & 10.5, 7.3 Hz, *E* & *Z*-H10), 1.25 (9H, s, *Z*-H16), 1.23 (9H, s, *E*-H16).

^{13}**C NMR** (101 MHz, Chloroform-*d*): δ 171.8 (*E*-C2), 171.6 (*Z*-C2), 139.5 (Ar), 139.4 (Ar), 136.6 (*E*-C6), 133.2 (*Z*-C5), 132.8 (*E*-C4) , 131.7 (*E*-C5), 131.4 (*Z*-C6), 130.2 (*Z*-C4), 129.4 (Ar), 128.4 (Ar), 126.4 (Ar), 119.6 (*E*/*Z*-C7), 117.1 (*E*/*Z*-C7), 53.4 (*E*-C3), 50.8 (*E* & *Z*-C11), 48.7 (*Z*-C3), 39.1 (E/Z-C10), 38.5 (*E*/*Z*-C10), 28.8 (*E*/*Z*-C12), 28.7 (*E*/*Z*-C12).

HRMS (ESI^{+}) *m/z* calcd for $C_{17}H_{24}NO$ $[M+H]^{+}$ 258.1858, found 258.1853.

MP: 94-95 °C.

IR: ν_{max} = 3291 (N-H), 2966 (C-H), 2924 (C-H), 1648 (C=O), 1635 (C=C), 1480, 1324, 1202.

(*E*)-4-benzyl-*N*-(*tert*-butyl)hexa-2,5-dienamide (10a)

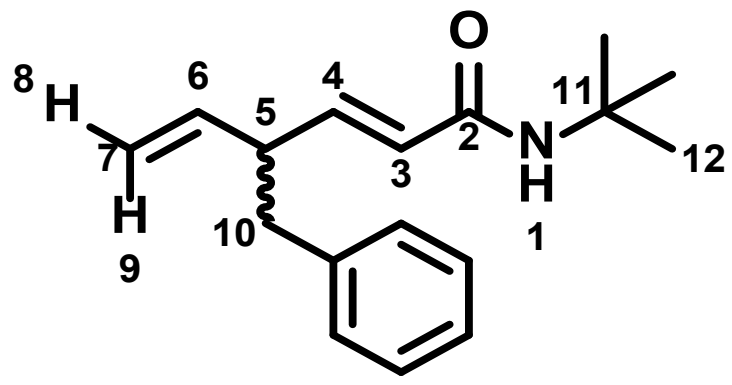

Synthesised according to General Procedure 2 on a 0.39 mmol scale using (*E*)-*N*-benzyl-*N*-(*tert*-butyl)hexa-3,5-dienamide **(8a)** (purification with an eluent of 5-10% EtOAc / petroleum ether) to give a colourless oil with an NMR yield of 10%, isolated yield of 8%.

^{1}H NMR (400 MHz, Chloroform-*d*): δ 7.29-7.24 (2H, m, Ar), 7.20-7.12 (3H, m, Ar), 6.77 (1H, dd, *J* = 15.2, 7.2 Hz, H4), 5.73 (1H, ddd, *J* = 17.2, 9.8, 7.6 Hz, H6), 5.61 (1H, dd, *J* = 15.2, 1.3 Hz, H3), 5.24 (1H, br s, H1), 5.03 (1H, dd, *J* = 10.3, 2.1 Hz, H8), 4.97 (1H, dd, *J* = 17.2, 2.1 Hz, H9), 3.16-3.08 (1H, m, H5), 2.81 (1H, dd, *J* = 13.5, 7.0 Hz, H10), 2.75 (1H, dd, *J* = 13.6, 7.7 Hz, H10), 1.36 (9H, s, H12).

^{13}C NMR (101 MHz, Chloroform-*d*): δ 166.4 (C2), 143.5 (C4), 139.8 (Ar), 138.9 (C6), 129.4 (Ar), 128.4 (Ar), 125.7 (Ar), 124.3 (C3), 116.2 (C7), 50.6 (C11), 48.7 (C5), 41.7 (C10), 28.5 (C12).

HRMS (ESI$^+$) *m/z* calcd for $C_{17}H_{24}NO$ $[M+H]^+$ 258.1858, found 258.1860.

IR: v_{max} = 3302 (N-H), 2968 (C-H), 1663 (C=O), 1627 (C=C), 1538, 1453, 1365, 1221.

(2*E*,4*E*)-*N*-(*tert*-butyl)-7-phenylhepta-2,4-dienamide (11a)

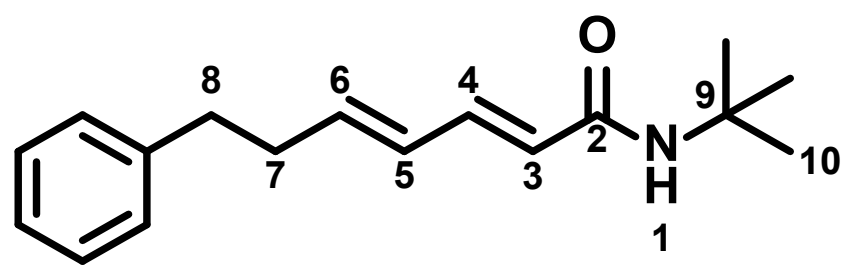

Synthesised according to General Procedure 2 on a 0.39 mmol scale using (*E*)-*N*-benzyl-*N*-(*tert*-butyl)hexa-3,5-dienamide **(8a)** (purification with an eluent of 5-10% EtOAc / petroleum ether) to give a white crystalline solid, with an NMR yield of 28%, isolated yield of 16%.

^{1}H NMR (400 MHz, Chloroform-*d*): δ 7.28-7.24 (2H, m, Ar), 7.19-7.13 (3H, m, Ar), 7.10 (1H, dd, *J* = 15.1, 9.9 Hz, H4), 6.14-6.00 (2H, m, H5, H6), 5.66 (1H, d, *J* = 15.0 Hz, H3), 5.31 (1H, br s, H1), 2.71 (2H, t, *J* = 7.4 Hz, H8), 2.44 (2H, dt, *J* = 9.3, 7.2 Hz, H7), 1.36 (9H, s, H10).

^{13}C NMR (101 MHz, Chloroform-*d*): δ 165.8 (C1), 141.4 (C5/C6), 140.5 (C4), 139.6 (Ar), 128.9 (C5/C6), 128.6 (Ar), 128.5 (Ar), 126.1 (Ar), 123.5 (C3), 51.9 (C9), 36.7 (C8), 34.8 (C7), 30.5 (C10).

HRMS (ESI$^+$) *m/z* calcd for $C_{17}H_{24}NO$ $[M+H]^+$ 258.1858, found 258.1860.

MP: 127-128 °C.

IR: v_{max} = 3262 (N-H), 2968 (C=O), 1654 (C=O), 1628 (C=C), 1611 (C=C), 1553, 1364, 1228, 1033.

(*E*)-*N*-benzyl-*N*-methylhexa-3,5-dienamide (8b)

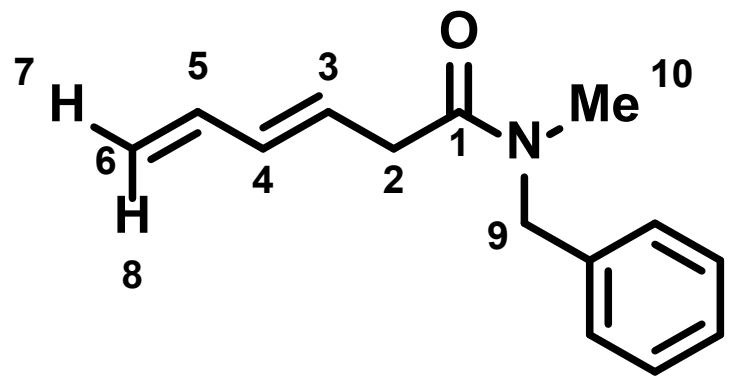

Synthesised according to General Procedure 1 on an 8.92 mmol scale to give a colourless liquid (7.40 mmol, 83%), using (*E*)-hexa-3,5-dienoic acid and *N*-methyl-1-phenylmethanamine as reagents. Purification was carried out with an eluent of 10% EtOAc / petroleum ether.

The NMR indicates a rotameric structure.

^{1}H NMR (400 MHz, Chloroform-*d*): δ 7.41-7.14 (5H, m, Ar), 6.43-6.29 (1H, m, H5), 6.20-6.05 (1H, m, H4), 5.94-5.84 (1H, m, H3), 5.14 (1H, 2 × overlapping d, *J* = 17.0, H8), 5.05 (1H, 2 × overlapping d, *J* = 10.5 Hz, H7), 4.60 (1.20H, s, H9), 4.55 (0.80H, s, H9), 3.24 (2H, t, *J* = 6.9 Hz, H2), 2.96 (1.20H, s, H10), 2.93 (1.80H, s, H10).

^{13}C NMR (101 MHz, Chloroform-*d*): δ 171.4 (C1), 171.0 (C1), 137.4 (Ar), 136.7 (C5), 136.6 (C5), 136.6 (Ar), 133.9 (C4), 133.8 (C4), 129.0 (Ar), 128.7 (Ar), 128.2 (Ar), 127.7 (Ar), 127.5 (Ar), 127.3 (C3), 127.0 (C3), 126.4 (Ar), 116.6 (C6), 116.5 (C6), 53.6 (C9), 51.0 (C9), 37.7 (C2), 37.4 (C2), 35.0 (C10), 34.1 (C10).

HRMS (ESI^{+}) *m/z* calcd for $C_{14}H_{18}NO$ [M+H]$^{+}$ 216.1383, found 216.1379.

IR: v_{max} = 2922 (C-H), 1634 (C=O and C=C broad), 1451, 1398, 1003.

2-Benzyl-*N*-methylhexa-3,5-dienamide (9b)

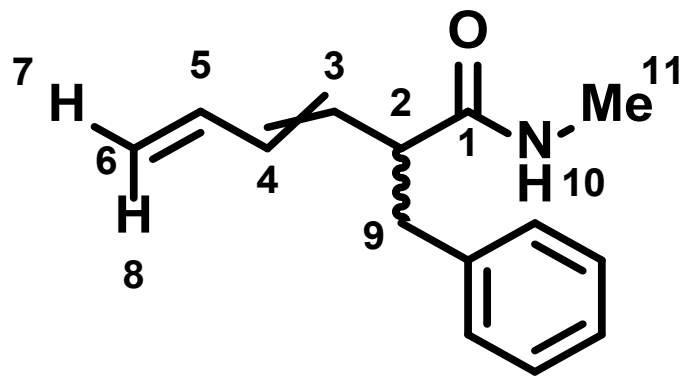

Synthesised according to General Procedure 2 on a 0.46 mmol scale using (*E*)-*N*-benzyl-*N*-methylhexa-3,5-dienamide **(8b)** (purification with an eluent of 0-5% EtOAc / petroleum ether) to give a mixture of *E* and *Z* isomers (in a ratio of 1:0.42 respectively) of the title compound as a colourless oil with an isolated yield of 13%. *E* and *Z* in the assignments refer to the stereochemistry of the molecule and not the individual atom. Starting material was also isolated from this reaction (15%). Not all signals seen in the ^{13}C NMR, likely due to overlap.

^{1}H NMR (400 MHz, Chloroform-*d*): δ 7.30-7.11 (10H, m, Ar), 6.42 (1H, dt, *J* = 16.9, 10.0 Hz, *Z*-H5), 6.29 (1H, dt, *J* = 16.9, 10.0 Hz, *E*-H5), 6.16-6.03 (2H, *E* & *Z*-H4), 5.74 (1H, dd, *J* = 15.5, 9.2 Hz, *E*-H3), 5.51 (1H, t, *J* = 9.5 Hz, *Z*-H3), 5.40 (2H, br s, *E* & *Z*-H10), 5.24 (1H, dd, *J* = 16.9, 1.1 Hz, *Z*-H8), 5.18-5.10 (2H, m, *E*-H8, *Z*-H7), 5.06 (1H, dd, *J* = 10.0, 1.1 Hz, *E*-H7), 3.52 (1H, dt, *J* = 9.5, 7.2 Hz, *Z*-H2), 3.24 (1H, dd, *J* = 13.7, 6.7 Hz, *Z*-H9), 3.19 (1H, dd, *J* = 13.0, 6.7 Hz, *E*-H9), 3.05 (1H, q, *J* = 7.0 Hz, *E*-H2), 2.87-2.78 (2H, m, *Z*-H9, *E*-H9), 2.73 (3H, d, *J* = 4.9 Hz, *Z*-H11), 2.72 (3H, d, *J* = 4.9 Hz, *E*-H11).

^{13}C NMR (101 MHz, Chloroform-*d*): δ 173.2 (*E*-C1), 173.1 (*Z*-C1), 139.4 (Ar), 139.3 (Ar), 136.4 (*E*-C5), 133.9 (*E*-C4), 132.3 (*Z*-C4), 132.1 (*E*-C3), 131.2 (*Z*-C3), 129.6 (Ar), 129.3 (Ar), 128.5 (Ar), 126.4 (Ar), 120.0 (*Z*-C6), 117.5 (*E*-C6), 53.0 (*E*-C2), 47.9 (*Z*-C2), 38.8 (*E*/*Z*-C9), 26.6 (*Z*-C11), 26.5 (*E*-C11).

HRMS (ESI^{+}) *m/z* calcd for $C_{14}H_{18}NO$ $[M+H]^{+}$ 216.1383, found 216.1378.

IR: ν_{max} = 3380 (N-H), 2922 (C-H), 1641 (C=O and C=C), 1451, 1400, 1117.

(*E*)-4-Benzyl-*N*-methylhexa-2,5-dienamide (10b)

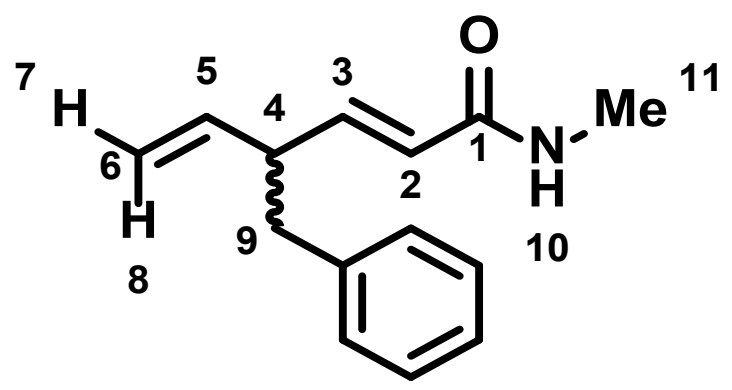

Synthesised according to General Procedure 2 on a 0.46 mmol scale using (*E*)-*N*-benzyl-*N*-methylhexa-3,5-dienamide **(8b)** (purification with an eluent of 0-5% EtOAc / petroleum ether) to give the title compound as a colourless oil with an isolated yield of 10%. Starting material was also isolated from this reaction (15%).

^{1}H NMR (400 MHz, Chloroform-*d*): δ 7.30-7.10 (5H, m, Ar), 6.80 (1H, dd, *J* = 15.4, 7.4 Hz, H3), 5.75 (1H, ddd, *J* = 17.6, 10.5, 7.2 Hz, H5), 5.66 (1H, dd, *J* = 15.1, 1.0 Hz, H2), 5.47 (1H, br s, H10), 5.04 (1H, dd, *J* = 10.5, 1.0 Hz, H7), 4.99 (1H, dd, *J* = 17.6, 1.0 Hz, H8), 3.14 (1H, pent, *J* = 7.4 Hz, H4), 2.84 (3H, d, *J* = 5.0 Hz, H11), 2.79 (2H, d, *J* = 7.4 Hz, H9).

^{13}C NMR (101 MHz, Chloroform-*d*): δ 166.5 (C1), 145.4 (C3), 139.2 (Ar), 138.9 (C5), 129.4 (Ar), 128.4 (Ar), 126.3 (Ar), 123.8 (C2), 116.2 (C6), 47.9 (C4), 41.0 (C9), 26.4 (C11).

HRMS (ESI$^+$) *m/z* calcd for $C_{14}H_{18}NO$ $[M+H]^+$ 216.1383, found 216.1378.

IR: v_{max} = 3291 (N-H), 3084 (C-H), 2923 (C-H), 1664 (C=O), 1623 (C=C), 1550 (C=C), 1272, 1158, 698.

(2*E*,4*E*)-*N*-methyl-7-phenylhepta-2,4-dienamide (11b)

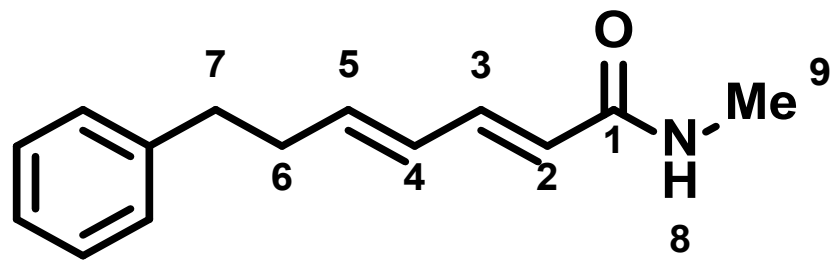

Synthesised according to General Procedure 2 on a 0.46 mmol scale using (*E*)-*N*-benzyl-*N*-methylhexa-3,5-dienamide **(8b)** (purification with an eluent of 0-5% EtOAc / petroleum ether) to give the title compound as a white solid with an isolated yield of 13%. Starting material was also isolated from this reaction (15%).

^{1}H NMR (400 MHz, Chloroform-*d*): δ 7.37-7.19 (6H, m, H3, Ar), 6.23-6.09 (2H, m, H4, H5), 5.81 (1H, d, *J* = 14.9 Hz, H2), 5.72 (1H, br s, H8), 2.93 (3H, d, *J* = 4.9 Hz, H9), 2.79 (2H, t, *J* = 6.9 Hz, H7), 2.52 (1H, q, *J* = 6.9 Hz, H6).

^{13}C NMR (101 MHz, Chloroform-*d*): δ 167.1 (C1), 141.8 (C4/C5), 141.4 (Ar), 141.0 (C3), 129.0 (C4/C5), 128.5 (Ar), 128.4 (Ar), 126.1 (Ar), 122.1 (C2), 35.3 (C7), 34.8 (C6), 26.5 (C9).

HRMS (ESI^{+}) *m/z* calcd for $C_{14}H_{18}NO$ [M+H]$^{+}$ 216.1383, found 216.1378.

MP: 82-83 °C.

IR: ν_{max} = 3302 (N-H), 2955 (C-H), 2923 (C-H), 2853 (C-H), 1654 (C=O), 1628 (C=C), 1454, 1262, 996, 695.

(*E*)-*N*-allyl-*N*-benzylhexa-3,5-dienamide (8c)

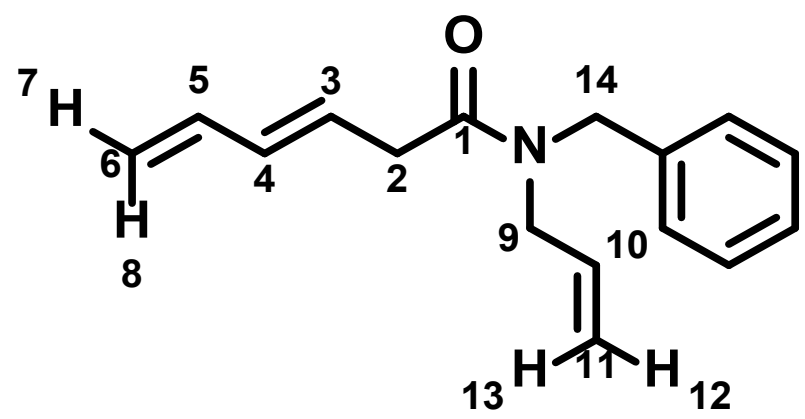

(*E*)-hexa-3,5-dienoic acid (4.46 mmol, 1.1 eq.) was dissolved in anhydrous CH_2Cl_2 (7 mL) with a few drops of DMF. Oxalyl chloride (5.28 mmol, 1.3 eq.) was added slowly to give a dark-purple solution before being left to stir for 2 hours. Excess volatiles were removed *in vacuo* before addition of anhydrous CH_2Cl_2 (14 mL) and (*E*)-*N*-allyl-*N*-benzylhexa-3,5-dienamide (4.05 mmol, 1.0 eq.). The mixture was left to stir for 16 hours before addition of water (10 mL) and the layers separated. The aqueous layer was washed with CH_2Cl_2 (3 × 20 mL) before the combined organics were washed with $NaHCO_{3(aq.)}$ (2 × 20 mL). The organics were dried with $MgSO_4$, filtered and concentrated *in vacuo*. The residue was purified by flash column chromatography (0-1% MeOH / CH_2Cl_2) to afford the title compound as a yellow oil (0.61 mmol, 15%).

The NMR indicates a rotameric structure.

^{1}H NMR (400 MHz, Chloroform-*d*): δ 7.36-7.10 (5H, m, Ar), 6.30 (1H, ddd, *J* = 17.0, 10.2, 10.0 Hz, H5), 6.09 (1H, dd, *J* = 15.7, 10.0 Hz, H4), 5.90-5.78 (1H, m, H3), 5.77-5.64 (1H, m, H10), 5.19 (1H, dd, *J* = 10.4, 1.4 Hz, H12), 5.14-4.96 (3H, m, H7, H8, H13), 4.55 (1.2H, s, H14), 4.47 (0.8H, s, H14), 3.97 (0.8H, d, *J* = 6.5 Hz, H9), 3.79 (1.2H, d, *J* = 6.5 Hz, H9), 3.17 (2H, t, *J* = 7.1 Hz, H2).

^{13}C NMR (101 MHz, Chloroform-*d*): δ 171.2 (C1), 171.1 (C1), 137.4 (Ar), 136.5 (C5), 136.4 (Ar), 133.7 (C4), 132.8 (C10), 132.5 (C10), 128.9 (Ar), 128.5 (Ar), 128.2 (Ar), 127.5 (Ar), 127.3 (Ar), 127.2 (C3), 127.0 (C3), 126.3 (Ar), 117.6 (C6/C11), 116.9 (C6/C11), 116.5 (C6/C11), 116.4 (C6/C11), 50.1 (C14), 49.1 (C9), 48.2 (C9), 48.0 (C14), 37.3 (C2), 37.1 (C2).

HRMS (ESI$^+$) *m/z* calcd for $C_{16}H_{20}NO$ [M+H]$^+$ 242.1539, found 242.1533.

IR: ν_{max} = 2958 (C-H), 2927 (C-H),M 2860 (C-H), 1732 (C=O), 1622 (C=C), 1451, 1166.

N-allyl-2-benzylhexa-3,5-dienamide (9c)

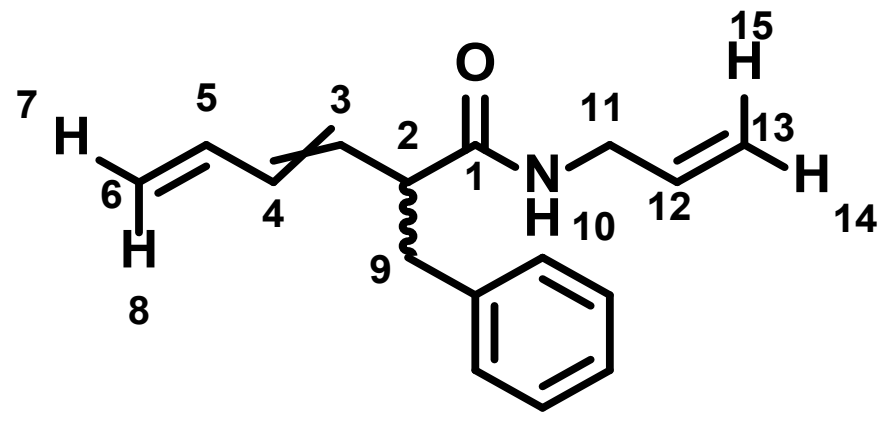

Synthesised according to General Procedure 2 on a 0.42 mmol scale using (*E*)-*N*-allyl-*N*-benzylhexa-3,5-dienamide **(8c)** (purification with an eluent of 0-20% EtOAc / petroleum ether) to give a mixture of *E* and *Z* isomers (in a ratio of 1:0.49 respectively) of the title compound as a colourless oil with an isolated yield of 14%. *E* and *Z* in the assignments refer to the stereochemistry of the molecule and not the individual atom. Not all aromatic signals seen in the ^{13}C NMR, likely due to overlap.

^{1}H NMR (400 MHz, Chloroform-*d*): δ 7.32-7.09 (10H, m, Ar), 6.45 (1H, dtd, *J* = 16.7, 10.1, 1.0 Hz, *Z*-H5), 6.30 (1H, dtd, *J* = 17.0, 10.1, 1.0 Hz, *E*-H5), 6.17-6.05 (2H, m, *E* & *Z*-H4), 5.77 (1H, dd, *J* = 15.2, 8.8 Hz, *E*-H3), 5.73-5.64 (2H, m, *E* & *Z*-H12), 5.53 (1H, t, *J* = 10.4 Hz, *Z*-H3), 5.46 (2H, br s, *E* & *Z*-H10), 5.25 (1H, dd, *J* = 16.6, 1.6 Hz, *Z*-H8), 5.19-5.12 (2H, m, *E*-H8, *Z*-H7), 5.09-5.05 (1H, dd, *J* = 9.8, 1.6 Hz, *E*-H7), 5.04-4.95 (4H, m, *E* & *Z*-H14 & H-15), 3.87-3.74 (4H, m, *E* & *Z*-H11), 3.59-3.51 (1H, m, *Z*-H2), 3.27-3.15 (2H, m, *E* & *Z*-H9), 3.08 (1H, q, *J* = 7.2 Hz, *E*-H2), 2.88-2.81 (2H, m, *E* & *Z*-H9).

^{13}C NMR (101 MHz, Chloroform-*d*): δ 172.5 (*E*-C1), 172.3 (*Z*-C1), 139.3 (Ar), 139.2 (Ar), 136.4 (*Z*-C5), 134.1 (*E*-C5), 133.9 (*E*-C4), 132.3 (*Z*-C4), 132.0 (*E*-C12), 131.2 (*Z*-C12), 129.5 (*Z*-C3), 129.3 (*E*-C3), 128.5 (Ar), 126.5 (Ar), 120.0 (*Z*-C6), 117.6 (*E*-C6), 116.4 (*E*-C13), 116.3 (*Z*-C13), 53.2 (*E*-C2), 48.0 (*Z*-C2), 42.0 (*E*-C11), 41.9 (*Z*-C11), 38.8 (*E*-C9), 38.8 (*Z*-C9).

HRMS (ESI^{+}) *m/z* calcd for $C_{16}H_{20}NO$ $[M+H]^{+}$ 242.1539, found 242.1532.

IR: ν_{max} = 3288 (N-H), 2922 (C-H), 2853 (C-H), 1638 (C=O), 1532 (C=C), 1258, 1029.

(*E*)-*N*-allyl-4-benzylhexa-2,5-dienamide (10c)

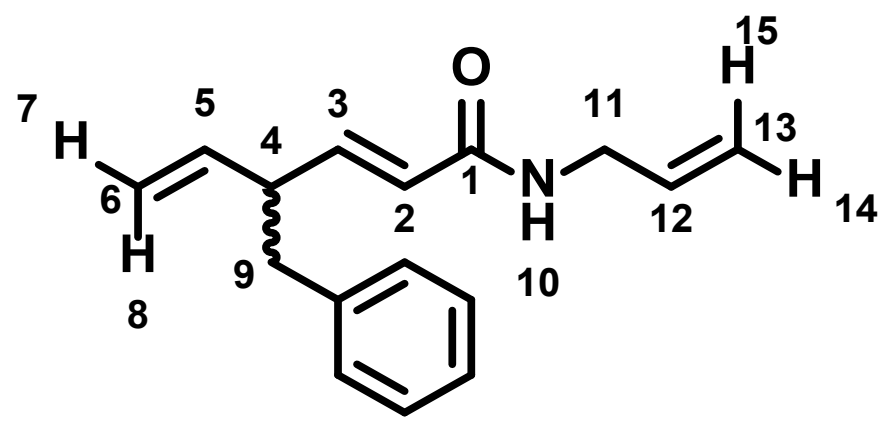

Synthesised according to General Procedure 2 on a 0.42 mmol scale using (*E*)-*N*-allyl-*N*-benzylhexa-3,5-dienamide **(8c)** (purification with an eluent of 0-20% EtOAc / petroleum ether) to give the title compound as an orange oil with an isolated yield of 6%.

^{1}H NMR (400 MHz, Chloroform-*d*): δ 7.36-7.09 (5H, m, Ar), 6.83 (1H, dd, *J* = 15.6, 8.0 Hz, H3), 5.91-5.72 (2H, H5, H12), 5.68 (1H, dd, *J* = 15.5, 1.3 Hz, H2), 5.51 (1H, br s, H10), 5.17 (1H, dd, *J* = 18.0, 1.3 Hz, H8/H15), 5.13 (1H, dd, *J* = 10.1, 1.3 Hz, H7/H14), 5.05 (1H, dd, *J* = 10.3, 1.3 Hz, H7/H14), 4.99 (1H, dd, *J* = 17.9, 1.4 Hz, H8/H15), 3.99-3.89 (2H, m, H11), 3.15 (1H, pent, *J* = 7.1 Hz, H4), 2.79 (2H, d, *J* = 7.1 Hz, H9).

^{13}C NMR (101 MHz, Chloroform-*d*): δ 165.6 (C1), 146.0 (C3), 139.2 (Ar), 138.8 (Ar), 134.2 (C12), 129.4 (C5), 128.4 (Ar), 126.3 (Ar), 123.7 (C2), 116.6 (C6/C13), 116.3 (C6/C13), 48.0 (C11), 42.0 (C4), 40.9 (C9).

HRMS (ESI$^+$) *m/z* calcd for $C_{16}H_{20}NO$ [M+H]$^+$ 242.1539, found 242.1532.

IR: ν_{max} = 3286 (N-H), 2922 (C-H), 1710 (C=O), 1665 (C=C), 1627 (C=C), 1537 (C=C), 1258, 985, 698.

(2*E*,4*E*)-*N*-allyl-7-phenylhepta-2,4-dienamide (11c)

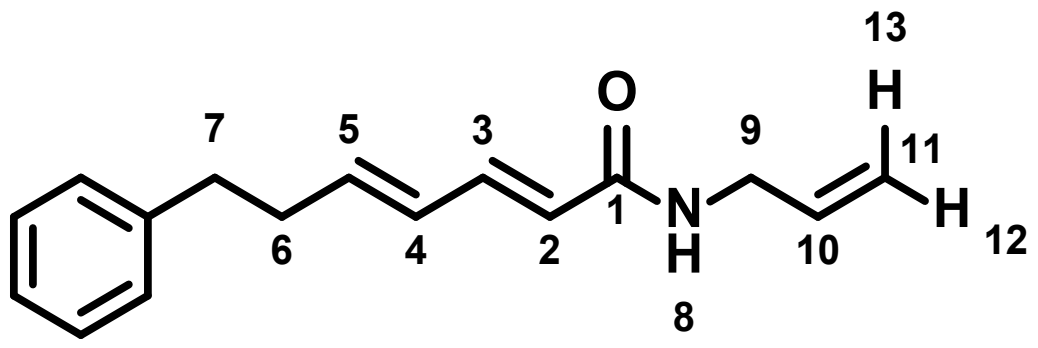

Synthesised according to General Procedure 2 on a 0.42 mmol scale using (*E*)-*N*-allyl-*N*-benzylhexa-3,5-dienamide **(8c)** (purification with an eluent of 0-20% EtOAc / petroleum ether) to give the title compound as a white crystalline solid with an isolated yield of 14%.

^{1}H NMR (400 MHz, Chloroform-*d*): δ 7.32-7.12 (6H, m, Ar, H3), 6.19-6.04 (2H, m, H4, H5), 5.86 (1H, ddt, *J* = 16.9, 10.1, 5.9 Hz, H10), 5.76 (1H, d, *J* = 15.3 Hz, H2), 5.62 (1H, br s, H8), 5.19 (1H, dd, *J* = 16.9, 1.3 Hz, H13), 5.13 (1H, dd, *J* = 10.1, 1.3 Hz, H12), 3.96 (2H, tt, *J* = 5.9, 1.5 Hz, H9), 2.74 (2H, t, *J* = 7.6 Hz, H7), 2.47 (1H, q, *J* = 7.6 Hz, H6).

^{13}C NMR (101 MHz, Chloroform-*d*): δ 166.3 (C1), 142.5 (C3), 141.5 (C5), 141.3 (Ar), 134.3 (C10), 128.9 (C4), 128.5 (Ar), 128.4 (Ar), 126.1 (Ar), 122.0 (C2), 116.6 (C11), 42.1 (C9), 35.3 (C7), 34.9 (C6).

HRMS (ESI$^+$) *m/z* calcd for $C_{16}H_{20}NO$ $[M+H]^+$ 242.1539, found 242.1533.

MP: 91-92 °C.

IR: ν_{max} = 3298 (N-H), 2919 (C-H), 1652 (C=O), 1625 (C=C), 1610 (C=C), 1538 (C=C), 1260, 989, 697.

(*E*)-*N*-benzyl-*N*-cyclopropylhexa-3,5-dienamide (8d)

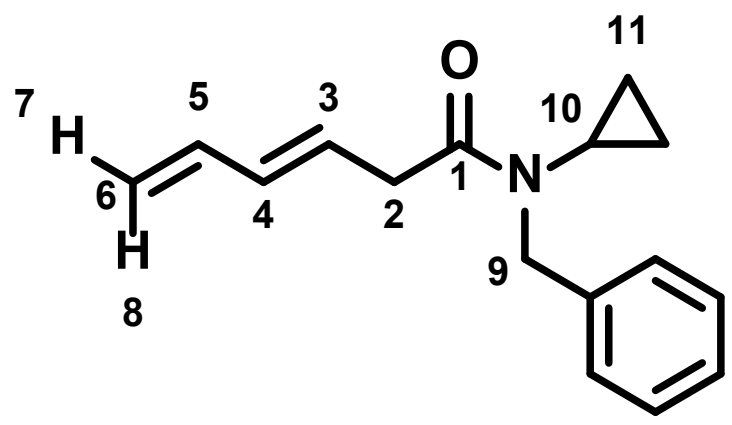

Synthesised according to General Procedure 1 on an 8.92 mmol scale to give a colourless oil (4.55 mmol, 51%), using (*E*)-hexa-3,5-dienoic acid and *N*-benzylcyclopropanamine as reagents. Purification was carried out with an eluent of 5% EtOAc / petroleum ether.

^{1}H NMR (400 MHz, Chloroform-*d*): δ 7.34-7.19 (5H, m, Ar), 6.36 (1H, dt, *J* = 17.1, 10.2 Hz, H5), 6.14 (1H, dd, *J* = 15.4, 10.2 Hz, H4), 5.97-5.87 (1H, m, H3), 5.12 (1H, d, *J* = 17.1 Hz, H8), 5.02 (1H, d, *J* = 10.2 Hz, H7), 4.60 (2H, s, H9), 3.43 (2H, d, *J* = 6.9 Hz, H2), 2.60-2.52 (1H, m, H10), 0.91-0.72 (4H, m, H11).

^{13}C NMR (101 MHz, Chloroform-*d*): δ 173.9 (C1), 138.3 (Ar), 136.8 (C5), 133.7 (C4), 128.5 (Ar), 127.9 (Ar), 127.7 (C3), 127.1 (Ar), 116.3 (C6), 49.9 (C9), 38.2 (C2), 30.1 (C10), 9.3 (C11).

HRMS (ESI$^+$) *m/z* calcd for $C_{16}H_{20}NO$ $[M+H]^+$ 242.1539, found 242.1536.

IR: ν_{max} = 3302 (N-H), 2925 (C-H), 1651 (C=O and C=C broad), 1397, 1253, 1002, 698.

2-Benzyl-*N*-cyclopropylhexa-3,5-dienamide (9d)

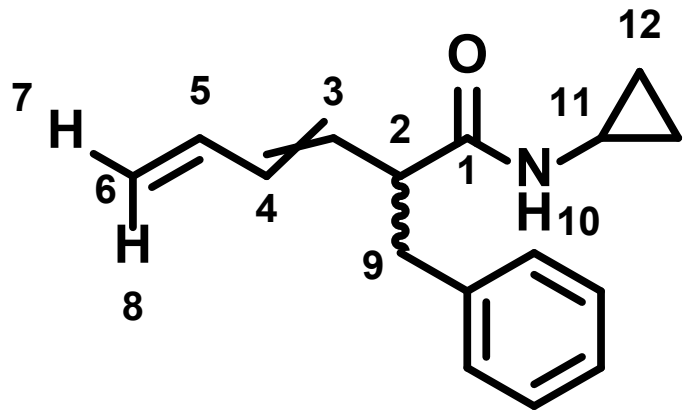

Synthesised according to General Procedure 2 on a 0.41 mmol scale using (*E*)-*N*-benzyl-*N*-cyclopropylhexa-3,5-dienamide **(8d)** (purification with an eluent of 5-30% EtOAc / petroleum ether) to give a mixture of *E* and *Z* isomers (in a ratio of 1:0.24 respectively) of the title compound as a colourless oil with an isolated yield of 27%. *E* and *Z* in the assignments refer to the stereochemistry of the molecule and not the individual atom. Not all signals seen in the ^{13}C NMR, likely due to overlap.

^{1}H NMR (400 MHz, Chloroform-*d*): δ 7.34-7.09 (5H, m, Ar), 6.43 (1H, dt, *J* = 16.8, 10.8 Hz, *Z*-H5), 6.30 (1H, dt, *J* = 17.2, 10.0 Hz, *E*-H5), 6.16-6.02 (2H, 2 × overlapping dd, *J* = 15.0, 9.8 Hz, *E* & *Z*-H4), 5.76 (1H, dd, *J* = 15.0, 9.0 Hz, *E*-H3), 5.64-5.48 (3H, br m, *Z*-H3, *E* & *Z*-H10), 5.24 (1H, dd, *J* = 16.8, 1.0 Hz, *Z*-H8), 5.19-5.10 (2H, m, *Z*-H7, *E*-H8), 5.07 (1H, d, *J* = 10.0 Hz, *E*-H7), 3.48 (1H, dt, *J* = 9.6, 6.9 Hz, *Z*-H2), 3.23-3.11 (2H, 2 × overlapping dd, *J* = 13.7, 8.5 Hz, *E* & *Z*-H9), 3.01 (1H, q, *J* = 7.4 Hz, *E*-H2), 2.87-2.74 (2H, 2 × overlapping dd, *J* = 13.7, 6.9 Hz, *E* & *Z*-H9), 2.65-2.57 (2H, m, *E* & *Z*-H11), 0.73-0.64 (4H, m, *E* & *Z*-H12), 0.37-0.30 (4H, m, *E* & *Z*-H12).

^{13}C NMR (101 MHz, Chloroform-*d*): δ 174.0 (*E*-C1), 173.9 (*Z*-C1), 139.3 (Ar), 139.2 (Ar), 136.5 (*E*-C5), 133.6 (*E*-C4), 132.0 (*E*-C3), 132.0 (*Z*-C4), 131.2 (*Z*-C5), 129.5 (*Z*-C3), 129.3 (Ar), 128.4 (Ar), 126.4 (Ar), 119.8 (*Z*-C6), 117.2 (*E*-C6), 52.9 (*E*-C2), 47.8 (*Z*-C2), 38.9 (*E*/*Z*-C9), 38.9 (*E*/*Z*-C9), 22.7 (*Z*-C11), 22.7 (*E*-C11), 6.7 (*E*-C12), 6.7 (*Z*-C12), 6.6 (*Z*-C12), 6.6 (*E*-C12).

HRMS (ESI^{+}) *m/z* calcd for $C_{16}H_{20}NO$ [M+H]$^{+}$ 242.1539, found 242.1535.

IR: ν_{max} = 3279 (N-H), 2980 (C-H), 2922 (C-H), 1646 (C=O and C=C), 1453, 1399.

(*E*)-4-benzyl-*N*-cyclopropylhexa-2,5-dienamide (10d)

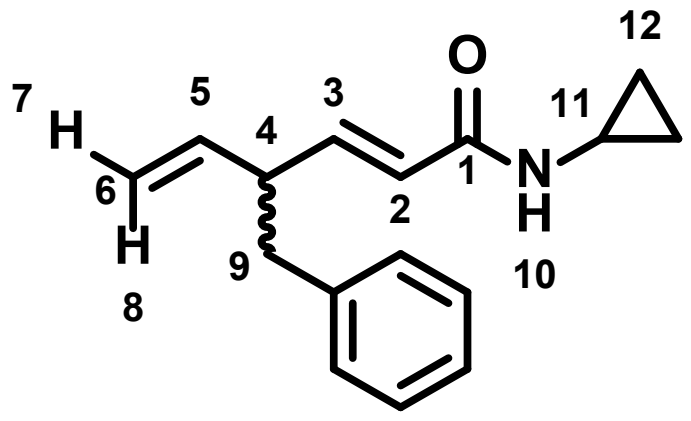

Synthesised according to General Procedure 2 on a 0.41 mmol scale using (*E*)-*N*-benzyl-*N*-cyclopropylhexa-3,5-dienamide **(8d)** (purification with an eluent of 5-30% EtOAc / petroleum ether) to give the title compound as a colourless oil with an isolated yield of 16%.

^{1}H NMR (400 MHz, Chloroform-*d*): δ 7.30-7.08 (5H, m, Ar), 6.82 (1H, dd, *J* = 15.4, 7.3 Hz, H3), 5.73 (1H, ddd, *J* = 17.3, 10.1, 7.5 Hz, H5), 5.65-5.56 (2H, m, H2, H10), 5.04 (1H, d, *J* = 10.1 Hz, H7), 4.97 (1H, d, *J* = 17.3 Hz, H8), 3.18-3.07 (1H, m, H4), 2.82-2.75 (3H, m, H9, H11), 0.81-0.71 (2H, m, H12), 0.55-0.46 (2H, m, H12).

^{13}C NMR (101 MHz, Chloroform-*d*): δ 167.1 (C1), 145.7 (C3), 139.2 (Ar), 138.8 (C5), 129.4 (Ar), 128.4 (Ar), 126.3 (Ar), 123.7 (C2), 116.2 (C6), 48.0 (C4), 40.9 (C9), 22.8 (C11), 6.8 (C12), 6.7 (C12).

HRMS (ESI^+) *m/z* calcd for $C_{16}H_{20}NO$ $[M+H]^+$ 242.1539, found 242.1537.

IR: ν_{max} = 3251 (N-H), 2955 (C-H), 2921 (C-H), 2852 (C-H), 1701 (C=O), 1634 (C=C), 1454, 975.

(2*E*,4*E*)-*N*-cyclopropyl-7-phenylhepta-2,4-dienamide (11d)

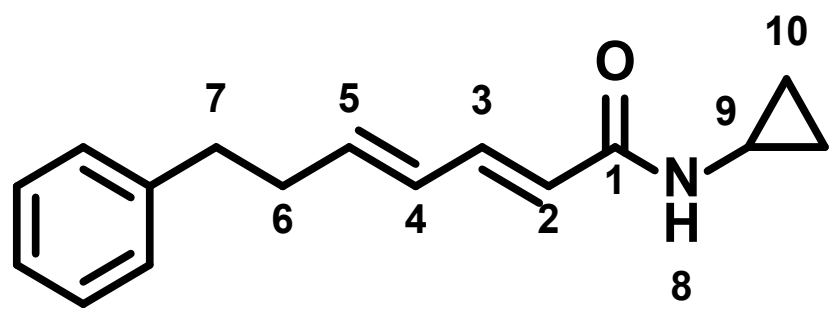

Synthesised according to General Procedure 2 on a 0.41 mmol scale using (*E*)-*N*-benzyl-*N*-cyclopropylhexa-3,5-dienamide **(8d)** (purification with an eluent of 5-30% EtOAc / petroleum ether) to give the title compound as a white solid with an isolated yield of 43%.

^{1}H NMR (400 MHz, Chloroform-*d*): δ 7.31-7.13 (6H, m, H3, Ar), 6.16-6.04 (2H, m, H4, H5), 5.68 (1H, d, *J* = 15.0 Hz, H2), 5.62 (1H, br s, H8), 2.86-2.76 (1H, m, H9), 2.73 (2H, t, *J* = 7.4 Hz, H7), 2.52-2.42 (2H, m, H6), 0.82-0.76 (2H, m, H10), 0.55-0.50 (2H, m, H10).

^{13}C NMR (101 MHz, Chloroform-*d*): δ 167.7 (C1), 142.0 (C4/C5), 141.4 (Ar), 141.3 (C3), 128.9 (C4/C5), 128.6 (Ar), 128.5 (Ar), 126.2 (Ar), 122.0 (C2), 35.3 (C7), 34.9 (C6), 22.9 (C9), 6.9 (C10).

HRMS (ESI$^+$) *m/z* calcd for $C_{16}H_{20}NO$ $[M+H]^+$ 242.1539, found 242.1538.

MP: 140-141 °C.

IR: ν_{max} = 3292 (N-H), 2980 (C-H), 2924 (C-H), 1628 (C=O), 1381, 1152.

(*S,E*)-*N*-isopropyl-*N*-(1-phenylethyl)hexa-3,5-dienamide (8e)

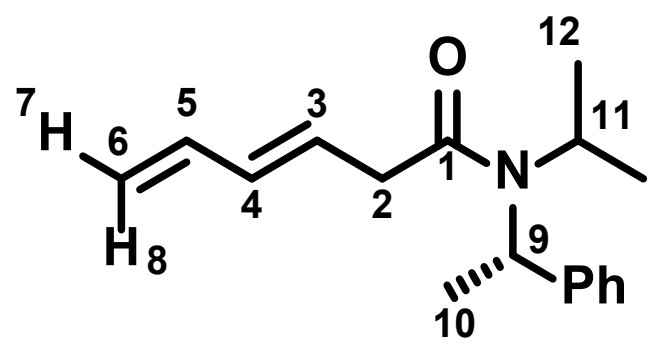

Synthesised according to General Procedure 1 on an 17.83 mmol scale to give a colourless oil (9.45 mmol, 53%), using (*E*)-hexa-3,5-dienoic acid and (*S*)-*N*-(1-phenylethyl)propan-2-amine as reagents. Purification was carried out with an eluent of 5-10% EtOAc / petroleum ether. Broad NMR peaks due to rotameric structure.

^{1}H NMR (400 MHz, Chloroform-*d*): δ 7.37-7.18 (5H, m, Ar), 6.33 (1H, dt, *J* = 17.1, 10.1 Hz, H5), 6.16-5.98 (1H, m, H4), 5.88 (1H, dt, *J* = 15.3, 6.7 Hz, H3), 5.10 (1H, d, *J* = 17.1 Hz, H8), 5.00 (1H, d, *J* = 10.6 Hz, H7), 4.99-4.93 (1H, m, H9), 3.86 (0.33H, br s, rotameric H11), 3.49 (0.66H, br s, rotameric H11), 3.19 (2H, d, *J* = 6.5 Hz, H2), 1.62 (3H, d, *J* = 6.7 Hz, H10), 1.33 (3H, d, *J* = 7.3 Hz, H12), 1.05 (3H, d, *J* = 6.1 Hz, H12).

^{13}C NMR (101 MHz, Chloroform-*d*): δ 171.1 (C1), 136.8 (C5), 133.7 (C4), 128.9 (Ar), 127.9 (C3), 127.1 (Ar), 123.2 (Ar), 115.9 (C6), 54.2 (C9), 47.6 (C11), 40.8 (C2), 21.0 (C10), 20.0 (C12), 18.2 (C10).

HRMS (ESI^{+}) *m/z* calcd for $C_{17}H_{24}NO$ [M+H]$^{+}$ 258.1858, found 258.1853.

IR: ν_{max} = 2971 (C-H), 1935 (C-H), 1632 (C=O), 1601 (C=C), 1439, 1329, 1116, 1004.

$[\alpha]_D^{24}$**:** −68 (*c* 1, $CHCl_3$).

N-(*tert*-butyl)-2-(1-phenylethyl)hexa-3,5-dienamide (*E*/*Z*-9e)

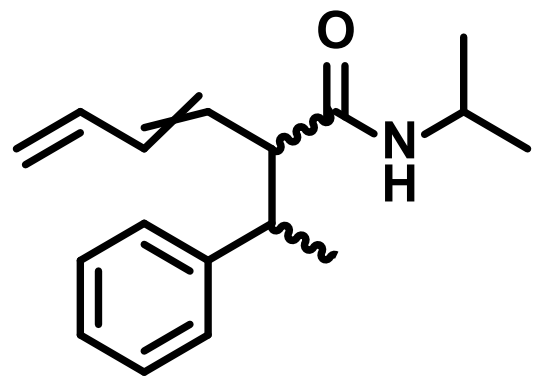

Synthesised according to General Procedure 2 on a 0.39 mmol scale using (*S,E*)-*N*-isopropyl-*N*-(1-phenylethyl)hexa-3,5-dienamide **(8e)** (purification with an eluent of 5-20% EtOAc / petroleum ether) to give a mixture of *E* and *Z* isomers and diastereomers of the title compound as a colourless oil with an isolated yield of 21%. Assignments and crude NMR yield not possible due to the complex mixture but note 12 doublets seen as expected for Me and ^{i}Pr signals for a mixture of four stereoisomers present. Not all carbon signals observed due to overlap. Diastereomeric ratio cannot be determined due to tricky separation of eight compounds present by chiral HPLC.

^{1}H NMR (400 MHz, Chloroform-*d*): δ 7.29-7.13 (m), 6.58 (dt, *J* = 10.2, 1.1 Hz), 6.53 (dt, *J* = 10.2, 1.0 Hz), 6.39 (dt, *J* = 10.4, 0.7 Hz), 6.34 (dt, *J* = 10.2, 0.5 Hz), 6.25-6.15 (m), 6.12 (ddd, *J* = 15.3, 10.5, 0.7 Hz), 6.03 (td, *J* = 11.1, 0.8 Hz), 5.94-5.82 (m), 5.65 (t, *J* = 10.5 Hz), 5.54 (ddd, *J* = 15.3, 9.1, 0.5 Hz), 5.30-4.81 (m), 4.11-4.02 (m), 3.84-3.72 (m), 3.41-3.12 (m), 2.88 (td, *J* = 9.7, 0.7 Hz), 2.72 (t, *J* = 9.7 Hz), 1.32 (d, *J* = 7.0 Hz), 1.30 (d, *J* = 7.1 Hz), 1.24 (d, *J* = 6.8 Hz), 1.23 (d, *J* = 7.2 Hz), 1.11 (d, *J* = 6.7 Hz), 1.09 (d, *J* = 6.6 Hz), 1.08 (d, *J* = 6.6 Hz), 1.04 (d, *J* = 6.5 Hz), 0.95 (d, *J* = 6.5 Hz), 0.94 (d, *J* = 6.6 Hz), 0.67 (d, *J* = 6.6 Hz), 0.62 (d, *J* = 6.6 Hz).

^{13}C NMR (101 MHz, Chloroform-*d*): δ 171.8, 171.6, 171.5, 171.3, 145.0, 144.9, 143.8, 143.3, 136.7, 136.6, 134.1, 133.9, 132.4, 132.3, 132.0, 131.8, 131.5, 131.0, 129.3, 128.5, 128.4, 128.3, 128.3, 128.1, 128.1, 127.6, 127.6, 127.6, 126.5, 126.5, 119.5, 119.3, 116.9, 60.3, 58.2, 54.4, 53.0, 42.4, 42.3, 42.3, 42.1, 41.5, 41.1, 41.0, 22.9, 22.8, 22.6, 22.6, 22.4, 19.6, 19.5, 19.2, 18.6.

HRMS (ESI$^+$) *m/z* calcd for $C_{17}H_{24}NO$ [M+H]$^+$ 258.1858, found 258.1853.

IR: ν_{max} = 3290 (N-H), 2969 (C-H), 1634 (C=O), 1544, 1453, 1366, 1003.

N-(*tert*-butyl)-4-(1-phenylethyl)hexa-2,5-dienamide (10e)

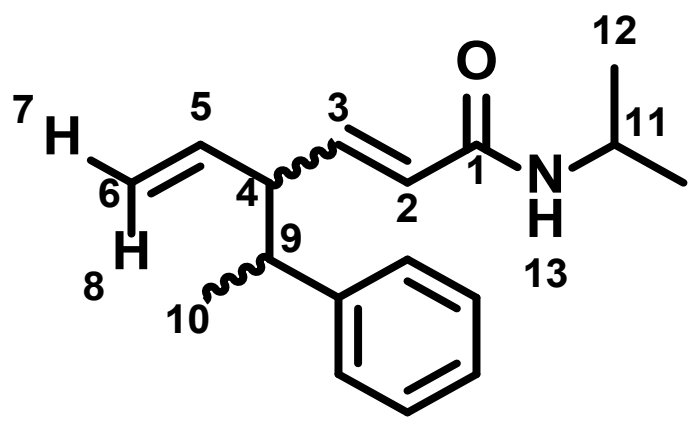

Synthesised according to General Procedure 2 on a 0.39 mmol scale using (*S,E*)-*N*-isopropyl-*N*-(1-phenylethyl)hexa-3,5-dienamide **(8e)** (purification with an eluent of 5-20% EtOAc / petroleum ether) to give an equal mixture of two diastereomers of the title compound as a colourless oil with an NMR yield of 13%, isolated yield of 10%. D1 and D2 refer to the diastereomer responsible for individual signals. Diastereomeric/enantiomeric ratio determined, through the use of chiral HPLC Registech® Whelk column with hexane:2-propanol (90:10) as eluent, as 26:19:26:29 with retention time (mins) 10.54:13.47:14.38:15.40.

^{1}H NMR (400 MHz, Chloroform-*d*): δ 7.30-7.24 (4H, m, Ar), 7.20-7.12 (6H, m, Ar), 6.81 (1H, dd, *J* = 15.4, 7.5 Hz, H3 D1), 6.67 (1H, dd, *J* = 15.4, 7.5 Hz, H3 D2), 5.72 (1H, td, *J* = 17.1, 10.1 Hz, H5 D1), 5.67 (1H, dd, *J* = 15.1, 1.2 Hz, H2 D1), 5.61 (1H, td, *J* = 17.0, 10.3 Hz, H5 D2), 5.54 (1H, dd, *J* = 15.3, 1.4 Hz, H2 D2), 5.37-5.27 (1H, br d, *J* = 7.6 Hz, H13 D1/D2), 5.17 (1H, br d, *J* = 7.6 Hz, H13 D1/D2), 5.10 (1H, dd, *J* = 9.9, 1.4 Hz, H7 D1), 5.01 (1H, dd, *J* = 17.2, 1.1 Hz, H8 D1), 4.94 (1H, dd, *J* = 10.1, 1.6 Hz, H7 D2), 4.89 (1H, dd, *J* = 17.5, 1.9 Hz, H8 D2), 4.19-4.03 (2H, m, H11 D1 & D2), 3.04-2.96 (2H, m, H4 D1 & D2), 2.88-2.80 (2H, m, H9 D1 & D2), 1.26 (3H, d, *J* = 7.1 Hz, H10 D1/D2), 1.25 (3H, d, *J* = 7.2 Hz, H10 D1/D2), 1.17 (3H, d, *J* = 6.6 Hz, H12 D1/D2), 1.16 (3H, d, *J* = 6.7 Hz, H12 D1/D2), 1.12 (3H, d, *J* = 6.7 Hz, H12 D1/D2), 1.11 (3H, d, *J* = 6.6 Hz, H12 D1/D2).

^{13}C NMR (101 MHz, Chloroform-*d*): δ 164.9 (C1 D1/D2), 164.8 (C1 D1/D2), 145.1 (C3 D1/D2), 144.6 (C3 D1/D2), 138.2 (C5 D2), 137.9 (C5 D1), 128.5 (Ar), 128.4 (Ar), 128.3 (Ar), 128.0 (Ar), 128.0 (Ar), 127.0 (Ar), 126.4 (Ar), 126.4 (Ar), 125.0 (C2 D1), 124.6 (C2 D2), 117.1 (C1 D1), 116.5 (C1 D2), 53.7 (C4 D1/D2), 52.6 (C4 D1/D2), 44.1 (C9 D1/D2), 44.0 (C9 D1/D2), 41.5 (C11 D1/D2), 41.4 (C11 D1/D2), 23.0 (C12 D1/D2), 22.9 (C12 D1/D2), 19.5 (C10 D1/D2), 19.2 (C10 D1/D2).

HRMS (ESI$^+$) *m/z* calcd for $C_{17}H_{24}NO$ $[M+H]^+$ 258.1858, found 258.1861.

IR: ν_{max} = 3272 (N-H), 2970 (C-H), 2925 (C-H), 1662 (C=O), 1623 (C=C), 1453, 1364, 1033.

(2*E*,4*E*)-*N*-(isopropyl)-7-phenylocta-2,4-dienamide (11e)

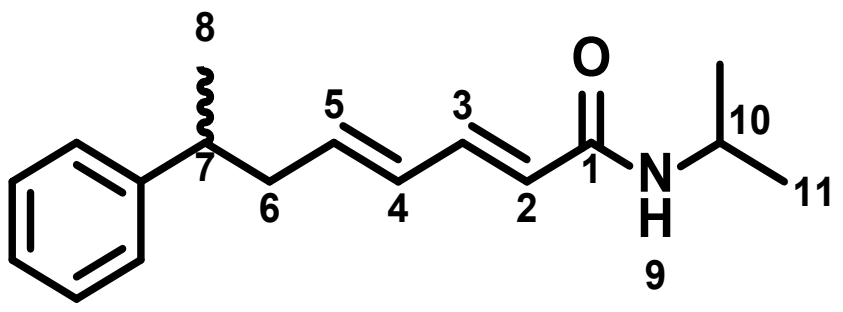

Synthesised according to General Procedure 2 on a 0.39 mmol using (*S,E*)-*N*-isopropyl-*N*-(1-phenylethyl)hexa-3,5-dienamide **(8e)** (purification with an eluent of 5-20% EtOAc / petroleum ether) to give the title compound as a colourless oil with an NMR yield of 27%, isolated yield of 20%. Enantiomeric ratio determined, through the use of chiral HPLC Chiralcel® OJ column with hexane:2-propanol (93:7) as eluent, as 50:50 with retention time (mins) 11.26:12.75.

^{1}H NMR (400 MHz, Chloroform-*d*): δ 7.31-7.26 (2H, m, Ar), 7.20-7.15 (3H, m, Ar), 7.11 (1H, dd, *J* = 15.4, 10.8 Hz, H3), 6.08 (1H, dd, *J* = 15.0, 10.4 Hz, H4), 5.94 (1H, td, *J* = 14.7, 7.4 Hz, H5), 5.69 (1H, d, *J* = 15.0 Hz, H2), 5.40 (1H, br d, *J* = 7.3 Hz, H9), 4.19-4.09 (1H, m, H10), 2.86-2.78 (1H, m, H7), 2.50-2.32 (2H, m, H6), 1.25 (3H, d, *J* = 7.1 Hz, H8), 1.16 (6H, d, *J* = 6.6 Hz, H11).

^{13}C NMR (101 MHz, Chloroform-*d*): δ 167.2 (C1), 147.0 (Ar), 141.0 (C5) 141.0 (Ar), 129.8 (C4), 128.5 (Ar), 127.0 (Ar), 126.3 (C3), 122.5 (C2), 41.9 (C6), 41.5 (C10), 39.9 (C7), 23.3 (C11), 21.6 (C8).

HRMS (ESI^{+}) *m/z* calcd for $C_{17}H_{24}NO$ $[M+H]^{+}$ 258.1858, found 258.1861.

IR: v_{max} = 3280 (N-H), 2968 (C-H), 2923 (C-H), 1654 (C=O), 1627 (C=C), 1539, 1453, 1003.

N-Benzhydrylpropan-2-amine

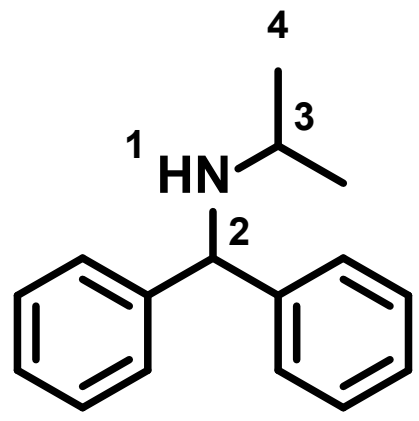

Synthesised according to a modified procedure from Gilbert *et. al.*[17]

To a solution of benzophenone (5.49 mmol, 1.0 eq.) in anhydrous CH_2Cl_2 (6 mL) was added $TiCl_4$ (6.10 mmol, 1.1 eq., 1 M solution in CH_2Cl_2) in an ice bath. After 10 minutes stirring at room temperature, a solution of $^{i}PrNH_2$ (12.80 mmol, 2.3 eq.) in anhydrous THF (6 mL) was added slowly and the mixture left to stir for 3 hours. A solution of $NaBH_3CN$ (6.52 mmol, 1.2 eq.) in anhydrous THF (6.5 mL) was added and the mixture left to stir for 16 hours. The reaction was quenched with 1 M $NaOH_{(aq.)}$ (50 mL) and left to stir for 30 minutes before the mixture was filtered through celite. The aqueous layer was extracted with EtOAc (3 × 30 mL). The combined organics were washed with brine (60 mL), dried with $MgSO_4$, filtered and concentrated *in vacuo*. Purification by flash column chromatography (5% EtOAc / petroleum ether) afforded the title compound as a colourless oil (3.95 mmol, 72%).

^{1}H NMR (400 MHz, Chloroform-*d*): δ 7.45-7.40 (4H, m, Ar), 7.36-7.30 (4H, m, Ar), 7.26-7.20 (2H, m, Ar), 5.02 (1H, s, H2), 2.80 (1H, sept, *J* = 6.6 Hz, H3), 1.13 (6H, d, *J* = 6.6 Hz, H4).

^{13}C NMR (101 MHz, Chloroform-*d*): δ 144.7 (Ar), 130.2 (Ar), 128.5 (Ar), 127.5 (Ar), 126.9 (Ar), 64.3 (C2), 46.2 (C3), 23.3 (C4).

Data consistent with literature.[17]

(*E*)-*N*-benzhydryl-*N*-isopropylhexa-3,5-dienamide (8f)

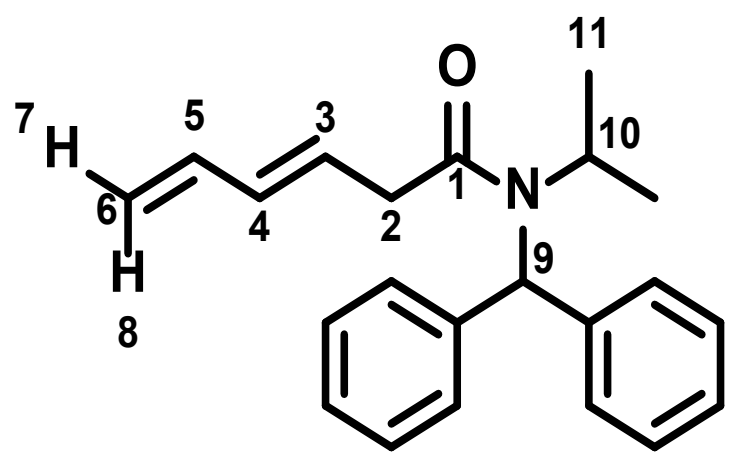

(*E*)-hexa-3,5-dienoic acid (2.95 mmol, 1.1 eq.) was dissolved in anhydrous CH_2Cl_2 (4 mL) with a few drops of DMF. Oxalyl chloride (3.48 mmol, 1.3 eq.) was added slowly to give a yellow solution before being left to stir for 2 hours. Excess volatiles were removed *in vacuo* before addition of anhydrous CH_2Cl_2 (9 mL) and *N*-benzhydrylpropan-2-amine (2.68 mmol, 1.0 eq.). The mixture was left to stir for 16 hours before addition of water (20 mL) and the layers separated. The aqueous layer was washed with CH_2Cl_2 (3 × 20 mL) before the combined organics were washed with $NaHCO_{3(aq.)}$ (2 × 20 mL). The organics were dried with $MgSO_4$, filtered and concentrated *in vacuo*. The residue was purified by flash column chromatography (0-10% EtOAc / petroleum Ether) to afford the title compound as a yellow oil (0.96 mmol, 36%).

NMR considerably broad due to rotamers present. Reported data was recorded at 50 °C.

^{1}H NMR (500 MHz, Chloroform-*d*): δ 7.40-7.25 (10H, m, Ar), 6.31 (1H, dt, *J* = 16.7, 10.2 Hz, H5), 6.13-6.03 (1H, br s, H9), 6.03-5.90 (1H, br m, H4), 5.81 (1H, br dt, *J* = 14.8, 6.7 Hz, H3), 5.09 (1H, d, *J* = 16.7 Hz, H8), 5.01 (1H, d, *J* = 10.2 Hz, H7), 4.25-4.05 (1H, br m, H10), 3.17-2.95 (1.8H, br m, H2), 1.23 (6H, d, *J* = 6.5 Hz, H11).

^{13}C NMR (101 MHz, Chloroform-*d*): δ 171.2 (C1), 136.9 (C5), 133.6 (C4), 130.5 (C9), 129.0 (Ar), 128.9 (Ar), 128.5 (Ar), 128.5 (Ar), 128.0 (C3), 116.2 (C6), 49.0 (C10), 40.2 (C2), 21.8 (C11).

HRMS (ESI$^+$) *m/z* calcd for $C_{22}H_{26}NO$ $[M+H]^+$ 320.2009, found 320.2013.

IR: v_{max} = 2980 (C-H), 2971 (C-H), 1650 (C=O), 1634 (C=C), 1599 (C=C), 1430, 1002.

(*E*)-2-benzhydryl-*N*-isopropylhexa-3,5-dienamide (9f)

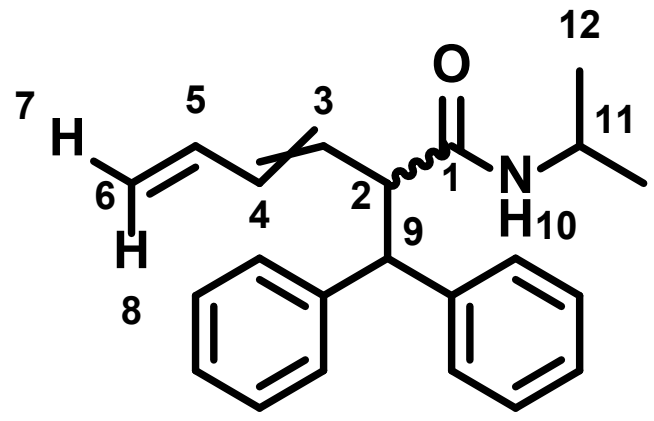

Synthesised according to General Procedure 2 on a 0.31 mmol scale using (*E*)-*N*-benzhydryl-*N*-isopropylhexa-3,5-dienamide **(8f)** (purification with an eluent of 5-10% EtOAc / petroleum ether) to give a mix of *E* and *Z* isomers in a ratio of 1:0.12 of the title compound as a white solid, with an isolated yield of 7%. *E* and *Z* in the assignments below correspond to the stereochemistry of the molecule and not the atom itself. Peaks corresponding to the *Z*-isomer were not seen in the ^{13}C NMR spectra, due to low concentration of this isomer in the sample. Reported signals are for the *E*-isomer of the title compound.

^{1}H NMR (400 MHz, Chloroform-*d*): δ 7.32-7.11 (20H, m, Ar), 6.63 (1H, dt, *J* = 16.6, 10.1 Hz, *Z*-H5), 6.17 (1H, dt, *J* = 16.1, 10.6 Hz, *E*-H5), 6.05 (2H, 2 × overlapping dd, *J* = 15.5, 10.6 Hz, *E* & *Z*-H4), 5.67 (1H, dd, *J* = 15.5, 8.5 Hz, *E*-H3), 5.47 (1H, t, *J* = 10.3 Hz, *Z*-H3), 5.23 (1H, d, *J* = 16.6 Hz, *Z*-H8), 5.19 (1H, d, *J* = 10.1 Hz, *Z*-H7), 5.06 (1H, dd, *J* = 16.1, 1.8 Hz, *E*-H8), 5.06 (2H, br s, *E* & *Z*-H10), 4.96 (1H, dd, *J* = 10.6, 1.8 Hz, *E*-H7), 4.54 (1H, d, *J* = 10.1 Hz, *Z*-H9), 4.44 (1H, d, *J* = 10.9 Hz, *E*-H9), 3.94 (1H, t, *J* = 10.1 Hz, *Z*-H2), 3.84 (2H, sept, *J* = 6.8 Hz, *E* & *Z*-H11), 3.52 (1H, dd, *J* = 10.9, 8.5 Hz, *E*-H2), 0.98 (3H, d, *J* = 6.8 Hz, *E*-H12), 0.97 (3H, d, *J* = 6.4 Hz, *Z*-H12), 0.75 (3H, d, *J* = 6.4 Hz, *Z*-H12), 0.71 (3H, d, *J* = 6.8 Hz, *E*-H12).

^{13}C NMR (101 MHz, Chloroform-*d*): δ 171.2 (C1), 143.1 (Ar), 141.8 (Ar), 136.7 (C5), 134.0 (C4), 131.7 (C3), 128.8 (Ar), 128.6 (Ar), 128.5 (Ar), 128.2 (Ar), 126.7 (Ar), 126.6 (Ar), 116.9 (C6), 56.6 (C2), 54.0 (C9), 41.2 (C11), 22.6 (C12), 22.5 (C12).

HRMS (ESI$^+$) *m/z* calcd for $C_{22}H_{26}NO$ [M+H]$^+$ 320.2009, found 320.2009.

MP: 86-87 °C.

IR: v_{max} = 3293 (N-H), 2980 (C-H), 2888 (C-H), 1633 (C=O & C=C broad), 1382, 1153.

4-Benzhydryl-*N*-isopropylhexa-2,5-dienamide (10f)

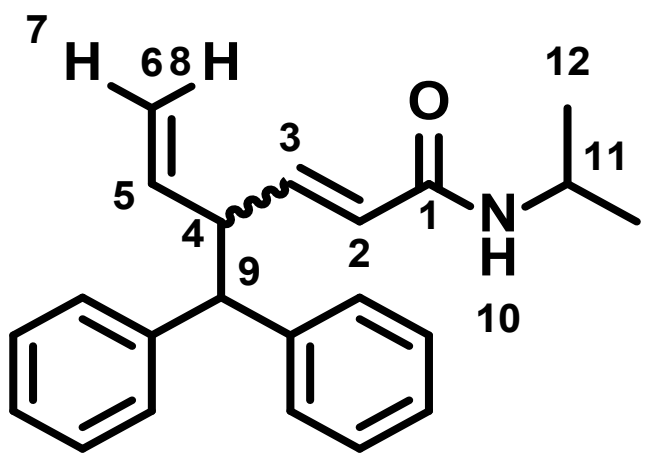

Synthesised according to General Procedure 2 on a 0.31 mmol scale using (*E*)-*N*-benzhydryl-*N*-isopropylhexa-3,5-dienamide **(8f)** (purification with an eluent of 5-10% EtOAc / petroleum ether) to give a yellow oil which is an inseparable mixture of [1,5] and [1,7]-migration product, with a yield of 6% for the title compound.

^{1}H NMR (400 MHz, Chloroform-*d*): δ 7.29-7.11 (10H, m, Ar), 6.69 (1H, dd, *J* = 15.6, 7.7 Hz, H3), 5.67-5.61 (1H, m, H5), 5.61-5.59 (1H, d, *J* = 15.6 Hz, H2), 5.20 (1H, br d, *J* = 7.9 Hz, H10), 5.00 (1H, dd, *J* = 16.2, 1.3 Hz, H8), 4.98 (1H, d, *J* = 10.7, 1.3 Hz, H7), 4.06 (1H, sept, *J* = 6.6 Hz, H11), 3.95 (1H, d, *J* = 10.5 Hz, H9), 3.77-3.69 (1H, m, H4), 1.10 (3H, d, *J* = 6.6 Hz, H12), 1.09 (3H, d, *J* = 6.6 Hz, H12).

^{13}C NMR (101 MHz, Chloroform-*d*): δ 164.8 (C1), 144.3 (Ar), 138.3 (C5), 128.7 (Ar), 128.5 (Ar), 128.5 (C3), 126.6 (Ar), 125.2 (C2), 117.3 (C6), 56.2 (C9), 41.4 (C11), 50.4 (C4), 22.9 (C12).

HRMS (ESI^{+}) *m/z* calcd for $C_{22}H_{26}NO$ $[M+H]^{+}$ 320.2009, found 320.2010.

IR: v_{max} = 3280 (N-H), 2980 (C-H), 1657 (C=O), 1626 (C=C), 1539, 1382, 1251, 1154.

(2*E*,4*E*)-*N*-isopropyl-7,7-diphenylhepta-2,4-dienamide (11f)

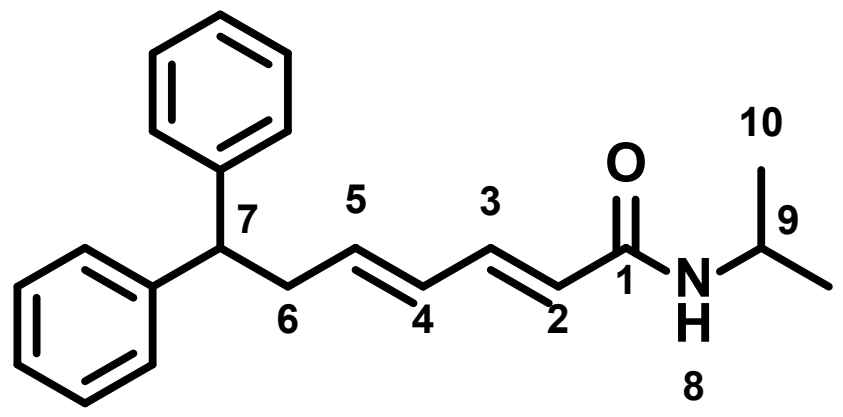

Synthesised according to General Procedure 2 on a 0.31 mmol scale using (*E*)-*N*-benzhydryl-*N*-isopropylhexa-3,5-dienamide **(8f)** (purification with an eluent of 5-10% EtOAc / petroleum ether) to give a yellow oil which is an inseparable mixture of [1,5] and [1,7]-migration product, with a yield of 23% for the title compound.

^{1}H NMR (400 MHz, Chloroform-*d*): δ 7.28-7.11 (10H, m, Ar), 7.04 (1H, dd, *J* = 15.7, 10.9 Hz, H3), 6.09 (1H, dd, *J* = 15.5, 10.9 Hz, H4), 5.93 (1H, dt, *J* = 15.5, 7.3 Hz, H5), 5.65 (1H, d, *J* = 15.7 Hz, H2), 5.37 (1H, br d, *J* = 8.2 Hz, H8), 4.12 (1H, sept, *J* = 7.8 Hz, H9), 4.01 (1H, t, *J* = 7.3 Hz, H7), 2.89 (2H, t, *J* = 7.3 Hz, H6), 1.14 (6H, d, *J* = 6.6 Hz, H10).

^{13}C NMR (101 MHz, Chloroform-*d*): δ 165.5 (C1), 144.2 (Ar), 140.7 (C3), 140.3 (C5), 130.0 (C4), 128.6 (Ar), 128.0 (Ar), 126.5 (Ar), 122.8 (C2), 51.2 (C7), 41.5 (C9), 39.2 (C6), 22.9 (C10).

HRMS (ESI$^+$) *m/z* calcd for $C_{22}H_{26}NO$ $[M+H]^+$ 320.2009, found 320.2010.

IR: ν_{max} = 3280 (N-H), 2980 (C-H), 1657 (C=O), 1626 (C=C), 1539, 1382, 1251, 1154.

(*E*)-*N*-allyl-*N*-(*tert*-butyl)hexa-3,5-dienamide (8g)

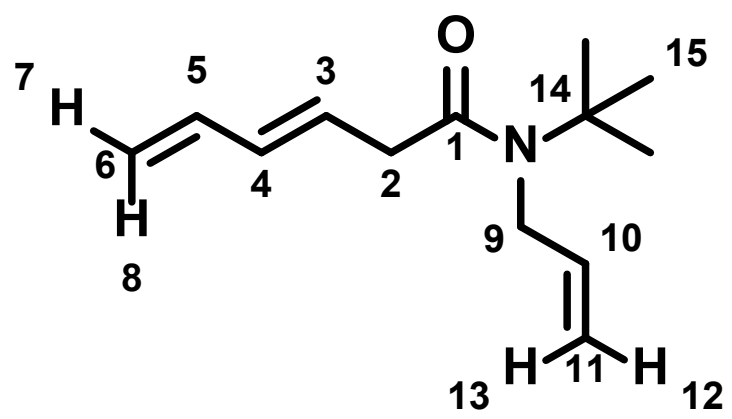

(*E*)-hexa-3,5-dienoic acid (8.92 mmol, 1.1 eq.) was dissolved in anhydrous CH_2Cl_2 (14 mL) with a few drops of DMF. Oxalyl chloride (10.55 mmol, 1.3 eq.) was added slowly to give an orange solution before being left to stir for 2 hours. Excess volatiles were removed *in vacuo* before addition of anhydrous CH_2Cl_2 (28 mL) and *N*-(*tert*-butyl)prop-2-en-1-amine (8.11 mmol, 1.0 eq.). The mixture was left to stir for 16 hours before addition of water (20 mL) and the layers separated. The aqueous layer was washed with CH_2Cl_2 (3 × 30 mL) before the combined organics were washed with $NaHCO_{3(aq.)}$ (2 × 20 mL). The organics were dried with $MgSO_4$, filtered and concentrated *in vacuo*. The residue was purified by flash column chromatography (0-1% MeOH / CH_2Cl_2) to afford the title compound as an orange oil (1.95 mmol, 24%).

^{1}H NMR (400 MHz, Chloroform-*d*): δ 6.32 (1H, dt, *J* = 17.3, 10.0 Hz, H5), 6.05 (1H, dd, *J* = 15.7, 10.3 Hz, H4), 5.88-5.78 (2H, m, H3, H10), 5.23-5.16 (2H, m, H12, H13), 5.09 (1H, d, *J* = 17.3 Hz, H8), 4.99 (1H, d, *J* = 10.4 Hz, H7), 3.94-3.91 (2H, m, H9), 3.10 (2H, d, *J* = 7.0 Hz, H2), 1.42 (9H, s, H15).

^{13}C NMR (101 MHz, Chloroform-*d*): δ 172.2 (C1), 136.9 (C5), 136.1 (C3/C10), 133.2 (C4), 128.5 (C3/C10), 116.2 (C6), 116.0 (C11), 57.7 (C14), 47.4 (C9), 40.0 (C2), 28.8 (C15).

HRMS (ESI$^+$) *m/z* calcd for $C_{13}H_{22}NO$ $[M+H]^+$ 208.1696, found 208.1690.

IR: ν_{max} = 2963 (C-H), 2925 (C-H), 1715 (C=O), 1625 (C=C), 1393, 1361, 1194.

(*E*)-2-allyl-*N*-(*tert*-butyl)hexa-3,5-dienamide (9g)

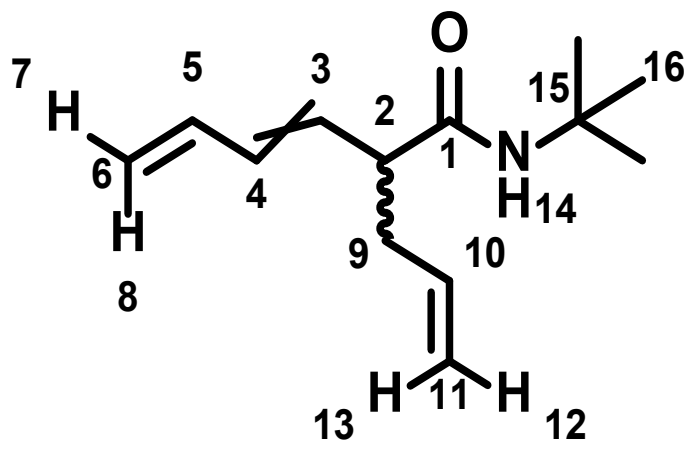

Synthesised according to General Procedure 2 on a 0.48 mmol scale using (*E*)-*N*-allyl-*N*-(*tert*-butyl)hexa-3,5-dienamide **(8g)** (purification with an eluent of 0-10% EtOAc / petroleum ether) to give a mixture of *E* and *Z* isomers (in a ratio of 1:0.45 respectively) of the title compound as a colourless oil with an isolated yield of 14%. *E* and *Z* in the assignments refer to the stereochemistry of the molecule and not the individual atom.

^{1}H NMR (400 MHz, Chloroform-*d*): δ 6.55 (1H, dt, *J* = 17.9, 9.3 Hz, *Z*-H5), 6.32 (1H, dt, *J* = 17.9, 8.3 Hz, *E*-H5), 6.21-6.08 (2H, m, *E* & *Z*-H4), 5.78-5.64 (3H, m, *E*-H3, *E* & *Z*-H10), 5.45 (1H, t, *J* = 10.3 Hz, *Z*-H3), 5.35-5.21 (3H, m, *Z*-H13, *Z*-H7, *Z*-H8), 5.17 (1H, dd, *J* = 17.8, 1.8 Hz, *E*-H8), 5.09-5.04 (2H, m, *Z*-H12, *E*-H7), 5.04-4.99 (2H, m, *E*-H12, *E*-H13), 3.27-3.20 (1H, m, *Z*-H2), 2.77 (1H, q, *J* = 7.9 Hz, *E*-H2), 2.59-2.49 (2H, m, *E* & *Z*-H9), 2.33-2.24 (2H, m, *E* & *Z*-H6), 1.32 (9H, s, *E*-H16), 1.30 (9H, s, *Z*-H16).

^{13}C NMR (101 MHz, Chloroform-*d*): δ 171.9 (*E*-C1), 171.6 (*Z*-C1), 136.6 (*E*-C5), 135.7 (*E*-C10), 135.7 (*Z*-C10), 133.5 (*E*-C4), 132.6 (*E*-C3), 132.0 (*Z*-C4), 131.6 (*Z*-C5), 130.3 (*Z*-C3), 119.8 (*Z*-C6), 117.2 (*E*-C6), 117.0 (*Z*-11), 116.9 (*E*-C11), 51.4 (*E*-C2), 46.3 (*Z*-C2), 36.9 (*Z*-C9), 36.8 (*E*-C9), 28.9 (*E*-C16), 28.8 (*Z*-C16).

HRMS (ESI$^+$) *m/z* calcd for $C_{13}H_{22}NO$ $[M+H]^+$ 208.1696, found 208.1689.

IR: ν_{max} = 3319 (N-H), 2961 (C-H), 2923 (C-H), 1643 (C=O), 1538 (C=C), 1365, 1221.

(*E*)-*N*-(*tert*-butyl)-4-vinylhepta-2,6-dienamide (10g)

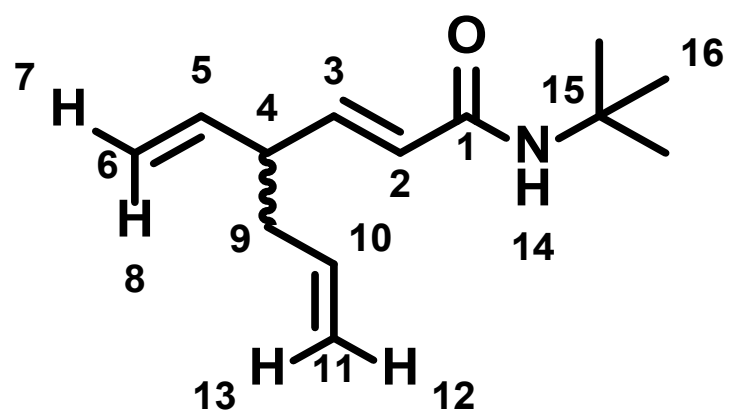

Synthesised according to General Procedure 2 on a 0.48 mmol scale using (*E*)-*N*-allyl-*N*-(*tert*-butyl)hexa-3,5-dienamide **(8g)** (purification with an eluent of 0-10% EtOAc / petroleum ether) to give the title compound as a colourless oil with an isolated yield of 6%.

^{1}H NMR (400 MHz, Chloroform-*d*): δ 6.73 (1H, dd, *J* = 15.3, 7.3 Hz, H3), 5.79-5.64 (3H, m, H2, H5, H10), 5.27 (1H, br s, H15), 5.09-5.00 (4H, m, H7, H8, H12, H13), 2.91 (1H, pent, *J* = 7.6 Hz, H4), 2.24 (2H, t, *J* = 7.6 Hz, H9), 1.38 (9H, s, H16).

^{13}C NMR (101 MHz, Chloroform-*d*): δ 165.2 (C1), 145.0 (C3), 139.1 (C2/C5/C10), 135.8 (C2/C5/C10), 124.8 (C2/C5/C10), 116.9 (C6/C11), 115.9 (C6/C11), 51.4 (C15), 46.0 (C4), 38.6 (C9), 29.0 (C16).

HRMS (ESI$^+$) *m/z* calcd for $C_{13}H_{22}NO$ [M+H]$^+$ 208.1696, found 208.1689.

IR: ν_{max} = 3287 (N-H), 3077 (C-H), 2967 (C-H), 2925 (C-H), 1664 (C=O), 1627 (C=C), 1547 (C=C), 1360, 1224, 986, 912.

(2*E*,4*E*)-*N*-(*tert*-butyl)nona-2,4,8-trienamide (11g)

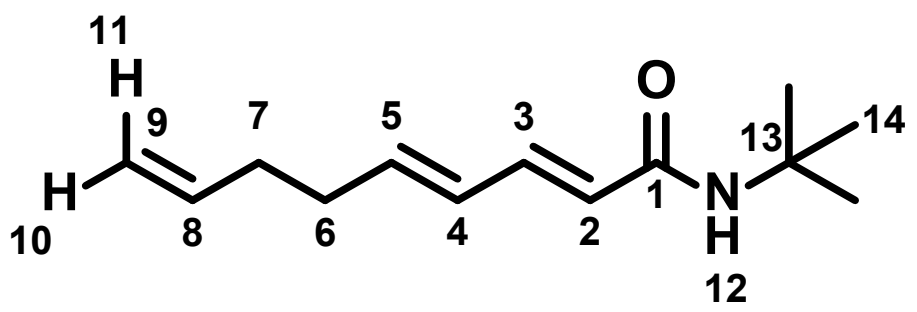

Synthesised according to General Procedure 2 on a 0.48 mmol scale using (*E*)-*N*-allyl-*N*-(*tert*-butyl)hexa-3,5-dienamide **(8g)** (purification with an eluent of 0-10% EtOAc / petroleum ether) to give the title compound as a white crystalline solid with an isolated yield of 9%.

^{1}H NMR (400 MHz, Chloroform-*d*): δ 7.12 (1H, dd, *J* = 15.1, 10.6 Hz, H3), 6.12 (1H, dd, *J* = 15.2, 10.6 Hz, H4), 6.06-5.97 (1H, m, H5), 5.78 (1H, ddt, *J* = 17.1, 10.6, 6.9 Hz, H8), 5.69 (1H, d, *J* = 15.0 Hz, H2), 5.31 (1H, br s, H12), 5.01 (1H, dd, *J* = 17.1, 1.6 Hz, H11), 4.97 (1H, dd, *J* = 10.6, 1.6 Hz, H10), 2.27-2.12 (4H, m, H6, H7), 1.38 (9H, s, H14).

^{13}C NMR (101 MHz, Chloroform-*d*): δ 165.8 (C1), 141.6 (C5), 140.5 (C3), 137.8 (C8), 128.8 (C4), 123.4 (C2), 115.8 (C9), 51.4 (C13), 33.8 (C6/C7), 32.4 (C6/C7), 29.0 (C14).

HRMS (ESI$^+$) *m/z* calcd for $C_{13}H_{22}NO$ $[M+H]^+$ 208.1696, found 208.1689.

MP: 82-83 °C.

IR: v_{max} = 3261 (N-H), 3074 (C-H), 2954 (C-H), 2923 (C-H), 1715 (C=O), 1611 (C=C), 1652 (C=C), 1626 (C=C), 1550, 1361, 1223, 1005.

(*E*)-*N*-(*tert*-butyl)-*N*-methylhexa-3,5-dienamide (8h)

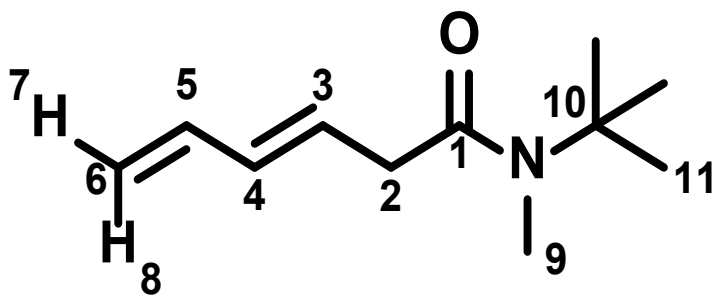

Synthesised according to General Procedure 1 on an 8.92 mmol scale to give a colourless oil (5.26 mmol, 59%), using (*E*)-hexa-3,5-dienoic acid and *N*-methyl-2-methylpropan-2-amine as reagents. Purification was carried out with an eluent of 5-10% EtOAc / petroleum ether.

^{1}H NMR (400 MHz, Chloroform-*d*): δ 6.33 (1H, dt, *J* = 17.0, 10.1 Hz, H5), 6.07 (1H, dd, *J* = 15.3, 10.3 Hz, H4), 5.82 (1H, dt, *J* = 15.4, 7.5 Hz, H3), 5.09 (1H, dd, *J* = 16.9, 1.7 Hz, H8), 4.99 (1H, dd, *J* = 10.1, 1.8 Hz, H7), 3.12 (1H, d, *J* = 7.4 Hz, H2), 2.87 (3H, s, H9), 1.38 (9H, s, H11).

^{13}C NMR (101 MHz, Chloroform-*d*): δ 171.5 (C1), 136.9 (C5), 133.4 (C4), 128.1 (C3), 116.1 (C6), 57.0 (C10), 41.0 (C2), 32.3 (C9), 28.4 (C11).

HRMS (ESI^{+}) *m/z* calcd for $C_{11}H_{20}NO$ [M+H]$^{+}$ 182.1545, found 182.1548.

IR: v_{max} = 2964 (C-H), 2924 (C-H), 1636 (C=O & C=C within peak), 1379, 1364, 1212, 1107, 1004 cm^{-1}.

(*E*)-*N*-(*tert*-butyl)-*N*-isopropylhexa-3,5-dienamide (8i)

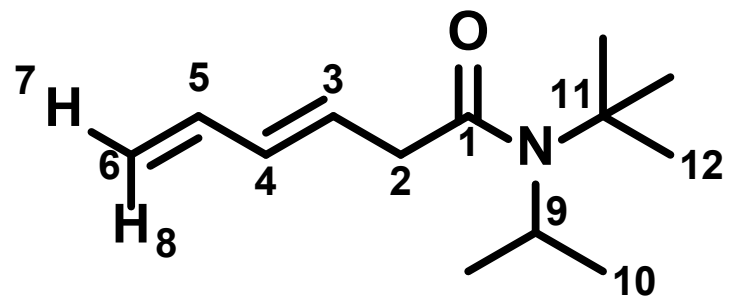

Synthesised according to General Procedure 1 on an 8.92 mmol scale to give a colourless oil (1.07 mmol, 12%), using (*E*)-hexa-3,5-dienoic acid and *N*-isopropyl-2-methylpropan-2-amine as reagents. Purification was carried out with an eluent of 5-10% EtOAc / petroleum ether.

^{1}H NMR (400 MHz, Chloroform-*d*): δ 6.29 (1H, dt, *J* = 17.0, 10.2 Hz, H5), 6.07 (1H, dd, *J* = 15.3, 10.3 Hz, H4), 5.83 (1H, dt, *J* = 15.4, 7.5 Hz, H3), 5.07 (1H, dd, *J* = 17.0, 1.7 Hz, H8), 4.95 (1H, dd, *J* = 10.3, 1.7 Hz, H7), 3.91 (1H, sept, *J* = 7.0 Hz, H9), 3.14 (2H, d, *J* = 7.0 Hz, H2), 1.93 (9H, s, H12), 1.33 (6H, d, *J* = 7.1 Hz, H10).

^{13}C NMR (101 MHz, Chloroform-*d*): δ 177.2 (C1), 137.1 (C5), 133.2 (C4), 129.4 (C3), 116.0 (C6), 47.0 (C9), 46.0 (C11), 42.3 (C2), 30.4 (C12), 27.3 (C10).

HRMS (ESI$^+$) *m/z* calcd for $C_{13}H_{24}NO$ $[M+H]^+$ 210.1858, found 210.1857.

IR: ν_{max} = 2972 (C-H), 2960 (C-H), 1622 (C=O), 1567 (C=C), 1382, 1370, 1176, 1003 cm^{-1}.

(*E*)-*N*-isopropyl-*N*-phenylhexa-3,5-dienamide (8j)

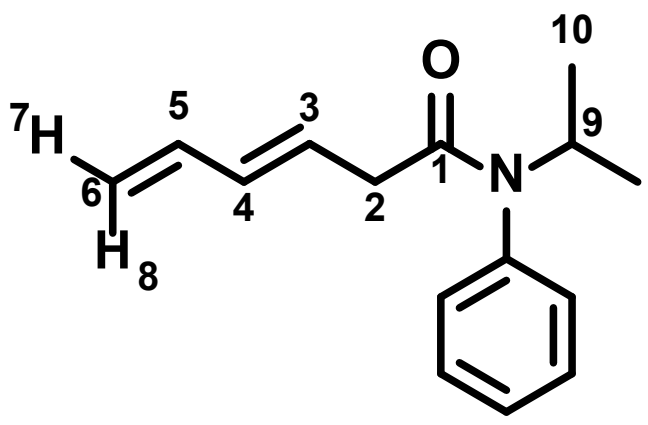

(*E*)-hexa-3,5-dienoic acid (8.92 mmol, 1.1 eq.) was dissolved in anhydrous CH_2Cl_2 (13 mL) with a few drops of DMF. Oxalyl chloride (11.59 mmol, 1.3 eq.) was added slowly to give a yellow solution before being left to stir for 2 hours. Excess volatiles were removed *in vacuo* before addition of anhydrous CH_2Cl_2 (25 mL) and *N*-isopropylaniline (8.03 mmol, 1.0 eq.). The mixture was left to stir for 16 hours before addition of water (20 mL) and the layers separated. The aqueous layer was washed with CH_2Cl_2 (3 × 30 mL) before the combined organics were washed with $NaHCO_{3(aq.)}$ (3 × 20 mL). The organics were dried with $MgSO_4$, filtered and concentrated *in vacuo*. The residue was purified by flash column chromatography (0-5% EtOAc / petroleum Ether) to afford the title compound as a yellow oil (3.92 mmol, 44%).

^{1}H NMR (400 MHz, Chloroform-*d*): δ 7.43-7.35 (3H, m, Ar), 7.09-7.06 (2H, m, Ar), 6.26 (1H, dt, *J* = 17.0, 9.9 Hz, H5), 5.85-5.70 (2H, m, H3 & H4), 5.02 (1H, dd, *J* = 16.0, 1.1 Hz, H8), 5.00-4.93 (1H, m, H9), 4.93 (1H, dd, *J* = 9.8, 1.1 Hz, H7), 2.73 (2H, d, *J* = 6.5 Hz, H2), 1.02 (6H, d, *J* = 6.8 Hz, H10).

^{13}C NMR (101 MHz, Chloroform-*d*): δ 170.2 (C1), 138.5 (Ar), 136.8 (C5), 134.6 (C3/C4), 131.3 (Ar), 129.2 (Ar), 128.4 (Ar), 127.9 (C3/C4), 116.9 (C6), 45.7 (C9), 39.1 (C2), 21.0 (C10).

HRMS (ESI^+) *m/z* calcd for $C_{15}H_{20}NO$ $[M+H]^+$ 230.1539, found 230.1542.

IR: ν_{max} = 2973 (C-H), 1645 (C=O), 1592 (C=C), 1494, 1391, 1118, 703 cm^{-1}.

Phenyl((1*R*,2*R*)-2-phenylcyclopropyl)methanone

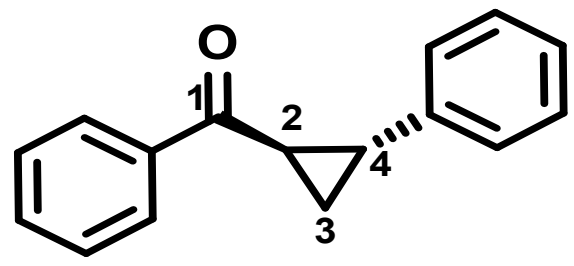

Synthesised according to the procedure by Ciaccio *et al.* in a modified Corey-Chaykovsky reaction.[18]

To a slurry of NaH (17.30 mmol, 1.2 eq., 60% in mineral oil) in anhydrous DMSO (15 mL) was added trimethylsulfoxonium iodide (17.30 mmol, 1.2 eq.) and the mixture left to stir for 1 hour. A solution of trans-chalcone (14.42 mmol, 1.0 eq.) in anhydrous DMSO (28 mL) was added slowly to give a clear, orange solution. The mixture was left to stir for 16 hours before addition of brine (50 mL). The aqueous layer was extracted with Et_2O (3 × 30 mL) before the combined organics were washed with water (2 × 50 mL), brine (50 mL), dried with $MgSO_4$, filtered and concentrated *in vacuo*. Purification by flash column chromatography (0-5% EtOAc / petroleum ether) afforded a peach-coloured solid (8.65 mmol, 60%).

^{1}H NMR (400 MHz, Chloroform-*d*): δ 8.03-7.97 (2H, m, Ar), 7.56 (1H, tt, *J* = 7.4, 1.3 Hz, Ar), 7.49-7.44 (2H, m, Ar), 7.35-7.29 (2H, m, Ar), 7.24 (1H, tt, *J* = 7.2, 1.3 Hz, Ar), 7.21-7.17 (2H, m, Ar), 2.91 (1H, ddd, *J* = 9.3, 5.3, 4.1 Hz, H2), 2.71 (1H, ddd, *J* = 10.6, 6.7, 4.1 Hz, H4), 1.94 (1H, ddd, *J* = 9.5, 5.3, 4.1 Hz, H3), 1.56 (1H, ddd, *J* = 10.6, 6.7, 4.1 Hz, H3).

^{13}C NMR (101 MHz, Chloroform-*d*): δ 198.7 (C1), 140.6 (Ar), 137.9 (Ar), 133.0 (Ar), 128.7 (Ar), 128.6 (Ar), 128.3 (Ar), 126.7 (Ar), 126.4 (Ar), 30.1 (C4), 29.4 (C2), 19.4 (C3).

Data is consistent with literature.[18]

N-(phenyl((1*R*,2*R*)-2-phenylcyclopropyl)methyl)propan-2-amine

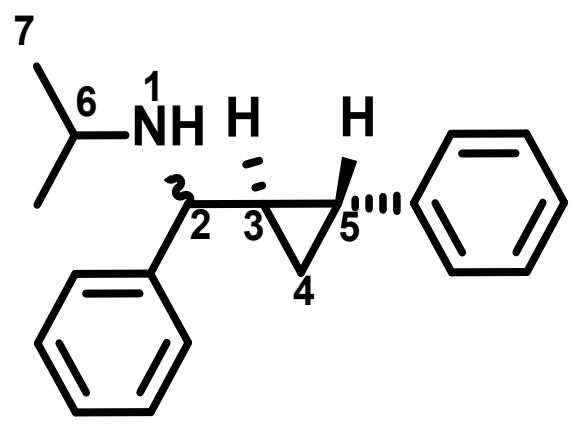

To a solution of phenyl((1*R*,2*R*)-2-phenylcyclopropyl)methanone (5.40 mmol, 1.0 eq.) in anhydrous CH_2Cl_2 (25 mL) was added $^iPrNH_2$ (21.61 mmol, 4.0 eq.). In an ice bath, $TiCl_4$ (10.81 mmol, 2.0 eq., 1 M solution in CH_2Cl_2) was added slowly and the mixture stirred for 16 hours at room temperature. A solution of $NaBH_3CN$ (6.48 mmol, 1.2 eq.) in anhydrous THF (10 mL) was added and the mixture left to stir for 4 hours. The reaction was quenched with 1 M $NaOH_{(aq.)}$ (40 mL) before the aqueous layer was extracted with CH_2Cl_2 (3 × 30 mL). The combined organics were washed with water (30 mL), brine (40 mL), dried with Na_2SO_4, filtered and concentrated *in vacuo* to afford an orange oil (4.70 mmol, 87%) as a mixture of 2 diastereomers (1:1.40). This was used without purification in the next step.

^{1}H NMR (400 MHz, Chloroform-*d*): δ 7.40-7.04 (18H, m, Ar), 6.91 (2H, d, *J* = 7.4 Hz, Ar), 4.25 (2H, br s, H1 D1, D2), 3.79-3.72 (1H, m, H2 D1), 3.29 (1H, dd, *J* = 8.3, 4.3 Hz, H2 D2), 3.13 (1H, sept, *J* = 6.8 Hz, H6 D1), 2.75-2.66 (1H, m, H6 D2), 1.89-1.81 (2H, m, H3 D1, D2), 1.48-1.34 (2H, m, H5 D1, D2), 1.27 (8H, d, *J* = 6.9 Hz, H7 D1), 1.06-1.00 (8H, m, H4 D1, H7 D2), 1.00-0.91 (1H, m, H4 D2), 0.83 (1H, dt, *J* = 8.8, 5.2 Hz, H4 D2).

^{13}C NMR (101 MHz, Chloroform-*d*): δ 144.1 (Ar), 144.0 (Ar), 143.0 (Ar), 142.7 (Ar), 128.5 (Ar), 128.5 (Ar), 128.4 (Ar), 128.3 (Ar), 127.4 (Ar), 127.2 (Ar), 127.2 (Ar), 127.1 (Ar), 126.3 (Ar), 125.9 (Ar), 125.6 (Ar), 125.5 (Ar), 68.1 (C2 D1), 64.9 (C2 D2), 49.6 (C6 D1), 45.8 (C6 D2), 31.0 (C5 D1/D2), 30.4 (C5 D1/D2), 24.1 (C7 D2), 23.6 (C3 D1/D2), 22.6 (C7 D2), 21.6 (C7 D1), 21.2 (C3 D1/D2), 15.3 (C4 D1), 14.0 (C4 D2).

HRMS (ESI$^+$) *m/z* calcd for $C_{19}H_{24}N$ $[M+H]^+$ 266.1903, found 266.1906.

IR: ν_{max} = 3658 (N-H), 2979 (C-H), 2970 (C-H), 1496, 1364, 1088.

(*E*)-*N*-isopropyl-*N*-(phenyl((1*R*,2*R*)-2-phenylcyclopropyl)methyl)hexa-3,5-dienamide (±12)

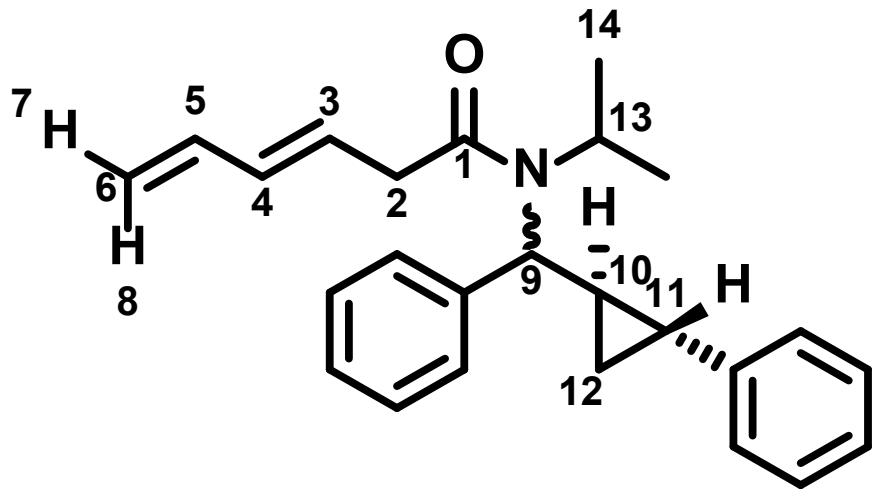

Synthesised according to General Procedure 1 on a 3.57 mmol scale to give a colourless oil (1.04 mmol, 29%) as a mixture of two diastereomers (~1:1), using (*E*)-hexa-3,5-dienoic acid and *N*-(phenyl((1*R*,2*R*)-2-phenylcyclopropyl)methyl)propan-2-amine as reagents. Purification was carried out with an eluent of 0-20% EtOAc / petroleum ether. D1 and D2 relate to the diastereomer of product. The NMR spectra is considerably broad, due to rotameric structure, meaning some signals are not observed clearly and some carbon signals are duplicated.

^{1}H NMR (400 MHz, Chloroform-*d*): δ 7.49-6.88 (20H, m, Ar), 6.28 (2H, dt, *J* = 17.1, 10.8 Hz, H5 D1, D2), 6.17-5.94 (2H, br m, H4 D1, D2), 5.94-5.81 (2H, m, H3 D1, D2), 5.05 (2H, dd, *J* = 17.1, 4.4 Hz, H8 D1, D2), 4.95 (2H, d, *J* = 10.4 Hz, H7 D1, D2), 4.26-4.06 (1H, br m, H9 D1), 3.41-3.23 (1H, br m, H13 D1), 3.23-3.09 (5H, m, H2 D1, D2, H9 D2), 2.60 (1H, sept, *J* = 6.1 Hz, H13 D2), 1.97 (1H, br s, H11 D1), 1.83 (1H, br s, H11 D2), 1.77-1.70 (2H, m, H10 D1, D2), 1.37-1.28 (1H, m, H12 D1/D2), 1.11-1.00 (7H, br m, H12 D1/D2, H14 D1), 0.92 (3H, d, *J* = 6.1 Hz, H14 D2), 0.90 (3H, d, *J* = 6.1 Hz, H14 D2), 0.83 (1H, dt, *J* = 8.9, 5.4 Hz, H12 D1/D2), 0.71 (1H, dt, *J* = 8.6 Hz, H12 D1/D2).

^{13}C NMR (101 MHz, Chloroform-*d*): δ 171.6 (C1 D1/D2), 170.4 (C1 D1/D2), 143.9 (Ar), 142.9 (Ar), 136.7 (br, C5 D1, D2), 133.7 (br, C4 D1, D2), 128.5 (Ar), 128.3 (C3 D1, D2), 128.3 (Ar), 128.2 (Ar), 127.7 (Ar), 127.3 (Ar), 127.0 (Ar), 125.8 (Ar), 125.6 (Ar), 125.5 (Ar), 116.6 (C6 D1, D2), 65.8 (C9 D1), 64.8 (C9 D2), 49.1 (C13 D1), 45.7 (C13 D2), 39.5 (C2 D1, D2), 31.8 (C12 D1/D2), 25.5 (C11 D2), 24.0 (C14 D2), 23.5 (C10 D1/D2), 22.6 (C14 D2), 22.5 (C11 D1), 20.2 (C12 D1/D2, C14 D1), 18.6 (C12 D1/D2), 15.0 (C12 D1/D2, C14 D1), 14.2 (C12 D1/D2), 13.8 (C12 D1/D2).

HRMS (ESI$^+$) *m/z* calcd for $C_{25}H_{30}NO$ [M+H]$^+$ 360.2322, found 360.2325.

IR: ν_{max} = 3027 (C-H), 2930 (C-H), 1630 (C=O), 1602 (C=C), 1496, 1356, 1185.

(2*E*,4*E*,9*E*)-*N*-isopropyl-7,10-diphenyldeca-2,4,9-trienamide (±13)

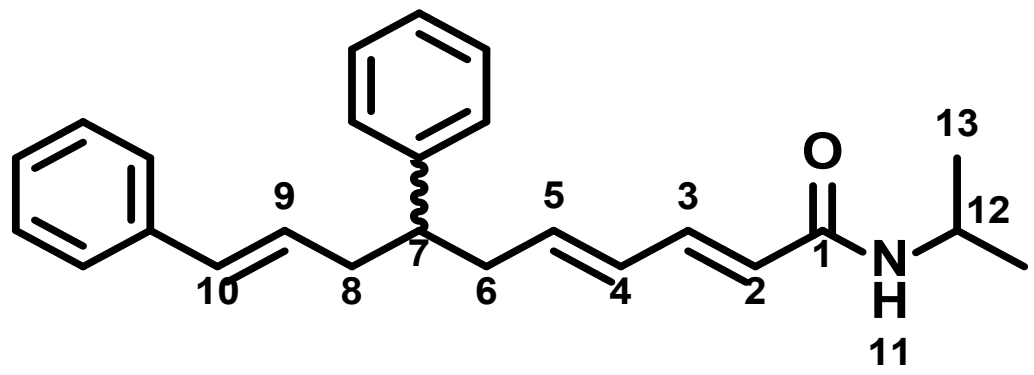

Synthesised according to General Procedure 2 on a 0.41 mmol scale using (*E*)-*N*-isopropyl-*N*-(phenyl((1*R*,2*R*)-2-phenylcyclopropyl)methyl)hexa-3,5-dienamide **(±12)** (purification with an eluent of 0-30% EtOAc / petroleum ether) to give the title compound as a colourless oil with an isolated yield of 26%.

^{1}H NMR (400 MHz, Chloroform-*d*): δ 7.20-7.05 (10H, m, Ar), 7.00 (1H, dd, *J* = 15.8, 10.4 Hz, H3), 6.27 (1H, dt, *J* = 15.8, 1.3 Hz, H10), 6.03-5.91 (2H, m, H5, H9), 5.88-5.77 (1H, m, H4), 5.59 (1H, d, *J* = 14.9 Hz, H2), 5.33 (1H, br d, *J* = 6.9 Hz, H11), 4.06 (1H, sept, *J* = 7.1 Hz, H12), 2.74 (1H, pent, *J* = 6.7 Hz, H7), 2.60-2.33 (4H, m, H6, H8), 1.07 (6H, d, *J* = 7.1 Hz, H13).

^{13}C NMR (101 MHz, Chloroform-*d*): δ 165.5 (C1), 144.1 (Ar), 140.7 (C3), 140.3 (C4), 137.7 (Ar), 131.8 (C10), 130.0 (C5/C9), 128.6 (Ar), 128.5 (Ar), 128.4 (C5/C9), 127.7 (Ar), 127.1 (Ar), 126.5 (Ar), 126.1 (Ar), 122.7 (C2), 46.0 (C7), 41.5 (C12), 39.7 (C6/C8), 39.4 (C6/C8), 22.9 (C13).

HRMS (ESI^{+}) *m/z* calcd for $C_{25}H_{30}NO$ [M+H]$^{+}$ 360.2322, found 360.2323.

IR: ν_{max} = 3281 (N-H), 2929 (C-H), 1651 (C=O), 1627 (C=C), 1496, 1385, 1130.

1-Methyl-6-phenylpiperidin-2-one

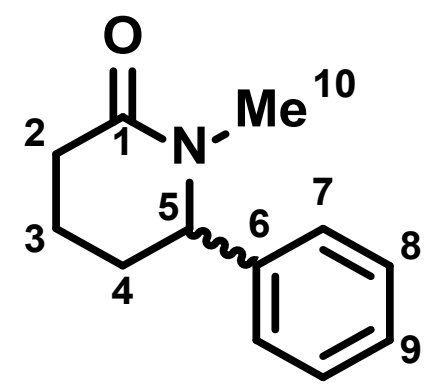

The title compound was synthesised according to General Procedure 3 using 5-oxo-5-phenylpentanoic acid (0.50 g, 2.60 mmol, 1.0 eq.) and *N*-methyl formamide (3.04 mL, 52.00 mmol, 20.0 eq.). Purification by flash column chromatography (20-50% EtOAc / petroleum ether) afforded a yellow oil (0.38 g, 2.03 mmol, 78%).

^{1}H NMR (400 MHz, Chloroform-*d*): δ 7.45 (2H, t, *J* = 7.3 Hz, H8), 7.37 (1H, tt, *J* = 7.3, 1.0 Hz, H9), 7.25 (2H, d, *J* = 7.3 Hz, H7), 4.60 (1H, t, *J* = 5.5 Hz, H5), 2.89 (3H, s, H10), 2.66-2.51 (2H, m, H2), 2.32-2.21 (1H, m, H4), 1.96-1.68 (3H, m, H3, H4).

^{13}C NMR (101 MHz, Chloroform-*d*): δ 171.0 (C1), 131.5 (C6), 128.8 (C8), 127.6 (C9), 126.5 (C7), 63.7 (C5), 34.0 (C10), 32.3 (C2), 32.3 (C4), 17.6 (C3).

HRMS (ESI$^+$) *m/z* calculated for $C_{12}H_{16}NO$ [M+H]$^+$ 190.1226, found 190.1228.

MP: 68-69 °C.

IR: ν_{max} = 2980 (C-H), 1626 (C=O), 1601 (C=C), 1392, 1155, 1074.

3-(But-2-en-1-ylidene)-1-methyl-6-phenylpiperidin-2-one (14a)

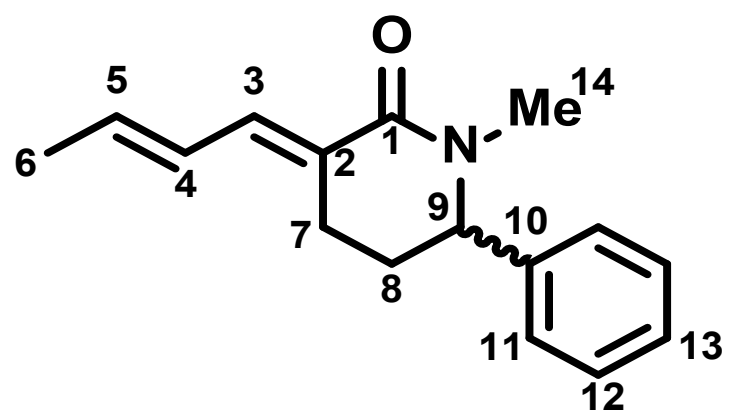

Various isomers of the conjugated lactam were synthesised according to General Procedure 4 and 5 using 1-methyl-6-phenylpiperidin-2-one (1.00 g, 5.29 mmol, 1.0 eq.) as the lactam and crotonaldehyde (0.53 mL, 6.35 mmol, 1.2 eq.) as the aldehyde component. Purification was carried out using flash column chromatography (15% EtOAc / petroleum ether) to separate the isomers. The main isomer is reported here, isolated as an orange oil (0.42 g, 1.75 mmol, 33%). The minor isomers could not be isolated with good purity, and their stereochemistry could not be determined and are therefore not included here.

^{1}H NMR (400 MHz, Chloroform-*d*): δ 7.41-7.22 (5H, m, H3, H12, H13), 7.13 (2H, d, *J* = 7.6 Hz, H11), 6.29-6.20 (1H, m, H4), 6.10 (1H, dq, *J* = 15.1, 6.5 Hz, H5), 4.59 (1H, t, *J* = 4.7 Hz, H9), 3.13 (3H, s, H14), 2.59-2.50 (1H, m, H7), 2.35-2.26 (1H, m, H7), 2.26-2.19 (1H, m, H8), 1.95-1.88 (1H, m, H8), 1.83 (3H, d, *J* = 6.5 Hz, H6).

^{13}C NMR (101 MHz, Chloroform-*d*): δ 166.4 (C1), 140.7 (C10), 137.3 (C5), 135.1 (C3), 128.8 (C11), 127.6 (C13), 126.9 (C4), 126.5 (C12), 125.8 (C2), 63.2 (C9), 31.7 (C14), 30.4 (C8), 20.5 (C7), 19.0 (C6).

HRMS (ESI^{+}) *m/z* calculated for $C_{16}H_{20}NO$ [M+H]$^{+}$ 242.1539, found 242.1537.

IR: ν_{max} = 2989 (C-H), 1648 (C=O), 1616 (C=C), 1393, 1311, 1170.

NOESY for this isomer:

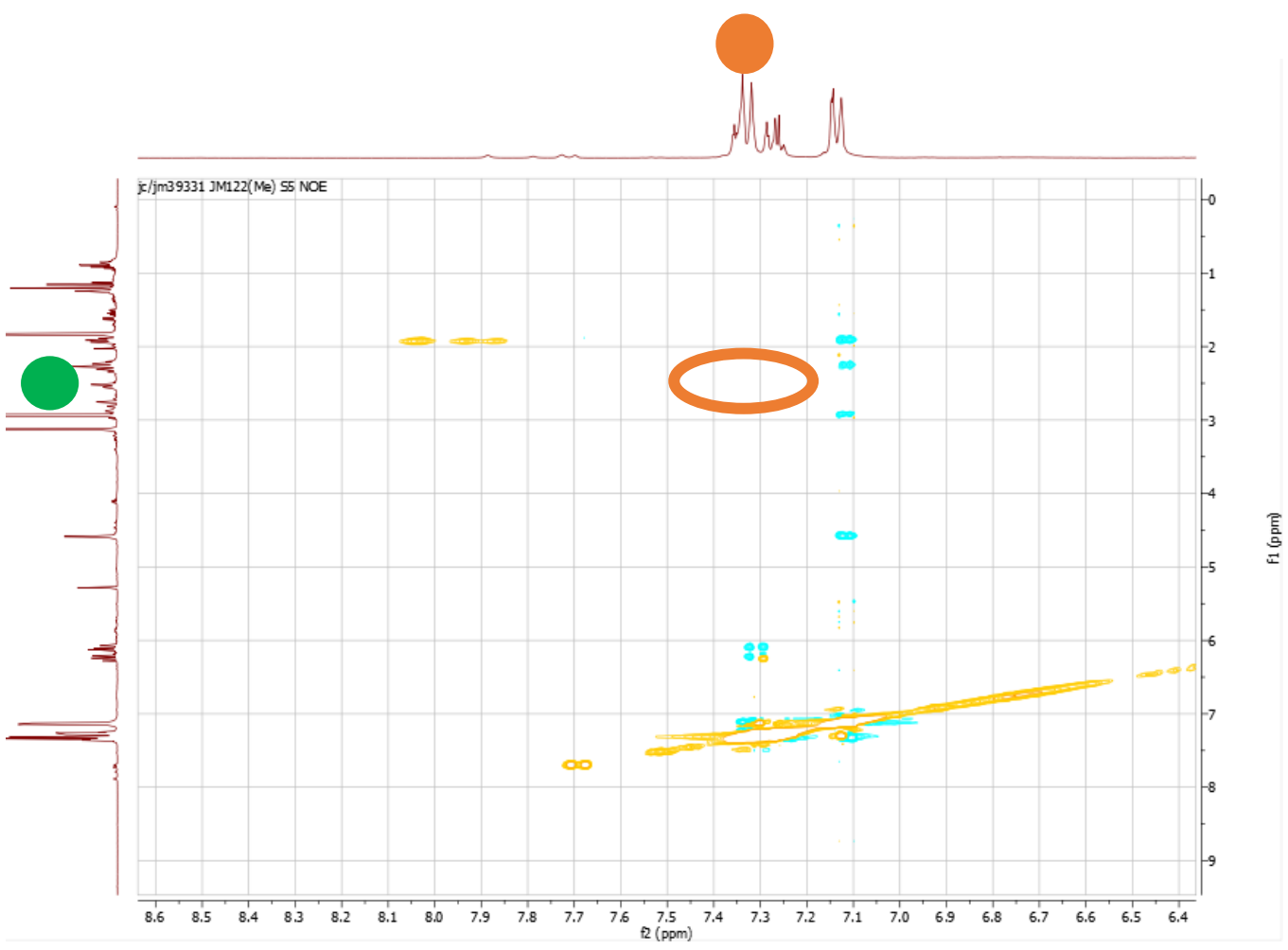

This isomer structure was confirmed by the lack of correlation between H3 and H7.

(*E*)-1-(Buta-1,3-dien-1-yl)-*N*-methyl-2-phenylcyclobutane-1-carboxamide (333) and *N*-Methyl-6-vinyl-1,2,3,6-tetrahydro-[1,1'-biphenyl]-4-carboxamide (15a and 16a)

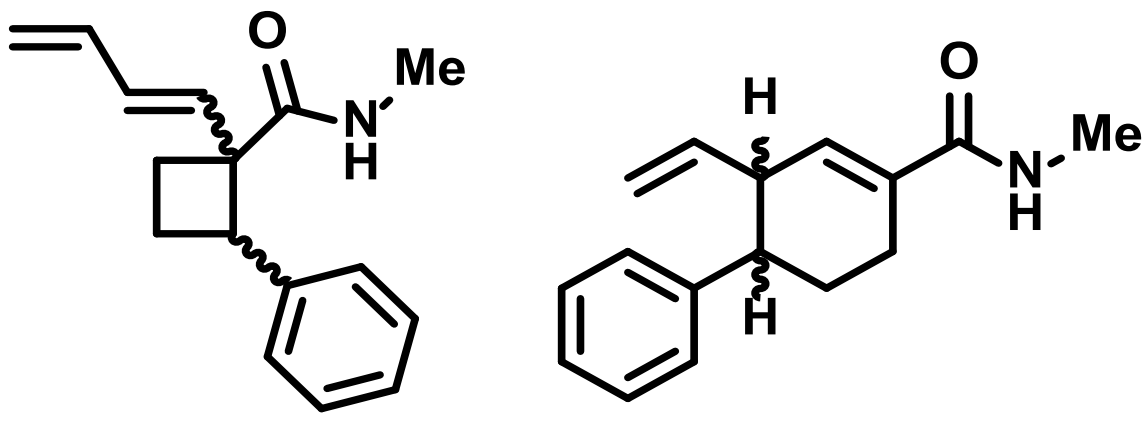

The title compound was synthesised according to General procedure 6 using 3-(but-2-en-1-ylidene)-1-methyl-6-phenylpiperidin-2-one (**14a**) (100 mg, 0.41 mmol, 1.0 eq.). Purification with flash column chromatography (20-50% EtOAc / petroleum ether) afforded the photochemical products.

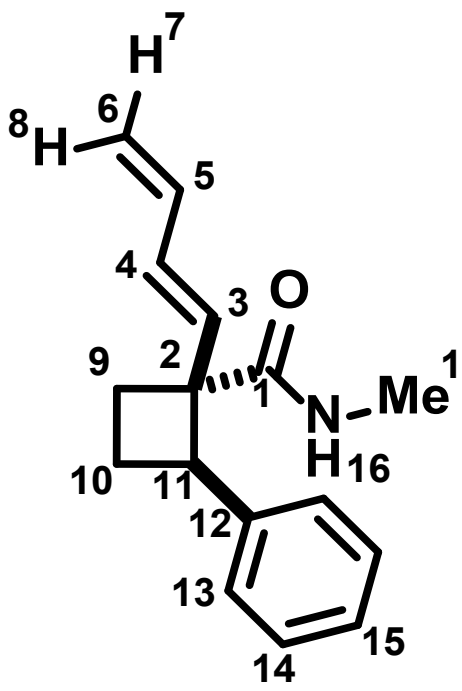

Ring-Contraction Isomers 15a: Colourless oil (16 mg, 0.06 mmol, 16%). Only the major diastereoisomers were isolated clean from purification and hence are the only diastereomers reported here. The reaction afforded a crude dr of 80:20.

^{1}H NMR (400 MHz, Chloroform-*d*): δ 7.36-7.31 (2H, m, H14), 7.25-7.20 (3H, m, H13, H15), 6.33-6.22 (2H, m, H4, H5), 5.46 (1H, br s, H16), 5.43 (1H, d, *J* = 14.5 Hz, H3), 5.24 (1H, dd, *J* = 16.5, 1.0 Hz, H8), 5.11 (1H, dd, *J* = 10.7, 1.0 Hz, H7), 4.39 (1H, dd, *J* = 10.2, 7.8 Hz, H11), 2.86 (3H, d, *J* = 4.8 Hz, H17), 2.56-2.48 (1H, m, H9), 2.36-2.28 (1H, m, H10), 2.27-2.20 (1H, m, H9), 2.19-2.11 (1H, m, H10).

^{13}C NMR (101 MHz, Chloroform-*d*): δ 175.1 (C1), 139.5 (C12), 136.6 (C4), 133.1 (C5), 132.3 (C3), 128.4 (C13), 128.2 (C14), 126.6 (C15), 117.6 (C6), 55.8 (C2), 46.3 (C11), 27.1 (C9), 26.8 (C17), 21.3 (C10).

HRMS (ESI$^+$) *m/z* calculated for $C_{16}H_{20}NO$ $[M+H]^+$ 242.1539, found 242.1531.

IR: ν_{max} = 3187 (N-H), 2921 (C-H), 1647 (C=O), 1601 (C=C), 1462, 1367, 1011.

NOESY for major diastereomers of 15a:

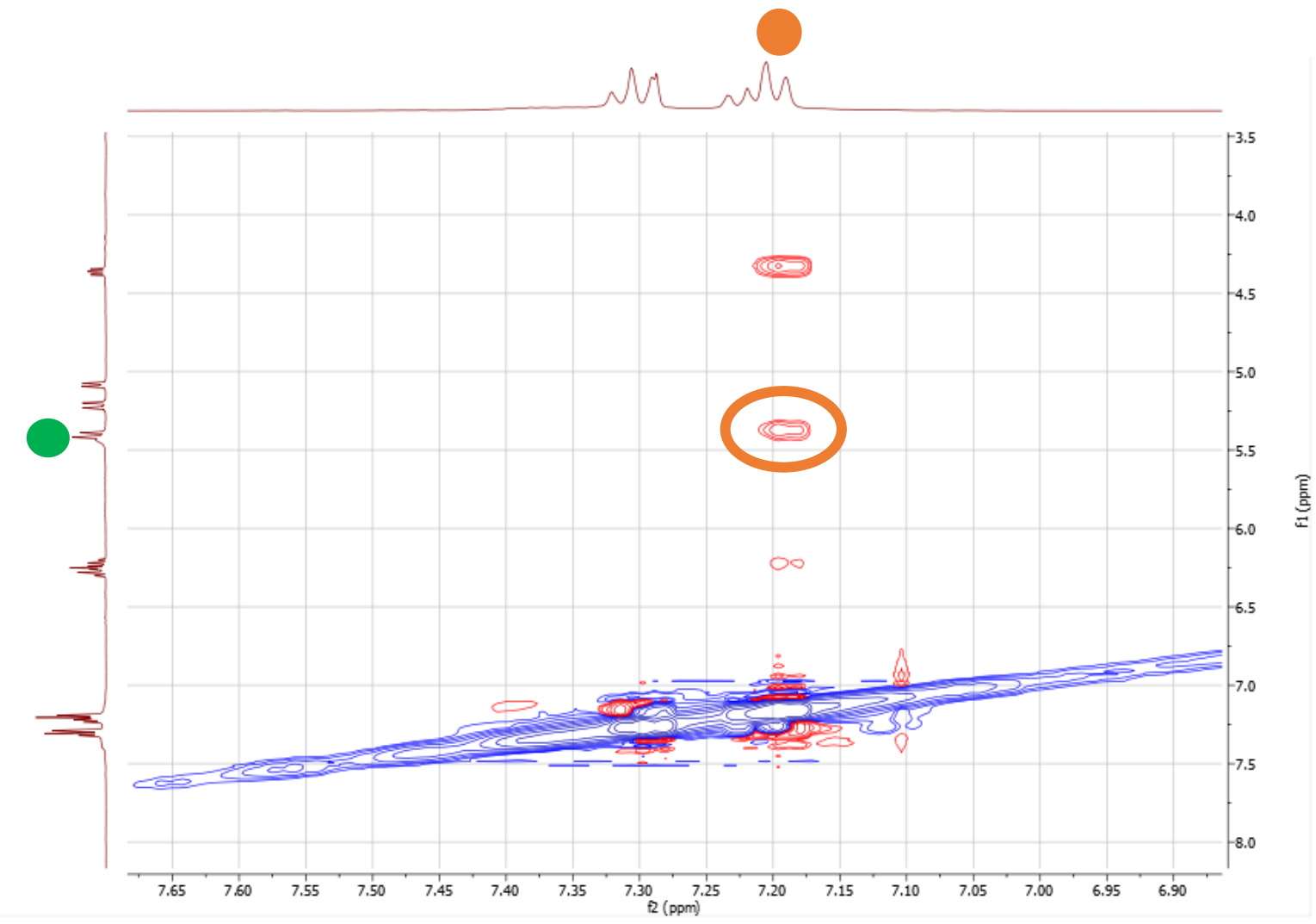

Note that for these diastereomers, the aromatic protons H13 sees the adjacent alkenyl proton H3, indicating a *cis*-relationship between the two groups.

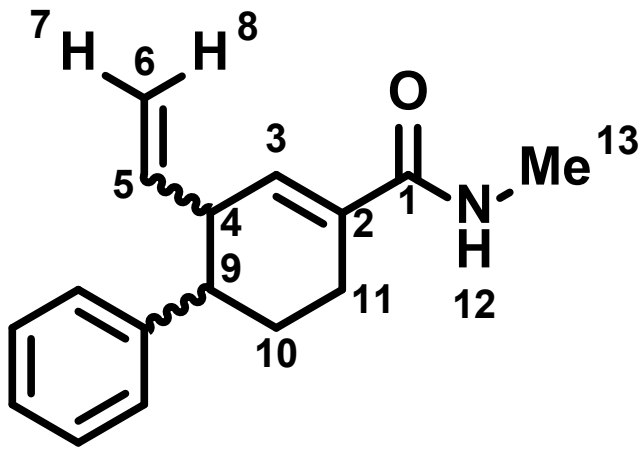

"**Ring-Swing**" **Isomers 16a**: Yellow oil (17 mg, 0.07 mmol, 17%) with a crude dr of 66:33 for D1:D2 where D1 and D2 refer to the diastereomer of product.

^{1}**H NMR** (400 MHz, Chloroform-*d*): δ 7.37-7.15 (10H, m, Ar), 6.63 (1H, br d, *J* = 5.2 Hz, H3 D1), 6.59-6.55 (1H, m, H3 D2), 5.83 (2H, br s, H12 D1, H12 D2), 5.68 (1H, ddd, *J* = 17.1, 10.2, 7.6 Hz, H5 D2), 5.45 (1H, ddd, *J* = 17.3, 10.2, 7.1 Hz, H5 D1), 5.05 (1H, d, *J* = 10.2 Hz, H7 D1), 4.99 (1H, dd, *J* = 10.2, 0.9 Hz, H7 D2), 4.93 (1H, dd, *J* = 17.1, 0.9 Hz, H8 D2), 4.88 (1H, d, *J* = 17.3 Hz, H8 D1), 3.30-3.23 (1H, m, H4 D1), 3.17-3.05 (2H, m, H4 D2, H9 D1), 2.95 (3H, d, *J* = 4.9 Hz, H13 D1), 2.94 (3H, d, *J* = 4.9 Hz, H13 D2), 2.63-2.53 (1H, m, H9 D2), 2.51-2.33 (4H, m, H11 D1, H11 D2), 2.09-1.84 (4H, m, H10 D1, H10 D2).

^{13}**C NMR** (101 MHz, Chloroform-*d*): δ 169.0 (C1 D1), 169.0 (C1 D2), 144.7 (Ar), 143.3 (Ar), 139.6 (C5 D2), 136.4 (C5 D1), 134.9 (C3 D2), 134.3 (C3 D1), 134.0 (C2 D1), 133.9 (C2 D2), 128.5 (Ar), 128.3 (Ar), 127.9 (Ar), 127.6 (Ar), 126.5 (Ar), 126.4 (Ar), 118.0 (C6 D1), 116.2 (C6 D2), 46.5 (C4 D2), 46.0 (C9 D2), 44.7 (C4 D1), 42.6 (C9 D1), 30.0 (C10 D2), 26.6 (C13 D2), 25.6 (C13 D1), 24.9 (C11 D2), 24.0 (C11 D1), 22.5 (C10 D1).

HRMS (ESI$^+$) *m/z* calculated for $C_{16}H_{20}NO$ $[M+H]^+$ 242.1539, found 242.1530.

IR: ν_{max} = 3330 (N-H), 2982 (C-H), 1658 (C=O), 1614 (C=C), 1393, 1154, 966.

6-(Phenylsulfonyl)piperidin-2-one

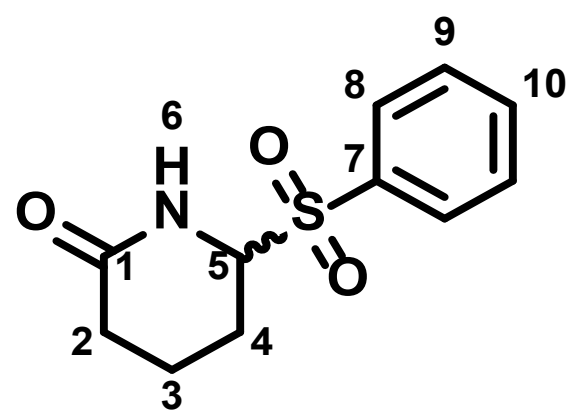

Synthesised according to a procedure by Martin *et. al.*[19]

Glutarimide (53.07 mmol, 1.0 eq.) was slurried in EtOH (0.2 M) before addition of $NaBH_4$ (0.13 mol, 2.5 eq.) at 0 °C. Using a syringe pump (1.41 mL/hr), a solution of 2 M HCl in EtOH was added over 4 hours at this temperature. Ethanolic HCl (90 mL, 2 M) was added cautiously at 0 °C before the mixture was allowed to stir at room temperature for 16 hours. 5 M $NaOH_{(aq.)}$ was added until the pH reached ~8/9 and the mixture was filtered before the filtrate was concentrated *in vacuo* to remove the ethanol. The aqueous layer was extracted with CH_2Cl_2 (3 × 40 mL) and then to this crude solution was added $PhSO_2Na$ (0.11 mol, 2.0 eq.) and formic acid (0.11 mol, 2.2 eq., 88%). The mixture was left to stir for 16 hours before addition of brine (100 mL) and the aqueous layer extracted with CH_2Cl_2 (3 × 50 mL). The combined organics were washed with brine (2 × 60 mL), dried with $MgSO_4$, filtered and concentrated *in vacuo* to give the title compound as a white solid (39.27 mmol, 74%).

^{1}H NMR (400 MHz, Chloroform-*d*): δ 7.90 (2H, d, *J* = 7.2 Hz, H8), 7.74-7.68 (1H, m, H10), 7.60 (2H, t, *J* = 7.2 Hz, H9), 6.91 (1H, br s, H6), 4.53 (1H, dt, *J* = 7.9, 6.1 Hz, H5), 2.29 (1H, t, *J* = 7.1 Hz, H2), 2.22 (1H, t, *J* = 7.1 Hz, H2), 2.18-2.08 (2H, m, H4), 1.99-1.87 (1H, m, H3), 1.74-1.62 (1H, m, H3).

^{13}C NMR (101 MHz, Chloroform-*d*): δ 173.0 (C1), 135.0 (C7), 134.9 (C10), 129.8 (C9), 129.7 (C8), 72.2 (C5), 30.8 (C2), 21.9 (C4), 17.5 (C3).

Data consistent with literature.[19]

6-Vinylpiperidin-2-one

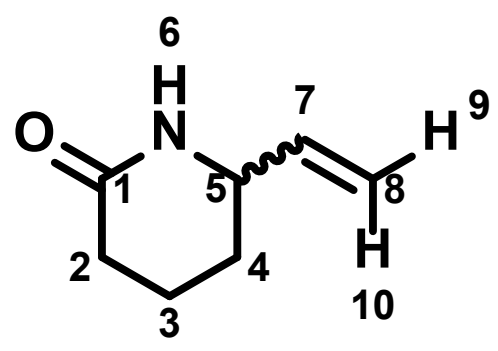

Synthesised according to a procedure by Martin *et. al*.[19]

To a slurry of 6-(phenylsulfonyl)piperidin-2-one (29.28 mmol, 1.0 eq.) in anhydrous THF (50 mL) was added vinyl magnesium bromide (87.84 mmol, 3.0 eq., 1 M in THF) *via* cannula transfer at −78 °C. After 15 minutes at this temperature, the mixture was placed in a NaCl/ice/water bath for 20 minutes, with temperature kept below −10 °C. The ice bath was removed, and the mixture stirred at room temperature for 30 minutes. $NaHCO_{3(aq.)}$ (50 mL) added and the mixture left to stir for 30 minutes. The mixture was filtered under vacuum, washing with $CHCl_3$ before the filtrate was concentrated *in vacuo* to remove solvent. The aqueous layer was extracted with $CHCl_3$ (3 × 30 mL) before combined organics washed with water (30 mL), brine (30 mL), dried with $MgSO_4$, filtered and concentrated *in vacuo*. Purification by flash column chromatography (0-1% MeOH / CH_2Cl_2) afforded the title compound as a yellow crystalline solid (11.42 mmol, 39%).

^{1}H NMR (400 MHz, Chloroform-*d*): δ 6.60 (1H, br s, H6), 5.73 (1H, ddd, *J* = 17.2, 10.3, 6.2 Hz, H7), 5.17 (1H, dt, *J* = 17.2, 1.1 Hz, H10), 5.10 (1H, dt, *J* = 10.3, 1.1 Hz, H9), 3.94-3.86 (1H, m, H5), 2.35-2.19 (2H, m, H2), 1.92-1.83 (1H, m, H4), 1.82-1.75 (1H, m, H3), 1.71-1.58 (1H, m, H3), 1.52-1.42 (1H, m, H4).

^{13}C NMR (101 MHz, Chloroform-*d*): δ 172.5 (C1), 139.0 (C7), 116.5 (C8), 55.3 (C5), 31.7 (C2), 28.5 (C4), 18.9 (C3).

Data consistent with literature.[19]

1-Methyl-6-vinylpiperidin-2-one

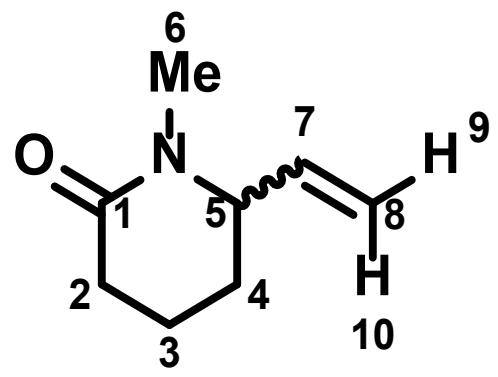

A solution of 6-vinylpiperidin-2-one (11.98 mmol, 1.0 eq.) in anhydrous THF (20 mL) was cooled to −78 °C before addition of ^{n}BuLi (14.38 mmol, 1.2 eq., 2.5 M in hexanes) at this temperature to give a thick orange slurry. After 30 minutes, MeI (23.96 mmol, 2.0 eq.) was added slowly before the mixture was allowed to warm to room temperature. After stirring at room temperature for 3.5 hours, $NH_4Cl_{(aq.)}$ (30 mL) and H_2O (30 mL) were added, and the layers separated. The aqueous layer was extracted with EtOAc (3 × 30 mL) before the organics were washed with brine (30 mL), dried with Na_2SO_4, filtered and concentrated *in vacuo* to give an orange liquid (10.42 mmol, 87%) which was used without further purification.

^{1}H NMR (400 MHz, Chloroform-*d*): δ 5.62 (1H, ddd, *J* = 17.2, 10.9, 6.3 Hz, H7), 5.11 (1H, d, *J* = 10.2 Hz, H9), 5.01 (1H, d, *J* = 17.2 Hz, H10), 3.76 (1H, q, *J* = 5.5 Hz, H5), 2.76 (3H, s, H6), 2.33-2.18 (2H, m, H2), 1.88-1.78 (1H, m, H4), 1.76-1.66 (1H, m, H3), 1.65-1.52 (2H, m, H3, H4).

^{13}C NMR (101 MHz, Chloroform-*d*): δ 170.5 (C1), 137.7 (C7), 116.8 (C8), 61.8 (C5), 33.6 (C6), 32.2 (C2), 28.8 (C4), 17.7 (C3).

HRMS (ESI$^+$) *m/z* calcd for $C_8H_{14}NO$ $[M+H]^+$ 140.1070, found 140.1066.

IR: ν_{max} = 2975 (C-H), 1620 (C=O & C=C broad), 1396, 1243, 1044.

(*E*)-3-((*E*)-but-2-en-1-ylidene)-1-methyl-6-vinylpiperidin-2-one (14b)

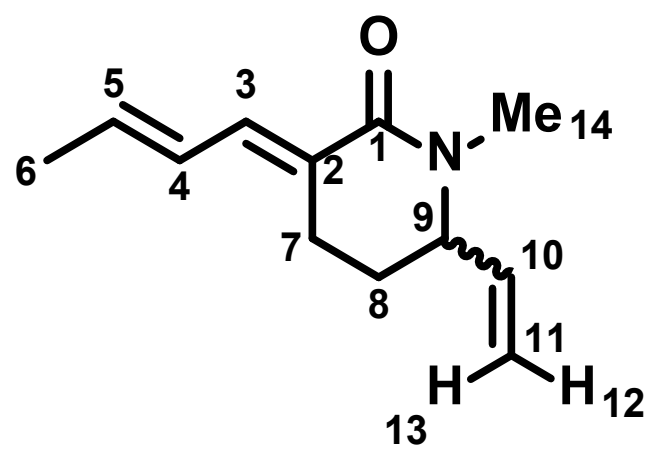

Various isomers of the conjugated lactam were synthesised according to General Procedure 4 and 5 using 1-methyl-6-vinylpiperidin-2-one (8.63 mmol) as the lactam and crotonaldehyde as the aldehyde component. Purification was carried out using flash column chromatography (0-20% EtOAc / petroleum ether) to separate the isomers. The main isomer is reported here, isolated as a yellow liquid (0.95 mmol, 11%). The minor isomers could not be isolated with good purity, and their stereochemistry could not be determined and hence they are not included here. No NOE was observed between H3 and H7, indicating this stereoisomer is present.

^{1}H NMR (400 MHz, Chloroform-*d*): δ 7.13 (1H, d, *J* = 11.6 Hz, H3), 6.17 (1H, ddd, *J* = 16.1, 11.6, 1.2 Hz, H4), 6.01-5.90 (1H, m, H5), 5.65 (1H, ddd, *J* = 17.4, 10.3, 5.9 Hz, H10), 5.11 (1H, dd, *J* = 10.3, 1.4 Hz, H12), 4.99 (1H, dd, *J* = 17.4, 1.4 Hz, H13), 3.84 (1H, br q, *J* = 3.9 Hz, H9), 2.87 (3H, s, H14), 2.50 (1H, dt, *J* = 16.3, 4.2 Hz, H7), 2.42-2.29 (1H, m, H7), 1.95-1.83 (1H, m, H8), 1.74 (3H, d, *J* = 6.4 Hz, H6), 1.73-1.67 (1H, m, H8).

^{13}C NMR (101 MHz, Chloroform-*d*): δ 165.5 (C1), 136.6 (C5), 136.3 (C10), 134.4 (C3), 126.7 (C4), 125.6 (C2), 116.4 (C11), 61.0 (C9), 34.3 (C14), 27.0 (C8), 20.6 (C7), 18.7 (C14).

HRMS (ESI$^+$) *m/z* calcd for $C_{12}H_{18}NO$ [M+H]$^+$ 192.1383, found 192.1383.

IR: ν_{max} = 2887 (C-H), 1644 (C=O), 1606 (C=C), 1475, 1395, 1314.

1-(Buta-1,3-dien-1-yl)-*N*-methyl-2-vinylcyclobutane-1-carboxamide (15b)

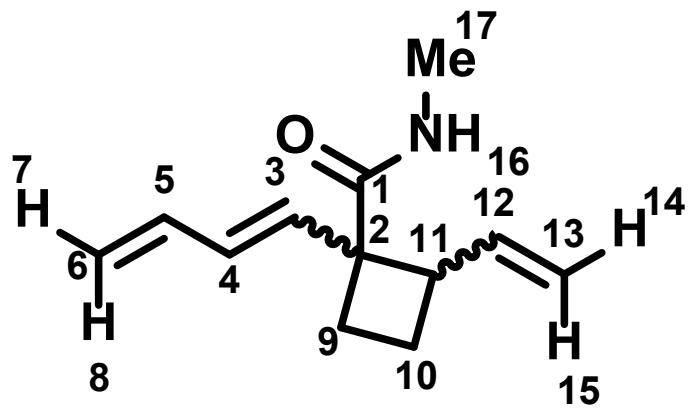

Only a single diastereomer of the title compound was isolated, synthesised according to General procedure 6 using (*E*)-3-((*E*)-but-2-en-1-ylidene)-1-methyl-6-vinylpiperidin-2-one **(14b)** (0.52 mmol). Purification with flash column chromatography (10-30% EtOAc / petroleum ether) afforded a colourless oil (0.01 mmol, 2%). Not enough material was isolated for a clear ^{13}C NMR spectra or IR spectra and hence it is not reported here.

^{1}H NMR (400 MHz, Chloroform-*d*): δ 6.36 (1H, dt, *J* = 16.8, 10.0 Hz, H5), 6.23-6.16 (1H, m, H4), 5.93-5.78 (2H, m, H3, H12), 5.48 (1H, br s, H16), 5.25 (1H, dd, *J* = 16.7, 1.3 Hz, H8), 5.13 (1H, dd, *J* = 9.9, 1.3 Hz, H7), 5.08 (1H, dd, *J* = 17.1, 1.1 Hz, H15), 5.02 (1H, dd, *J* = 10.3, 1.1 Hz, H14), 3.20 (1H, q, *J* = 8.9 Hz, H11), 2.78 (3H, d, *J* = 4.9 Hz, H17), 2.65-2.57 (1H, m, H9), 2.21-2.10 (1H, m, H10), 2.07-1.96 (1H, m, H10), 1.93-1.85 (1H, m, H9).

HRMS (ESI$^+$) *m/z* calcd for $C_{12}H_{18}NO$ [M+H]$^+$ 192.1383, found 192.1383.

N-methyl-3,4-divinylcyclohex-1-ene-1-carboxamide (16b)

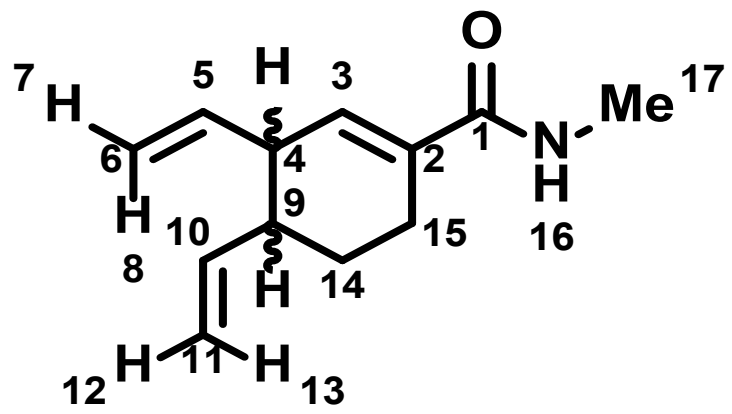

A diastereomeric mix (1.03:1) of the title compound was synthesised according to General procedure 3d using (*E*)-3-((*E*)-but-2-en-1-ylidene)-1-methyl-6-vinylpiperidin-2-one **(14b)** (0.52 mmol). Purification with flash column chromatography (10-30% EtOAc / petroleum ether) afforded a colourless oil (0.20 mmol, 39%). D1 and D2 relate to the diastereomer of product. The NMR signals for the diastereomers overlap significantly, hence appearing as complex multiplets.

^{1}H NMR (400 MHz, Chloroform-*d*): δ 6.47-6.42 (1H, m, H3 D1), 6.40-6.35 (1H, m, H3 D2), 5.93 (2H, br s, H16 D1, D2), 5.81-5.69 (3H, m, H5 D1/D2, H10 D1, D2), 5.66 (1H, ddd, *J* = 17.2, 10.0, 7.6 Hz, H5 D1/D2), 5.11-4.94 (8H, m, H6, H11 D1, D2), 2.99 (1H, br q, *J* = 6.3 Hz, H4 D1), 2.84 (6H, d, *J* = 4.8 Hz, H17 D1, D2), 2.72-2.63 (1H, m, H4 D2), 2.44-2.30 (3H, m, H9 D1, H14 D1), 2.28-2.16 (2H, m, H14 D2), 2.07-1.96 (1H, m, H9 D2), 1.90-1.82 (1H, m, H15 D2), 1.76-1.67 (1H, m, H15 D1), 1.64-1.54 (1H, m, H15 D1), 1.54-1.43 (H15 D2).

^{13}C NMR (101 MHz, Chloroform-*d*): δ 169.0 (C1 D1/D2), 168.9 (C1 D1/D2), 141.3 (C5/C10 D1/D2), 140.3 (C5/C10 D1/D2), 139.9 (C5/C10 D1/D2), 137.1 (C5 D1/D2), 134.0 (C3 D2), 133.9 (C3 D1), 133.8 (C2 D1/D2), 117.3 (C6, C11 D1, D2), 116.1 (C6, C11 D1, D2), 114.7 (C6, C11 D1, D2), 114.6 (C6, C11 D1, D2), 45.3 (C4 D2), 43.5 (C4 D1), 42.7 (C9 D1), 41.0 (C9 D2), 27.1 (C15 D2), 26.5 (C16 D1/D2), 24.0 (C14 D1/D2), 23.9 (C14 D1/D2), 23.7 (C15 D1).

HRMS (ESI$^+$) *m/z* calcd for $C_{12}H_{18}NO$ [M+H]$^+$ 192.1383, found 192.1382.

IR: ν_{max} = 3328 (N-H), 2976 (C-H), 1656 (C=O), 1635 (C=C), 1536, 1407, 1155.

2.9. Mechanistic Experimentation (TEMPO and crossover experiments)

To determine whether the photochemical rearrangement proceeds *via* a solvent-caged radical pair or freely diffusing radicals, a crossover experiment was conducted using a mixture of the doubly differentiated amides 8a and 8e. The amides were subjected to the standard reaction conditions in General Procedure 2, undergoing deprotonation to form the corresponding trienolates followed by irradiation. Subsequent analysis of the reaction mixture by GCMS showed no intermolecular exchange of the migrating groups. The lack of cross-reactivity and the exclusive formation of intramolecular rearrangement products indicate that the photochemically generated radicals do not freely diffuse. Instead, the reaction involves the formation of short-lived, solvent-caged radical pairs that undergo rapid geminate recombination.

To further probe the intermediacy of freely diffusing radicals, a radical trapping experiment was conducted using the persistent radical TEMPO. The model substrate 8a was subjected to the standard photochemical reaction condition (General Procedure 2) in the presence of an excess (2 eq.) of TEMPO. Subsequent analysis of the crude reaction mixture revealed the formation of the standard intramolecular rearrangement products, with absolutely no TEMPO-trapped adducts observed. This absence of intermolecular trapping corroborates the findings of the crossover experiment, providing further evidence that the photochemically generated radical pair undergoes rapid geminate recombination within a solvent cage at a rate that completely outcompetes diffusion into the bulk medium.

2.10. NMR Spectra

¹H NMR

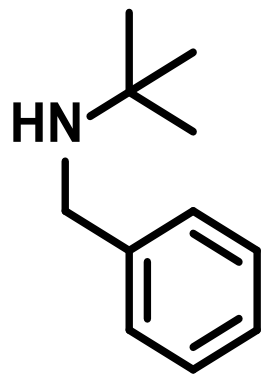

***N*-benzyl-2-methylpropan-2-amine**

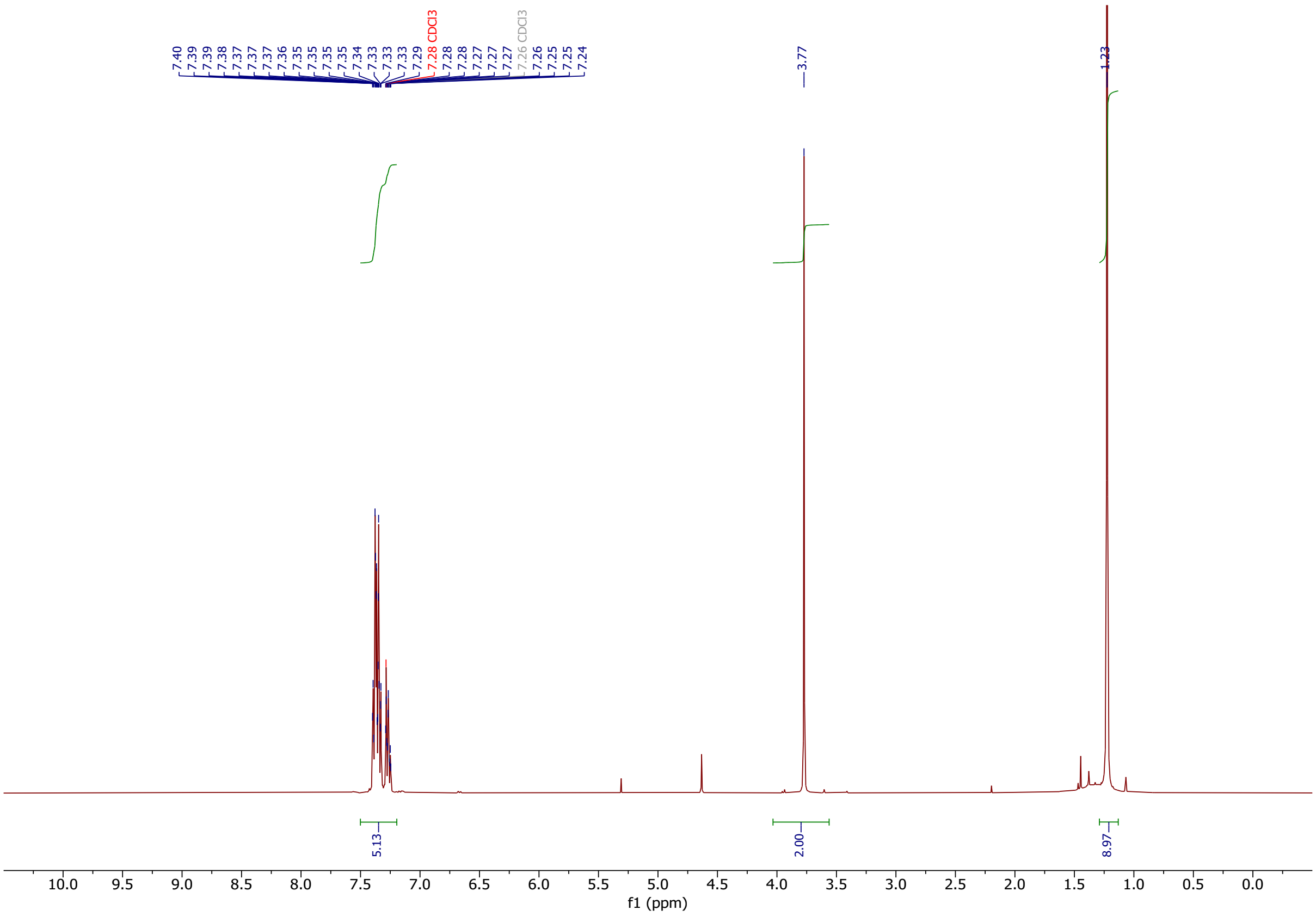

^{13}C NMR

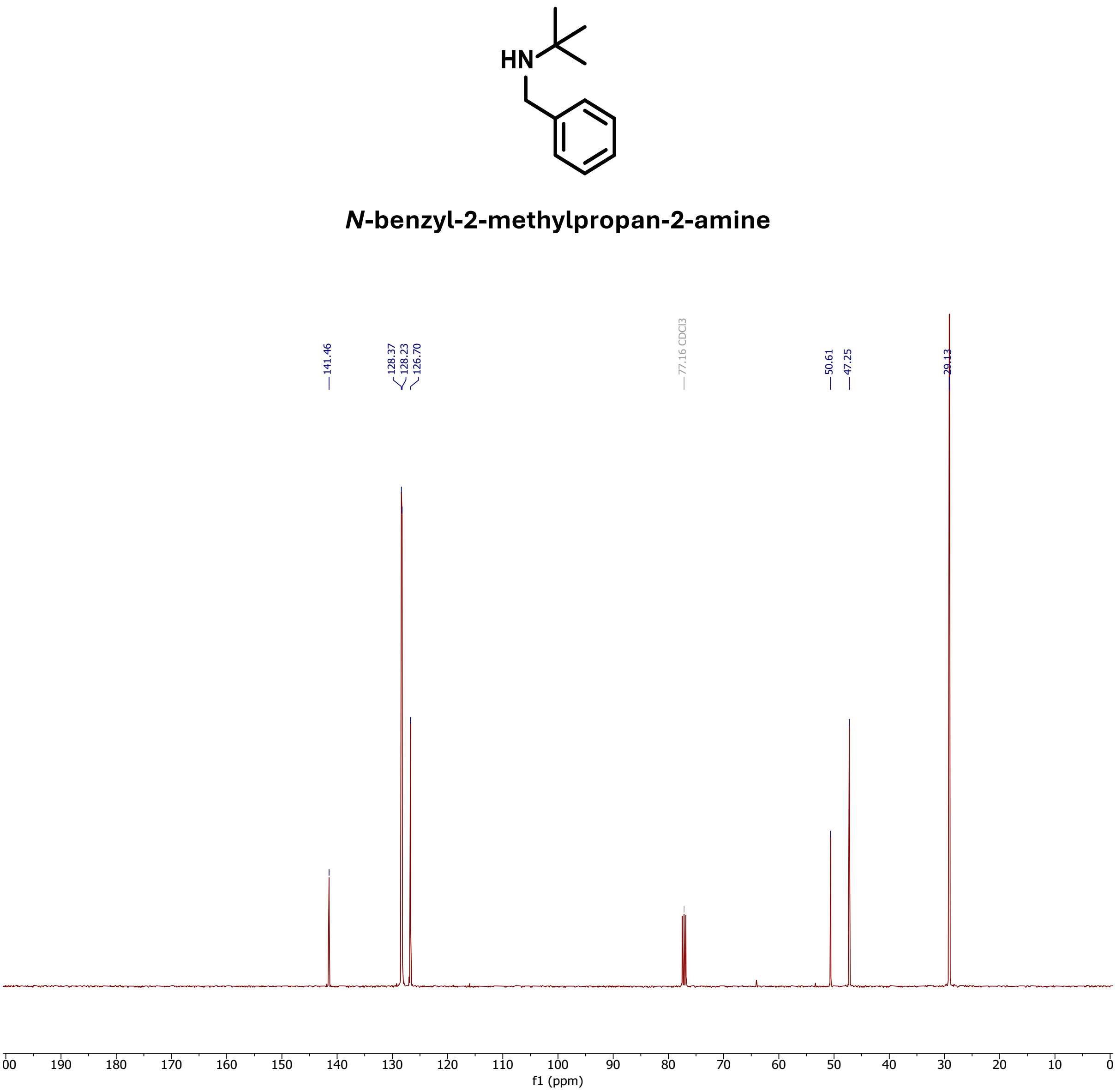
HN
N-benzyl-2-methylpropan-2-amine
141.46
128.37
128.23
126.70
77.16 CDCl3
50.61
47.25
29.13
f1 (ppm)

^{1}H NMR

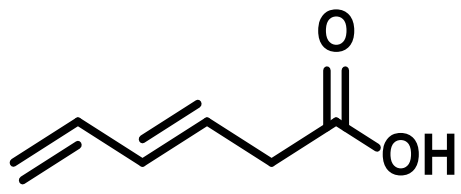

(*E*)-hexa-3,5-dienoic acid

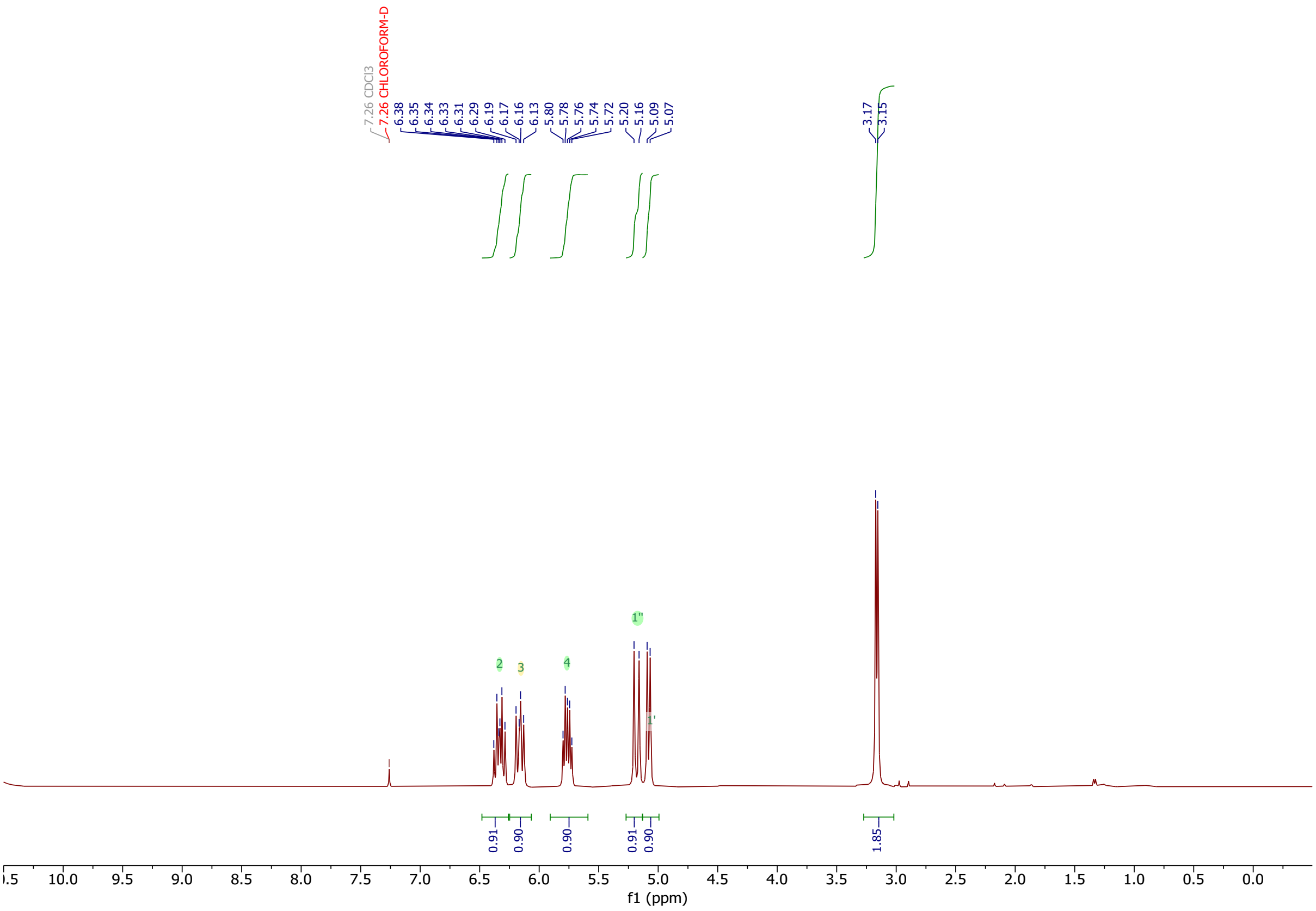

^{13}C NMR

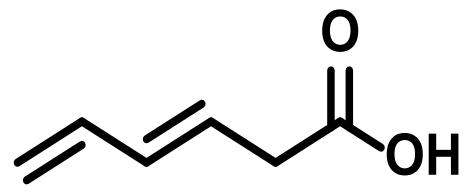

(*E*)-hexa-3,5-dienoic acid

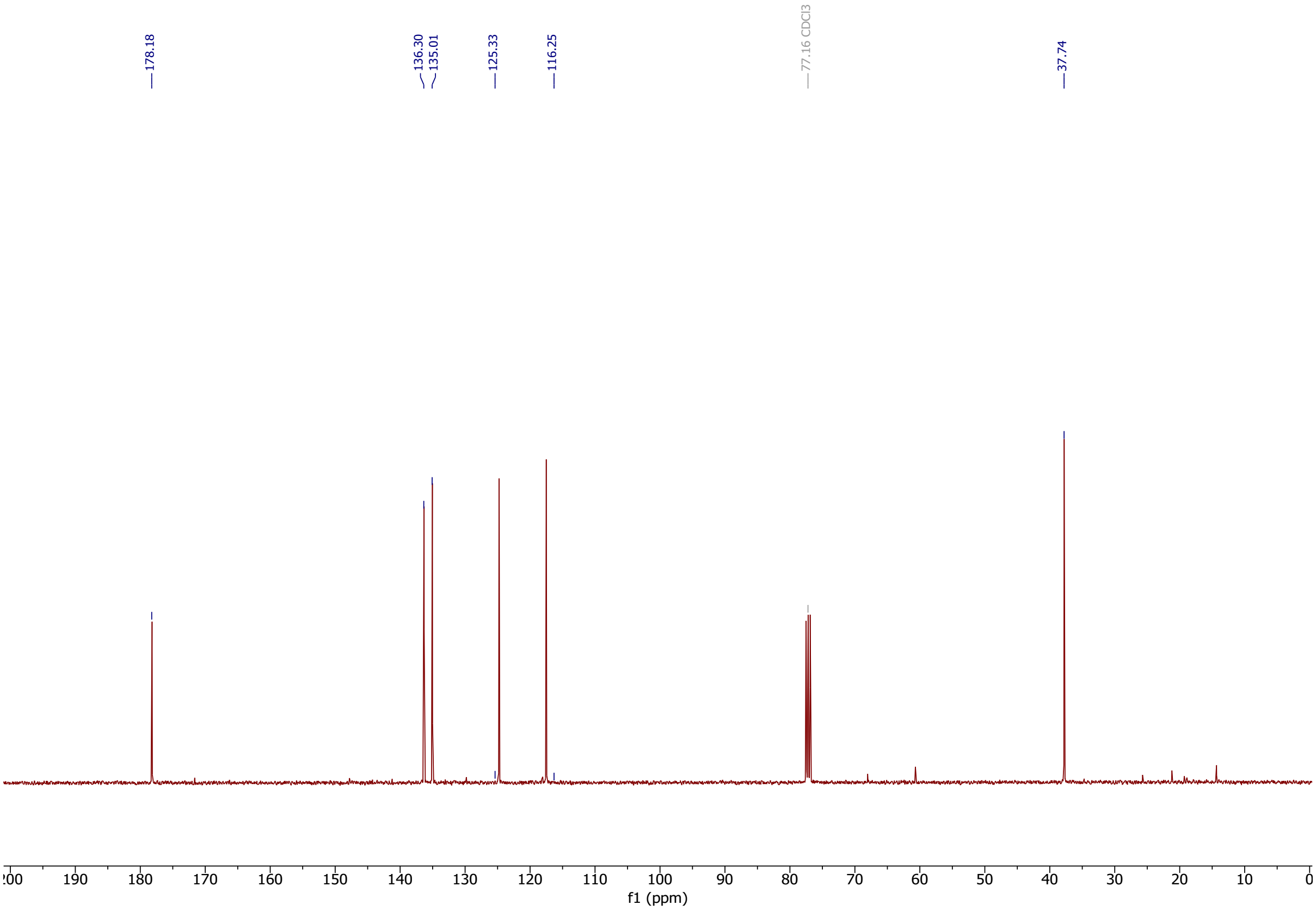

^{1}H NMR

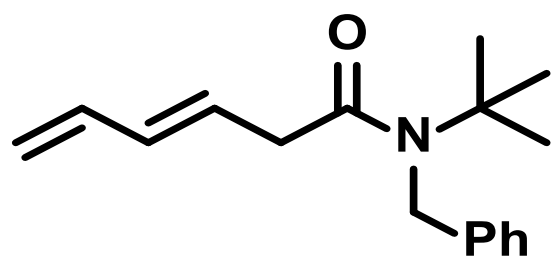

(*E*)-*N*-benzyl-*N*-(*tert*-butyl)hexa-3,5-dienamide

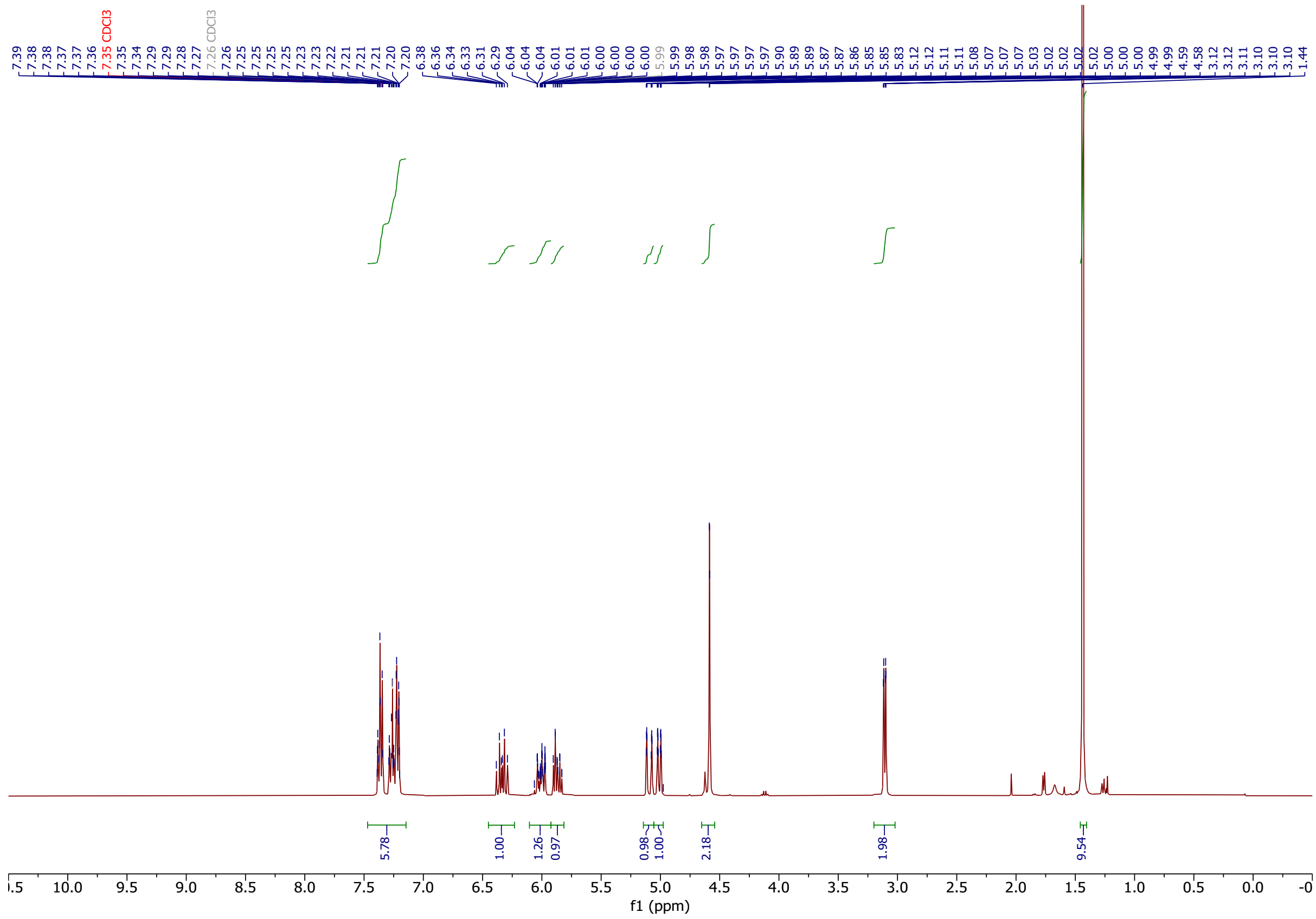

^{13}C NMR

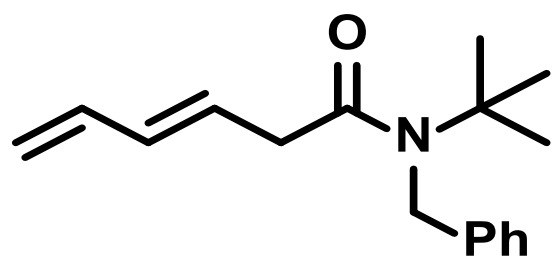

(*E*)-*N*-benzyl-*N*-(*tert*-butyl)hexa-3,5-dienamide

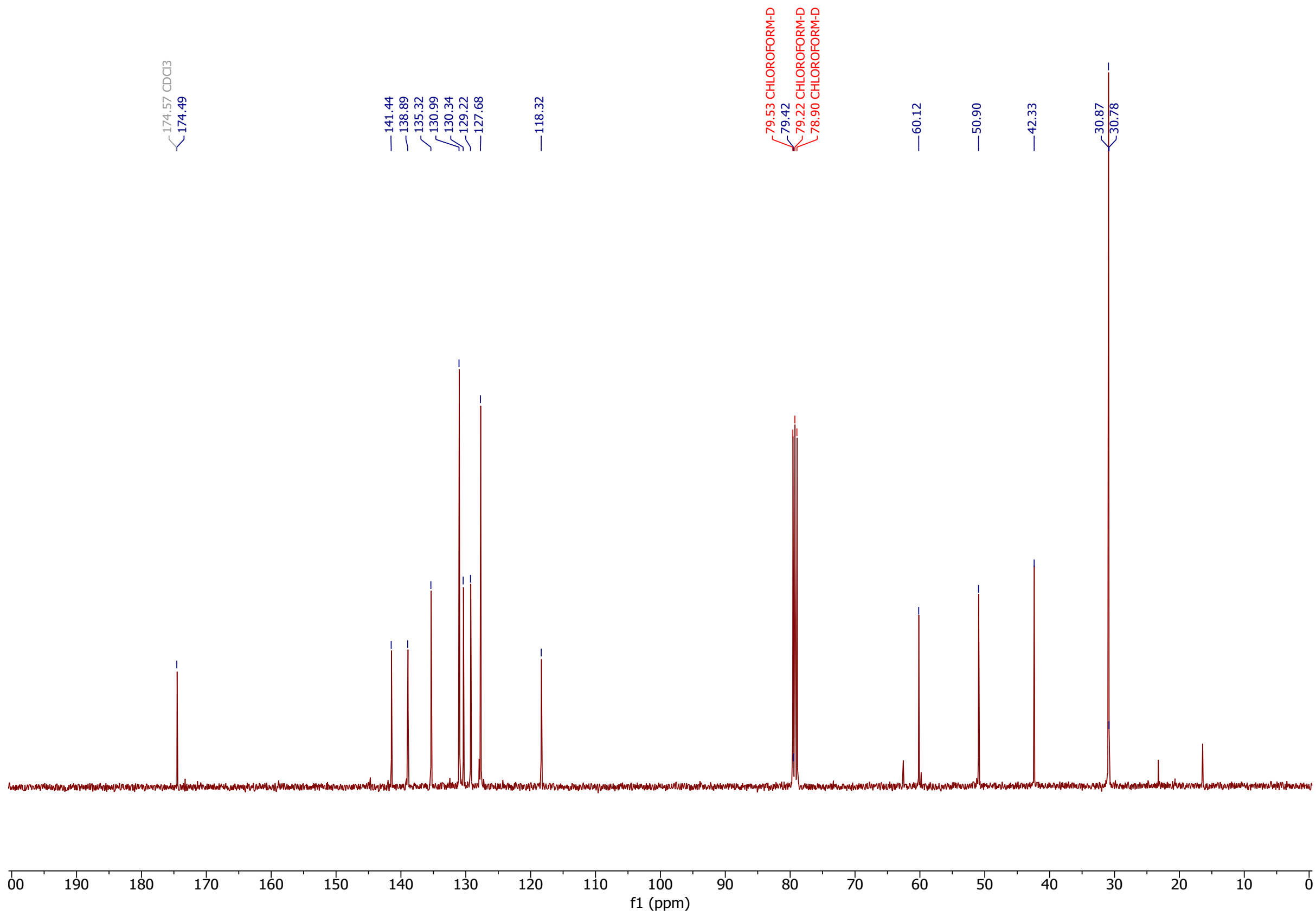

^{1}H NMR

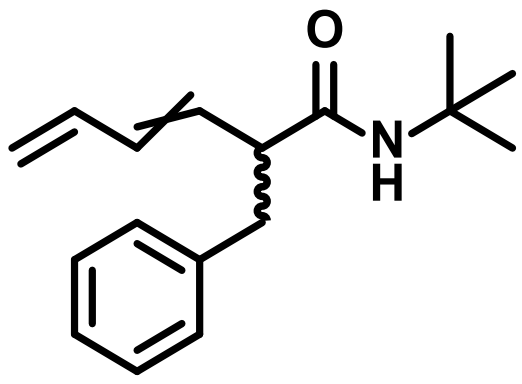

2-benzyl-*N*-(*tert*-butyl)hexa-3,5-dienamide

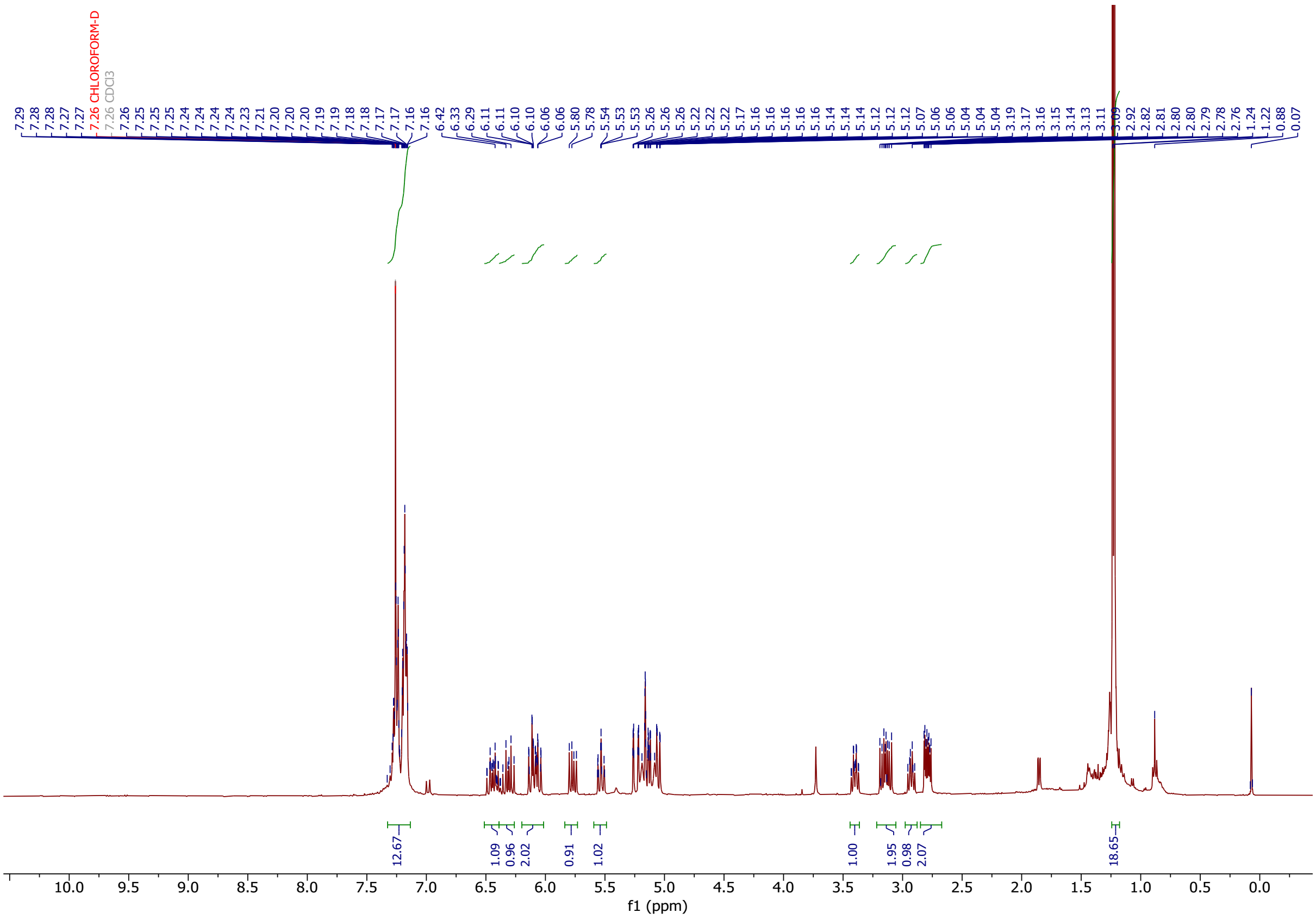

^{13}C NMR

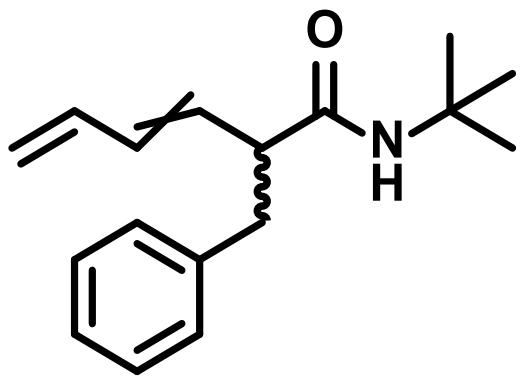

2-benzyl-*N*-(*tert*-butyl)hexa-3,5-dienamide

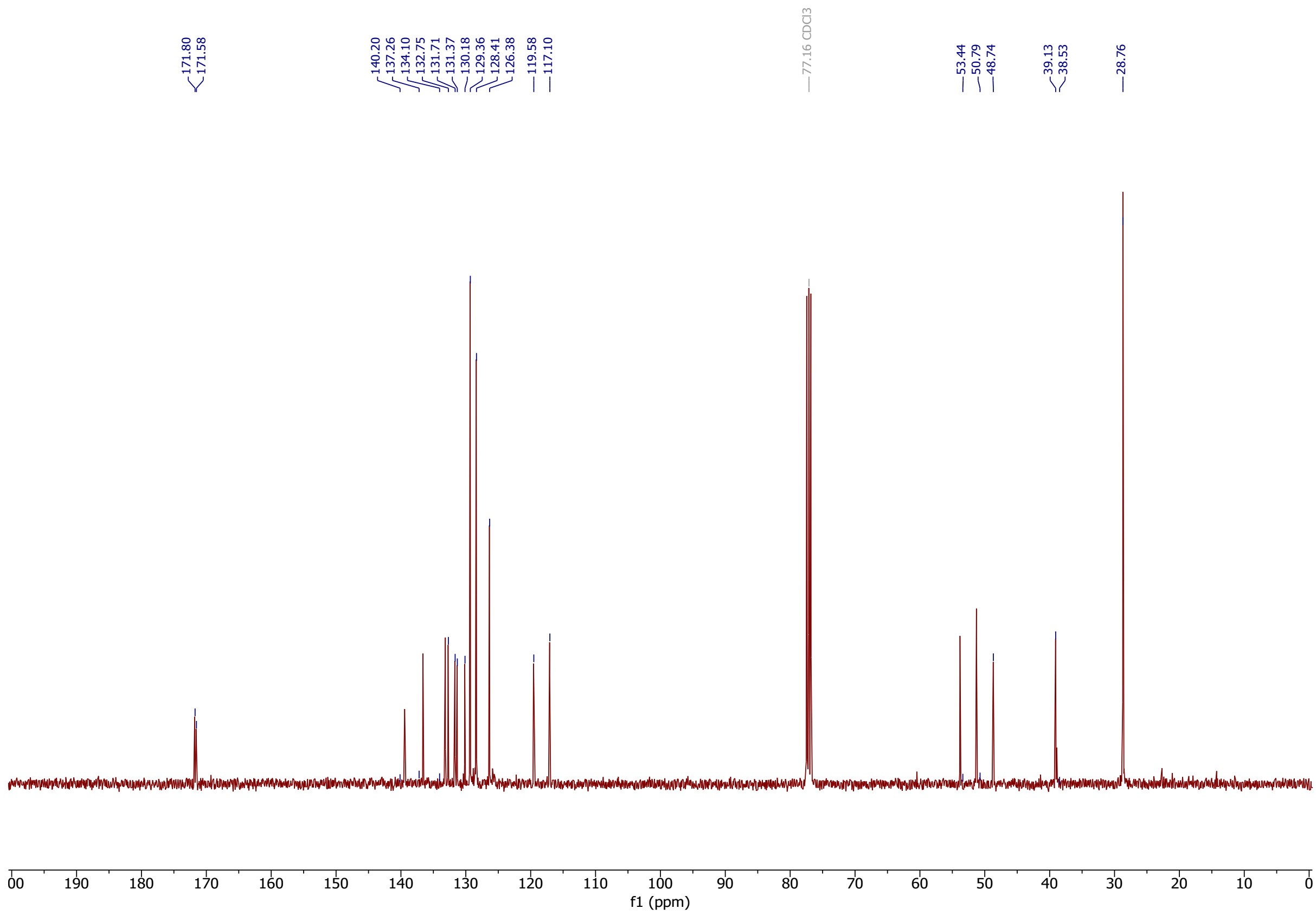

^{1}H NMR

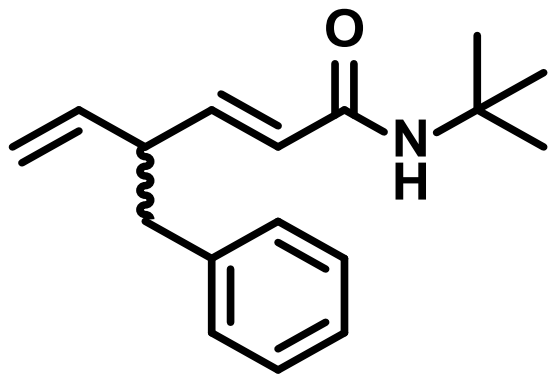

(*E*)-4-benzyl-*N*-(*tert*-butyl)hexa-2,5-dienamide

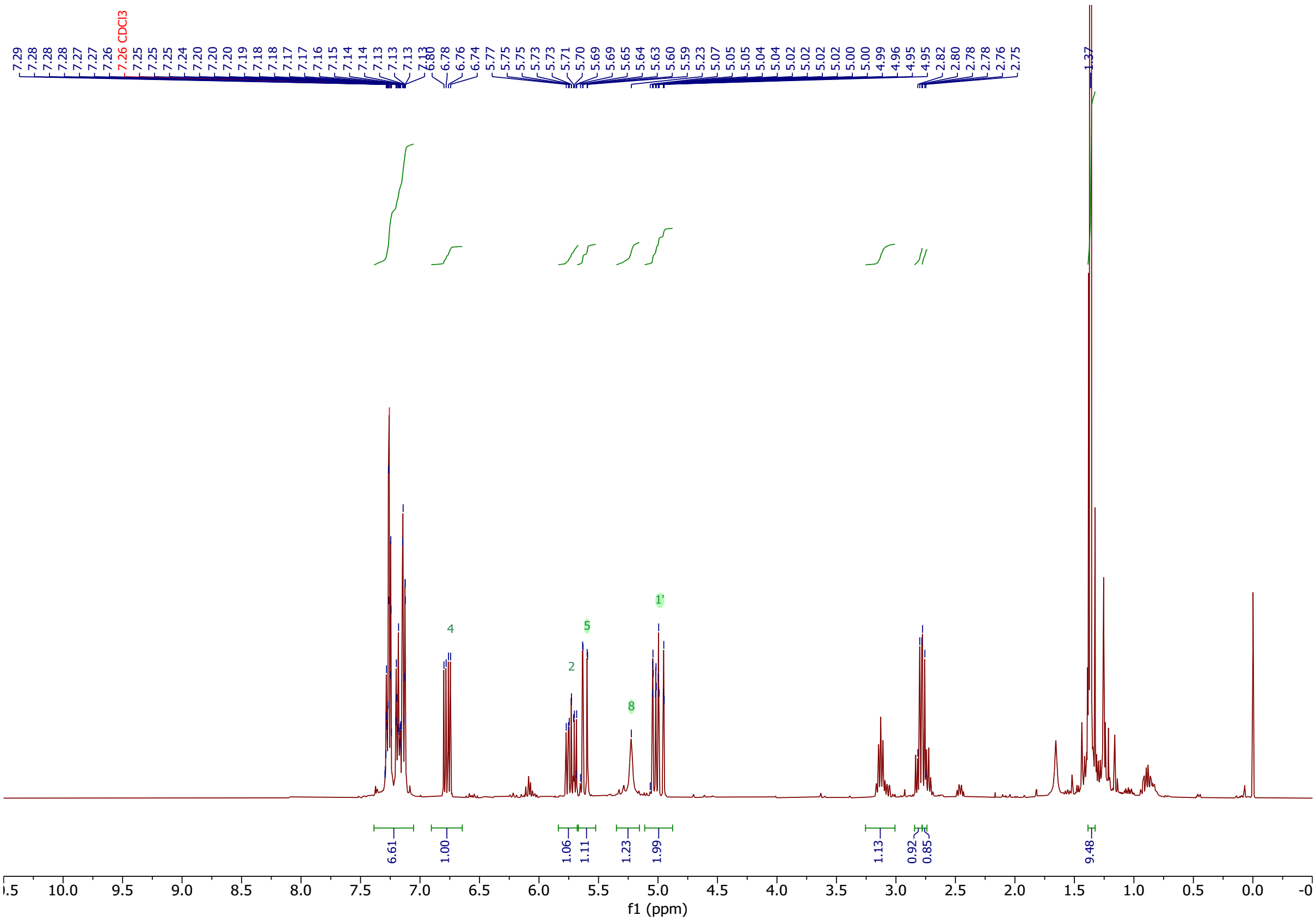

^{13}C NMR

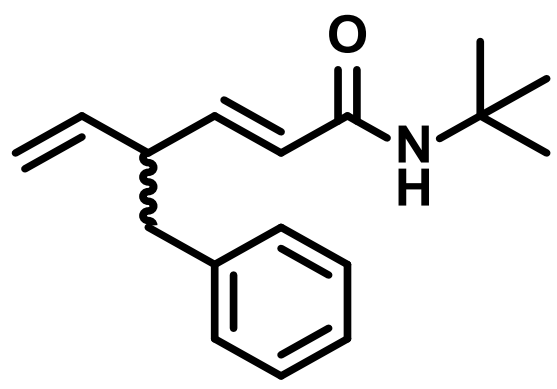

(*E*)-4-benzyl-*N*-(*tert*-butyl)hexa-2,5-dienamide

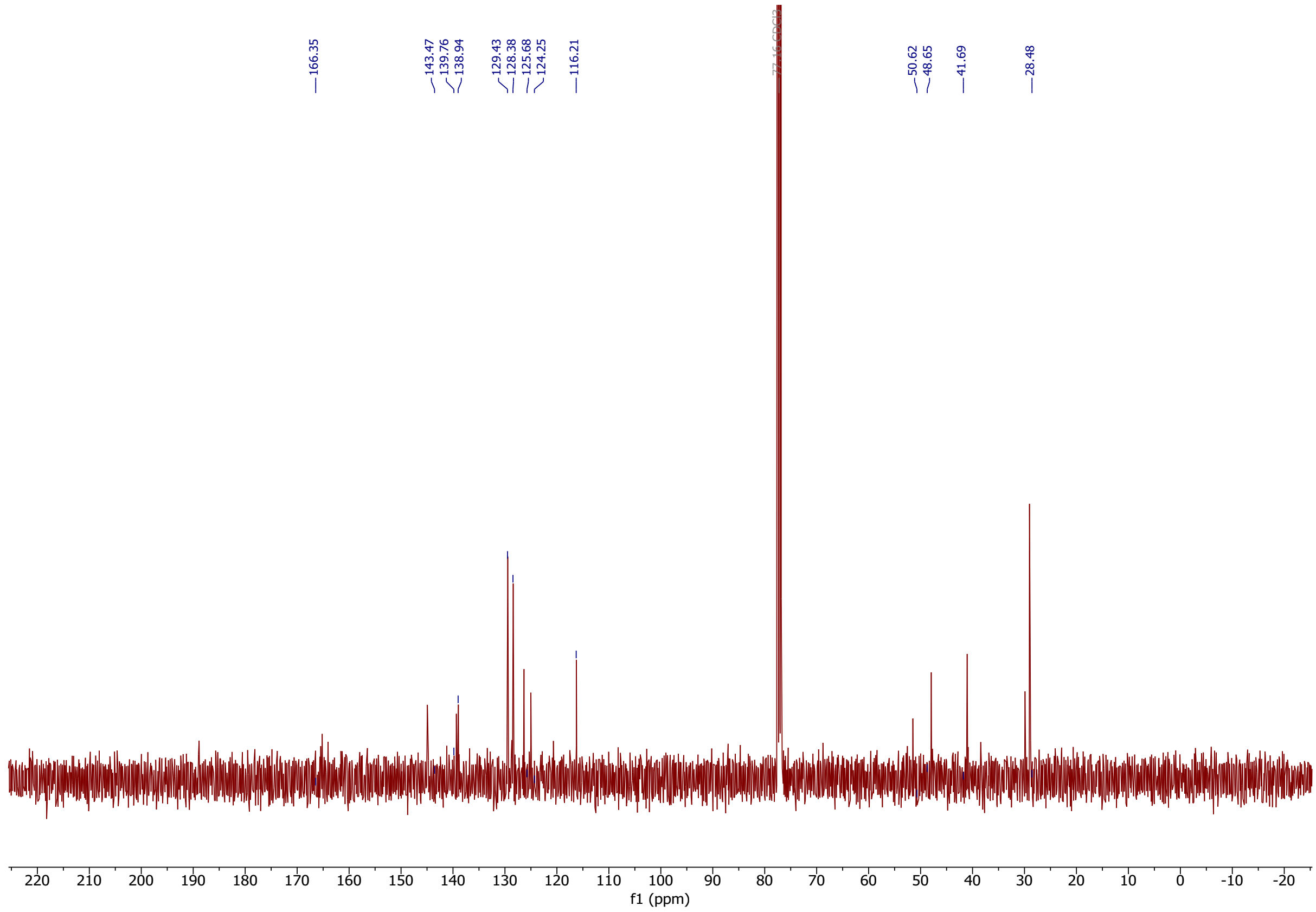

^{1}H NMR

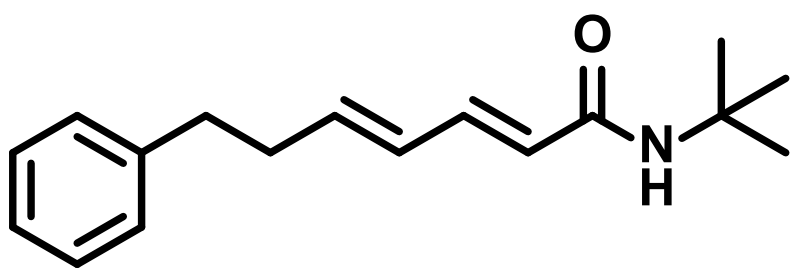

(2*E*,4*E*)-*N*-(*tert*-butyl)-7-phenylhepta-2,4-dienamide

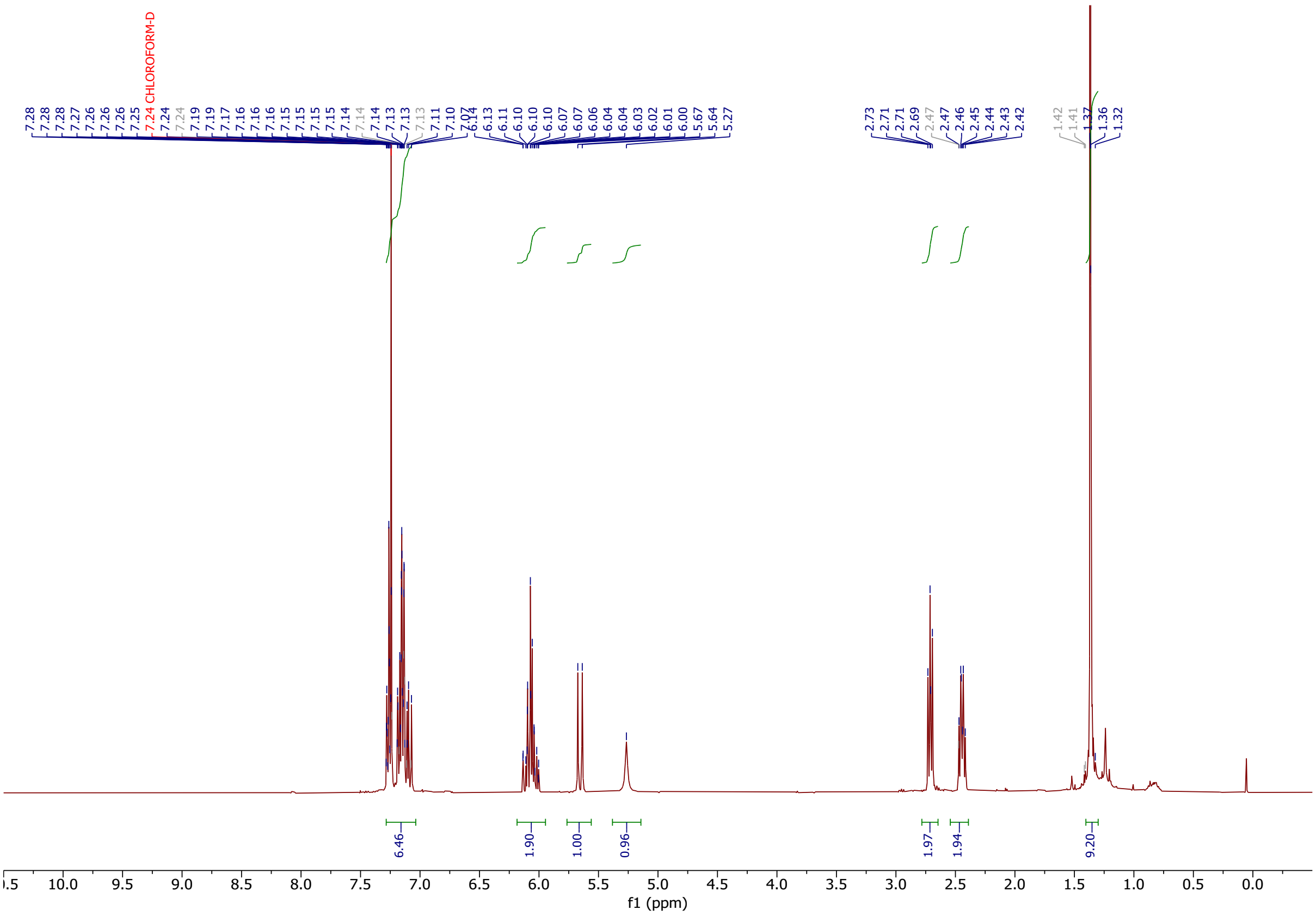

^{13}C NMR

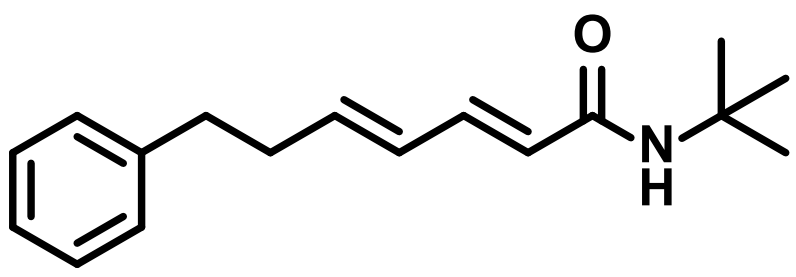

(2*E*,4*E*)-*N*-(*tert*-butyl)-7-phenylhepta-2,4-dienamide

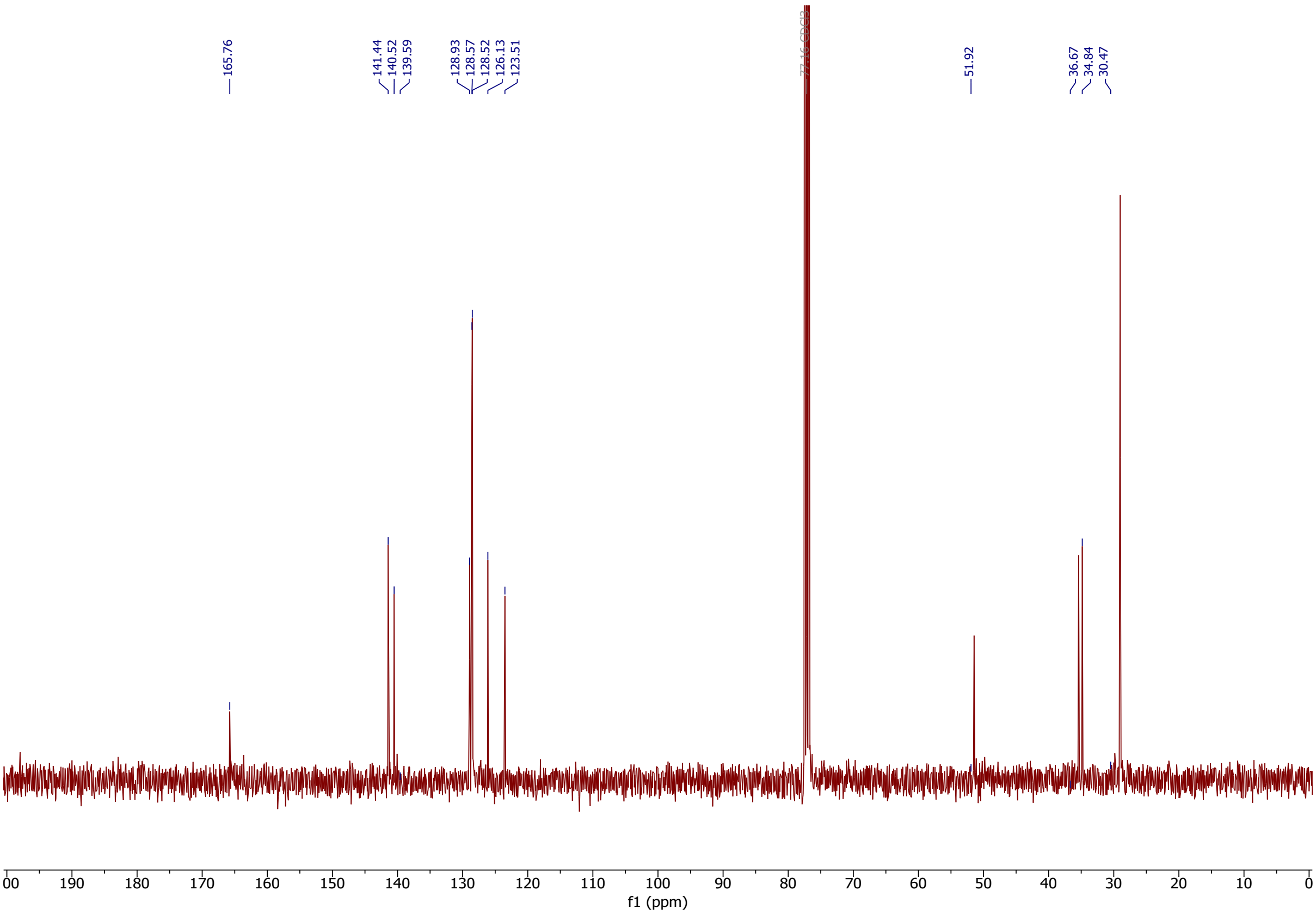

^{1}H NMR

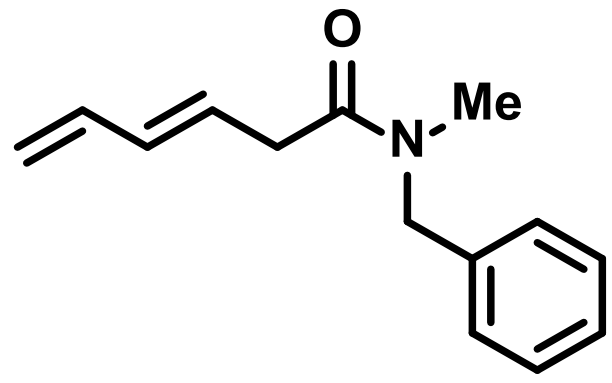

(*E*)-*N*-benzyl-*N*-methylhexa-3,5-dienamide

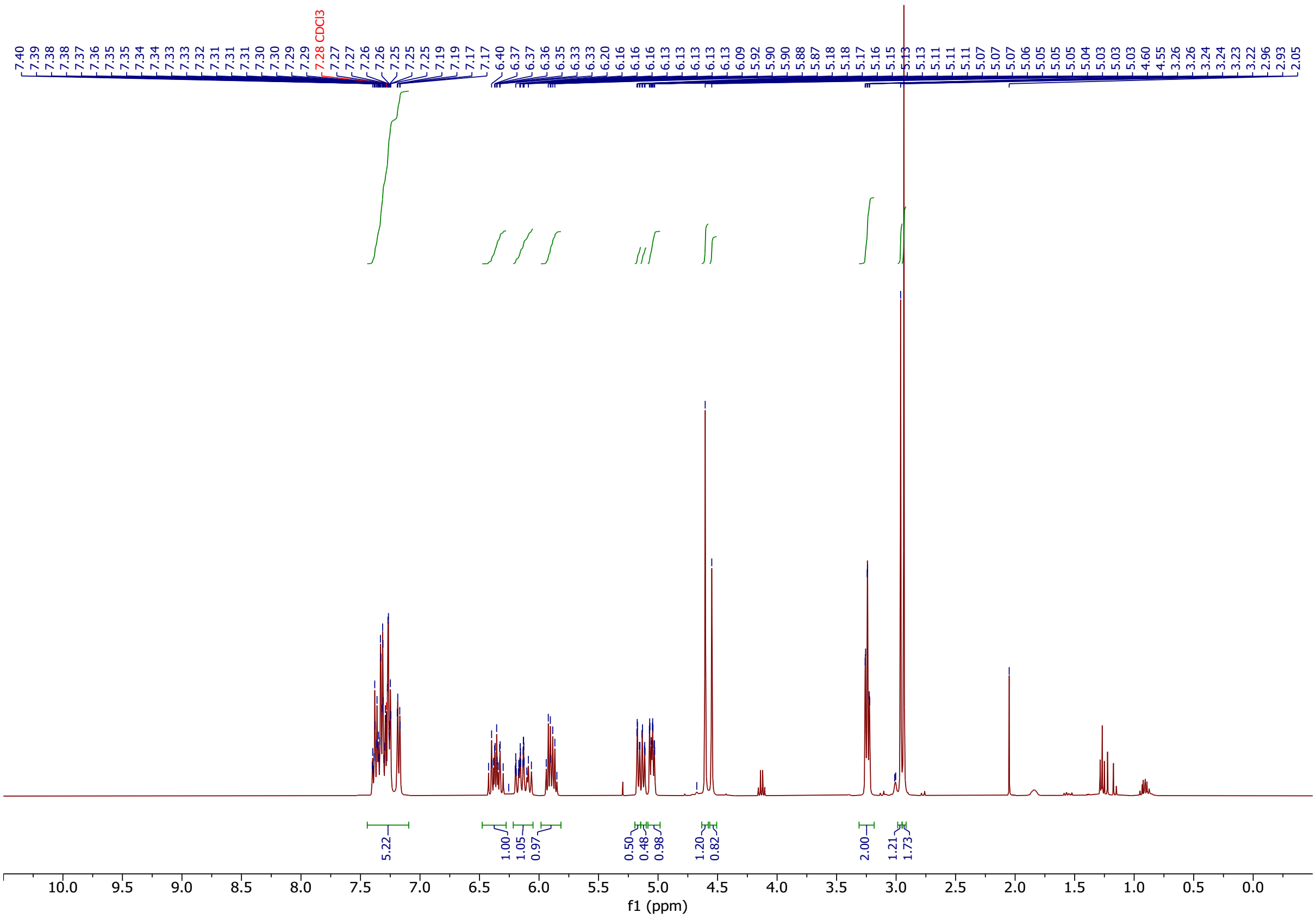

^{13}C NMR

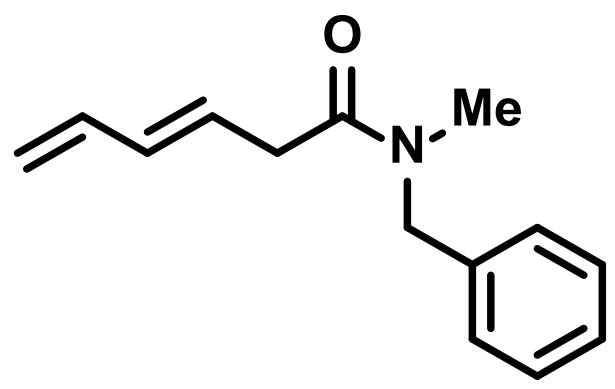

(*E*)-*N*-benzyl-*N*-methylhexa-3,5-dienamide

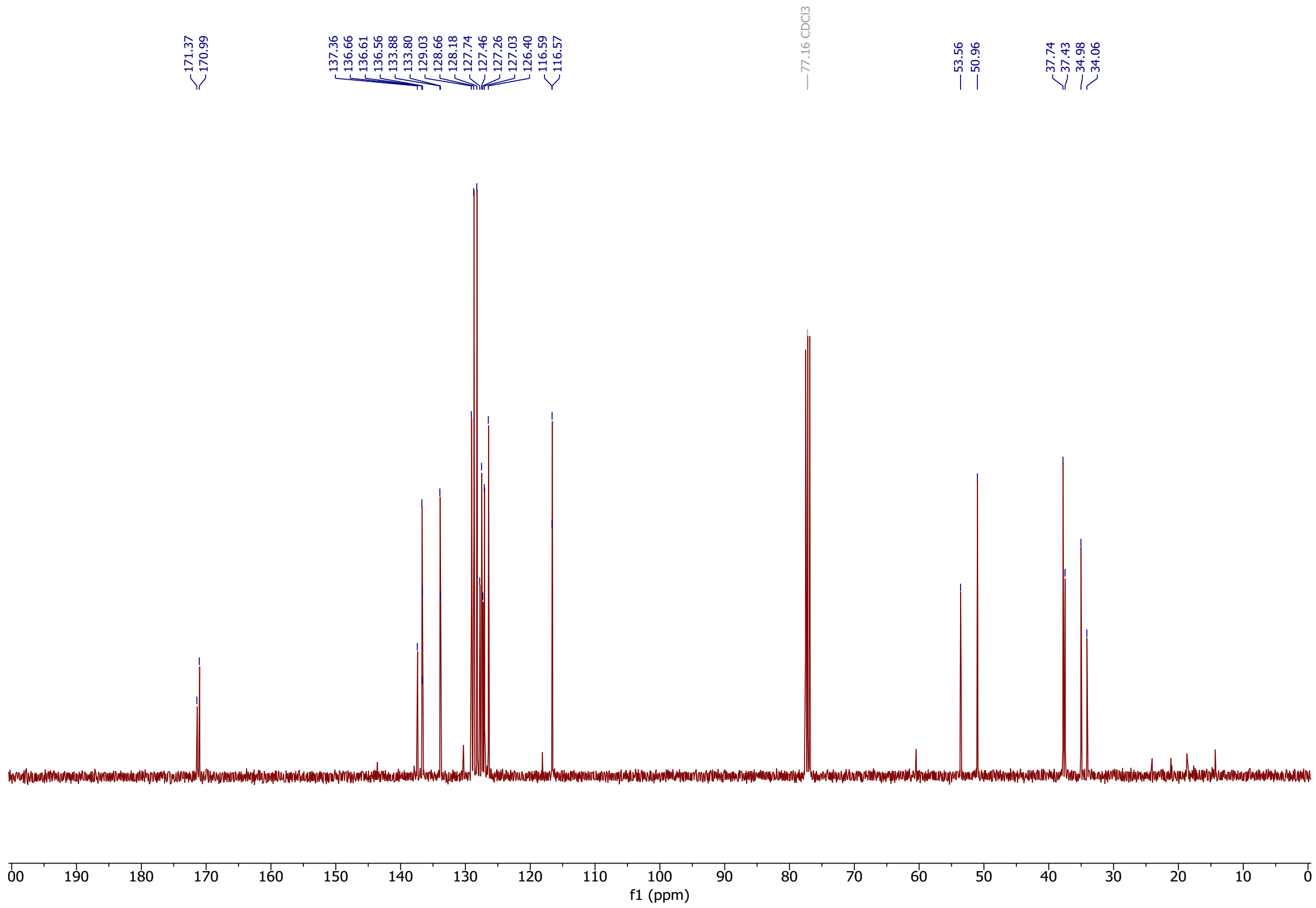

^{1}H NMR

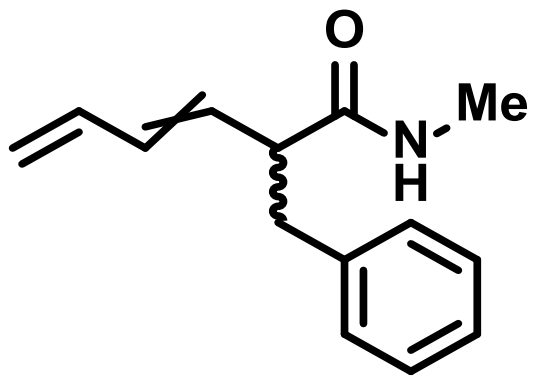

2-Benzyl-*N*-methylhexa-3,5-dienamide

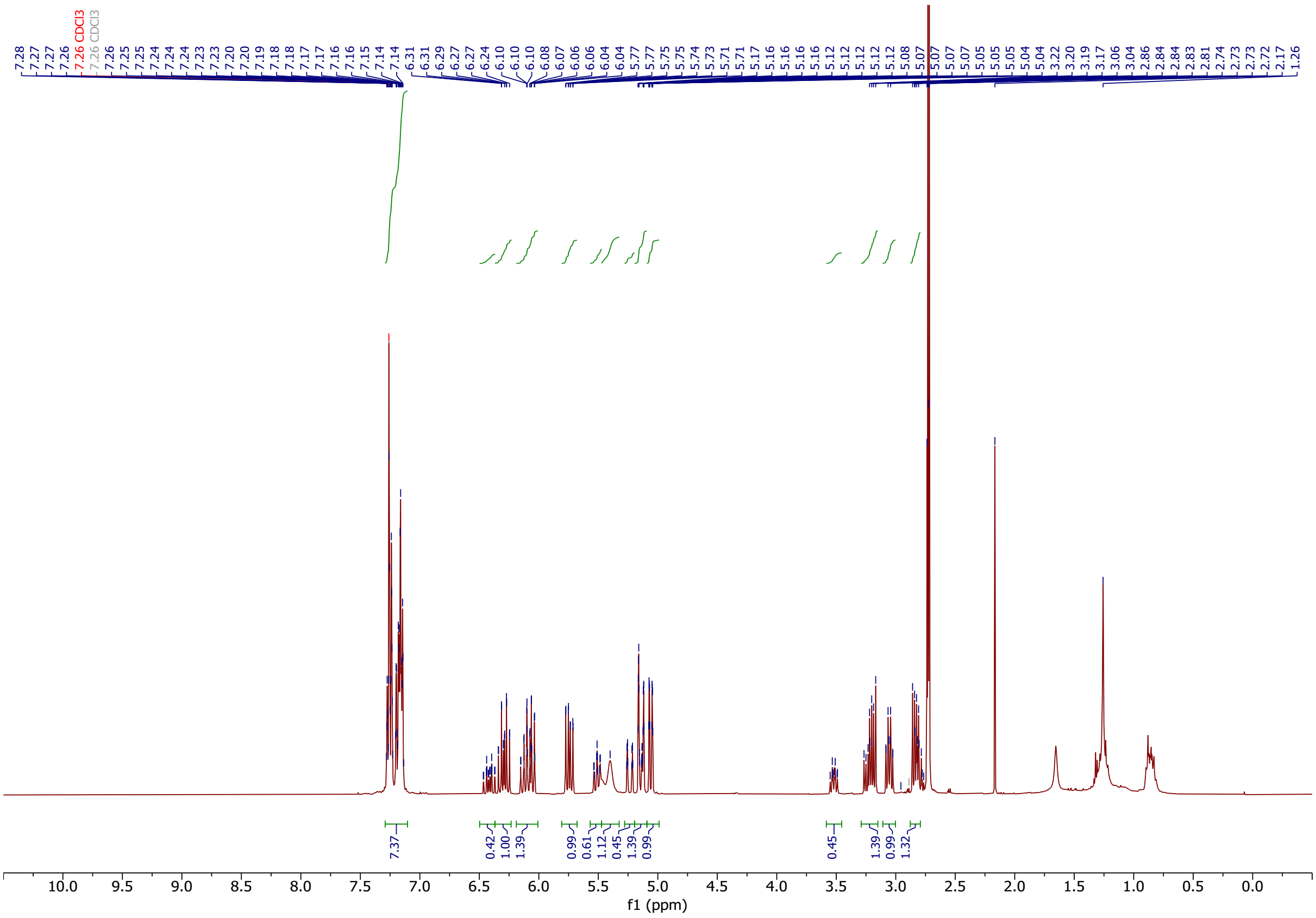

^{13}C NMR

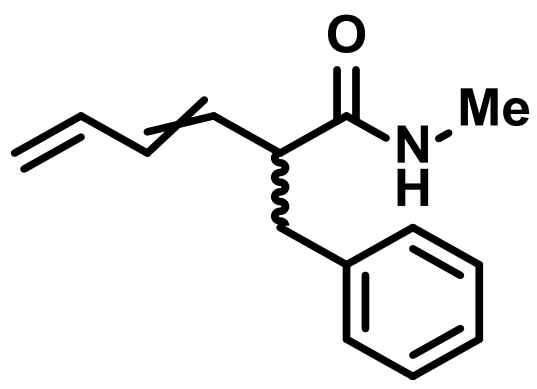

2-Benzyl-*N*-methylhexa-3,5-dienamide

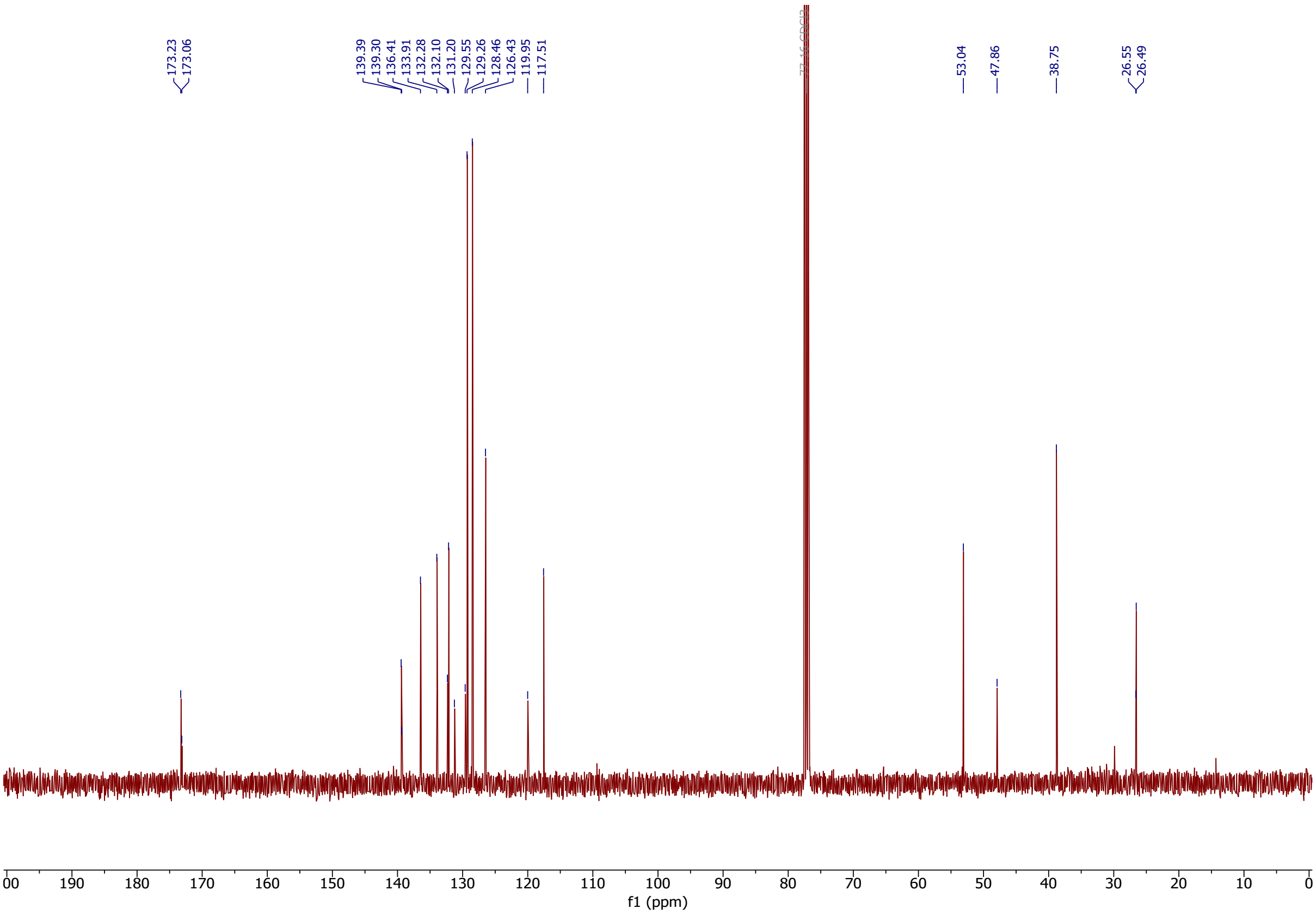

^{1}H NMR

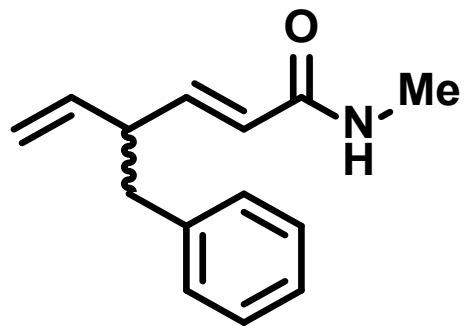

(*E*)-4-Benzyl-*N*-methylhexa-2,5-dienamide

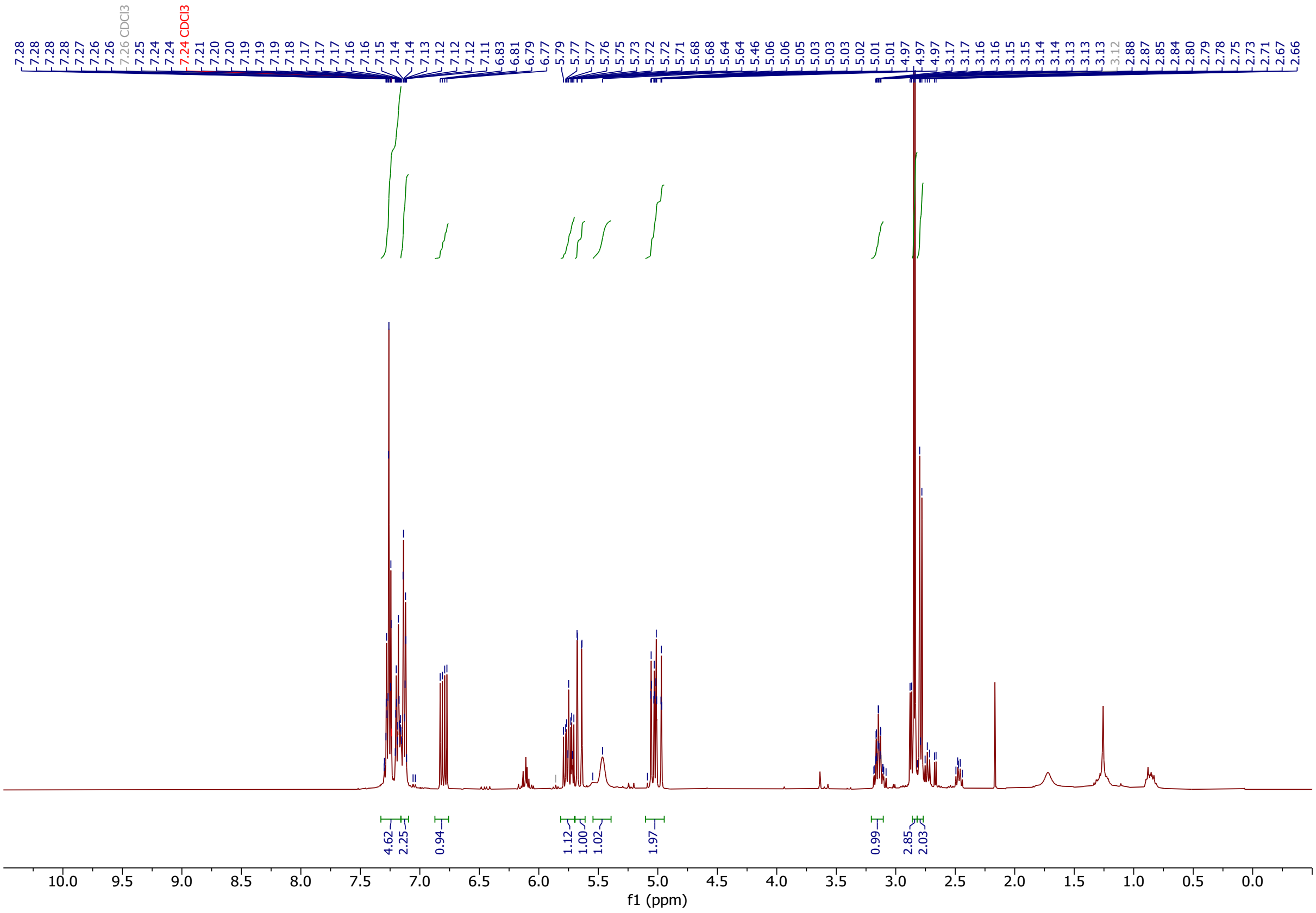

^{13}C NMR

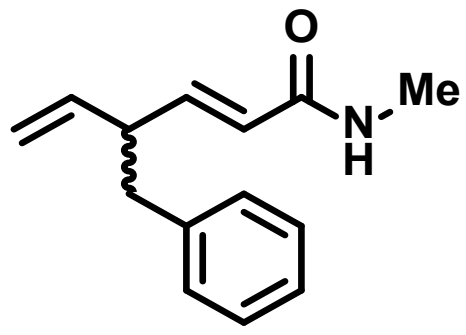

(*E*)-4-Benzyl-*N*-methylhexa-2,5-dienamide

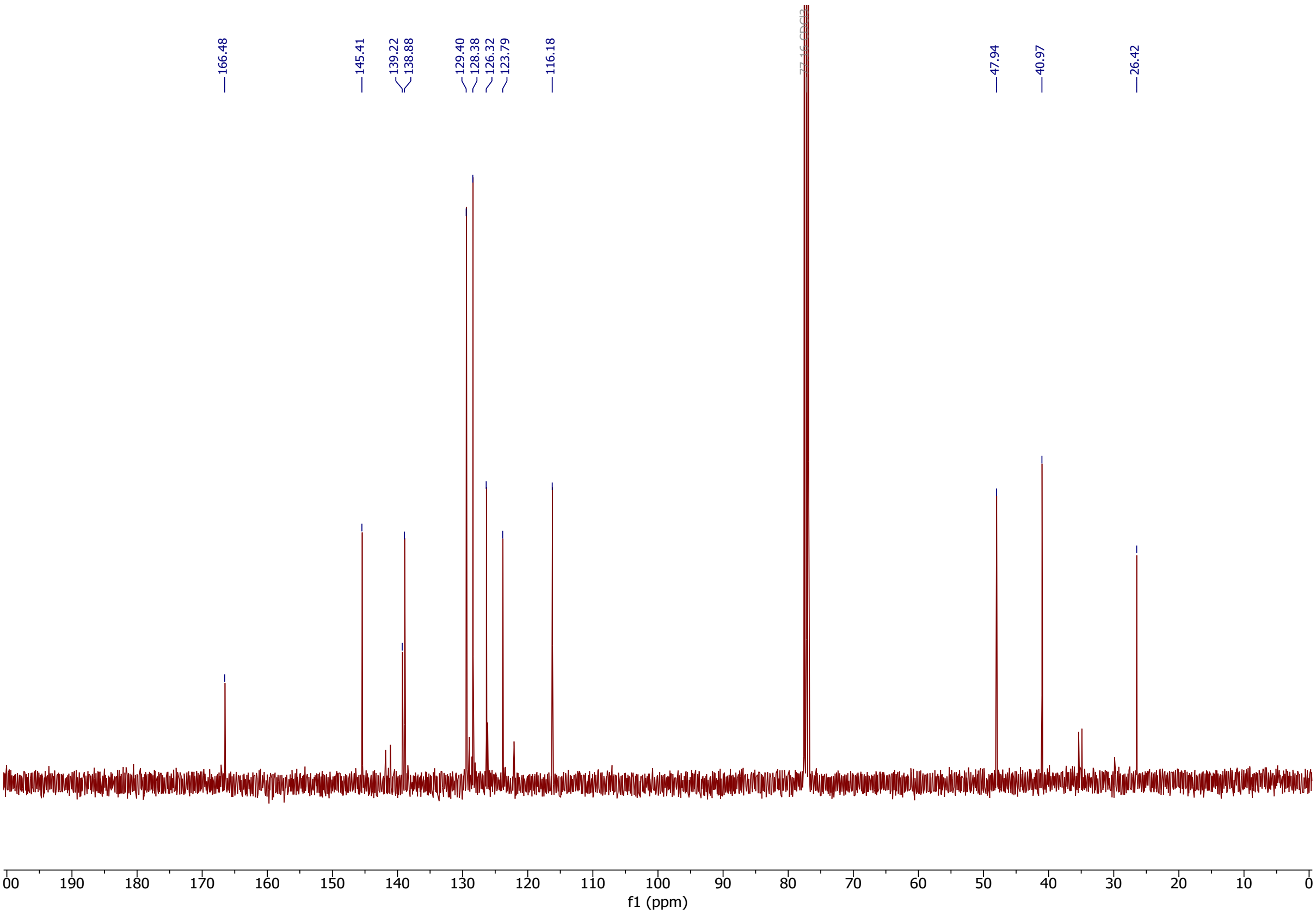

^{1}H NMR

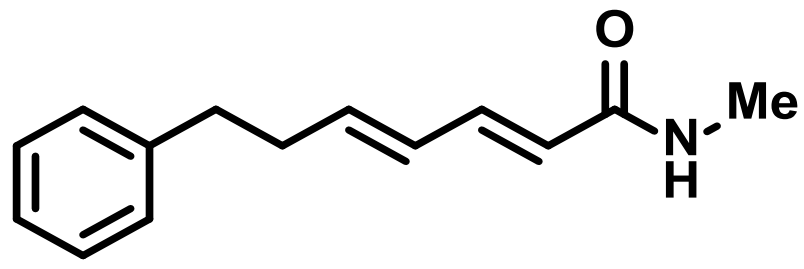

(2*E*,4*E*)-*N*-methyl-7-phenylhepta-2,4-dienamide

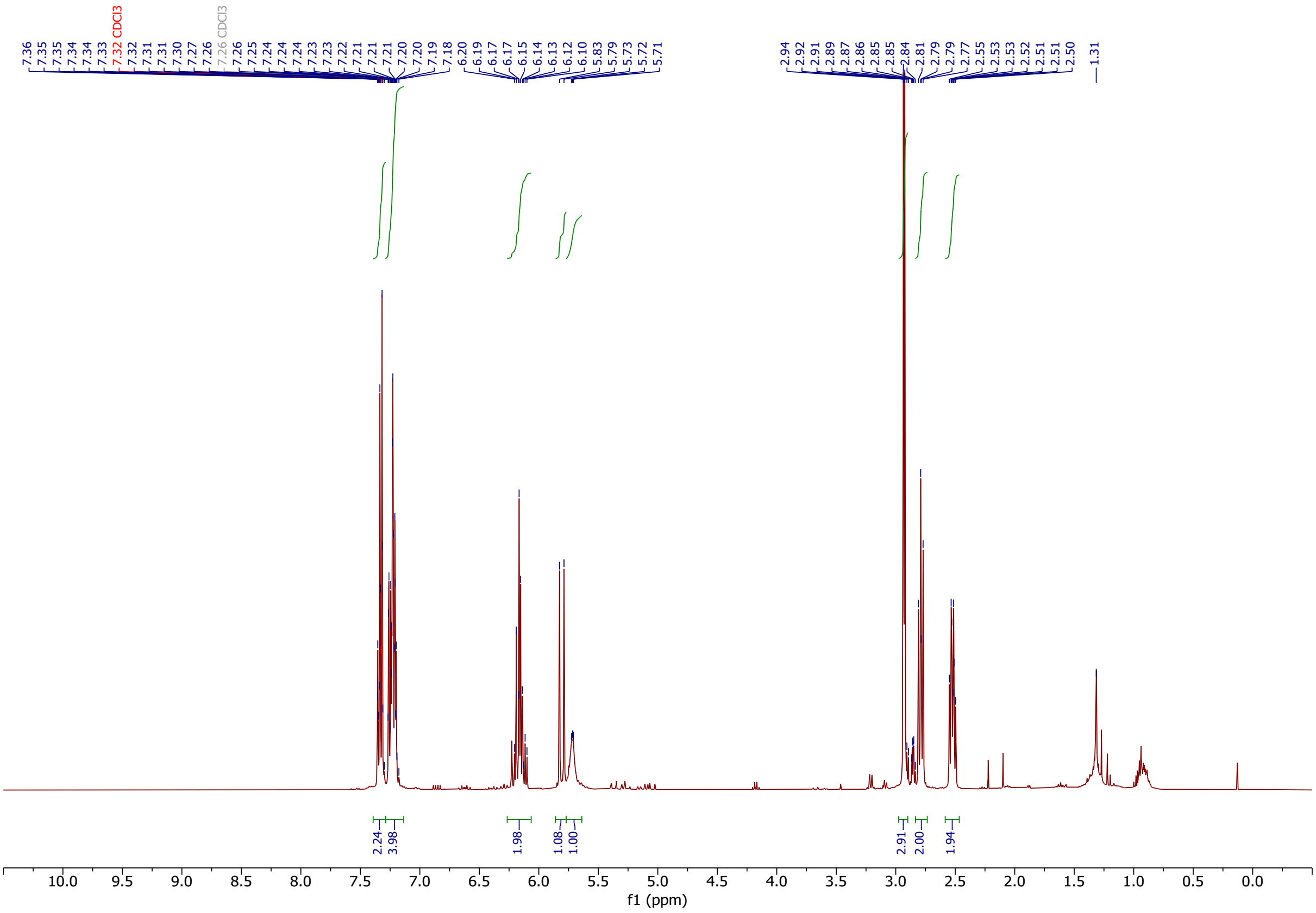

^{13}C NMR

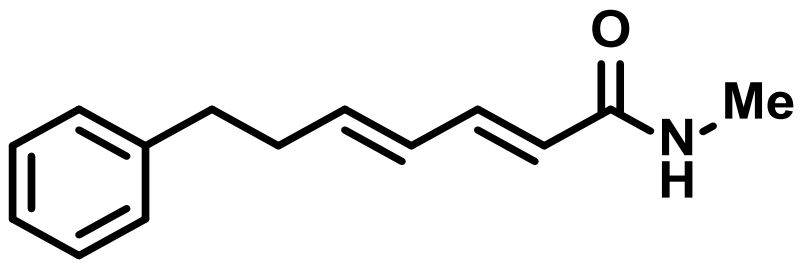

(2*E*,4*E*)-*N*-methyl-7-phenylhepta-2,4-dienamide

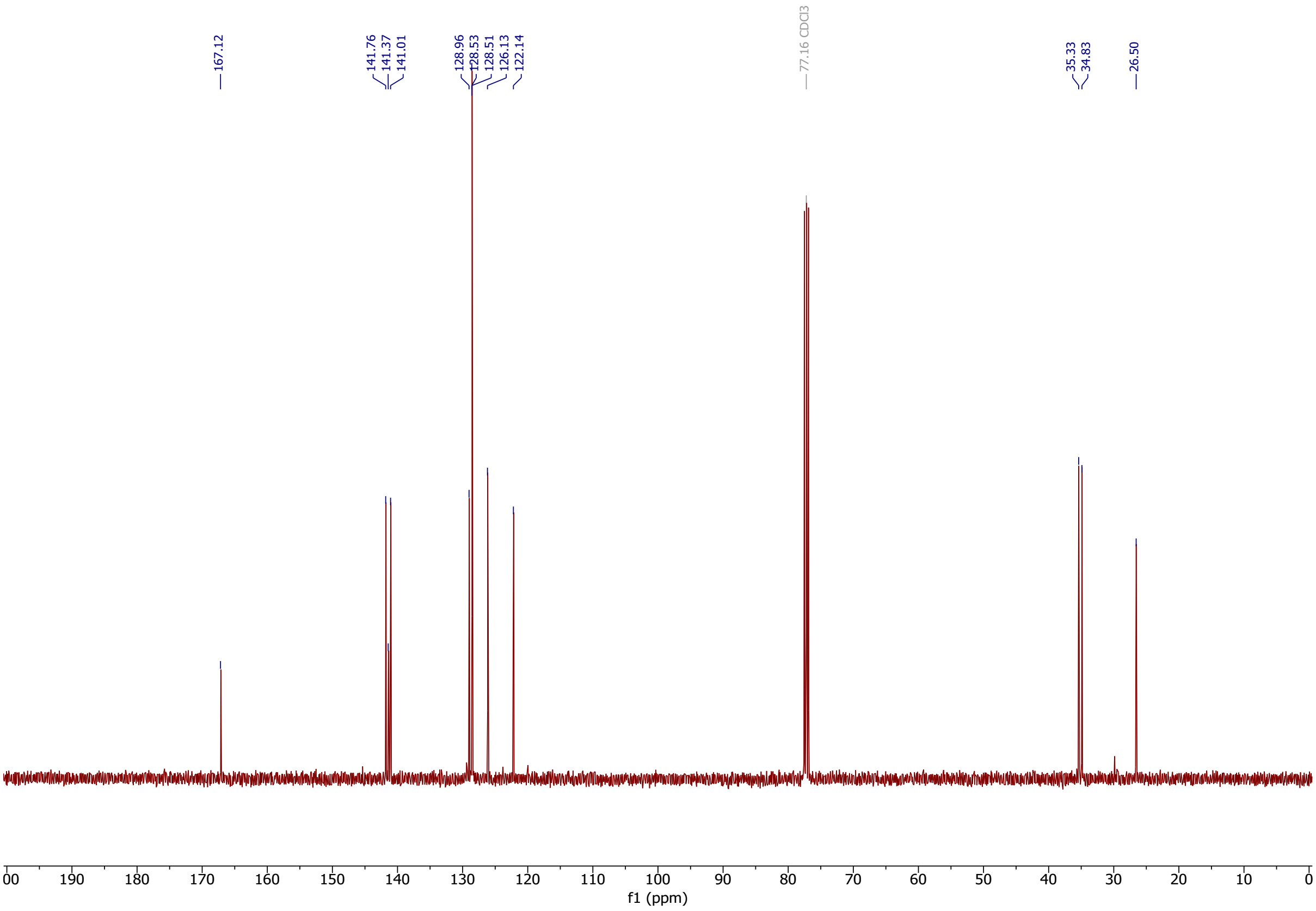

^{1}H NMR

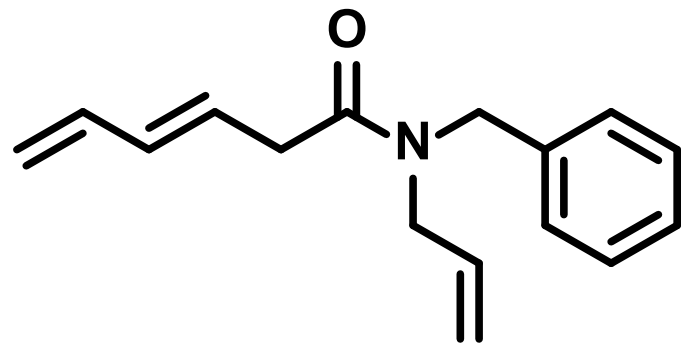

(*E*)-*N*-allyl-*N*-benzylhexa-3,5-dienamide

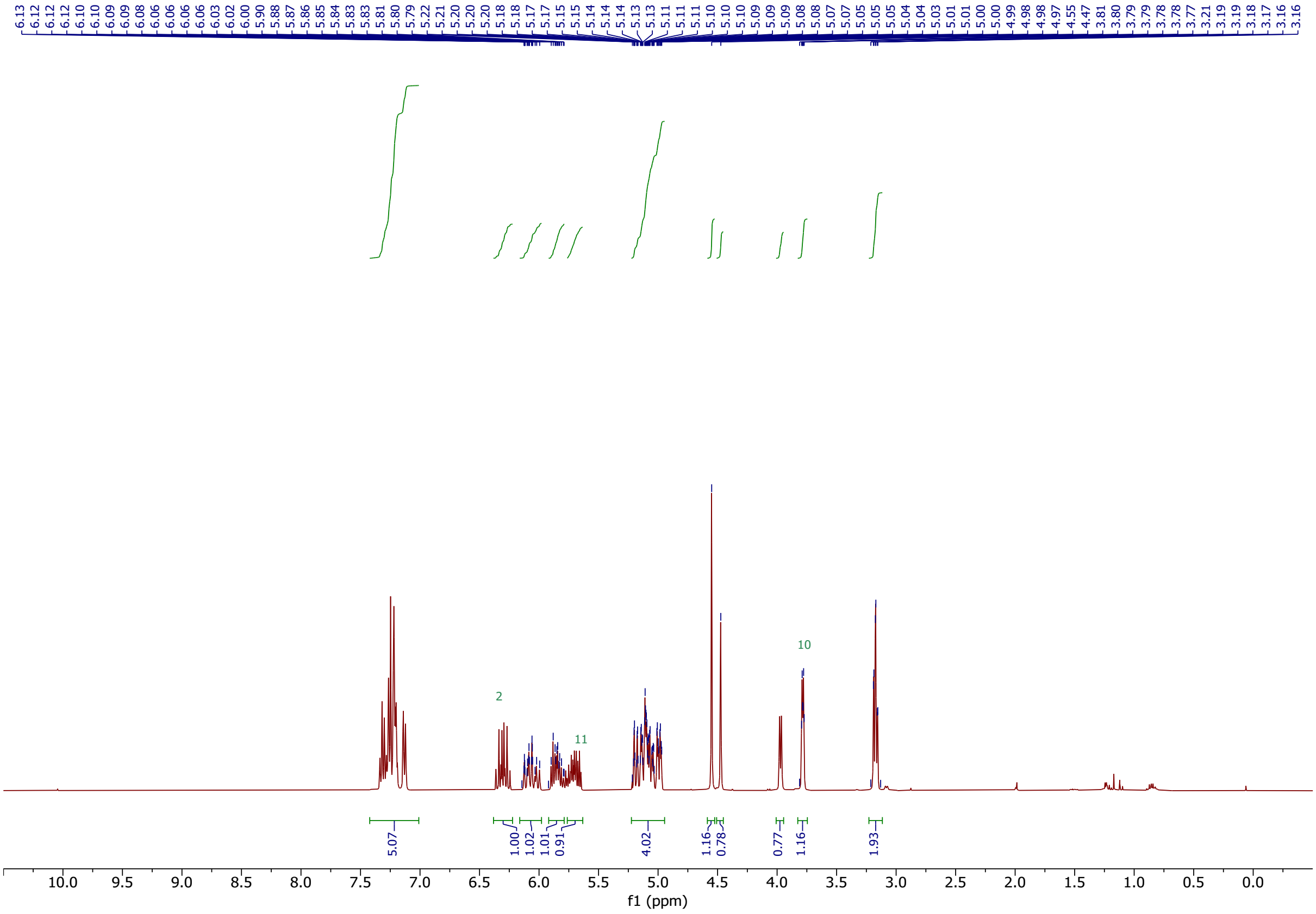

^{13}C NMR

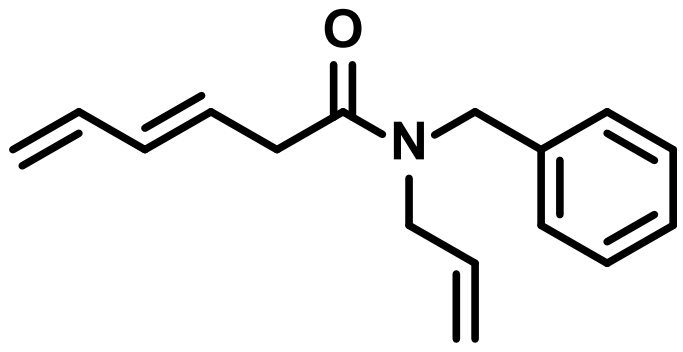

(*E*)-*N*-allyl-*N*-benzylhexa-3,5-dienamide

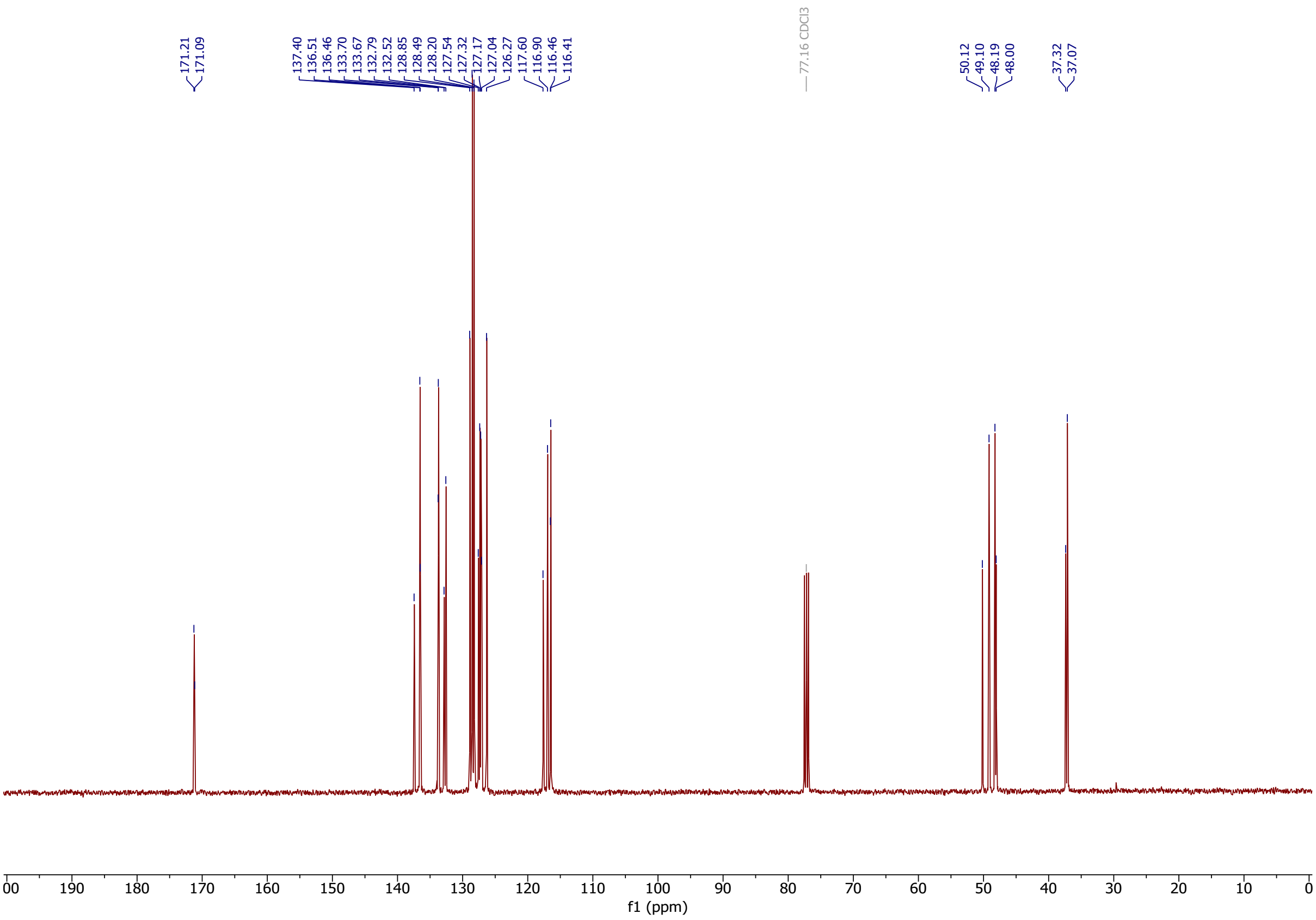

^{1}H NMR

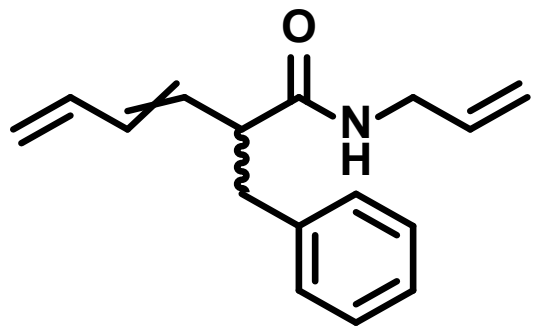

***N*-allyl-2-benzylhexa-3,5-dienamide**

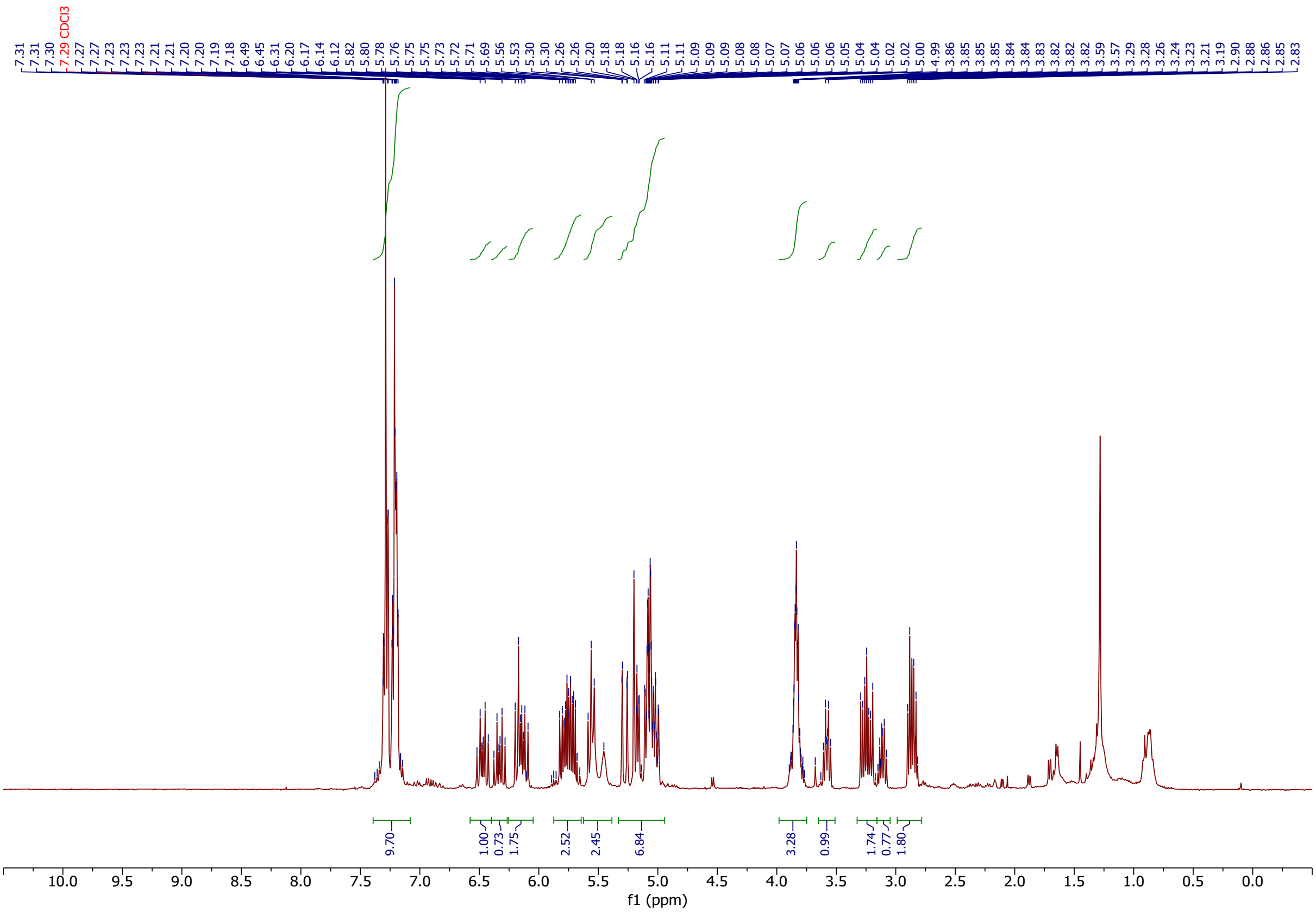

^{13}C NMR

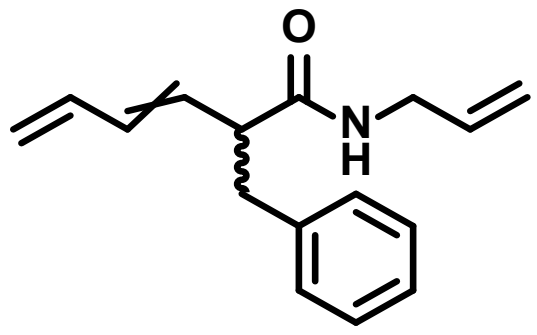

***N*-allyl-2-benzylhexa-3,5-dienamide**

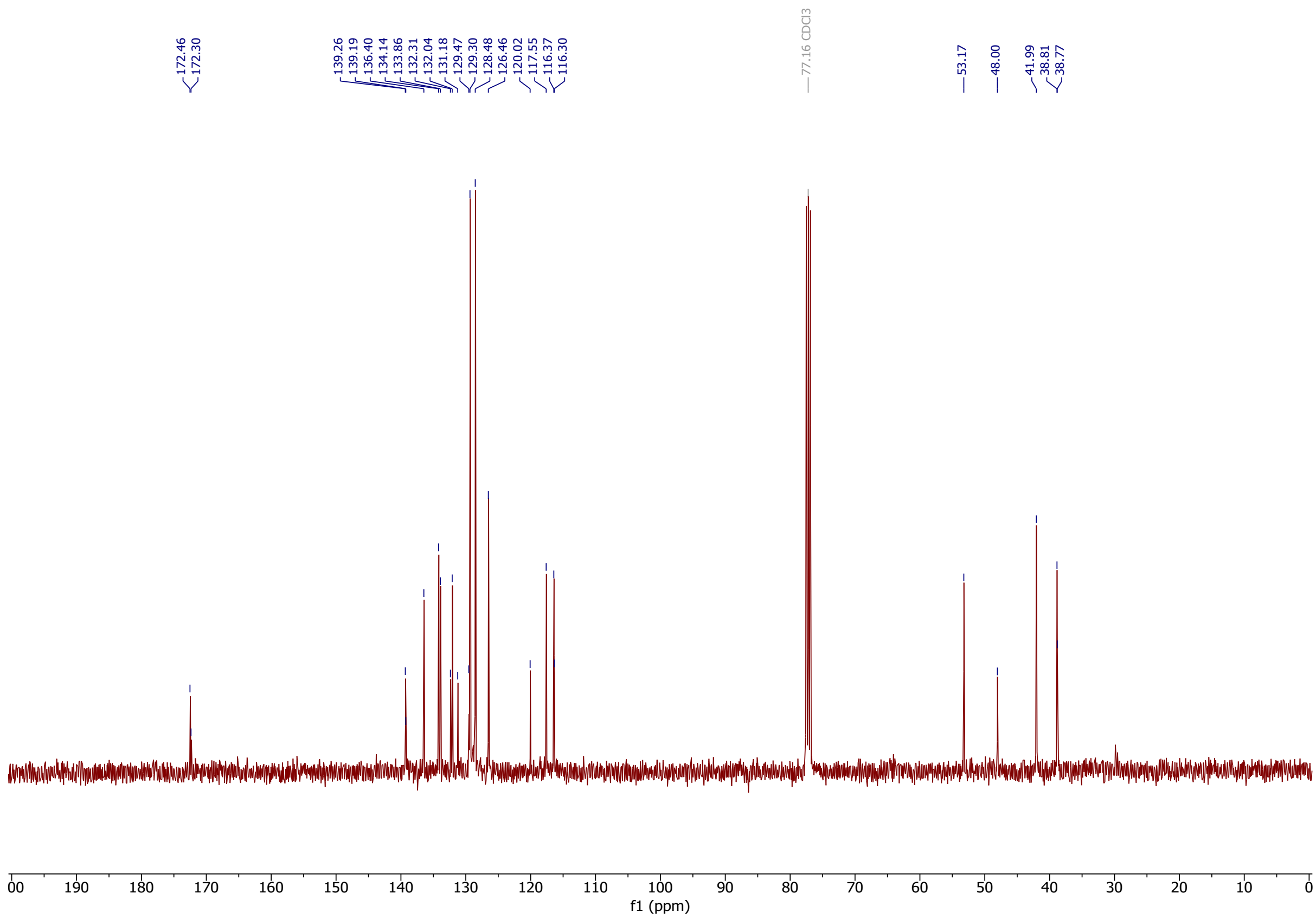

^{1}H NMR

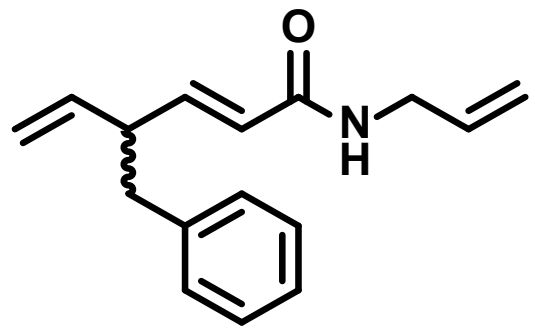

(*E*)-*N*-allyl-4-benzylhexa-2,5-dienamide

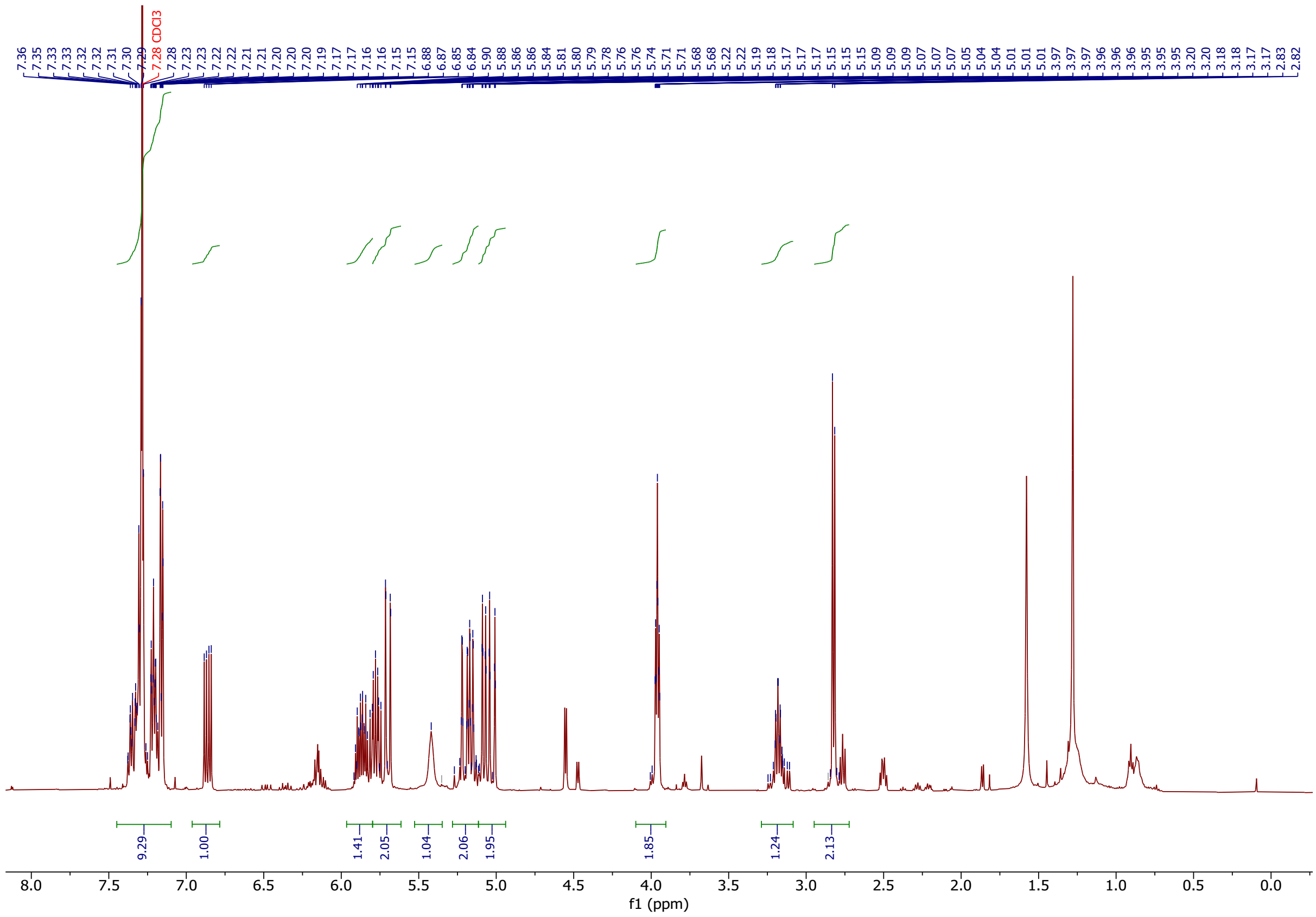

^{13}C NMR

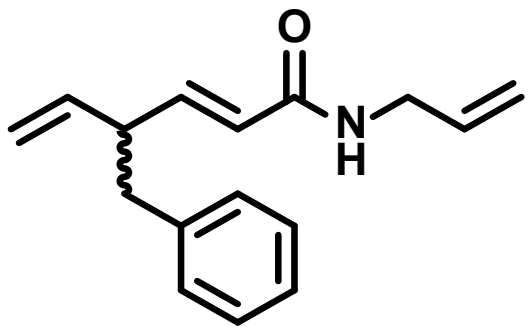

(*E*)-*N*-allyl-4-benzylhexa-2,5-dienamide

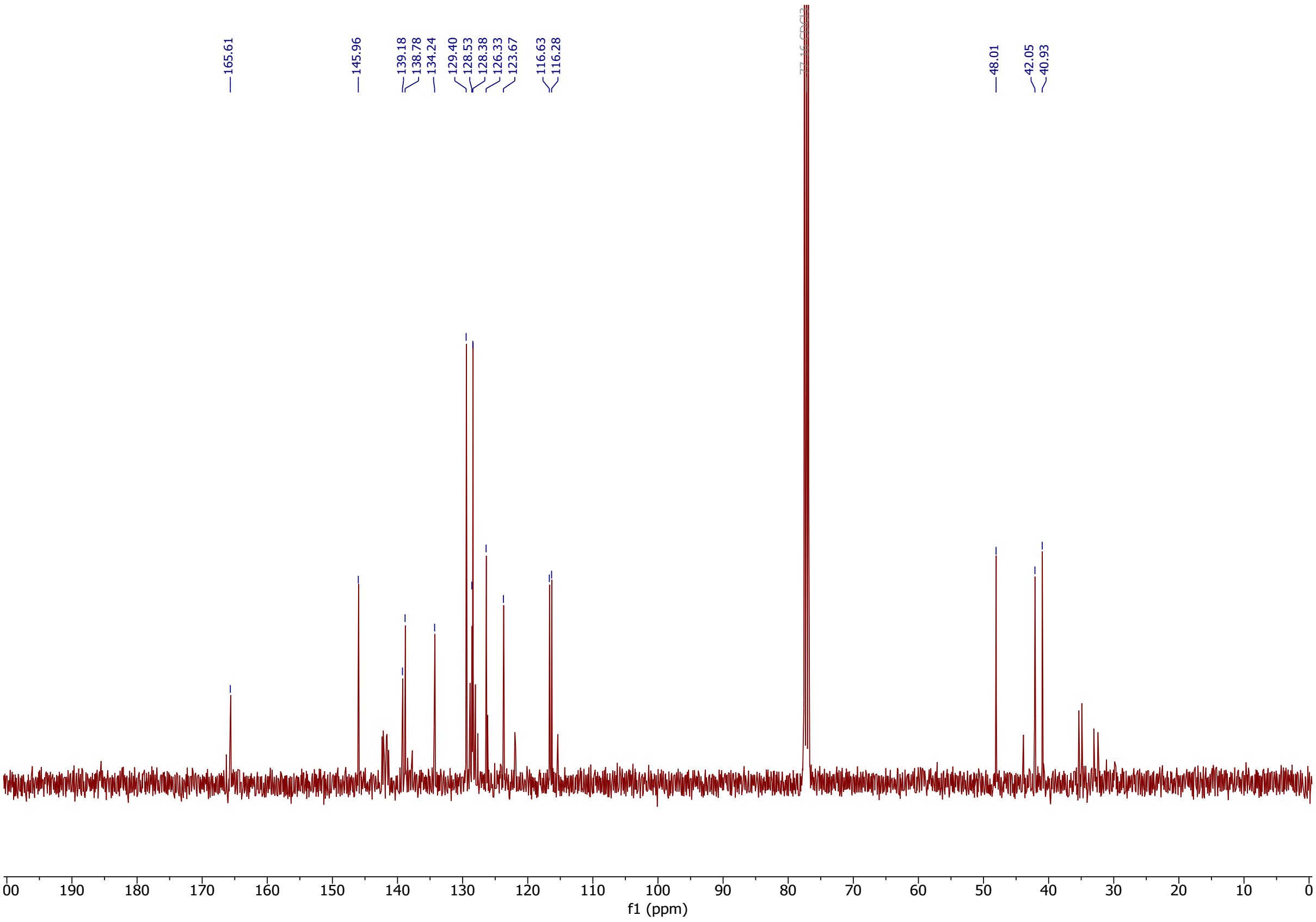

^{1}H NMR

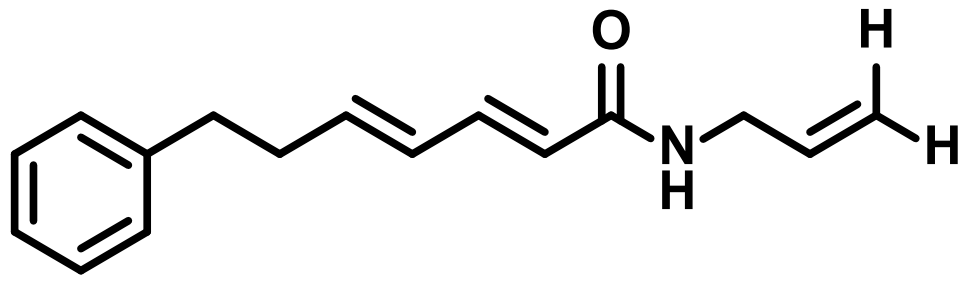

(2*E*,4*E*)-*N*-allyl-7-phenylhepta-2,4-dienamide

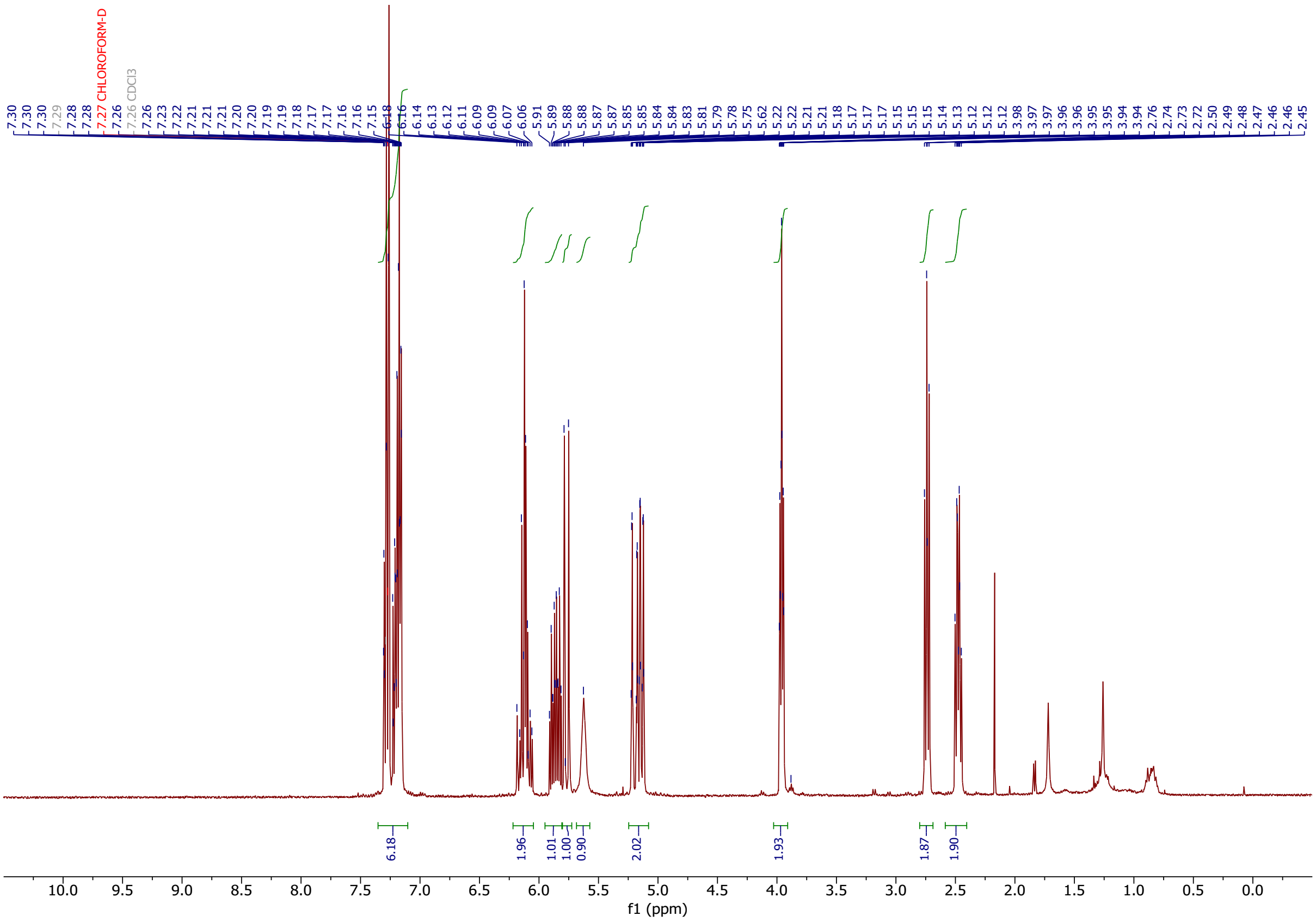

^{13}C NMR

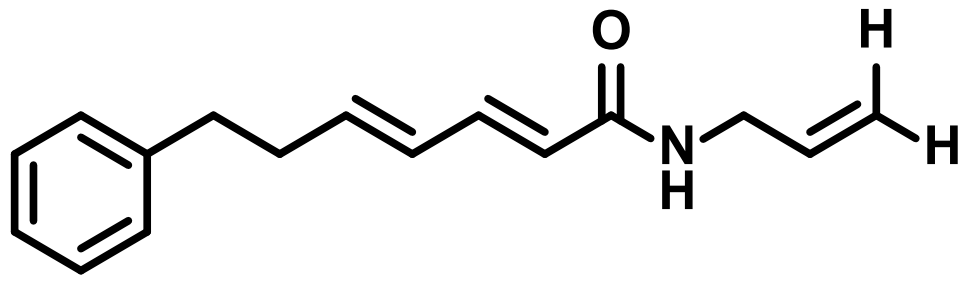

(2*E*,4*E*)-*N*-allyl-7-phenylhepta-2,4-dienamide

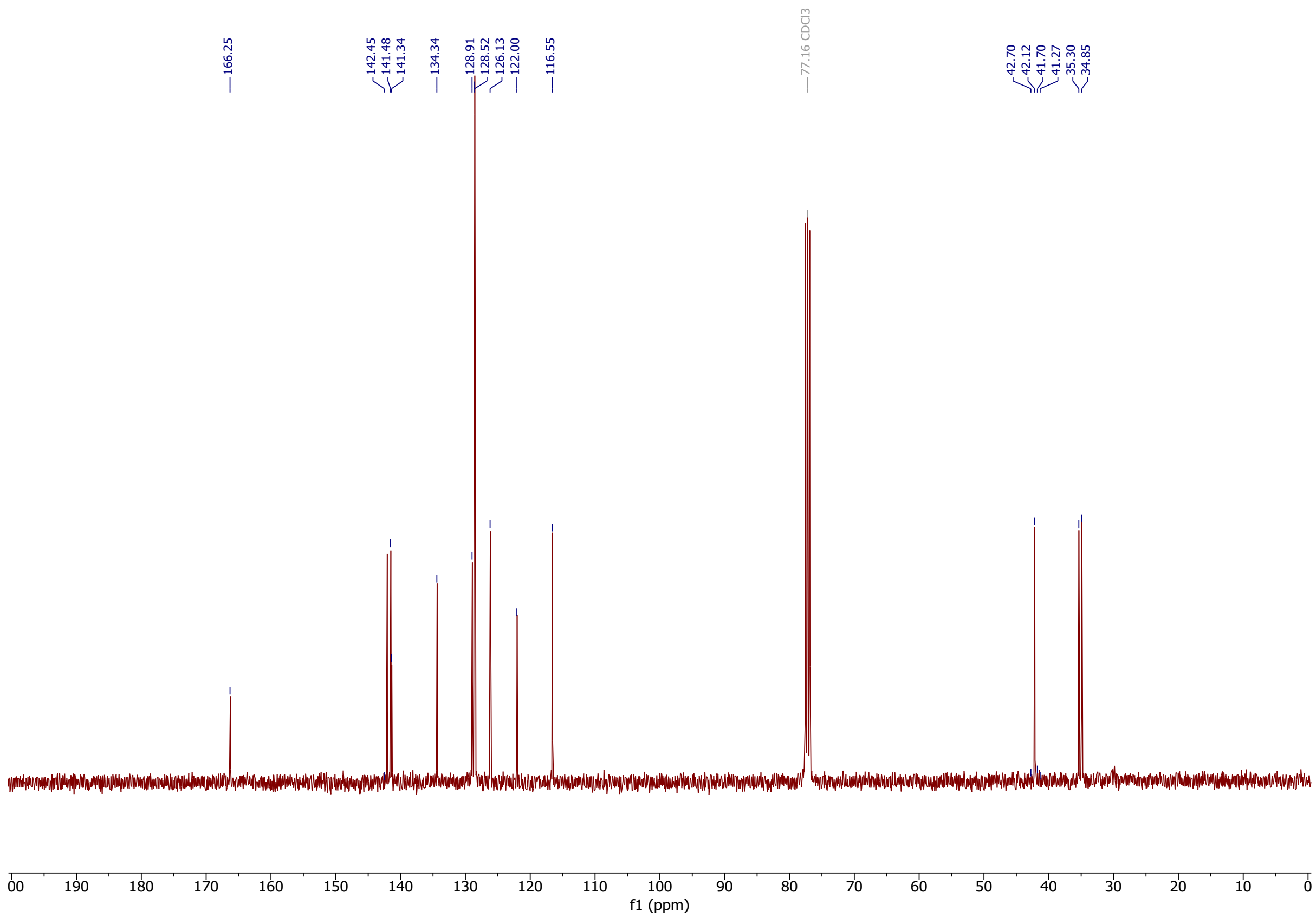

^{1}H NMR

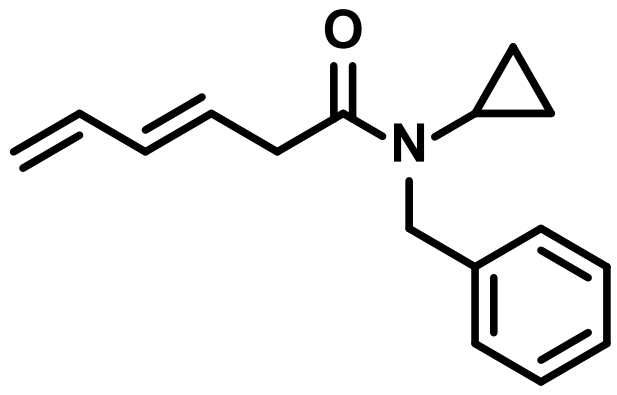

(*E*)-*N*-benzyl-*N*-cyclopropylhexa-3,5-dienamide

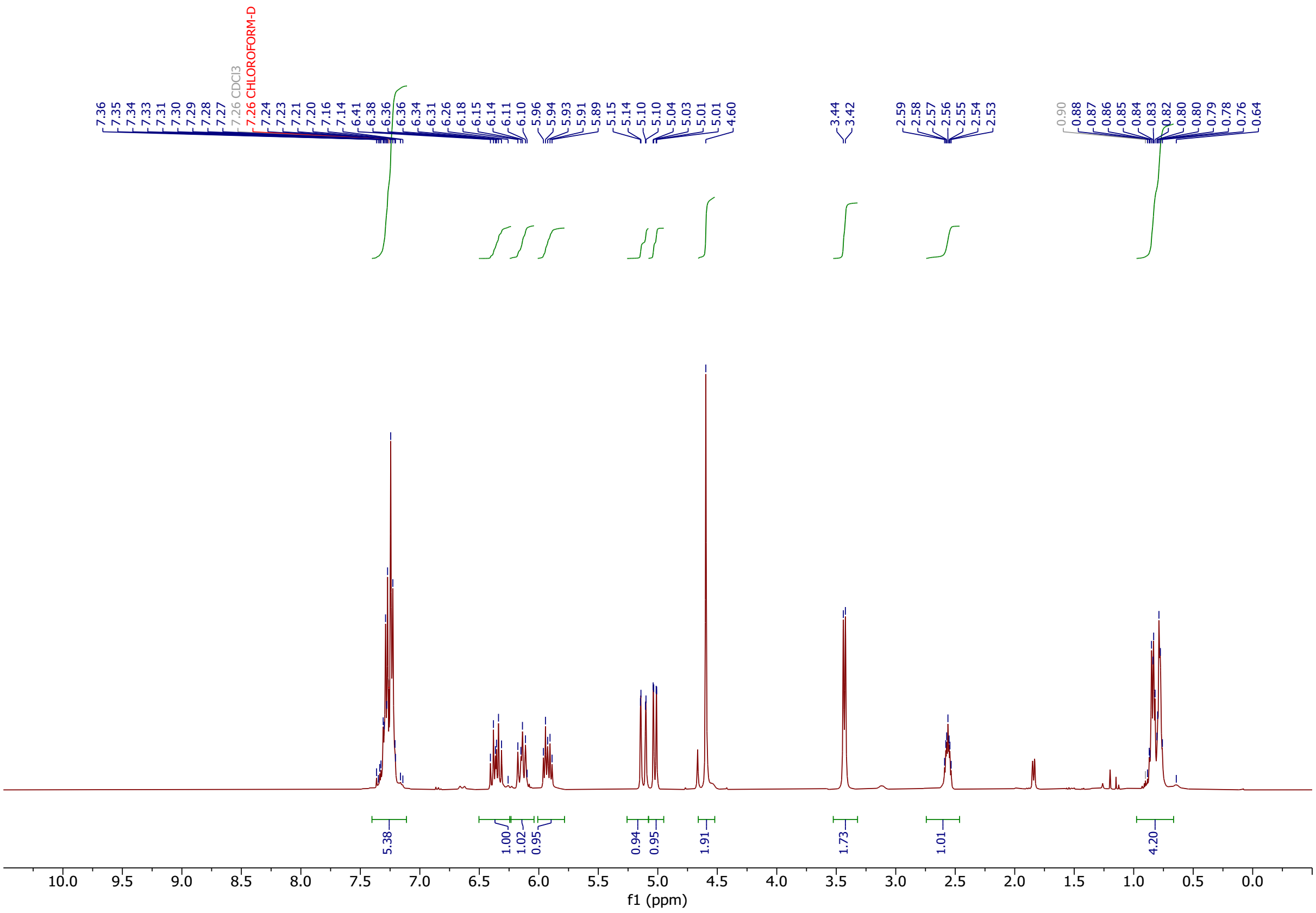

^{13}C NMR

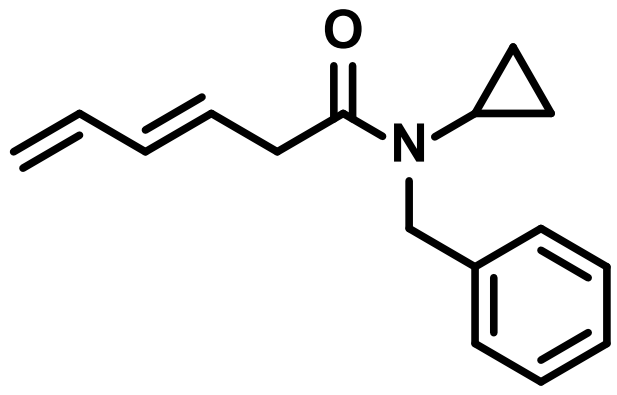

(*E*)-*N*-benzyl-*N*-cyclopropylhexa-3,5-dienamide

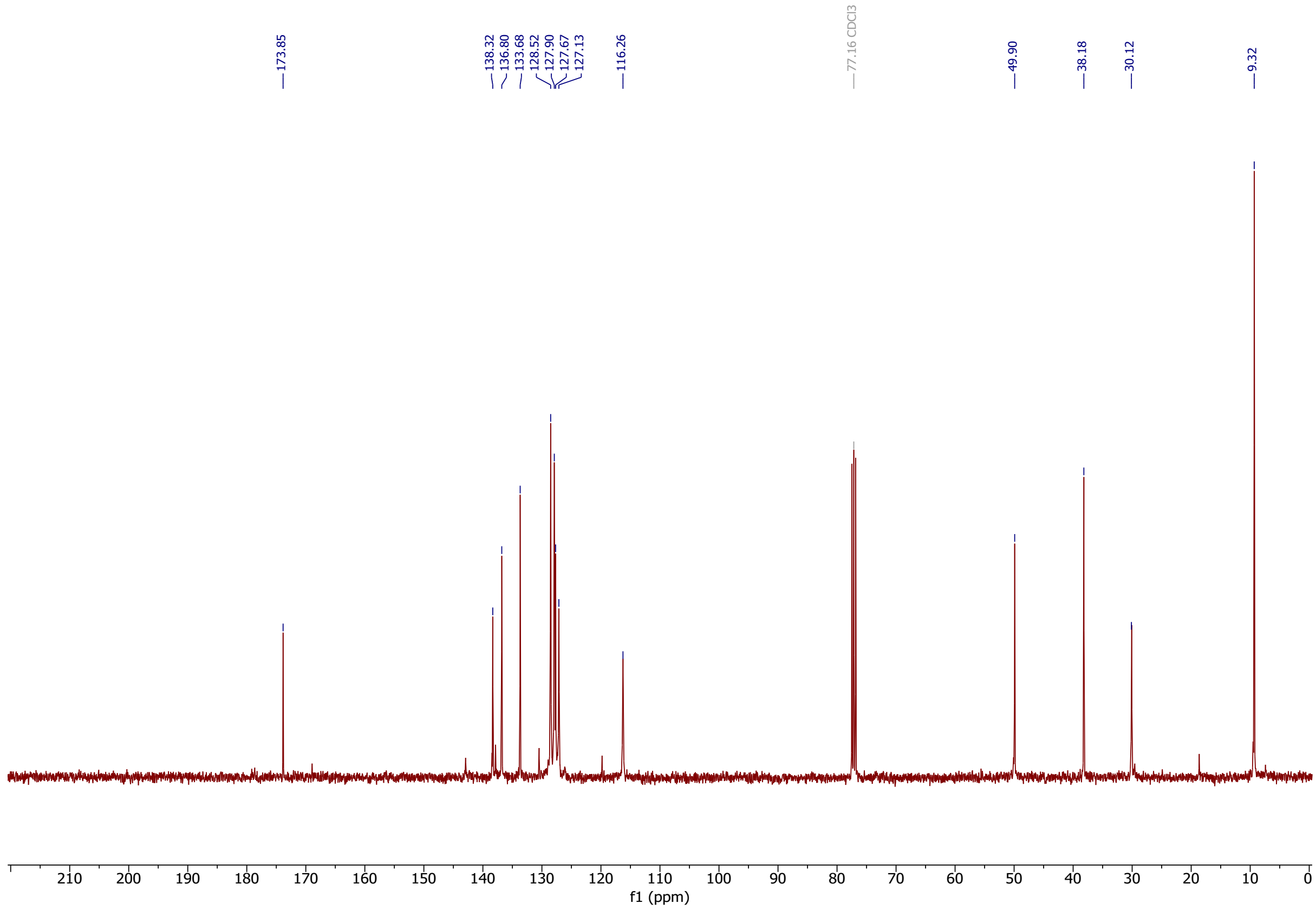

^{1}H NMR

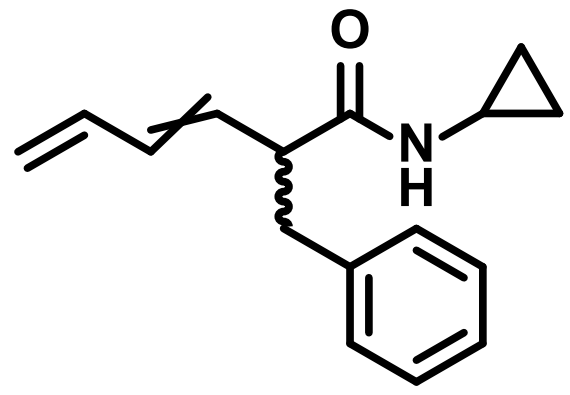

2-Benzyl-*N*-cyclopropylhexa-3,5-dienamide

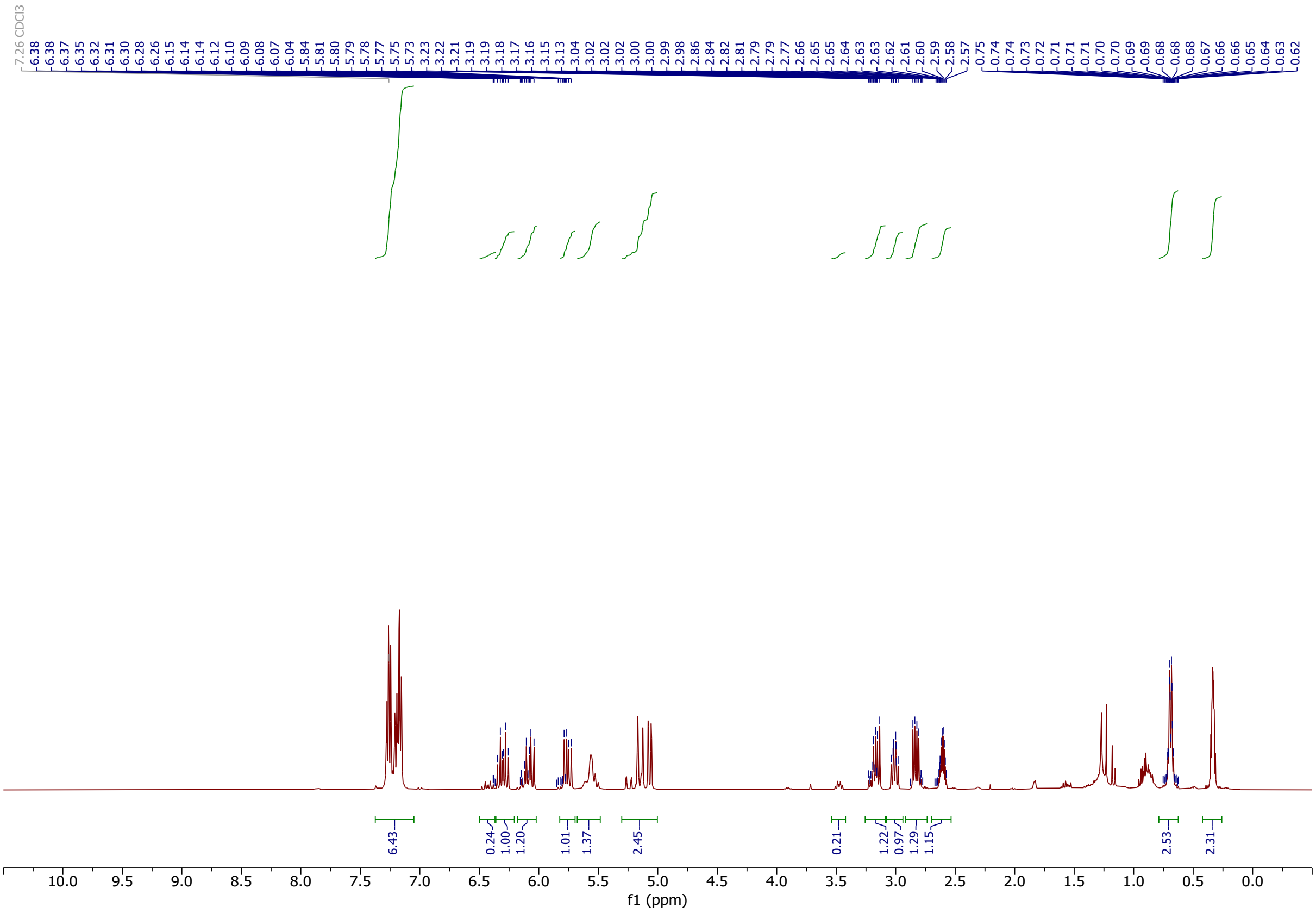

^{13}C NMR

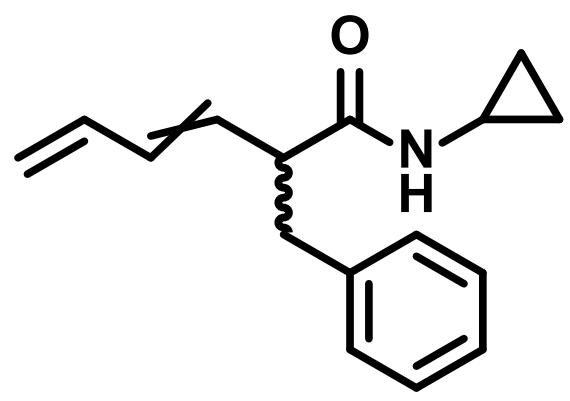

2-Benzyl-*N*-cyclopropylhexa-3,5-dienamide

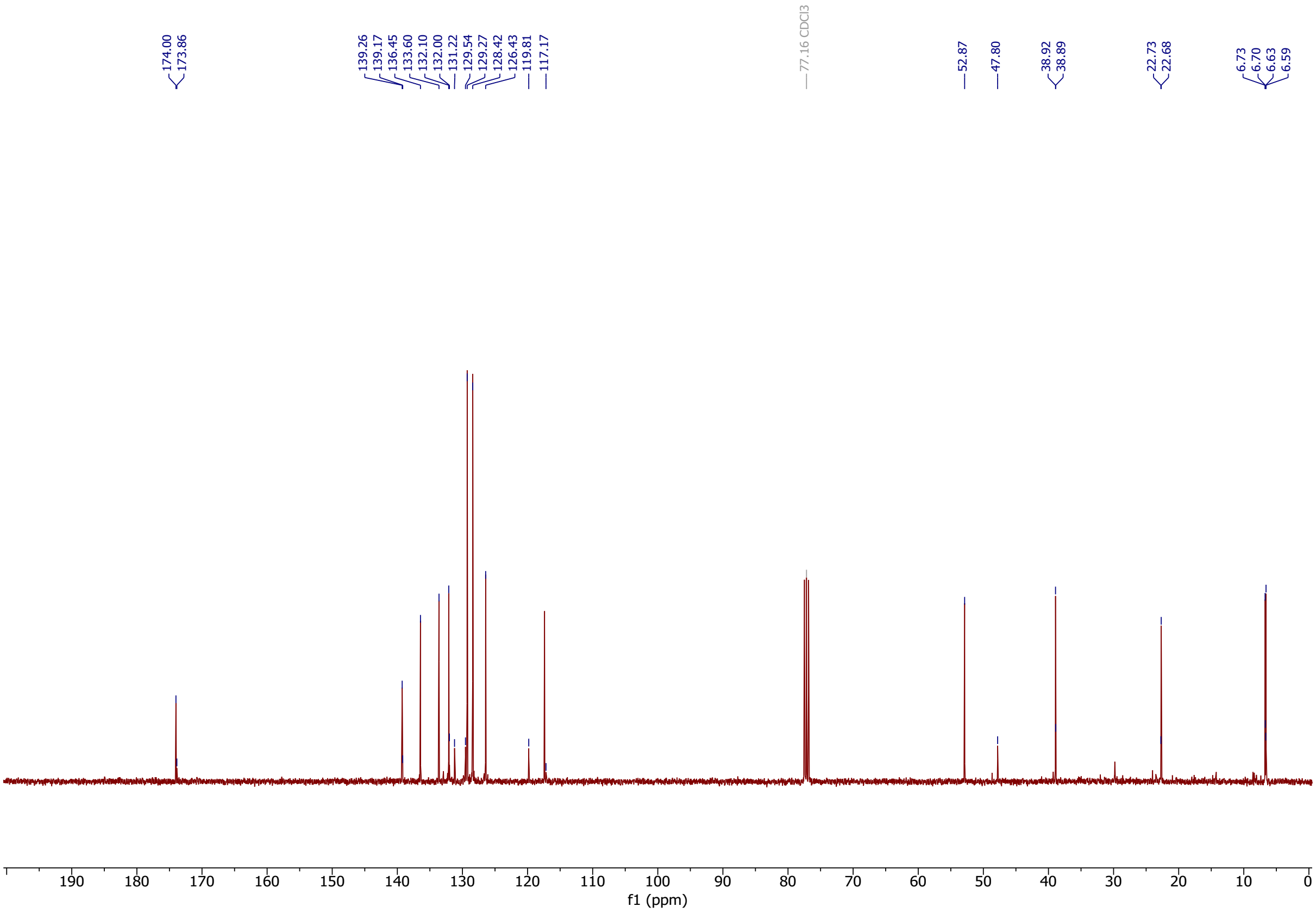

¹H NMR

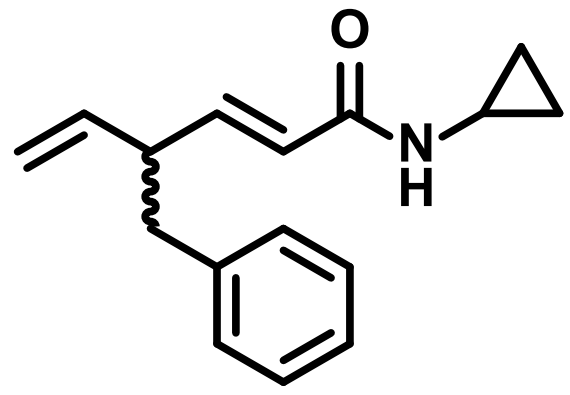

(*E*)-4-benzyl-*N*-cyclopropylhexa-2,5-dienamide

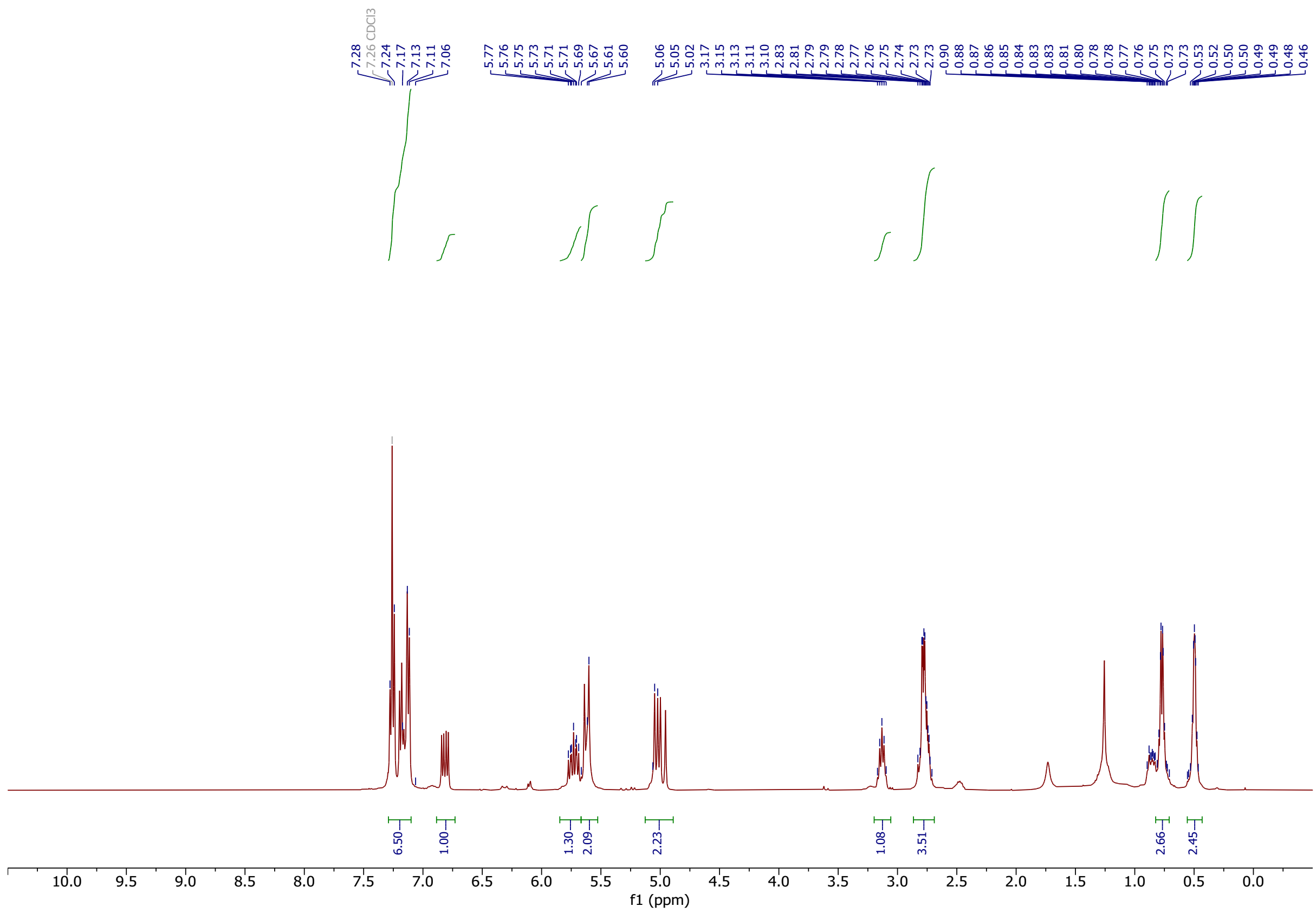

^{13}C NMR

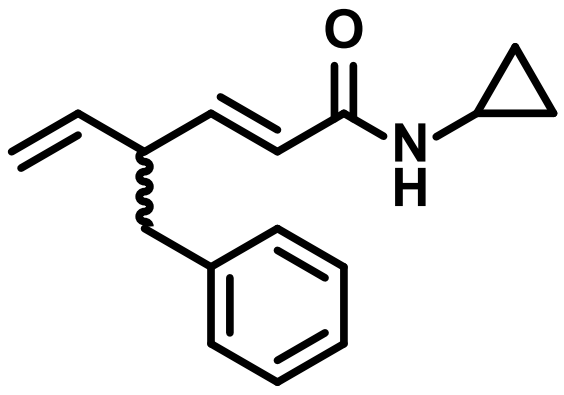

(*E*)-4-benzyl-*N*-cyclopropylhexa-2,5-dienamide

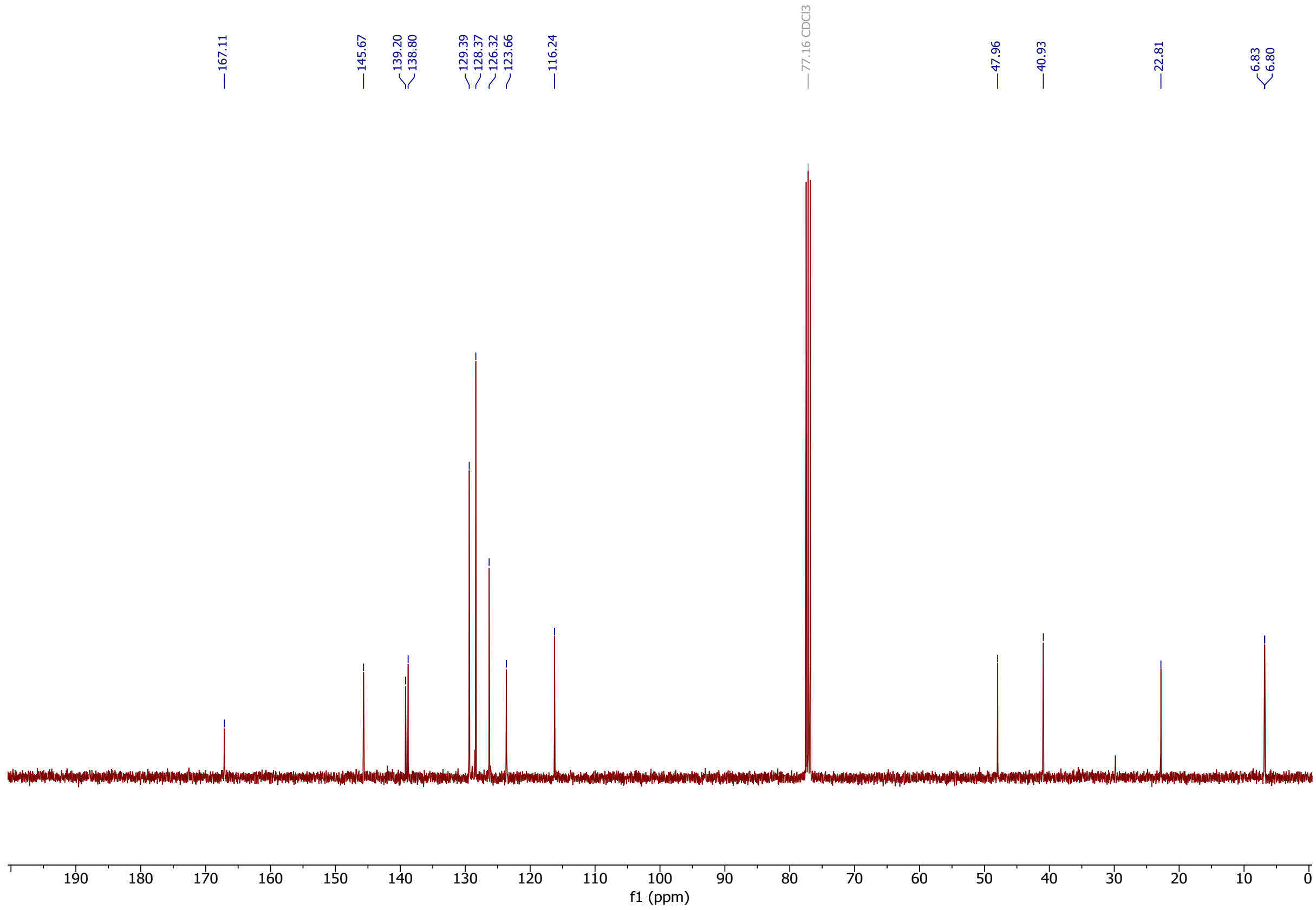

¹H NMR

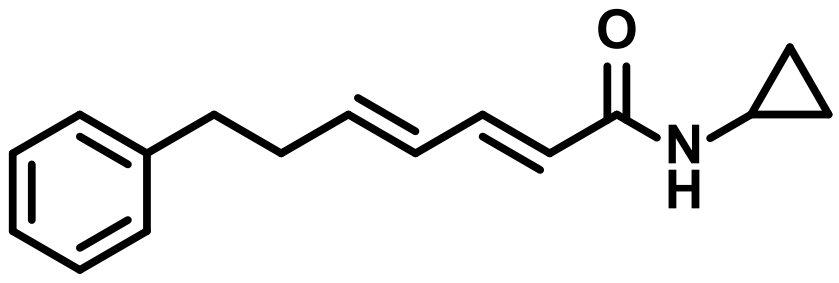

(2*E*,4*E*)-*N*-cyclopropyl-7-phenylhepta-2,4-dienamide

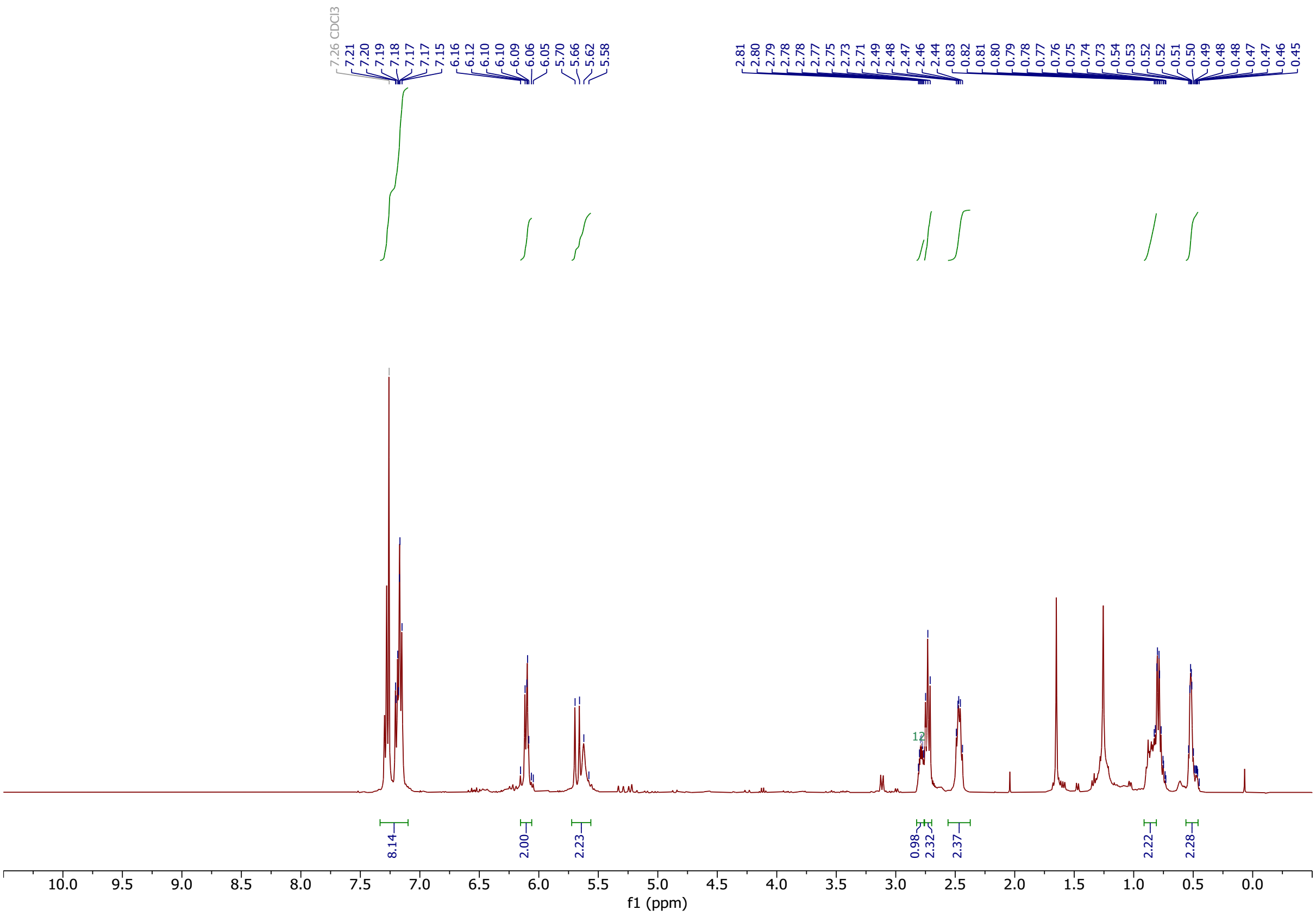

^{13}C NMR

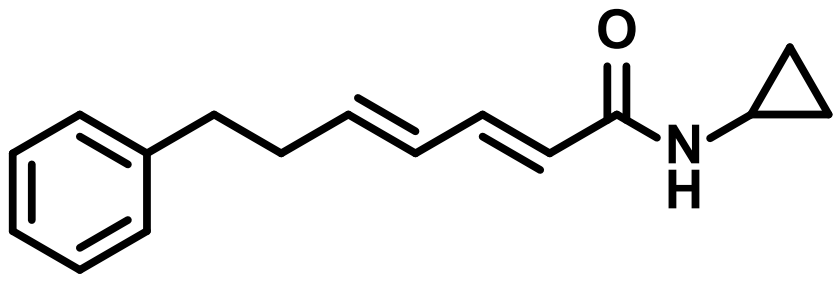

(2*E*,4*E*)-*N*-cyclopropyl-7-phenylhepta-2,4-dienamide

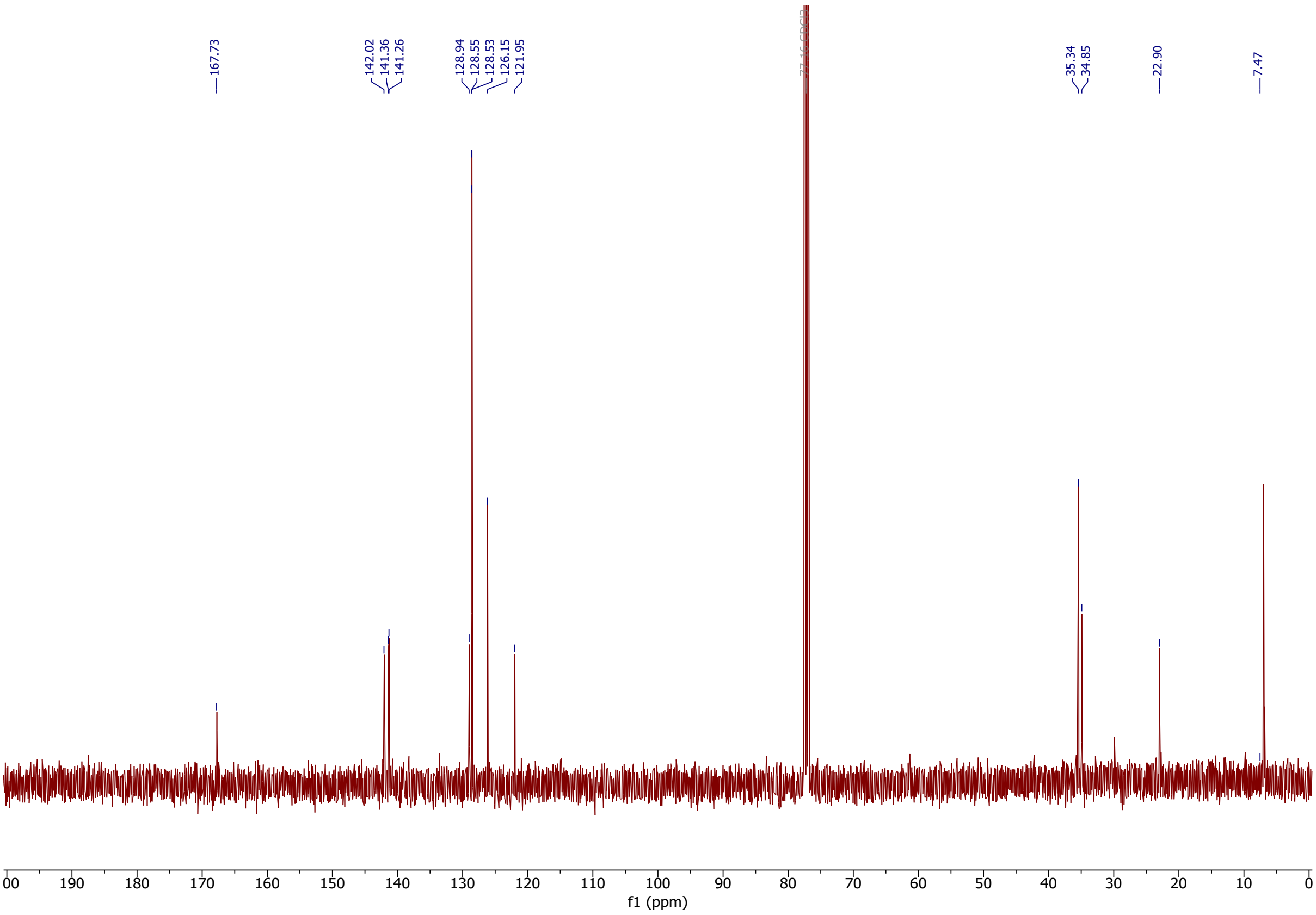

¹H NMR

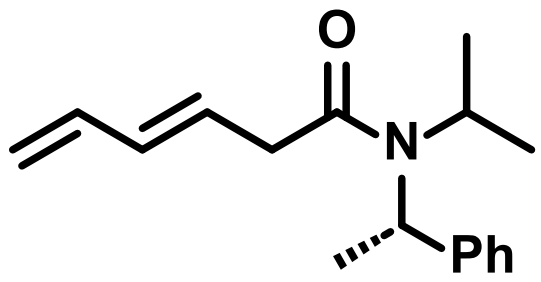

(*S*,*E*)-*N*-isopropyl-*N*-(1-phenylethyl)hexa-3,5-dienamide

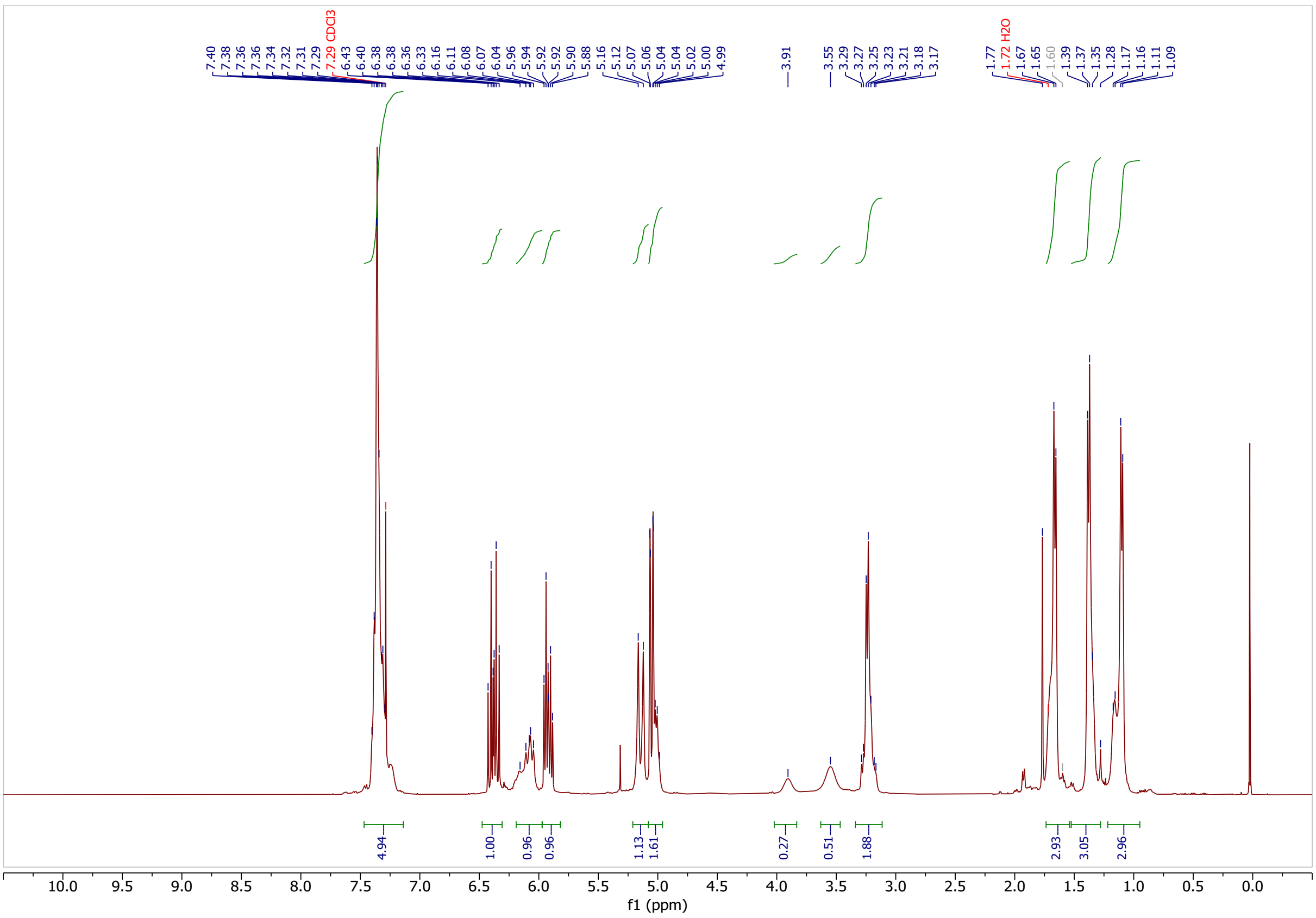

^{13}C NMR

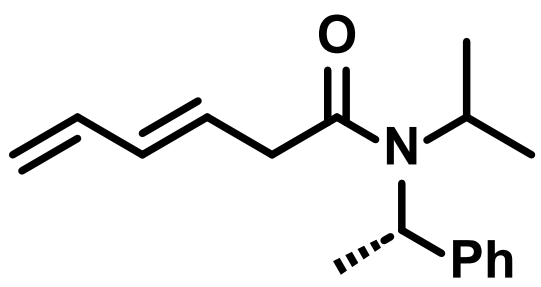

(*S*,*E*)-*N*-isopropyl-*N*-(1-phenylethyl)hexa-3,5-dienamide

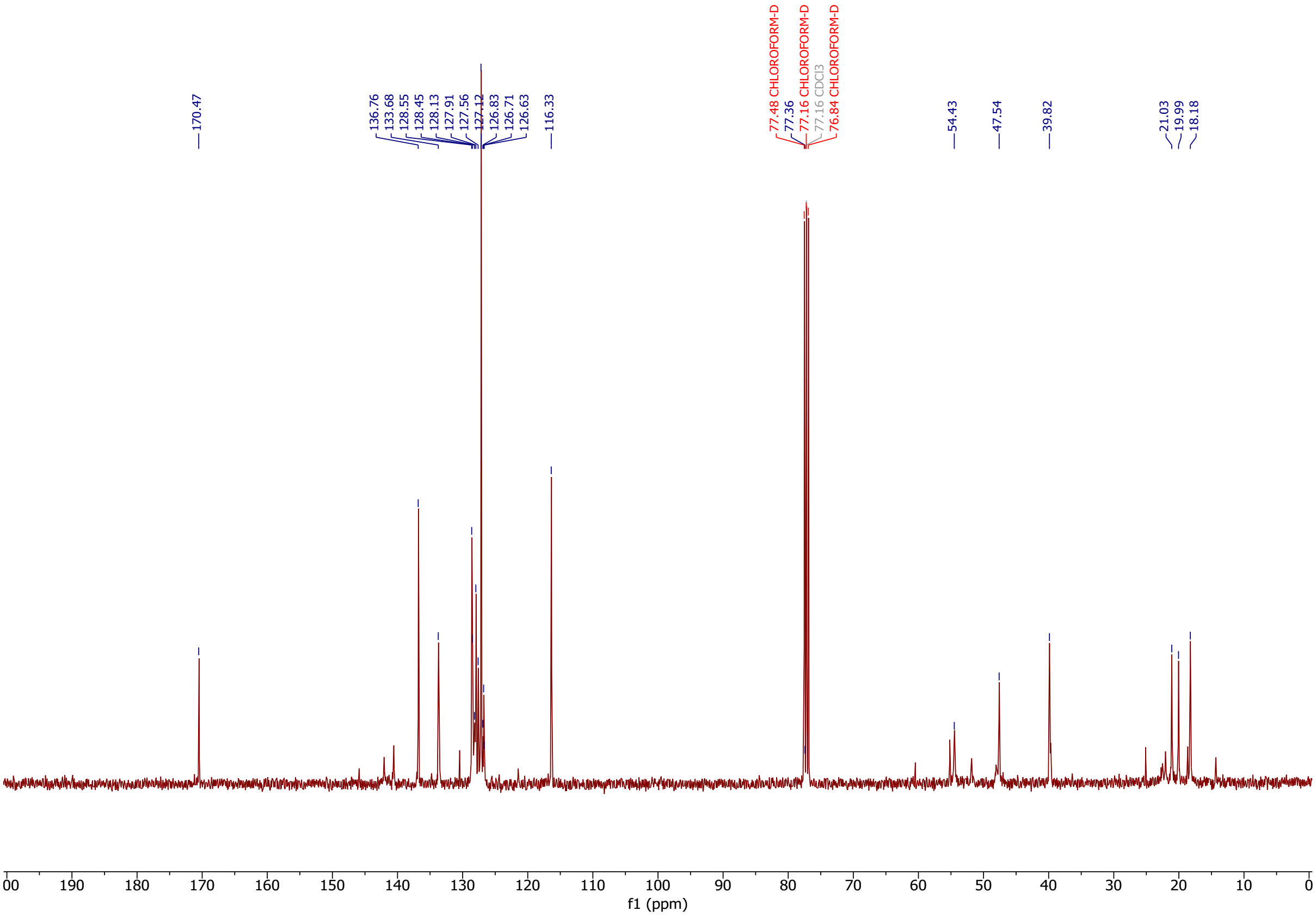

^{1}H NMR

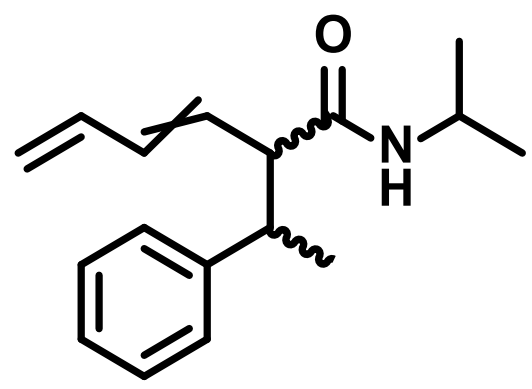

***N*-(*tert*-butyl)-2-(1-phenylethyl)hexa-3,5-dienamide**

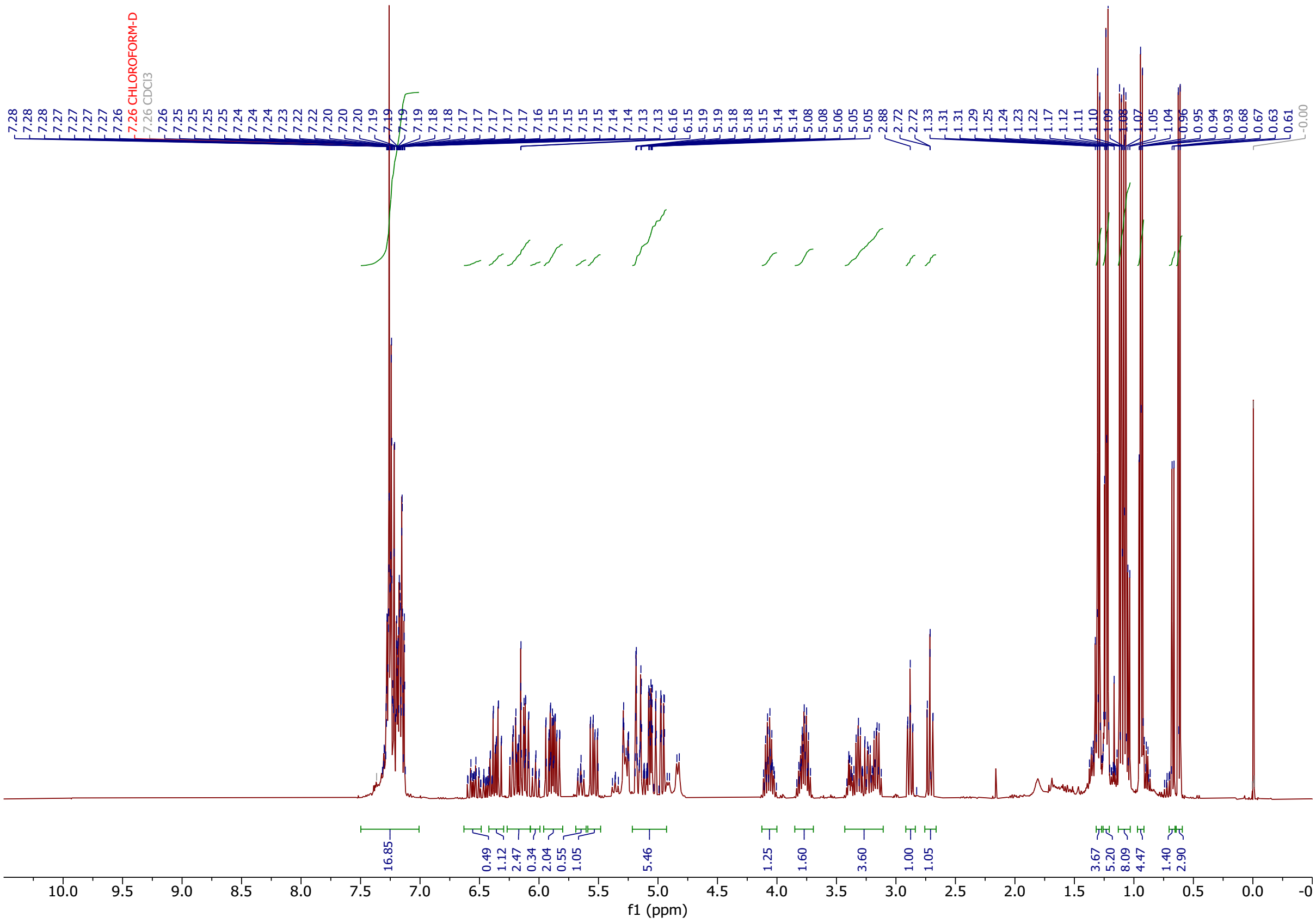

^{13}C NMR

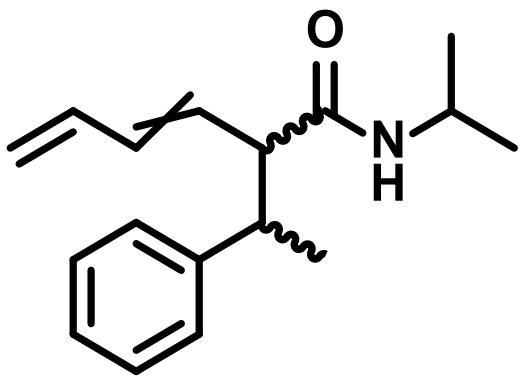

***N*-(*tert*-butyl)-2-(1-phenylethyl)hexa-3,5-dienamide**

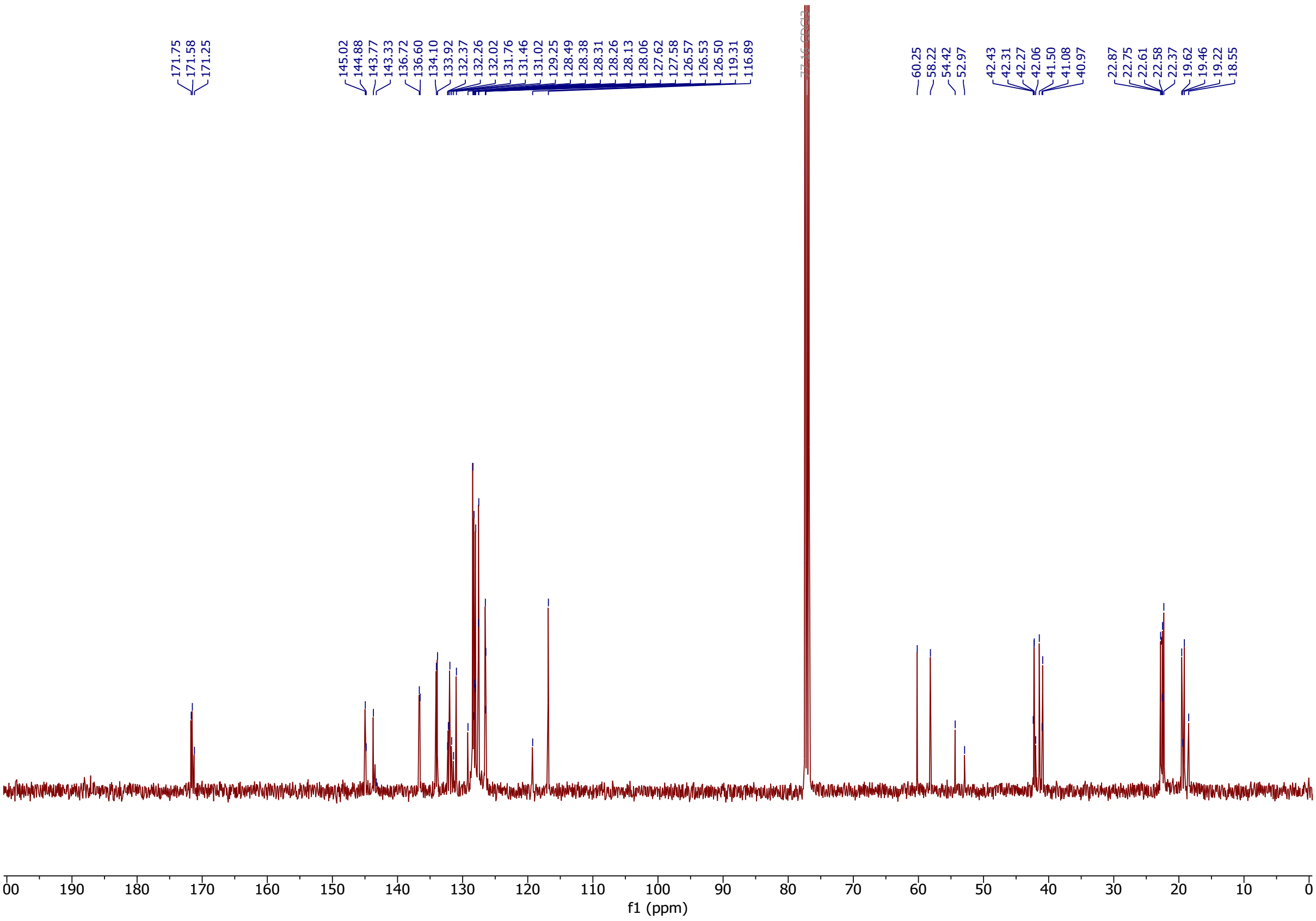

^{1}H NMR

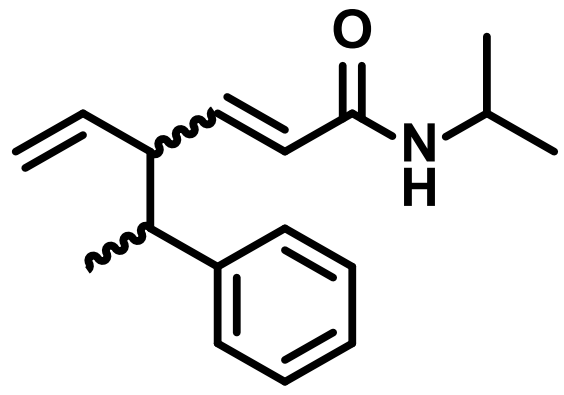

N-(tert-butyl)-4-(1-phenylethyl)hexa-2,5-dienamide

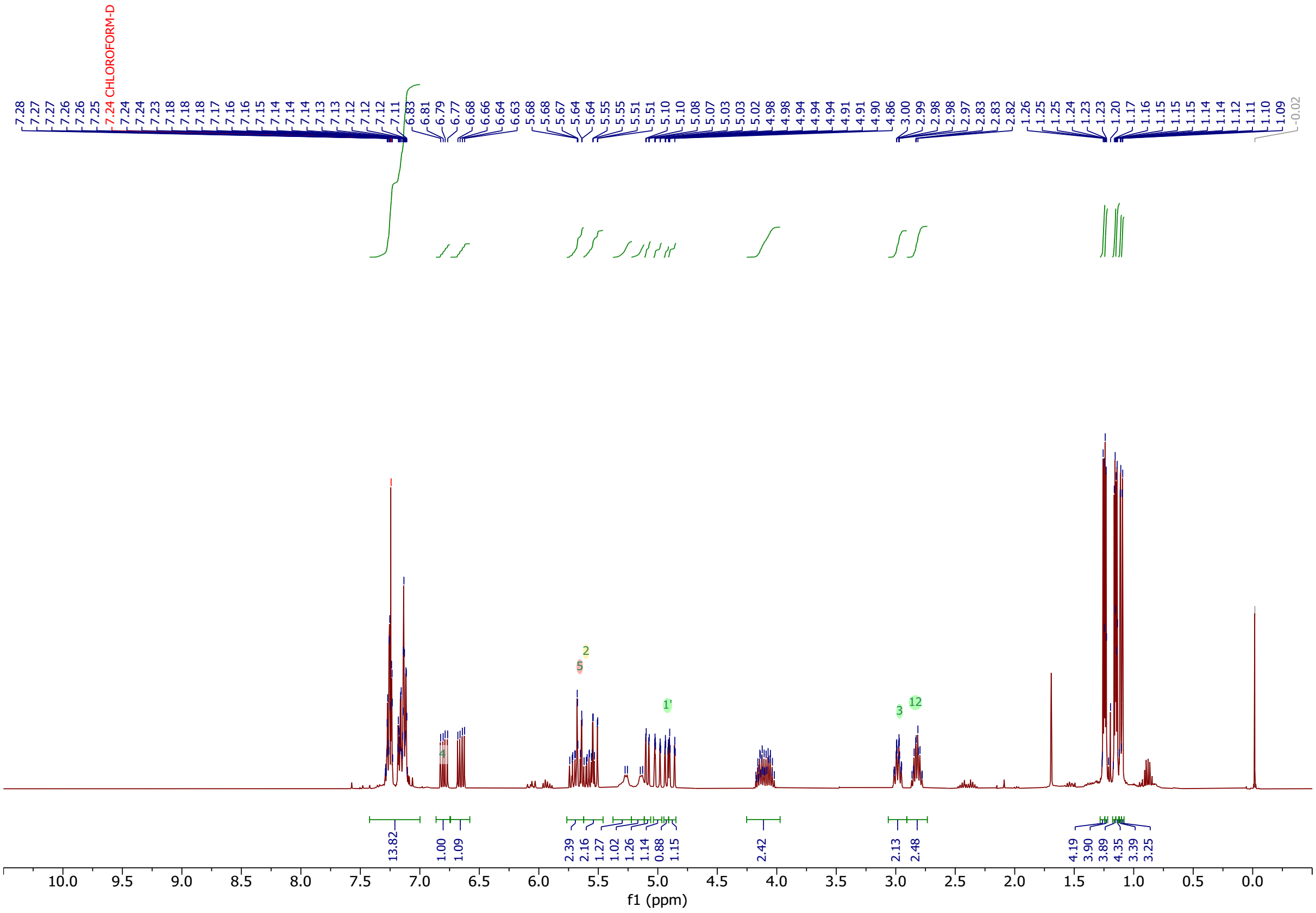

^{13}C NMR

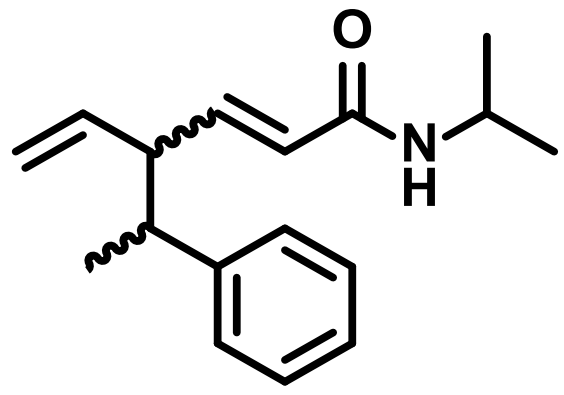

***N*-(*tert*-butyl)-4-(1-phenylethyl)hexa-2,5-dienamide**

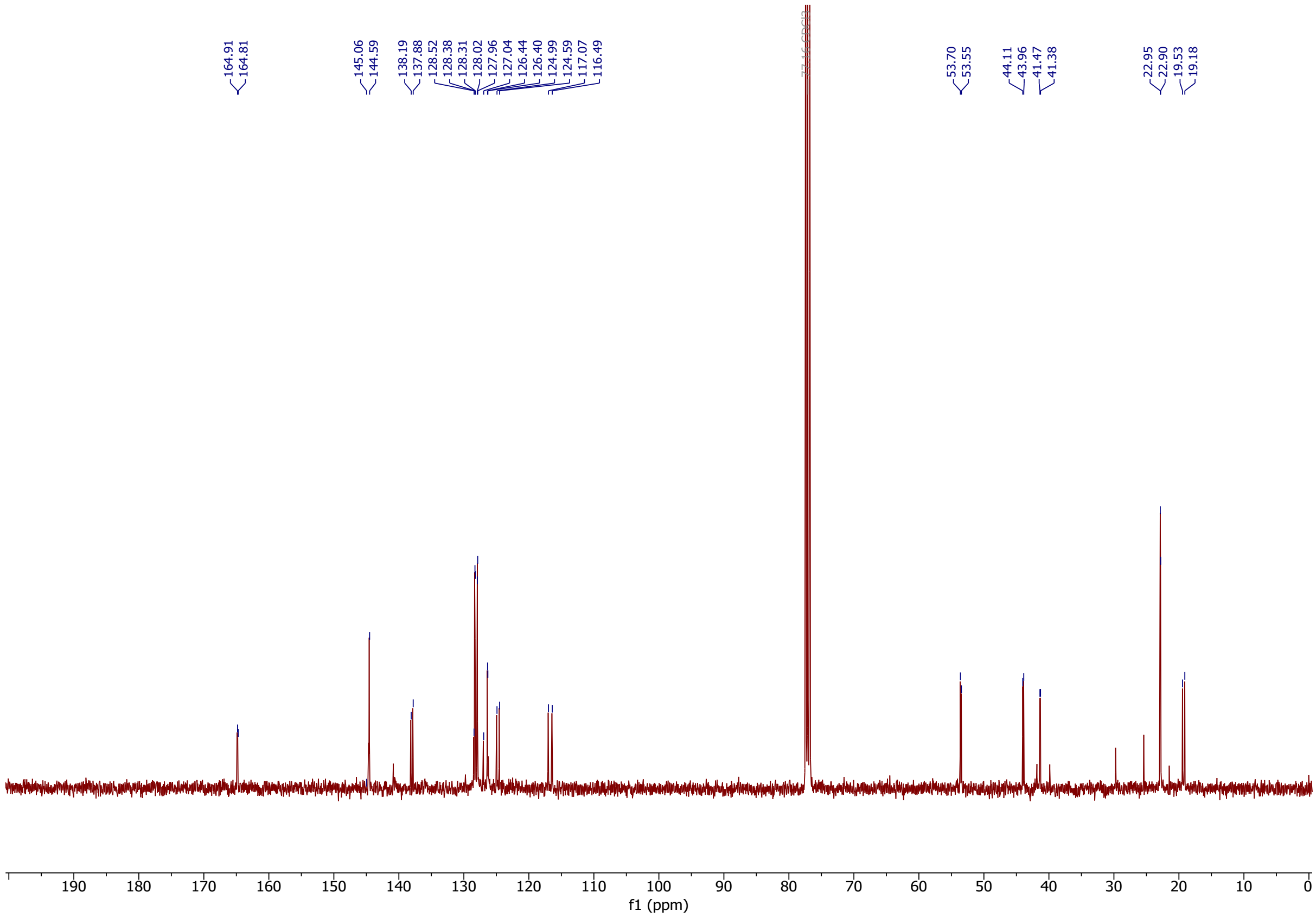

^{1}H NMR

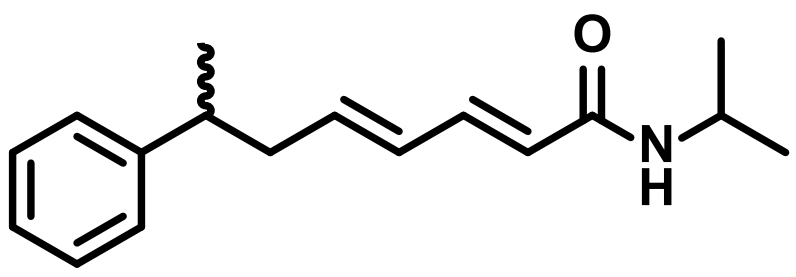

(2*E*,4*E*)-*N*-(isopropyl)-7-phenylocta-2,4-dienamide

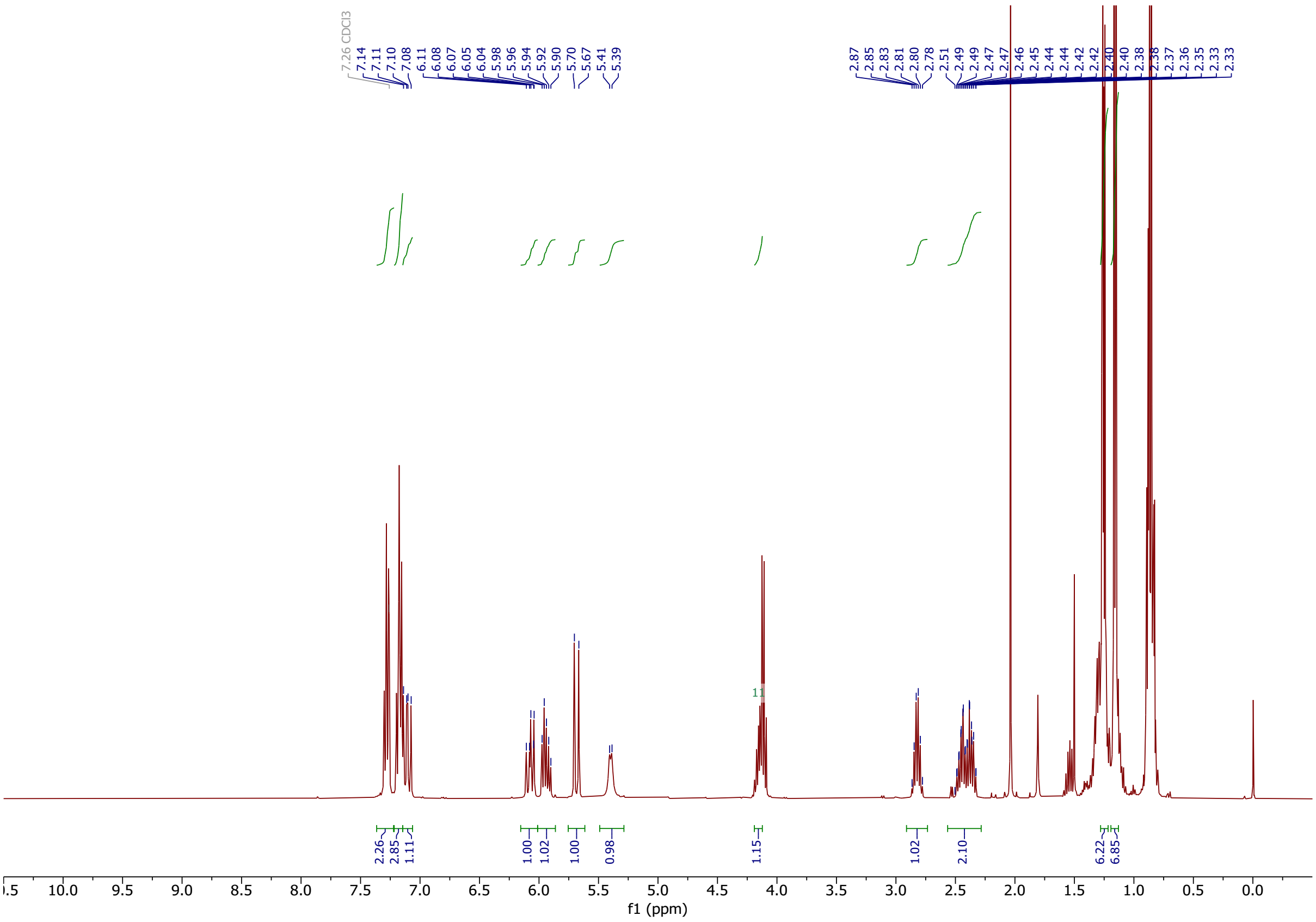

^{13}C NMR

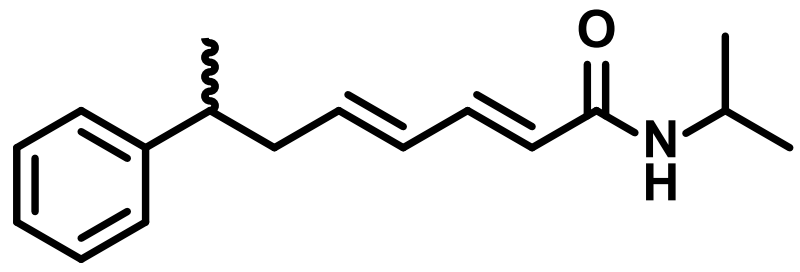

(2*E*,4*E*)-*N*-(isopropyl)-7-phenylocta-2,4-dienamide

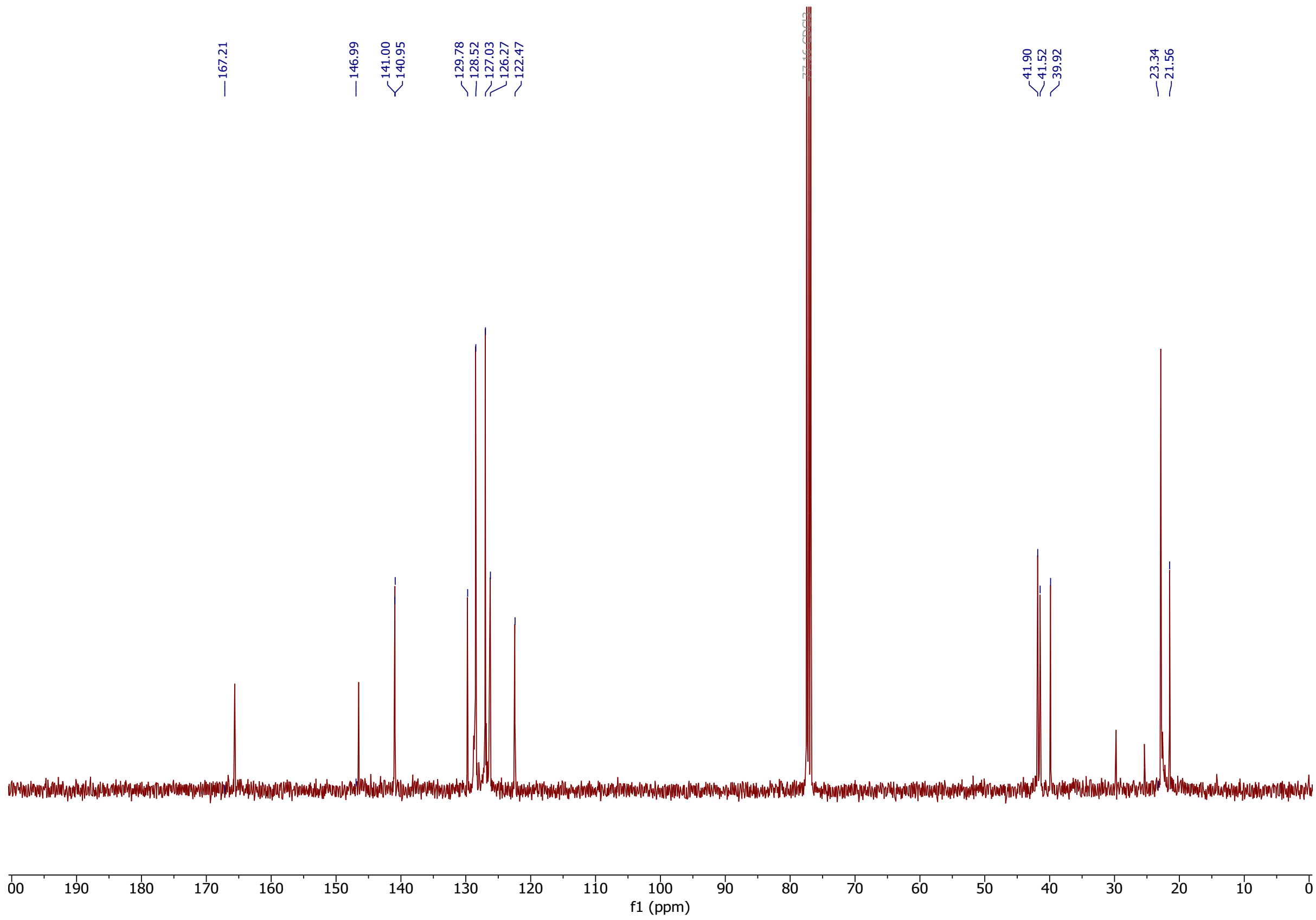

^{1}H NMR

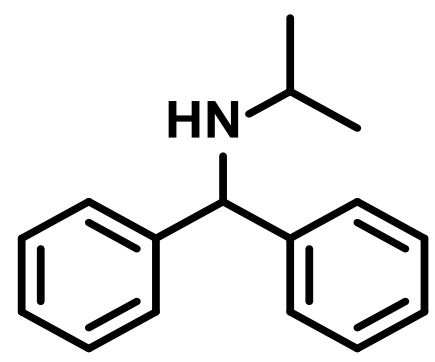

***N*-Benzhydrylpropan-2-amine**

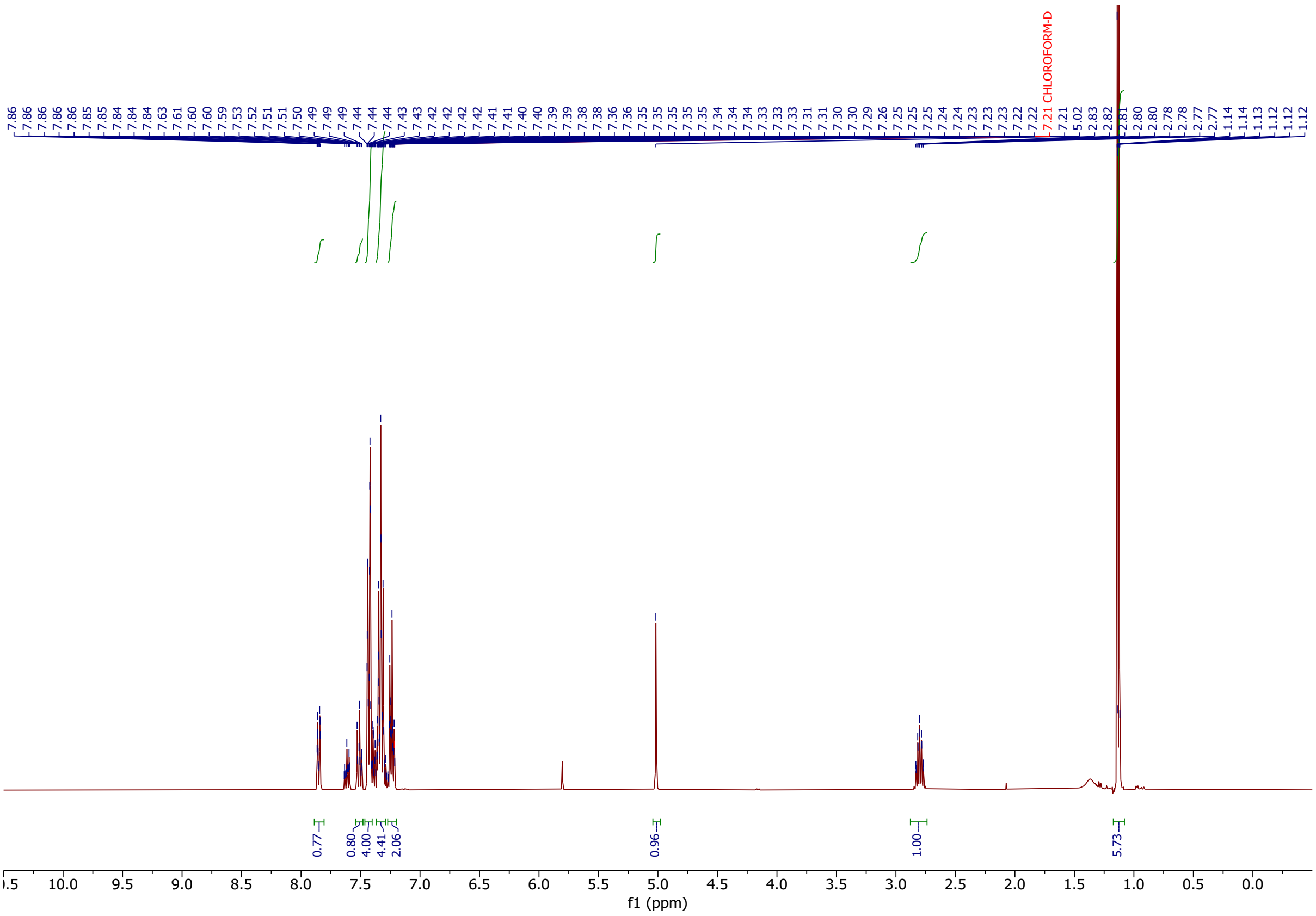

^{13}C NMR

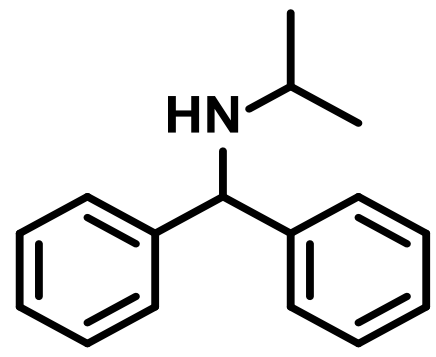

***N*-Benzhydrylpropan-2-amine**

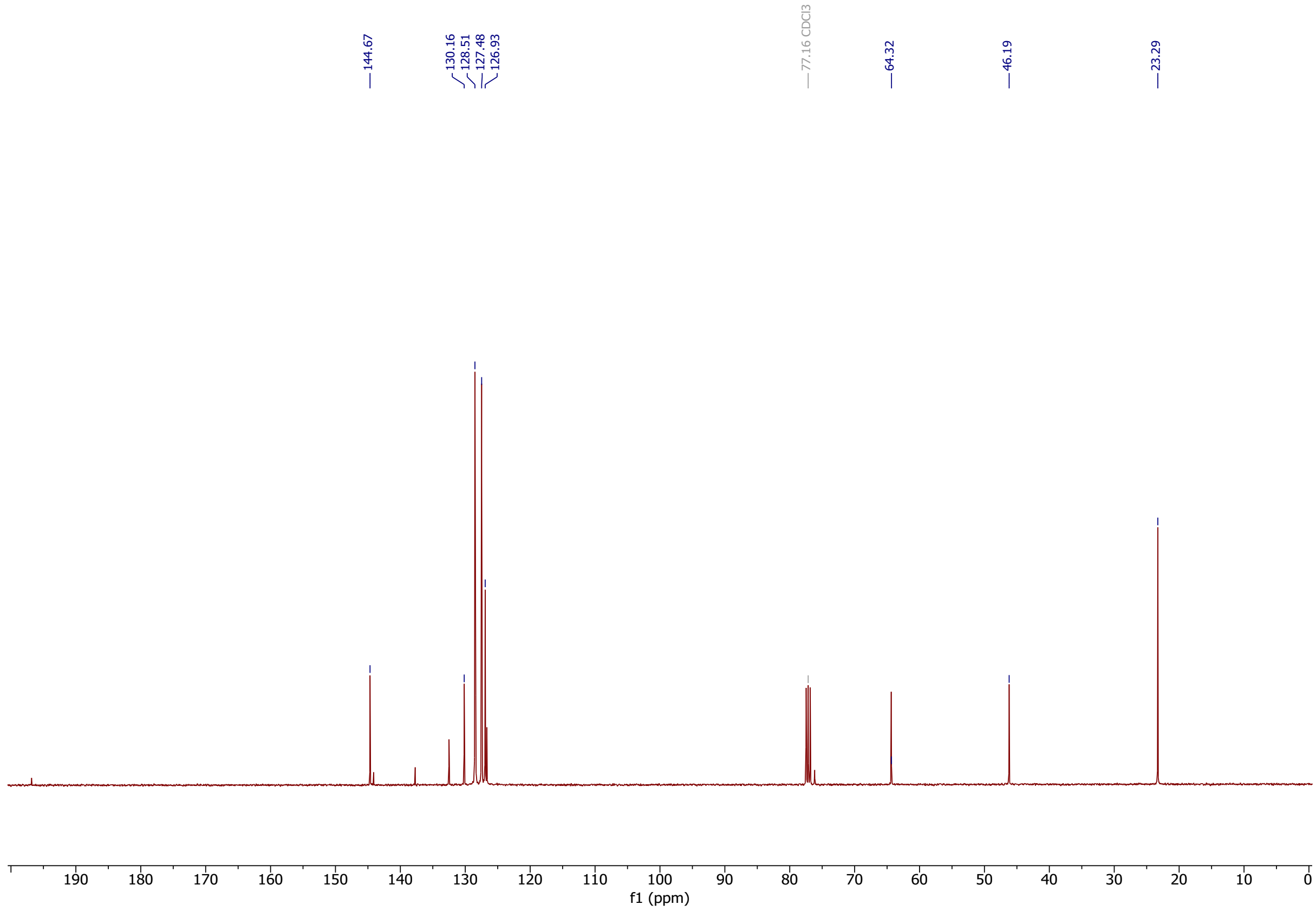

^{1}H NMR

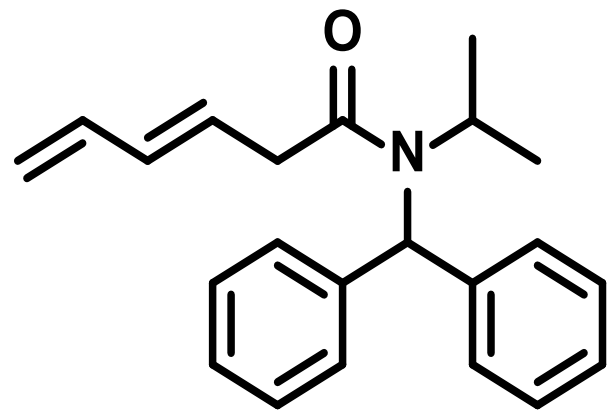

(*E*)-*N*-benzhydryl-*N*-isopropylhexa-3,5-dienamide

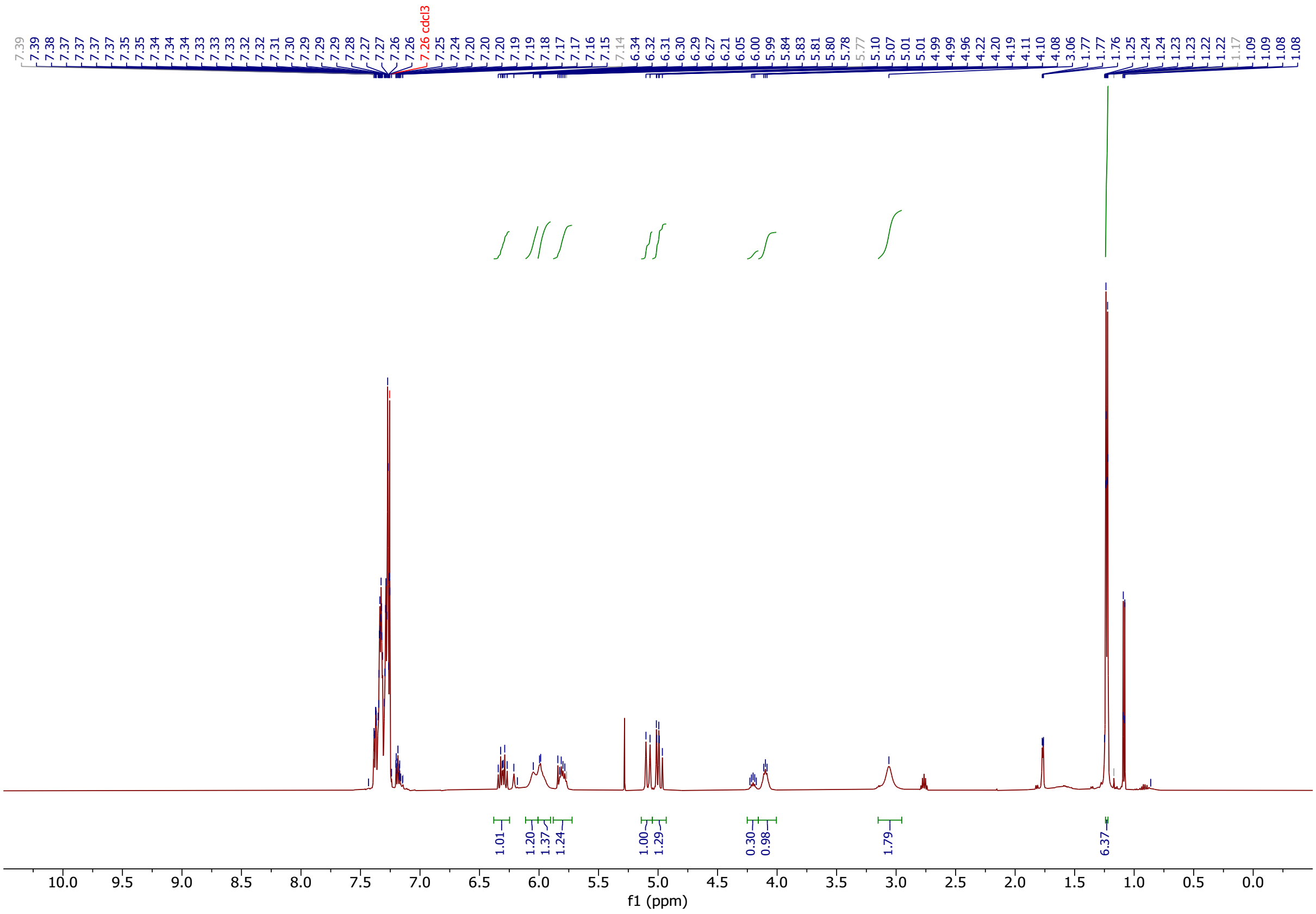

^{13}C NMR

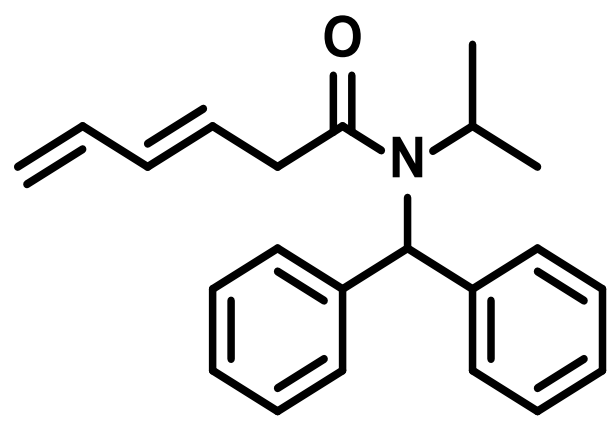

(*E*)-*N*-benzhydryl-*N*-isopropylhexa-3,5-dienamide

^{1}H NMR

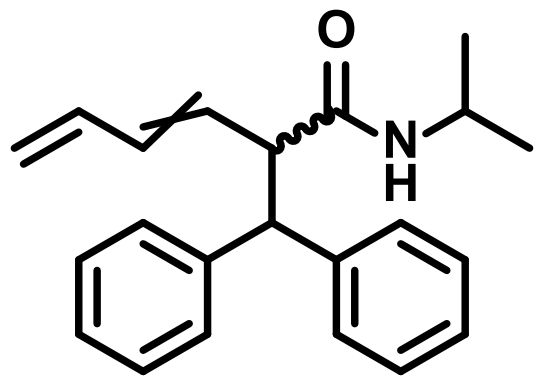

(*E*)-2-benzhydryl-*N*-isopropylhexa-3,5-dienamide

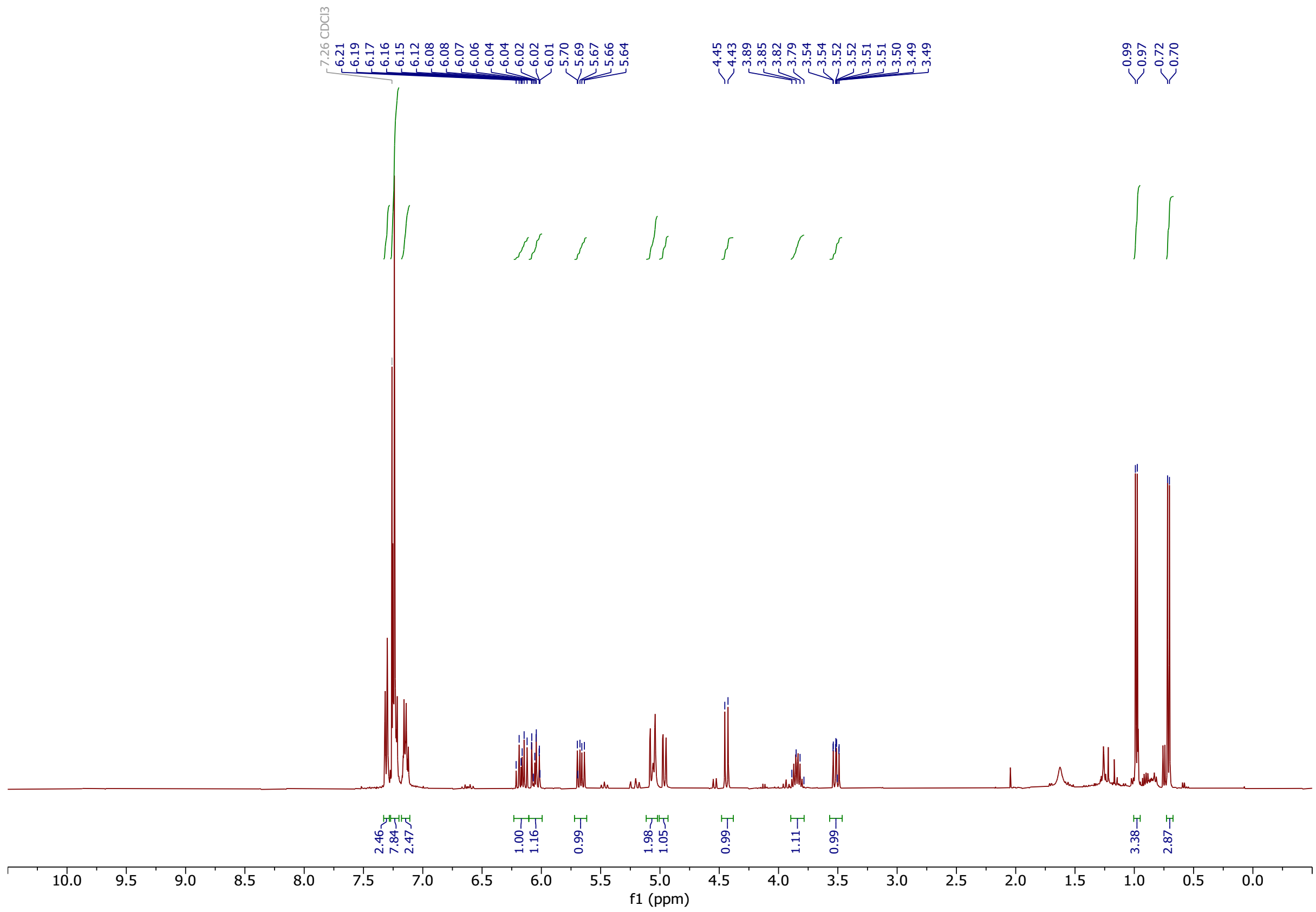

^{13}C NMR

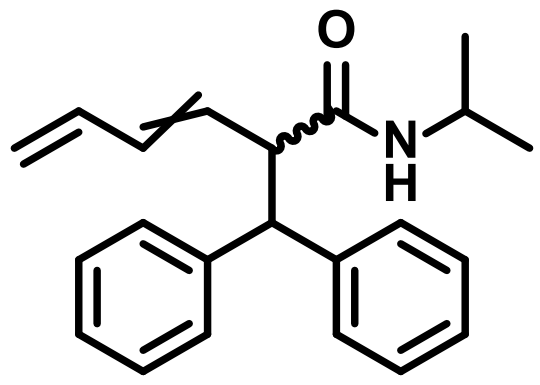

(*E*)-2-benzhydryl-*N*-isopropylhexa-3,5-dienamide

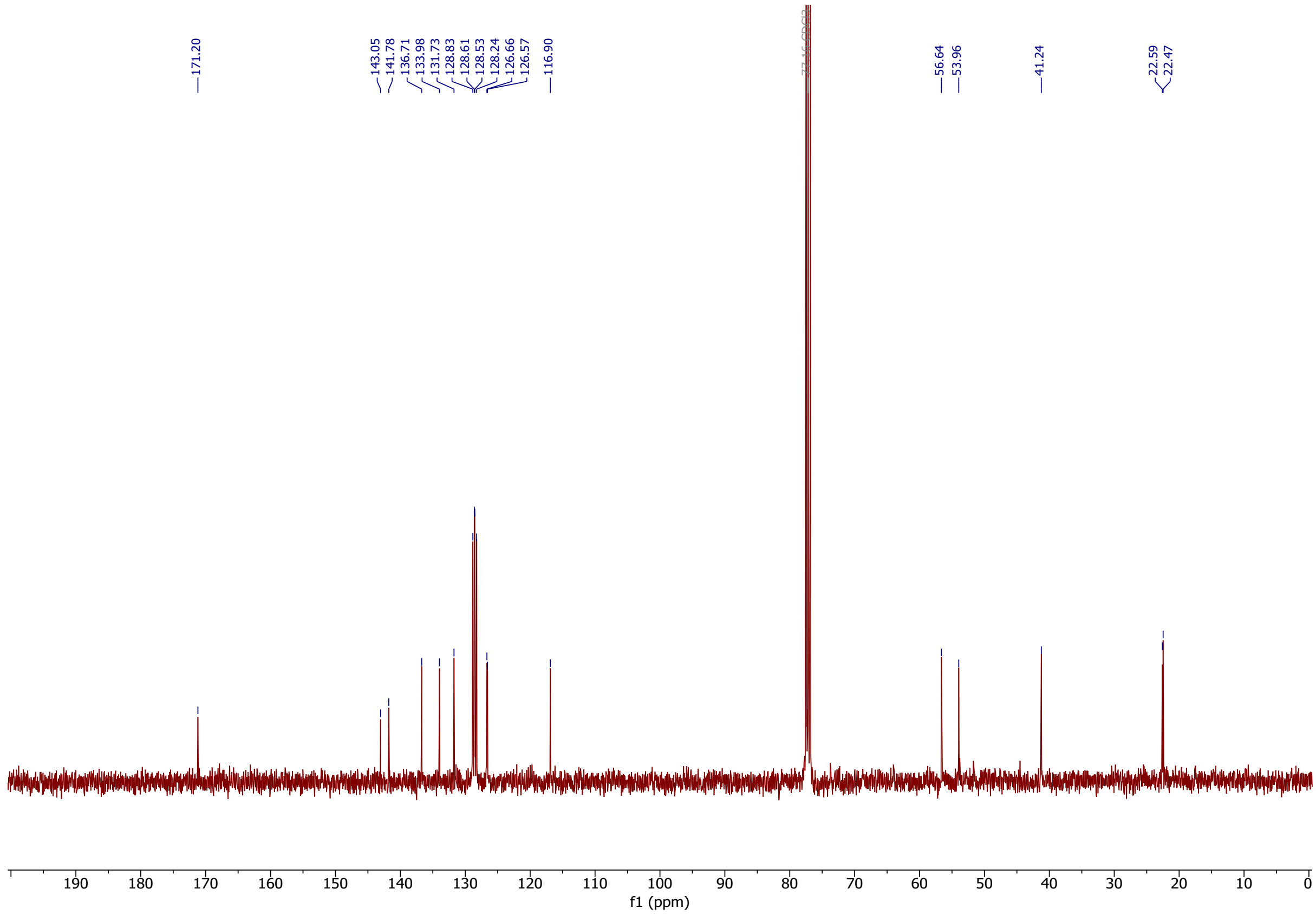

^{1}H NMR

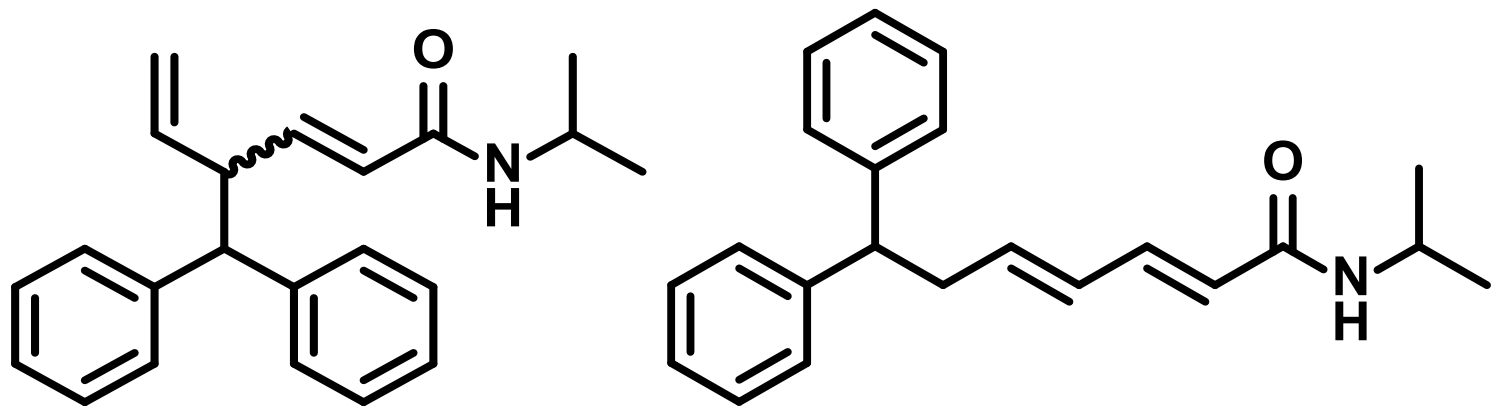

4-Benzhydryl-*N*-isopropylhexa-2,5-dienamide & (2*E*,4*E*)-*N*-isopropyl-7,7-diphenylhepta-2,4-dienamide

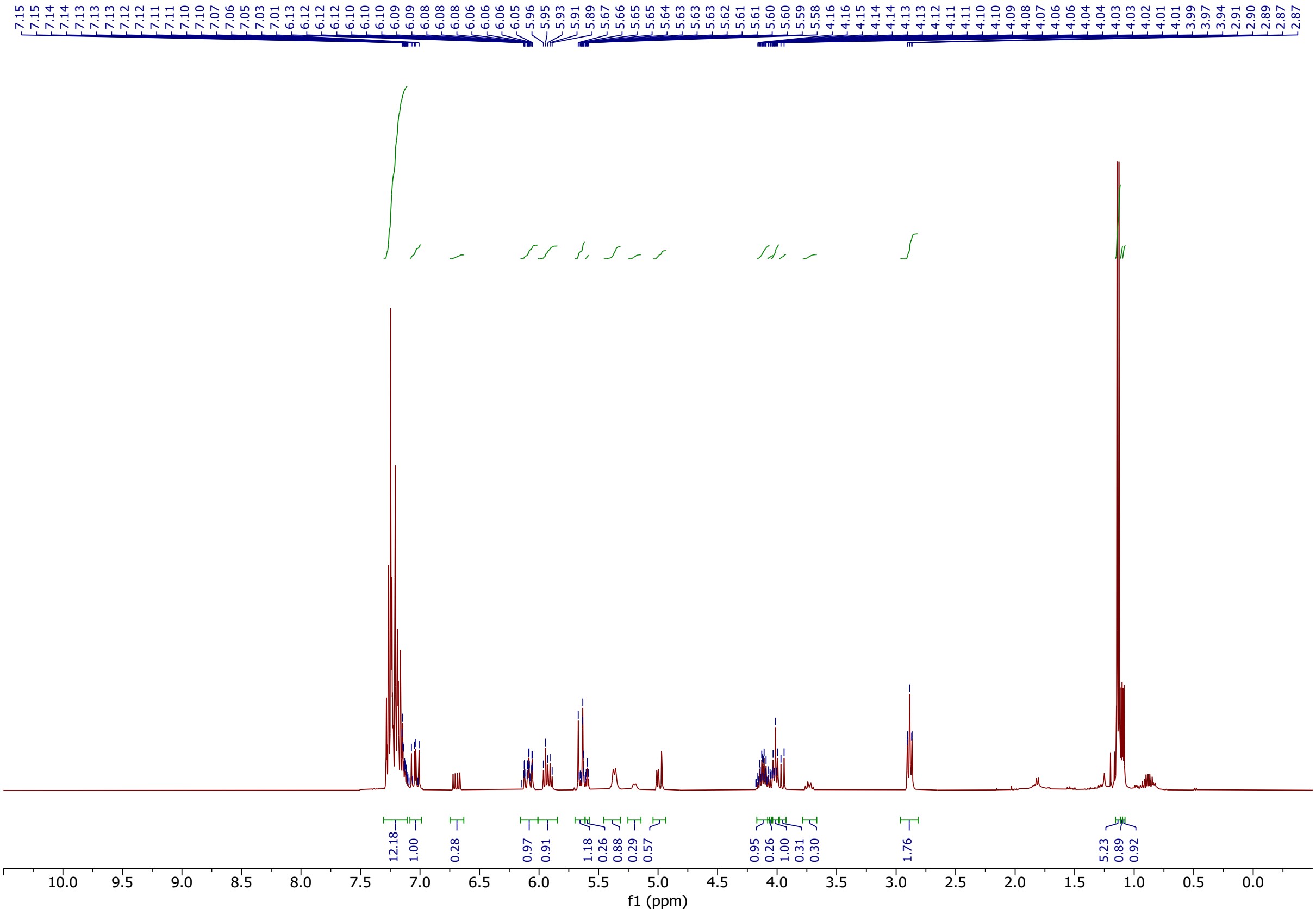

^{13}C NMR

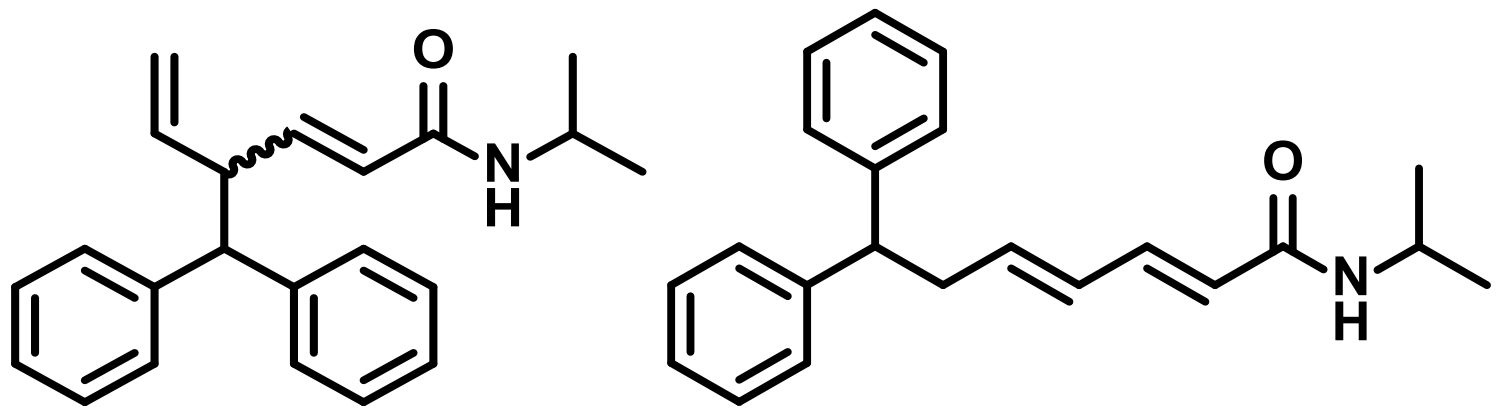

4-Benzhydryl-*N*-isopropylhexa-2,5-dienamide & (2*E*,4*E*)-*N*-isopropyl-7,7-diphenylhepta-2,4-dienamide

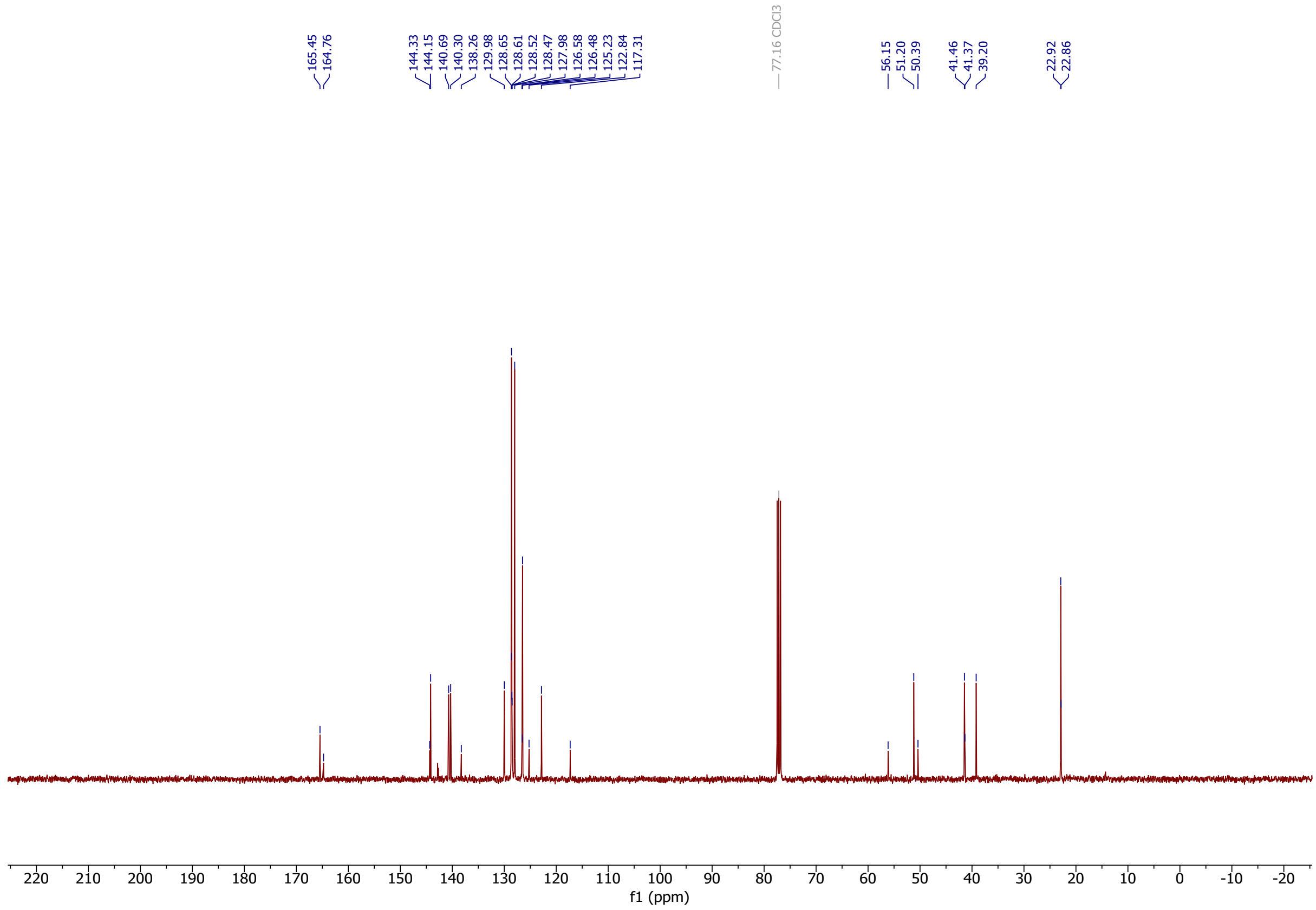

^{1}H NMR

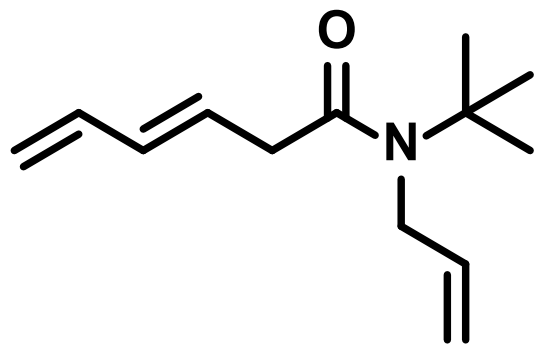

(*E*)-*N*-allyl-*N*-(*tert*-butyl)hexa-3,5-dienamide

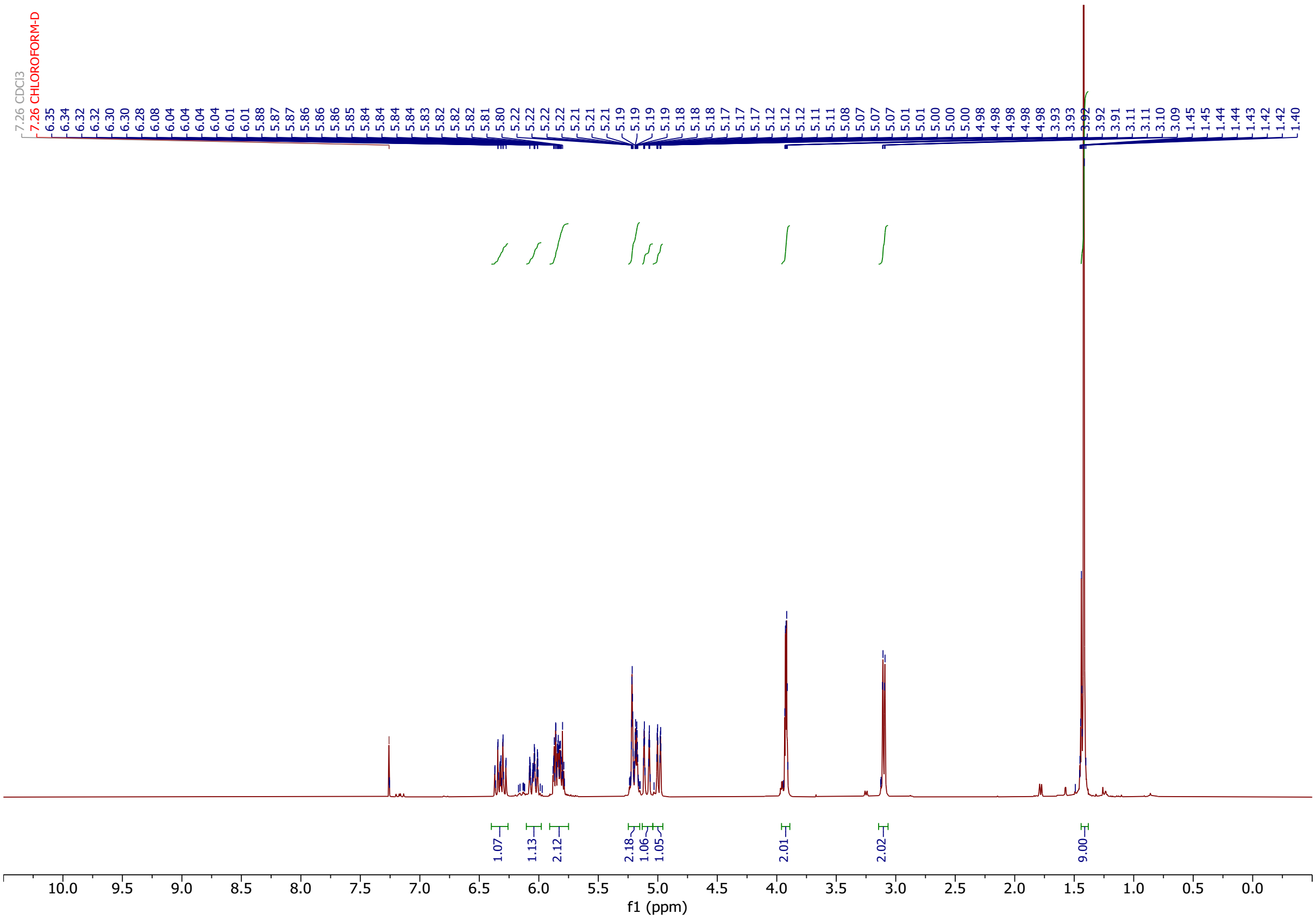

^{13}C NMR

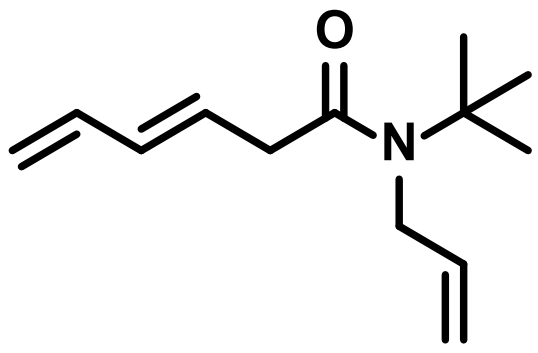

(*E*)-*N*-allyl-*N*-(*tert*-butyl)hexa-3,5-dienamide

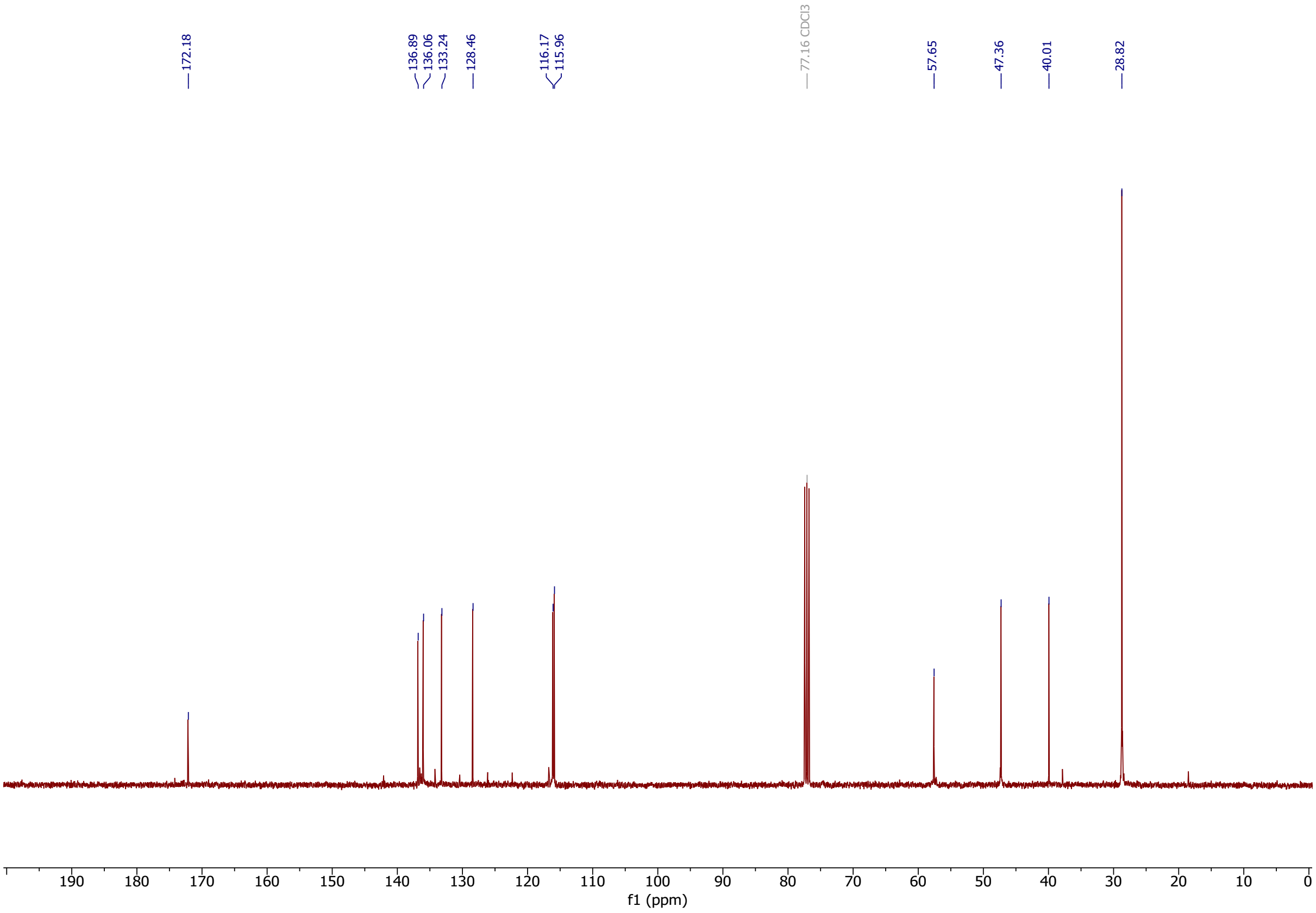

^{1}H NMR

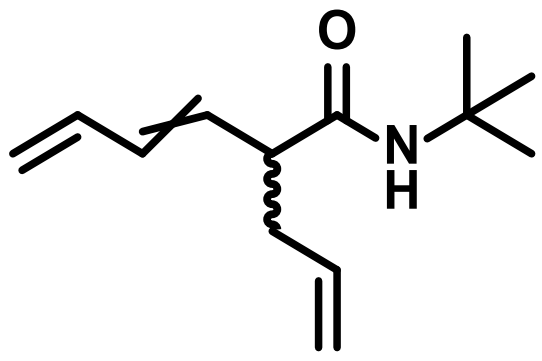

(*E*)-2-allyl-*N*-(*tert*-butyl)hexa-3,5-dienamide

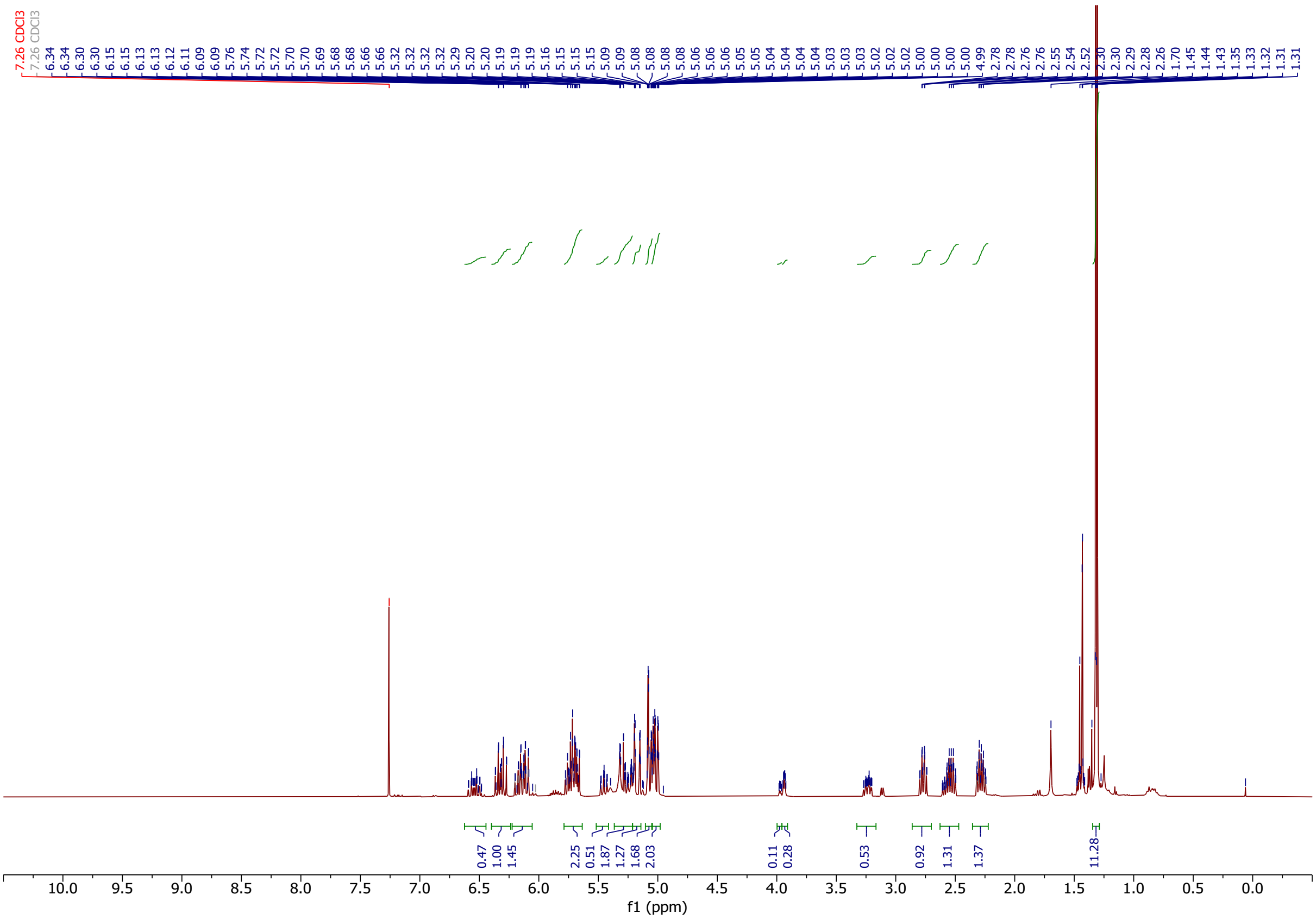

^{13}C NMR

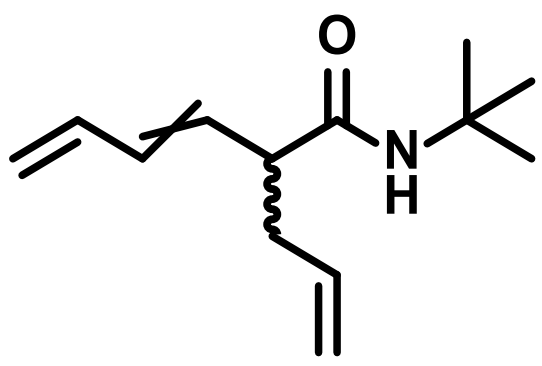

(*E*)-2-allyl-*N*-(*tert*-butyl)hexa-3,5-dienamide

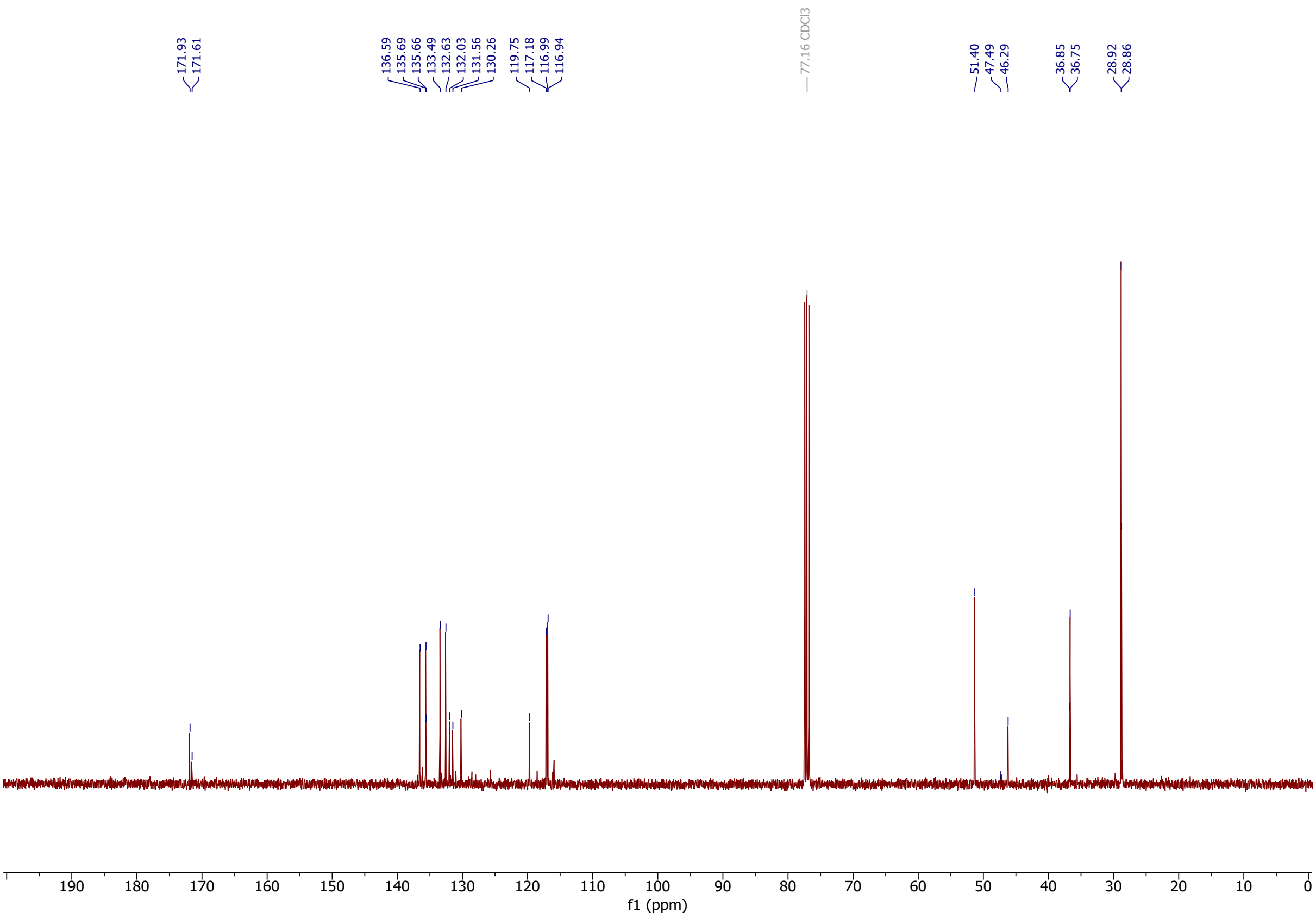

^{1}H NMR

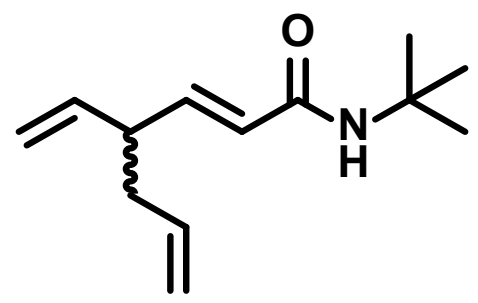

(*E*)-*N*-(*tert*-butyl)-4-vinylhepta-2,6-dienamide

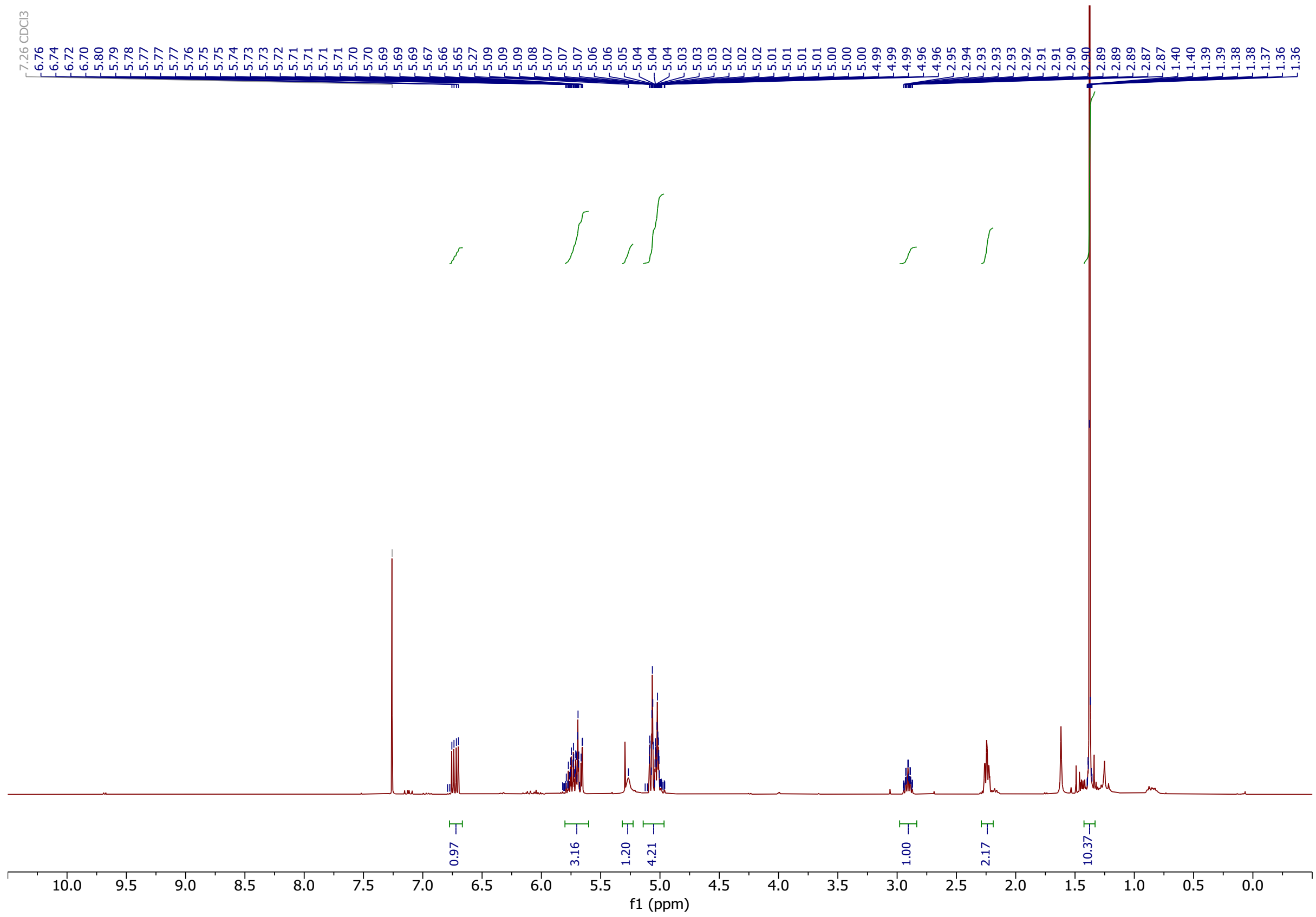

^{13}C NMR

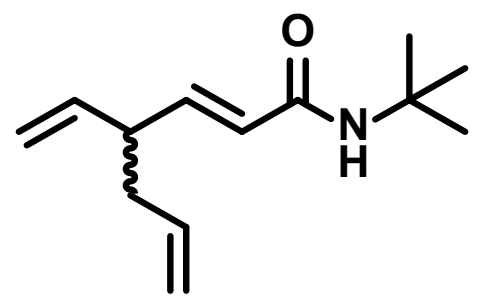

(*E*)-*N*-(*tert*-butyl)-4-vinylhepta-2,6-dienamide

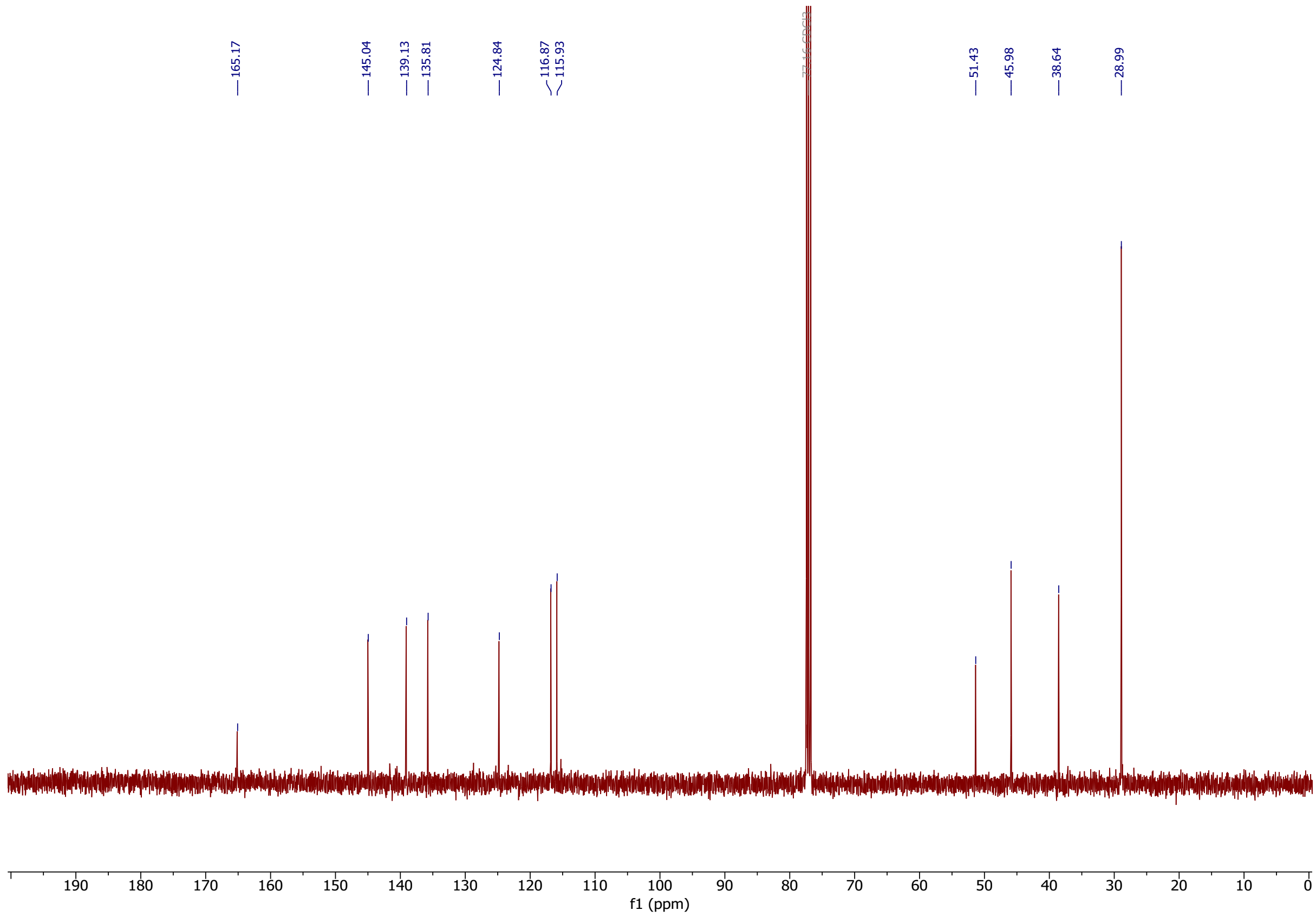

^{1}H NMR

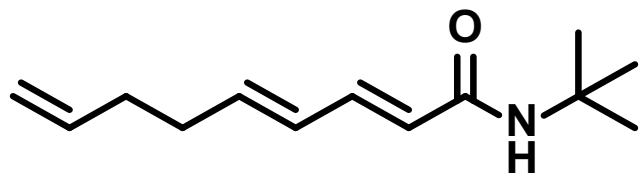

(2*E*,4*E*)-*N*-(*tert*-butyl)nona-2,4,8-trienamide

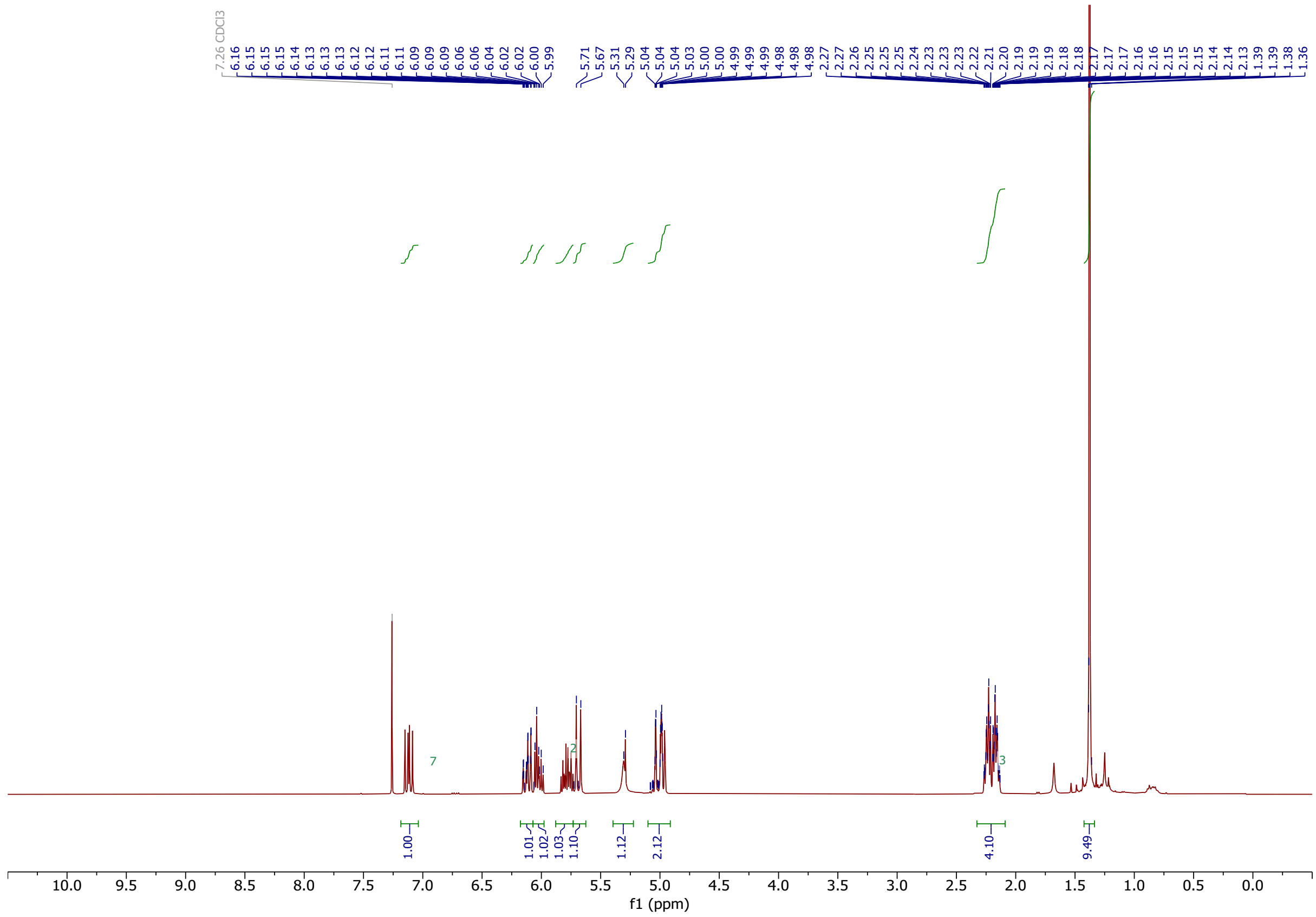

^{13}C NMR

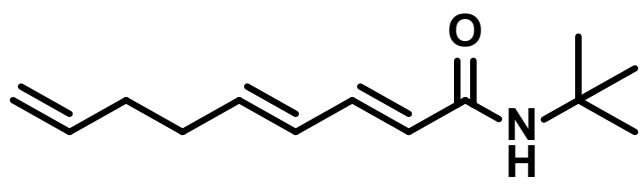

(2*E*,4*E*)-*N*-(*tert*-butyl)nona-2,4,8-trienamide

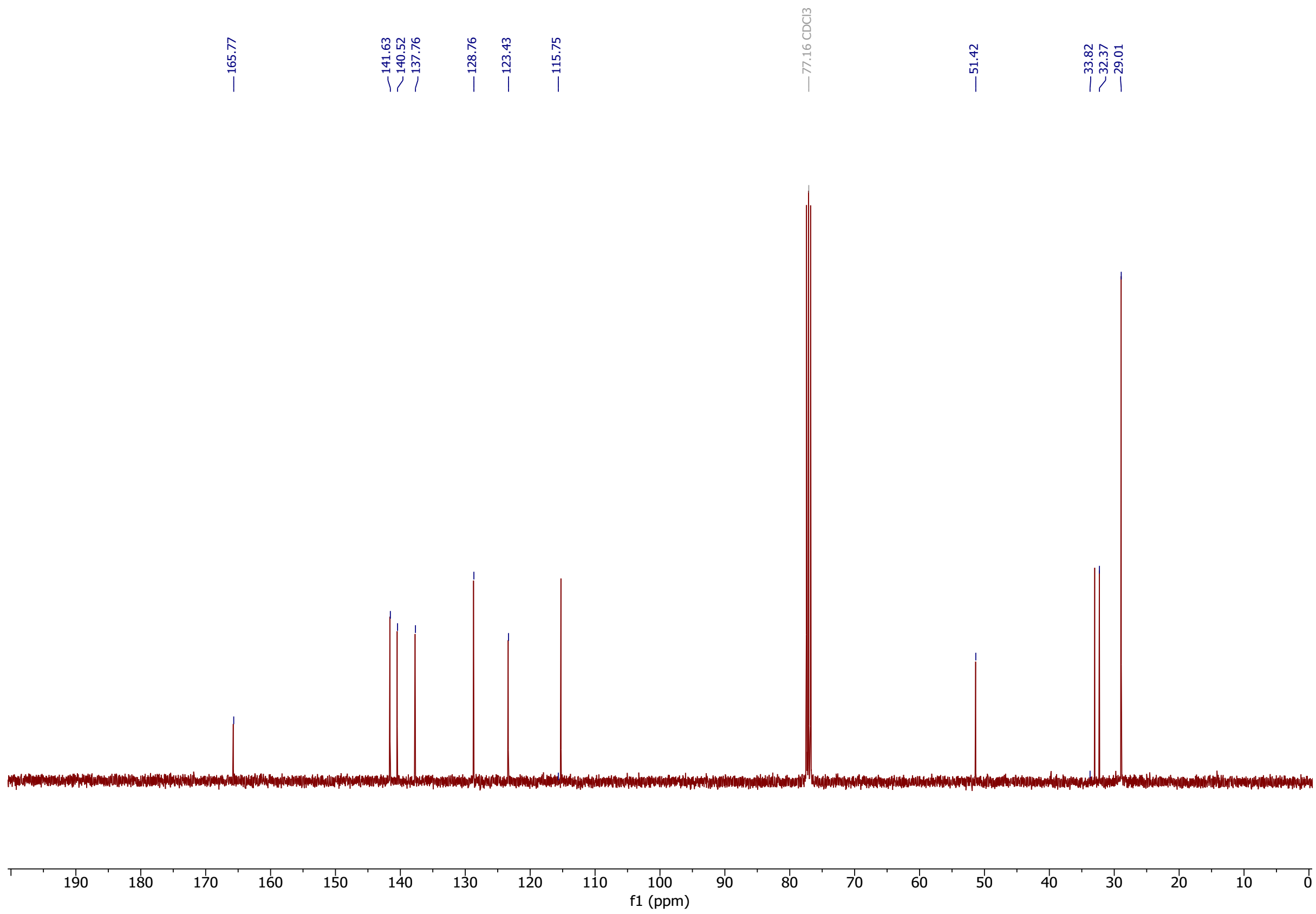

^{1}H NMR

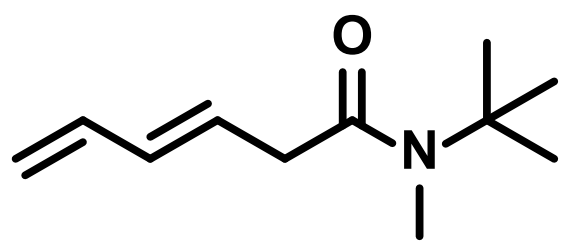

(*E*)-*N*-(*tert*-butyl)-*N*-methylhexa-3,5-dienamide

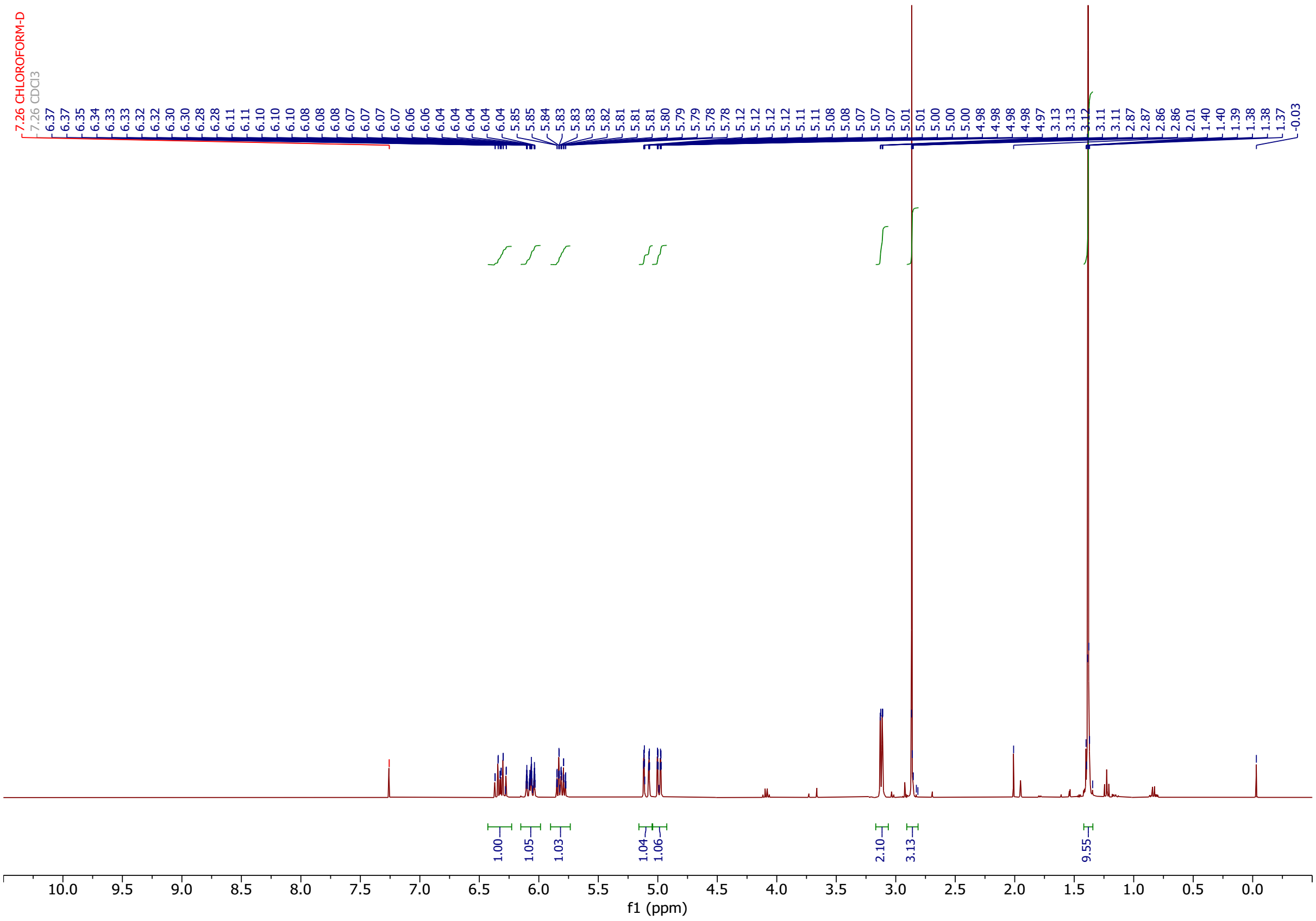

^{13}C NMR

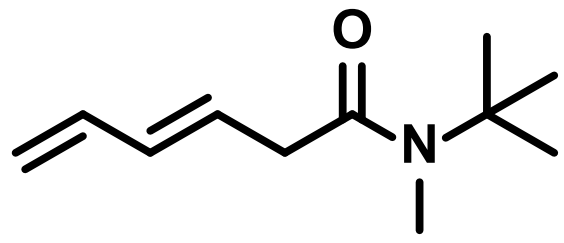

(*E*)-*N*-(*tert*-butyl)-*N*-methylhexa-3,5-dienamide

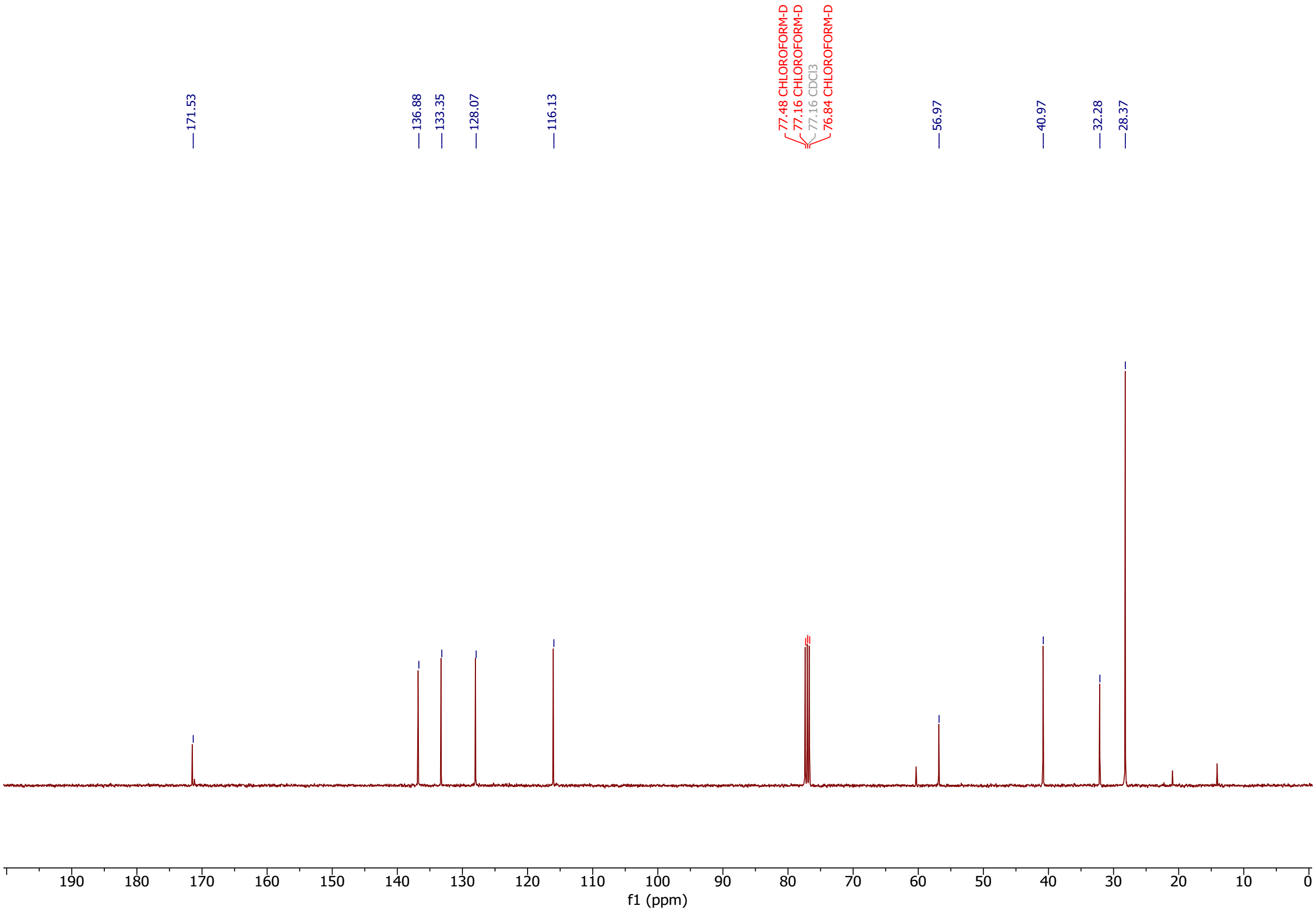

^{1}H NMR

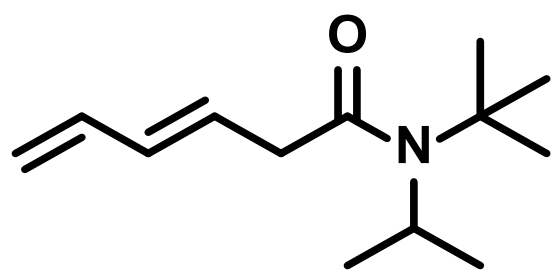

(*E*)-*N*-(*tert*-butyl)-*N*-isopropylhexa-3,5-dienamide

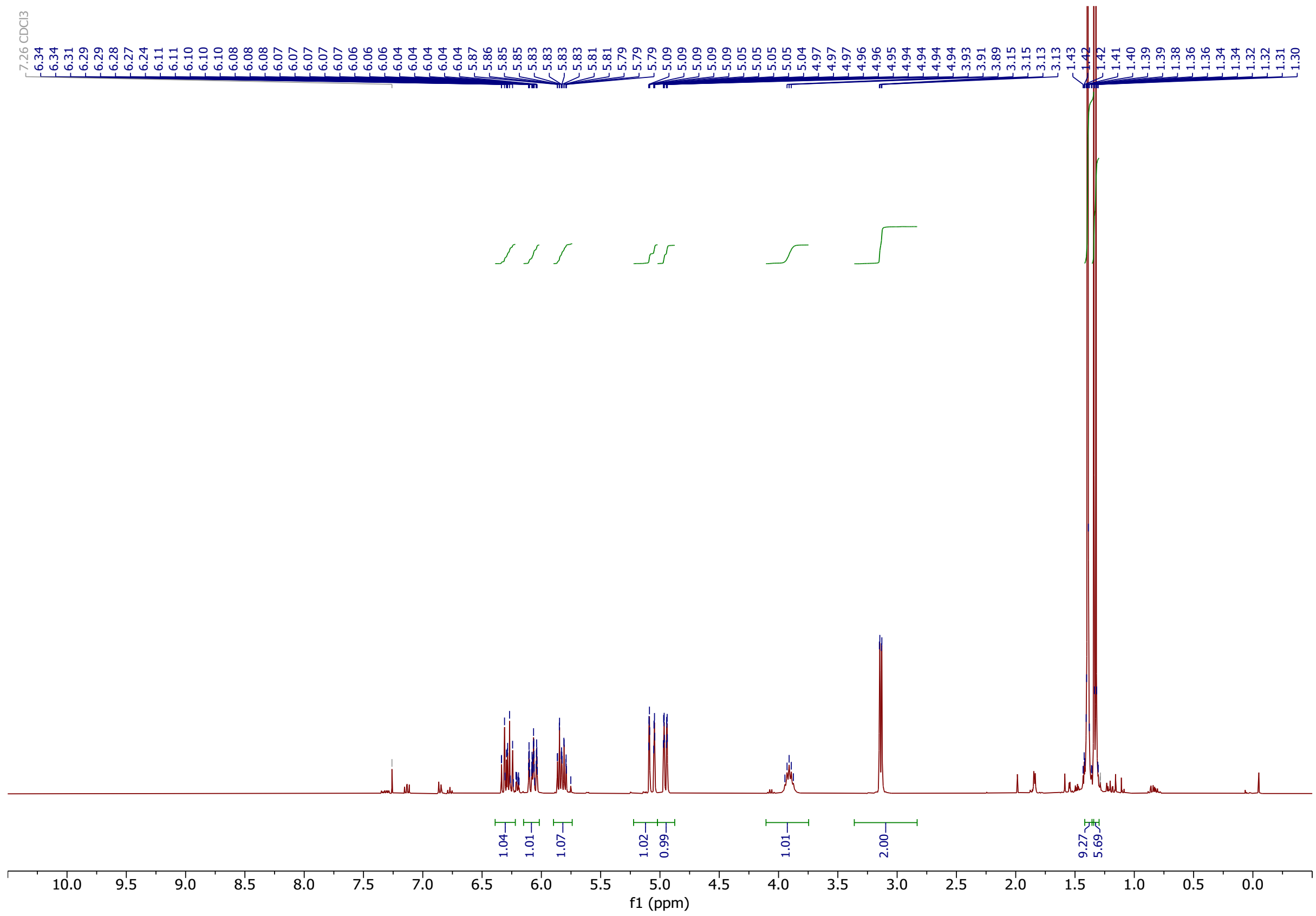

^{13}C NMR

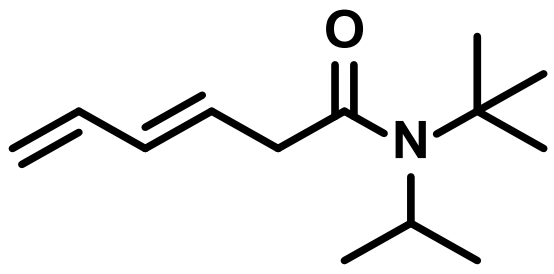

(*E*)-*N*-(*tert*-butyl)-*N*-isopropylhexa-3,5-dienamide

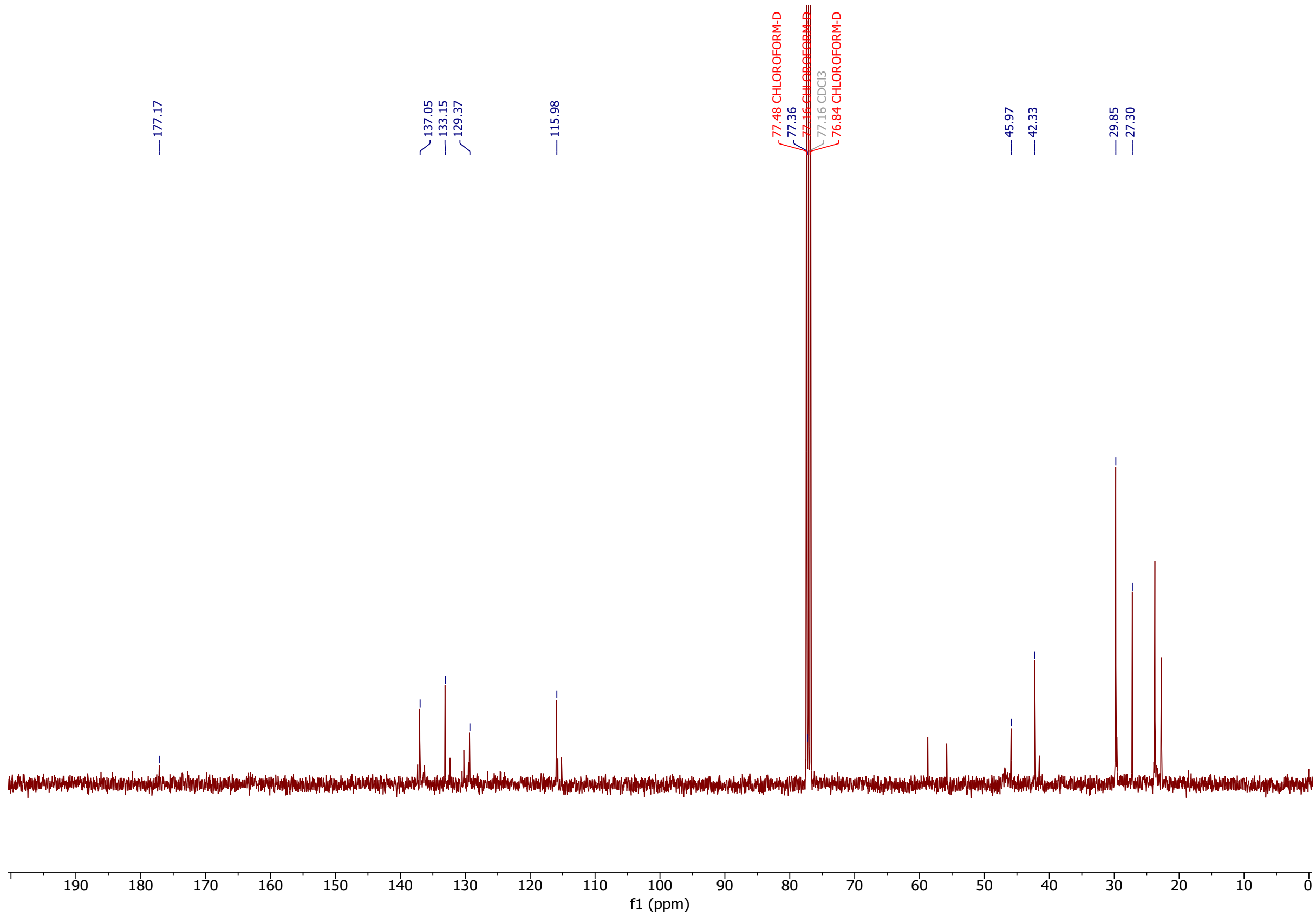

^{1}H NMR

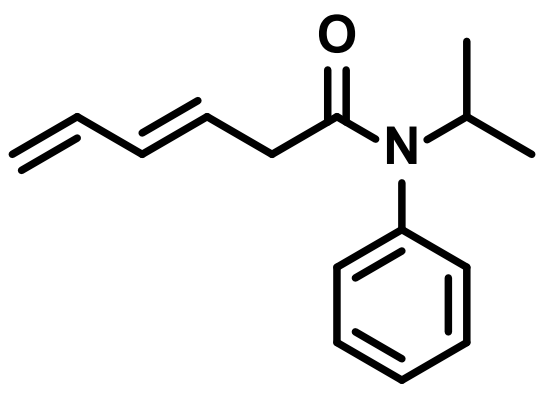

(*E*)-*N*-isopropyl-*N*-phenylhexa-3,5-dienamide

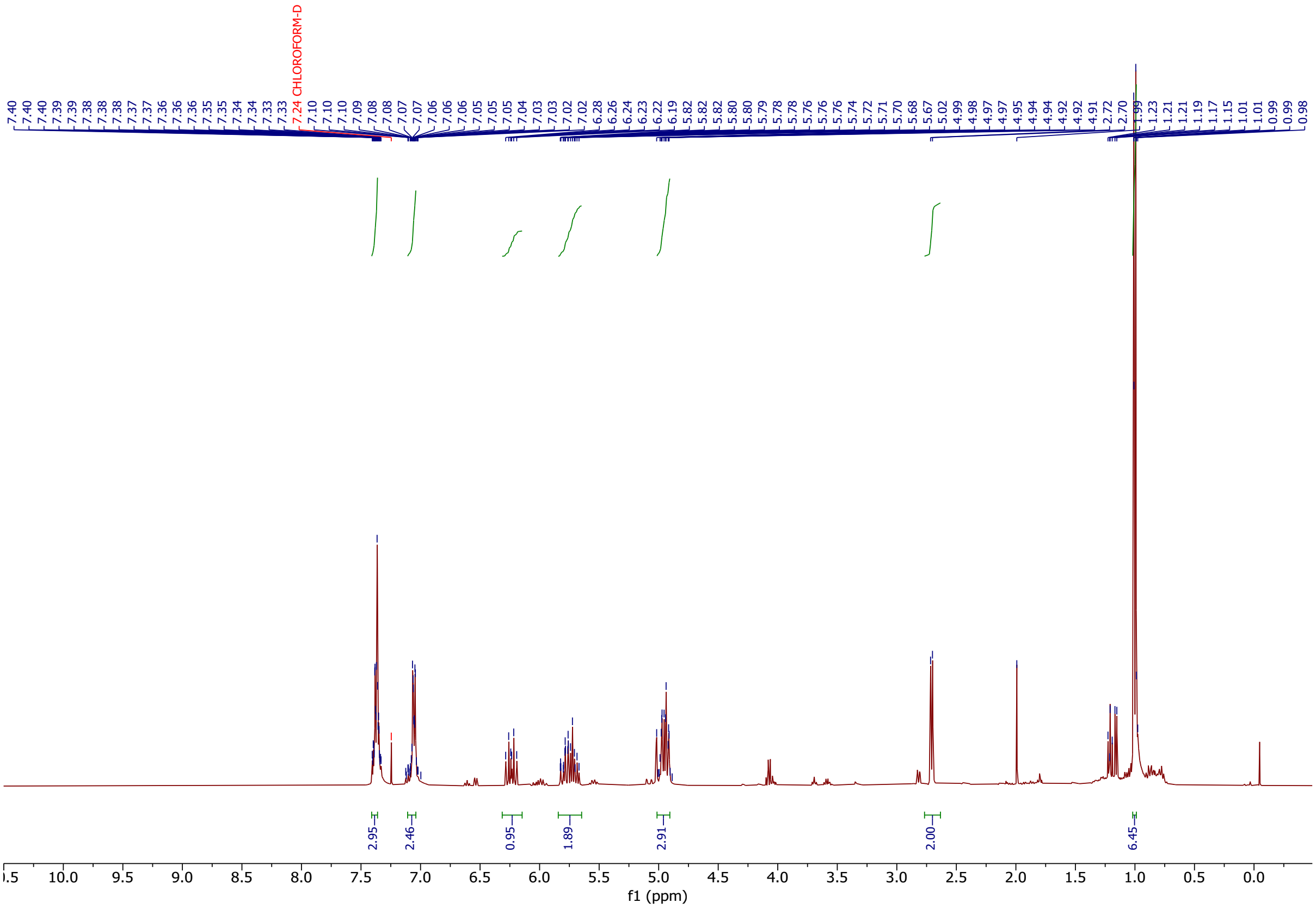

^{13}C NMR

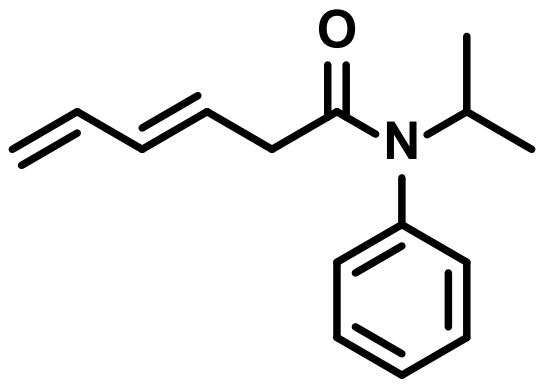

(*E*)-*N*-isopropyl-*N*-phenylhexa-3,5-dienamide

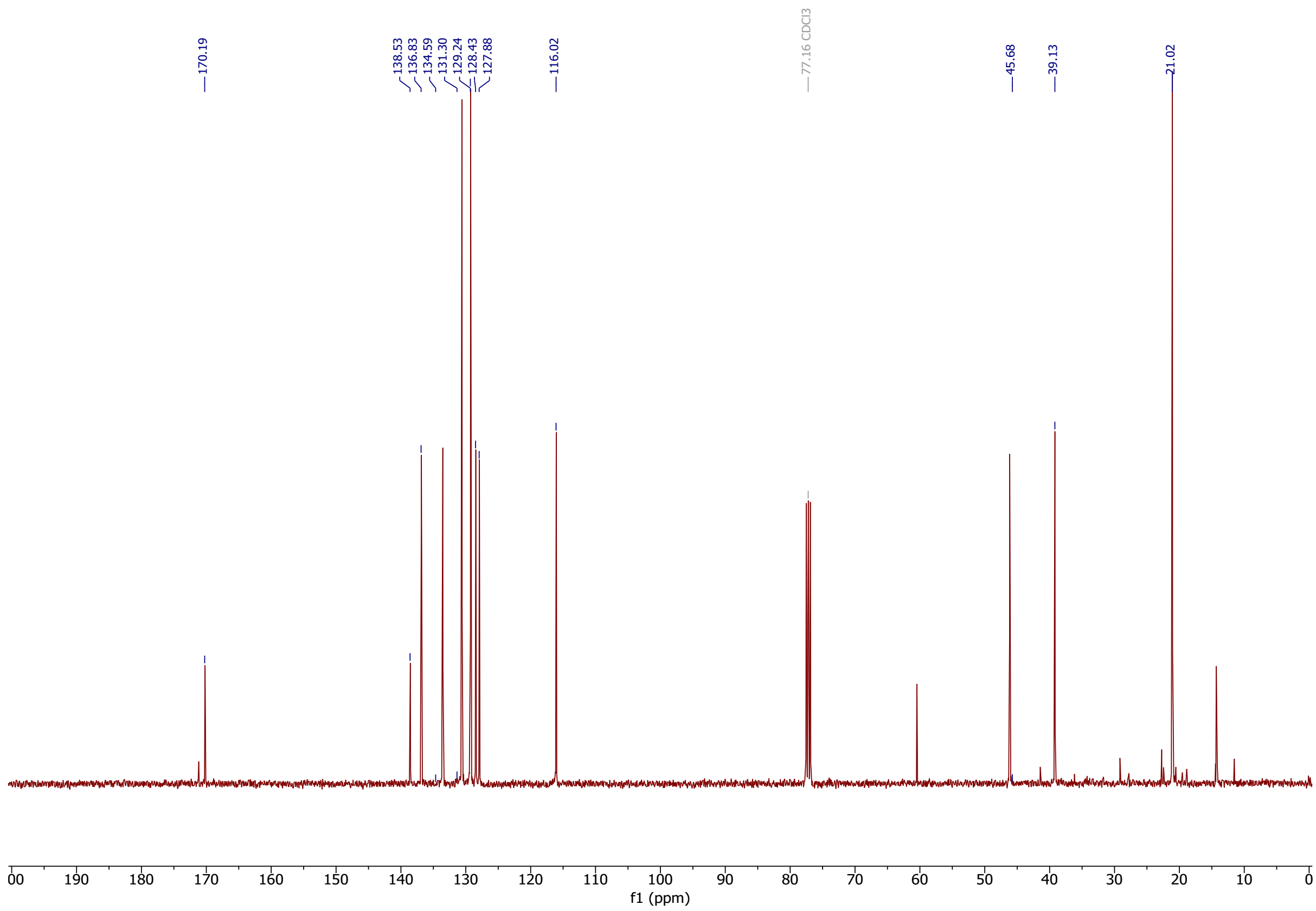

^{1}H NMR

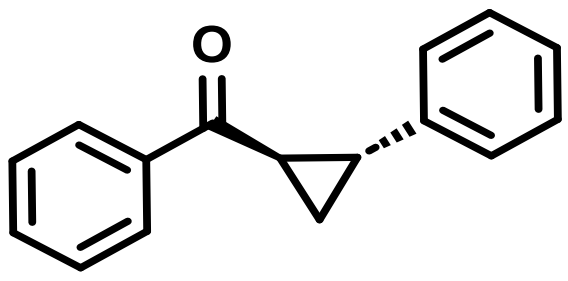

Phenyl((1*R*,2*R*)-2-phenylcyclopropyl)methanone

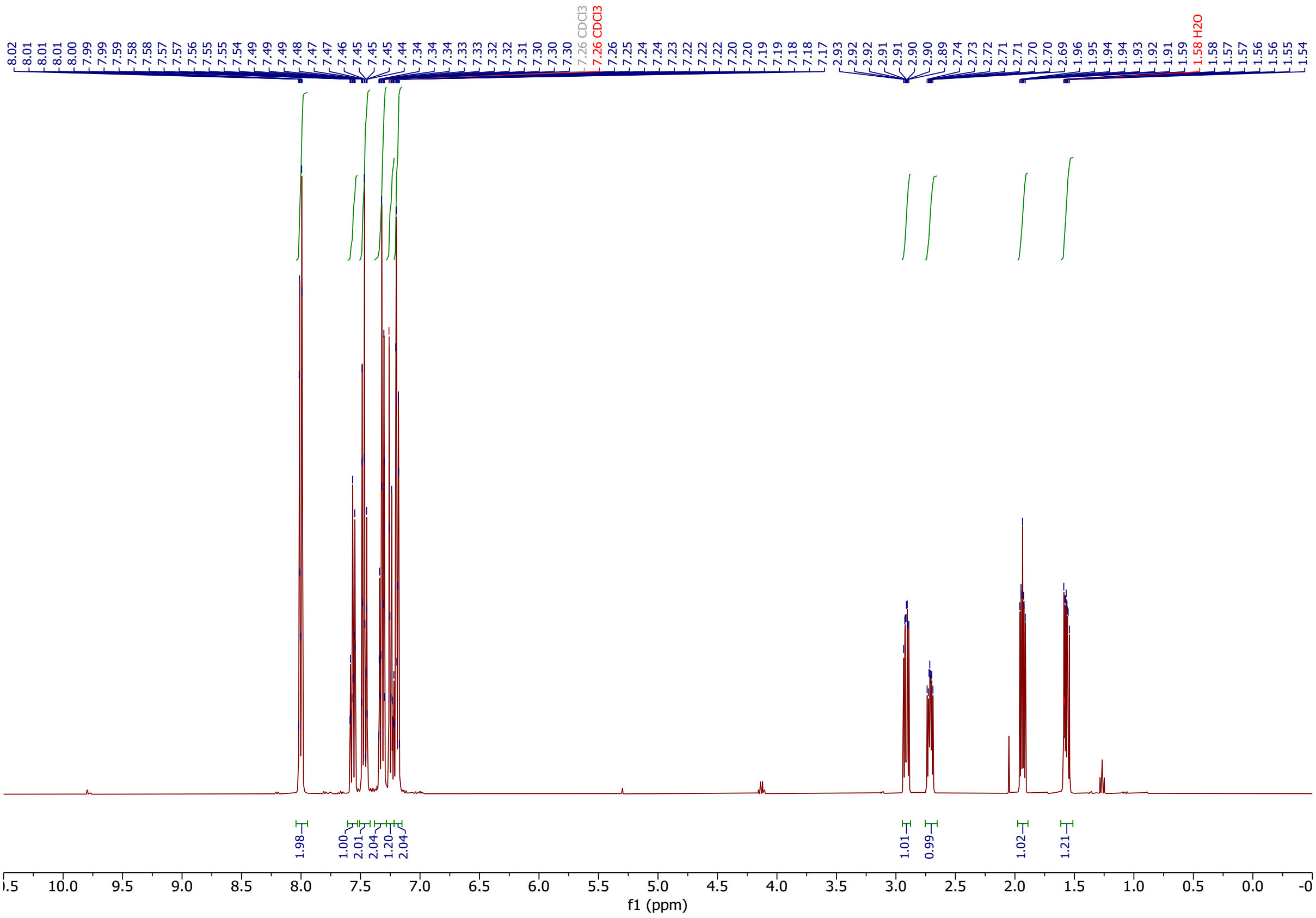

^{13}C NMR

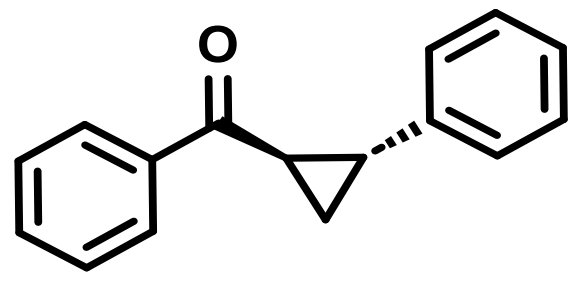

Phenyl((1*R*,2*R*)-2-phenylcyclopropyl)methanone

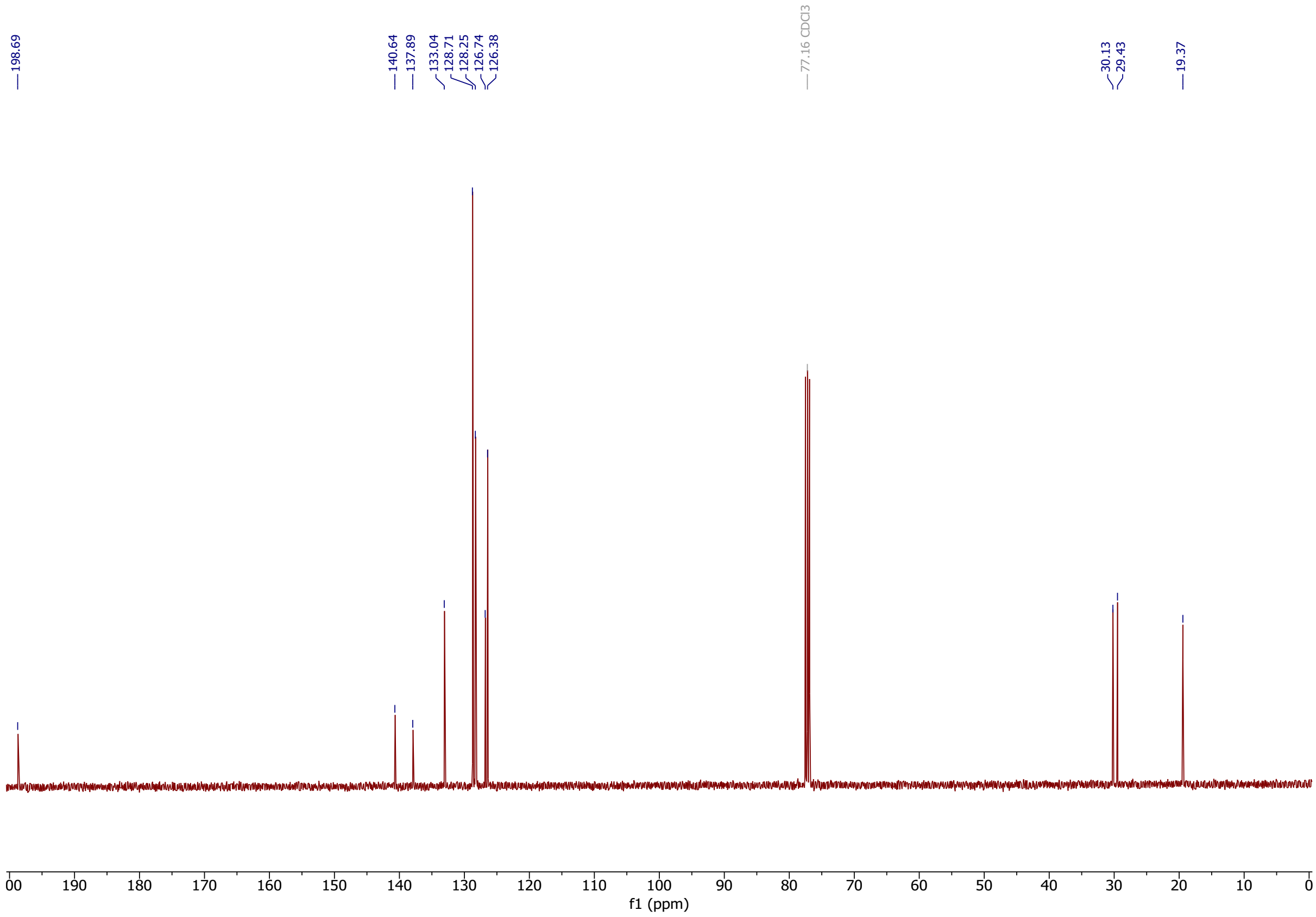

^{1}H NMR

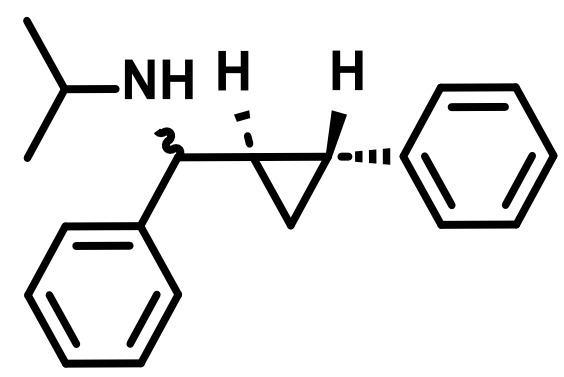

***N*-(phenyl((1*R*,2*R*)-2-phenylcyclopropyl)methyl)propan-2-amine**

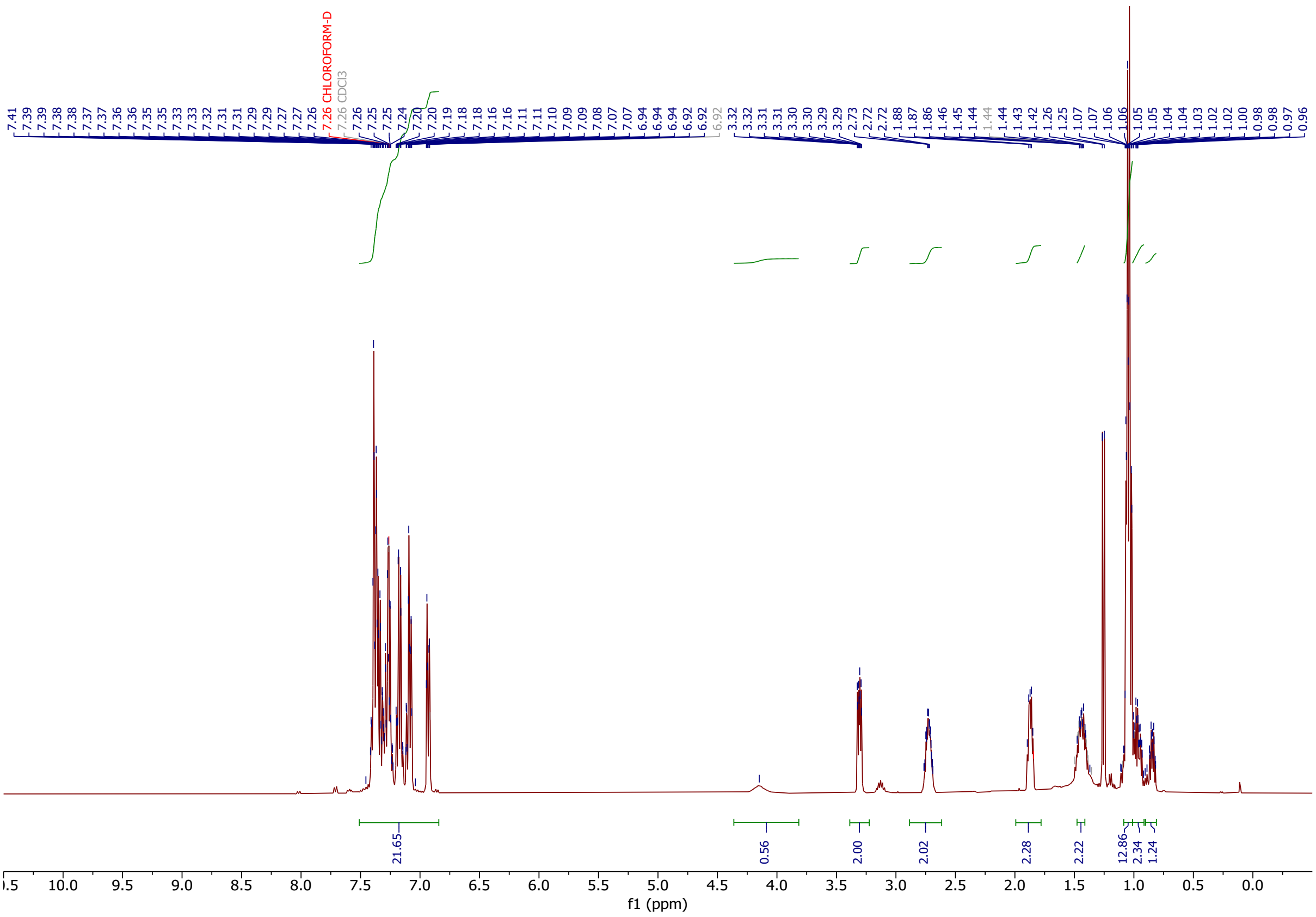

^{13}C NMR

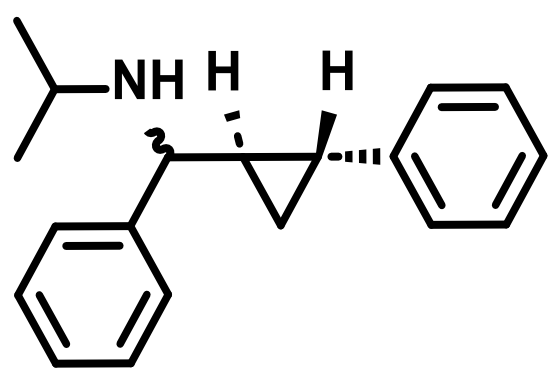

***N*-(phenyl((1*R*,2*R*)-2-phenylcyclopropyl)methyl)propan-2-amine**

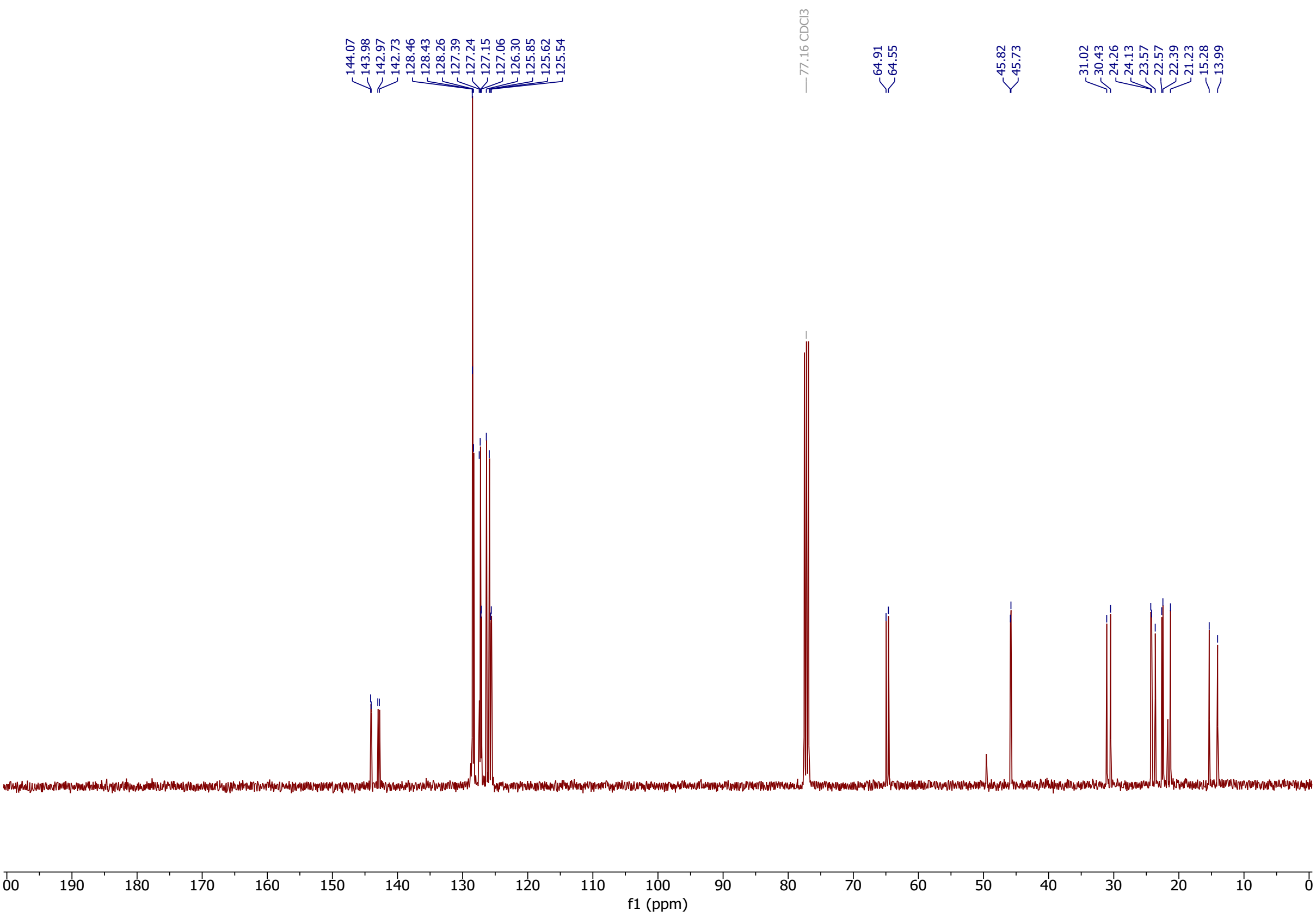

^{1}H NMR

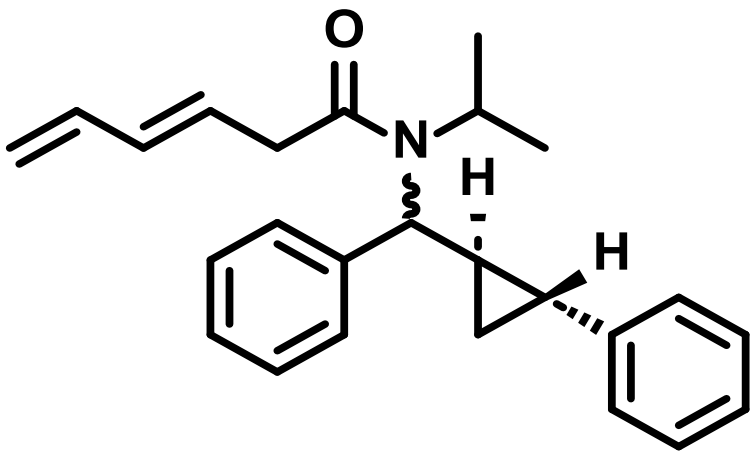

(*E*)-*N*-isopropyl-*N*-(phenyl((1*R*,2*R*)-2-phenylcyclopropyl)methyl)hexa-3,5-dienamide

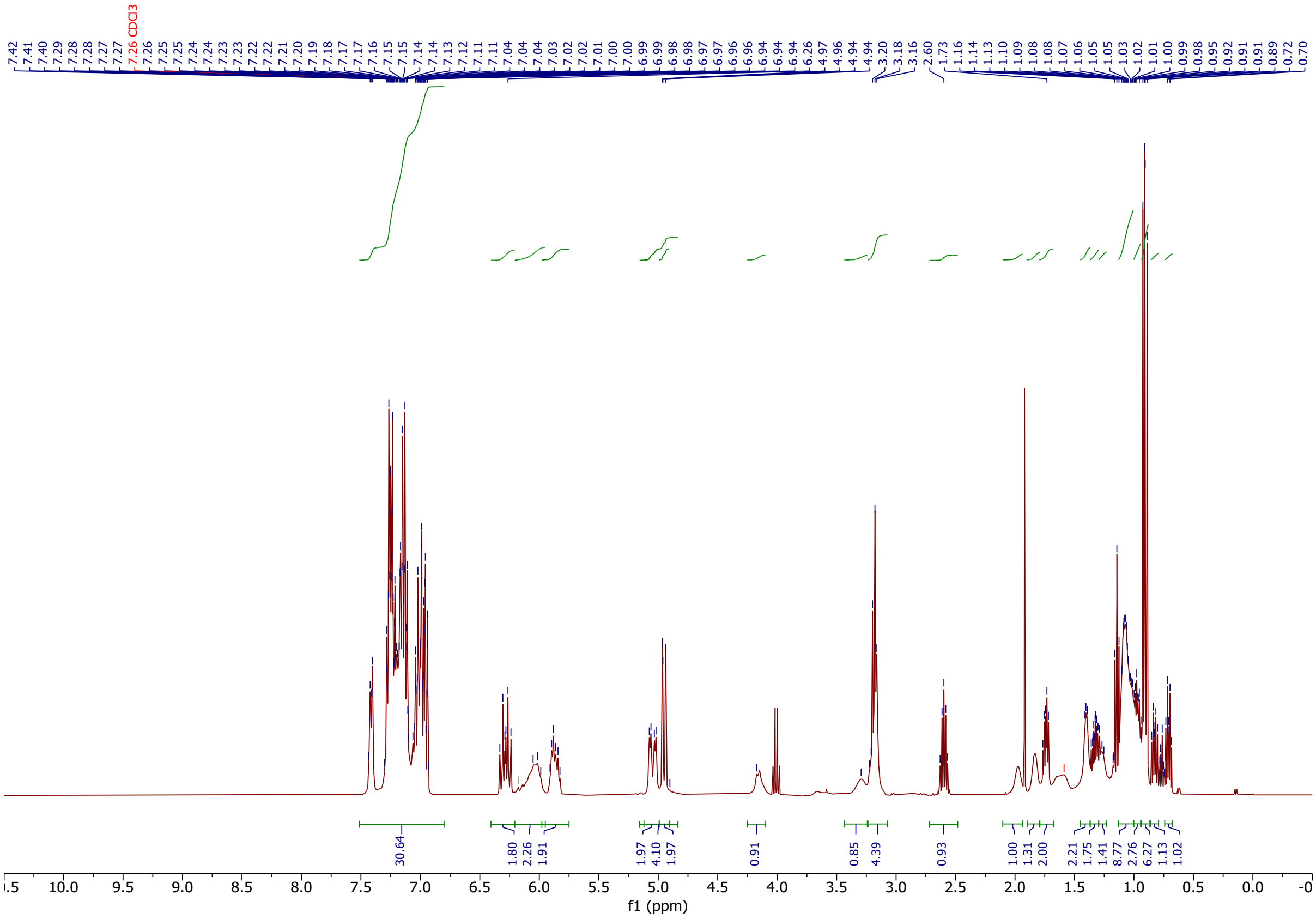

^{13}C NMR

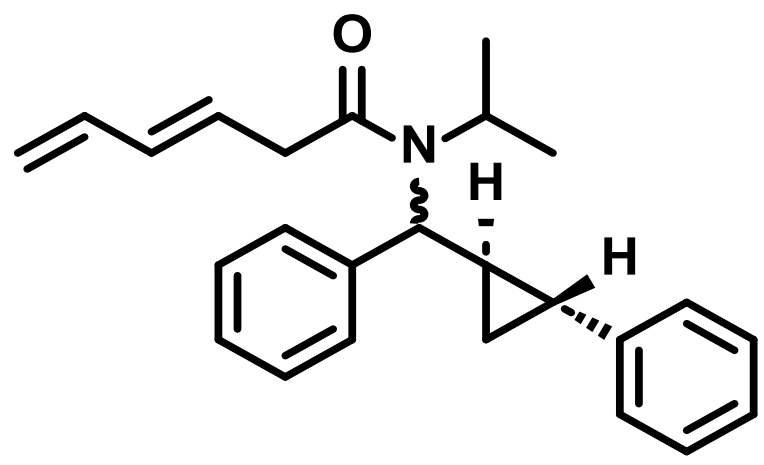

(*E*)-*N*-isopropyl-*N*-(phenyl((1*R*,2*R*)-2-phenylcyclopropyl)methyl)hexa-3,5-dienamide

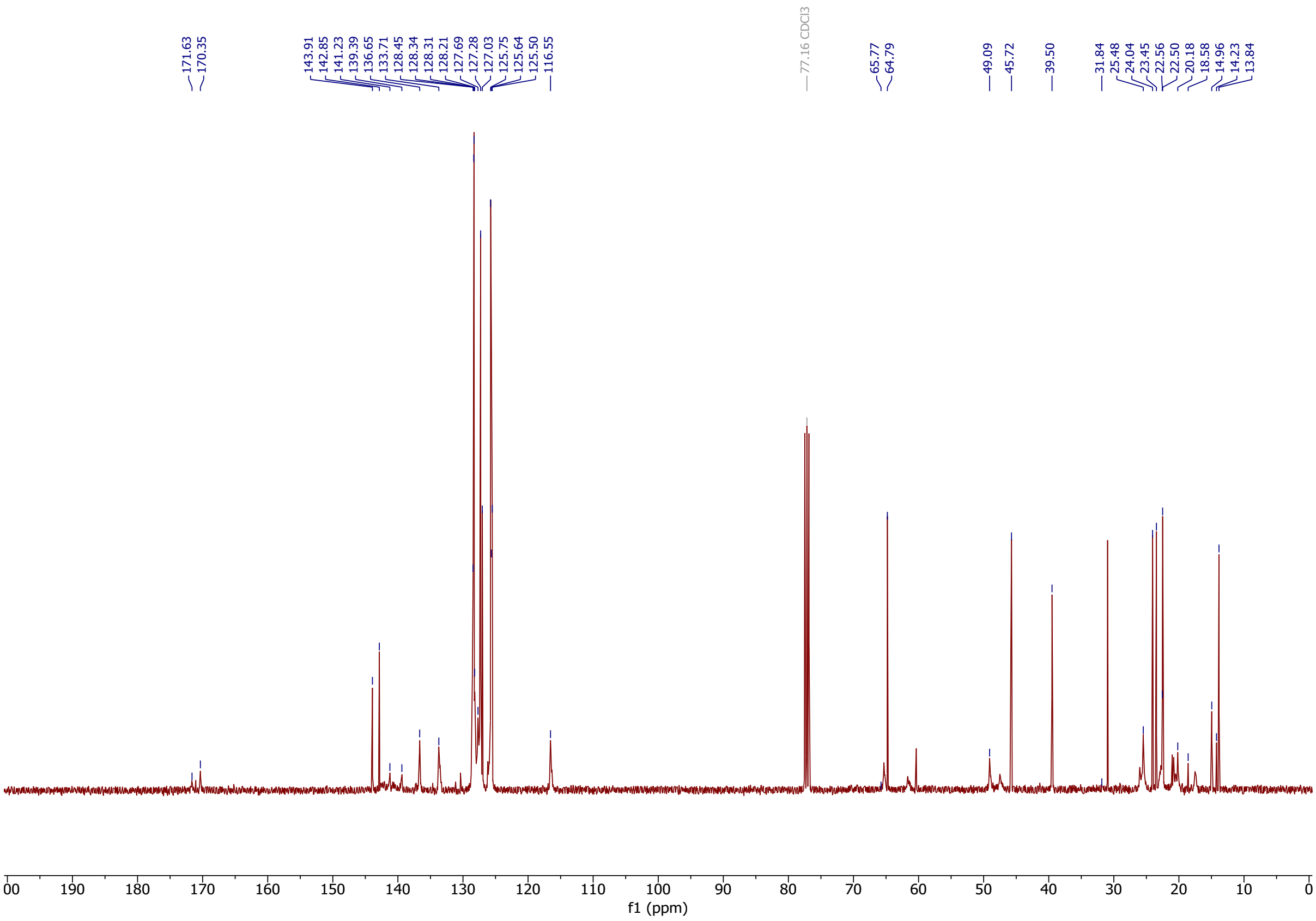

¹H NMR

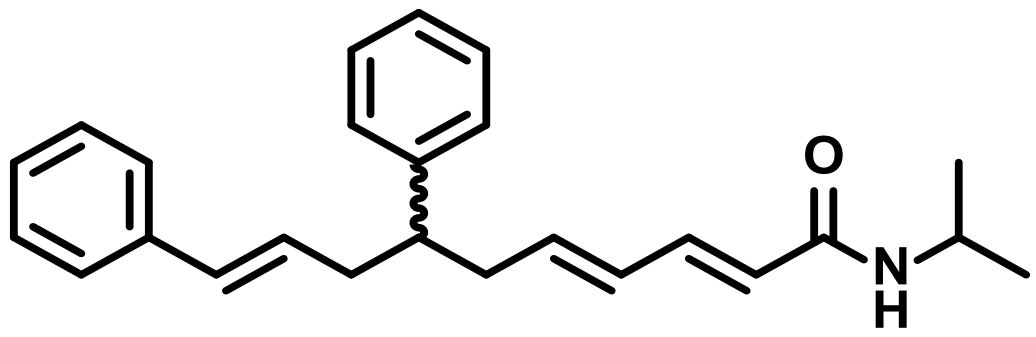

(2*E*,4*E*,9*E*)-*N*-isopropyl-7,10-diphenyldeca-2,4,9-trienamide

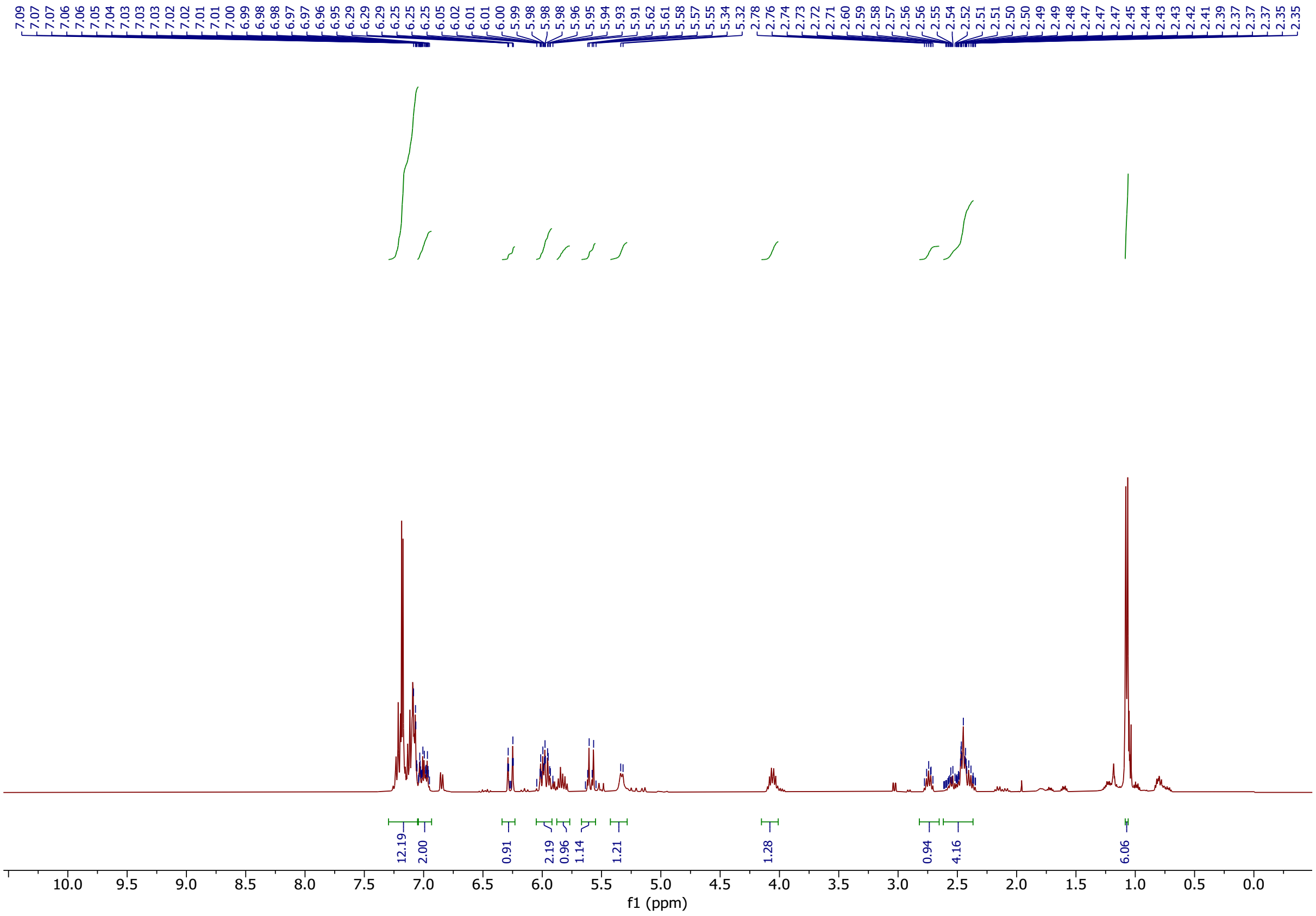

[13] C NMR

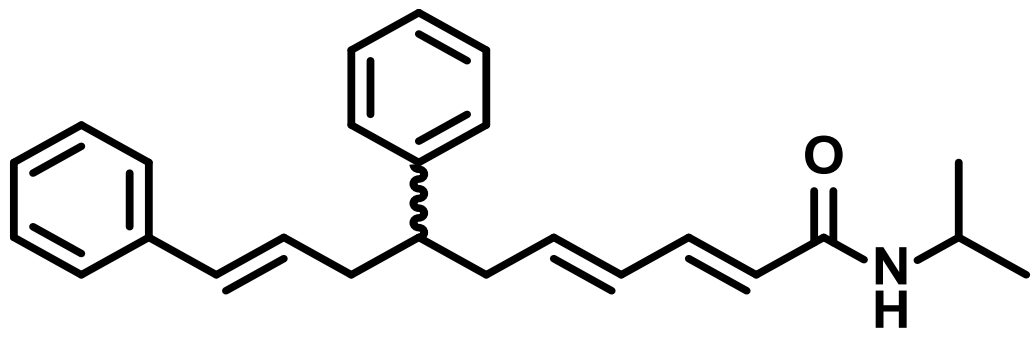

(2*E*,4*E*,9*E*)-*N*-isopropyl-7,10-diphenyldeca-2,4,9-trienamide

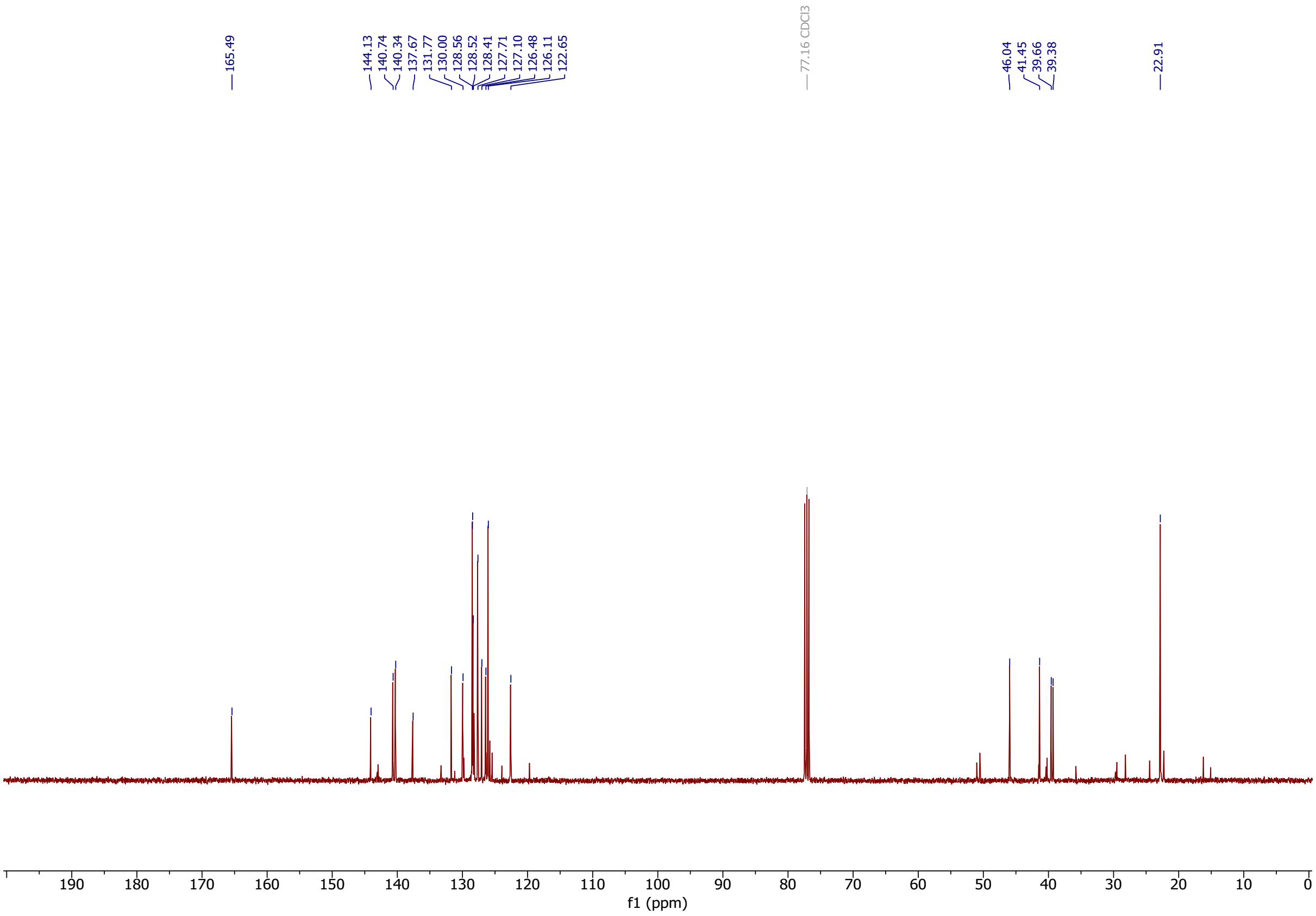

^{1}H NMR

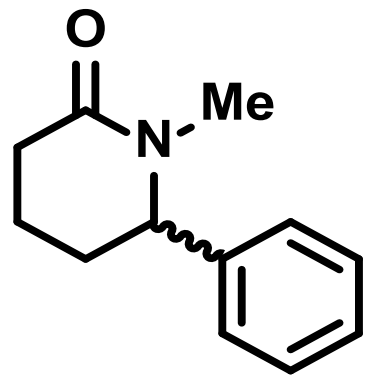

1-Methyl-6-phenylpiperidin-2-one

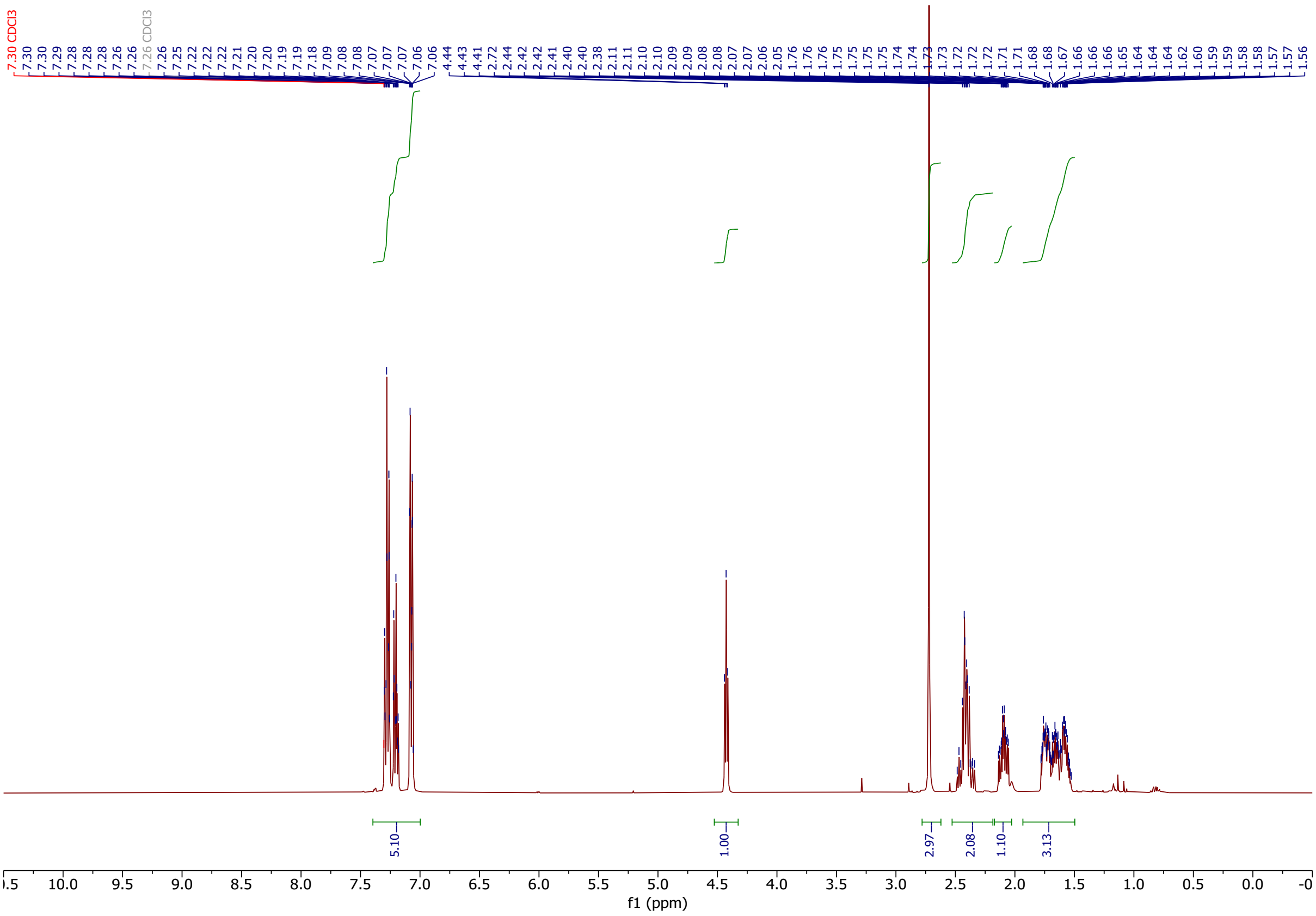

^{13}C NMR

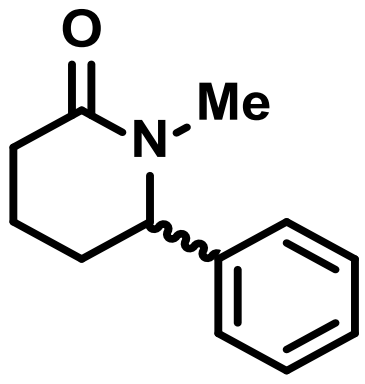

1-Methyl-6-phenylpiperidin-2-one

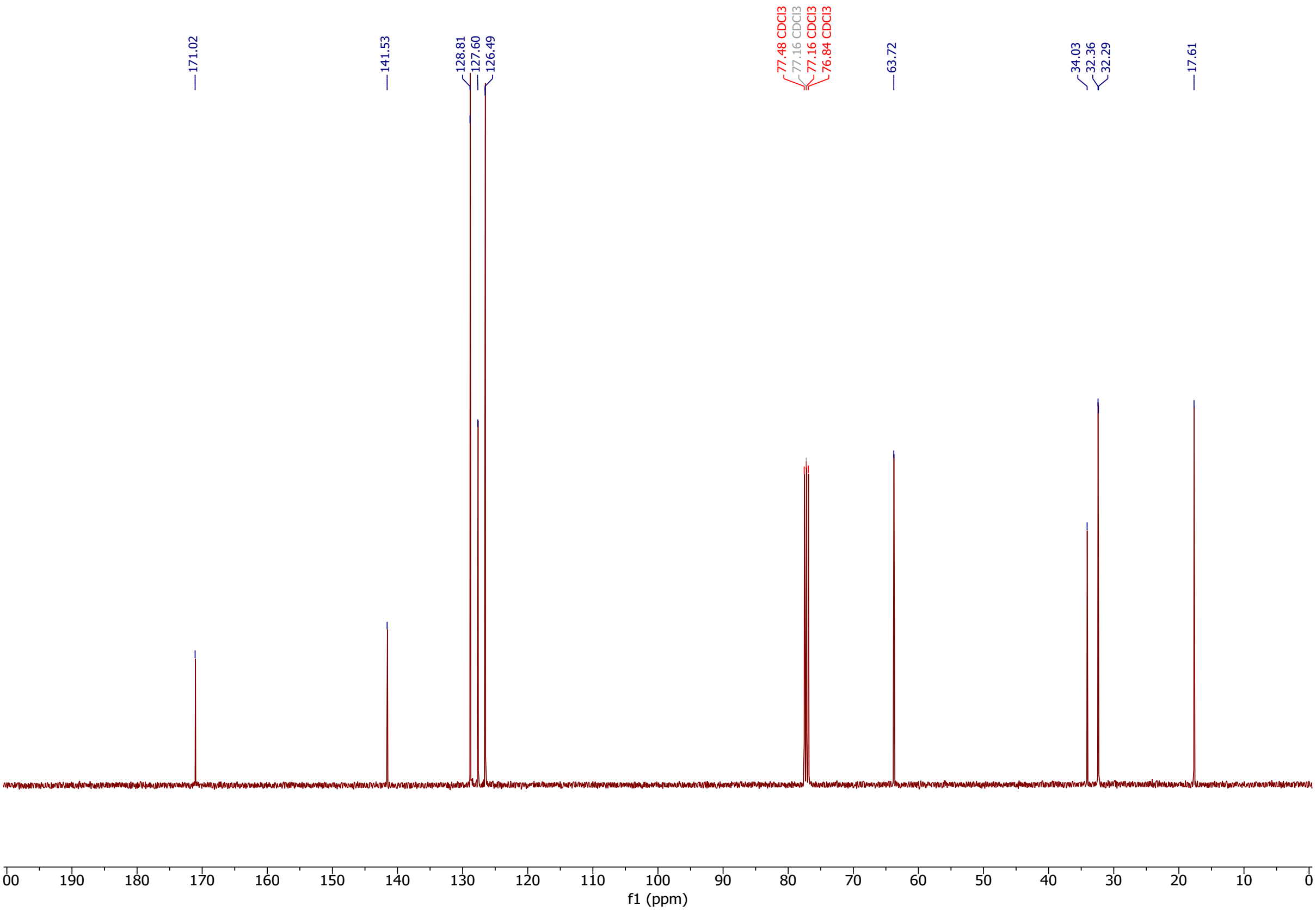

^{1}H NMR

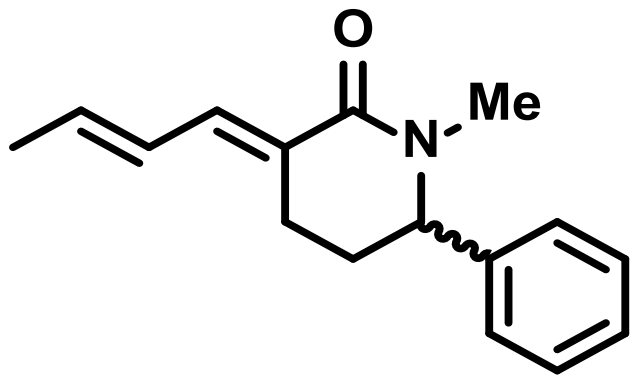

3-(But-2-en-1-ylidene)-1-methyl-6-phenylpiperidin-2-one

^{13}C NMR

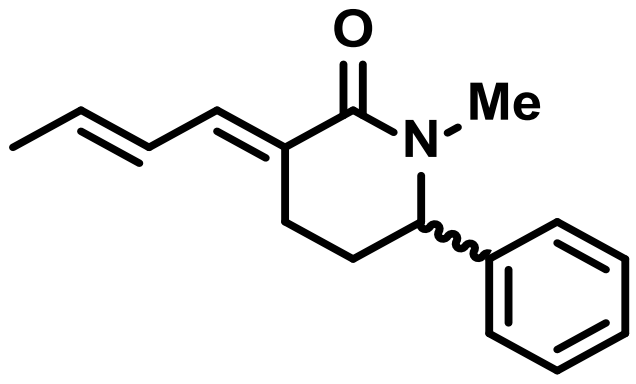

3-(But-2-en-1-ylidene)-1-methyl-6-phenylpiperidin-2-one

^{1}H NMR

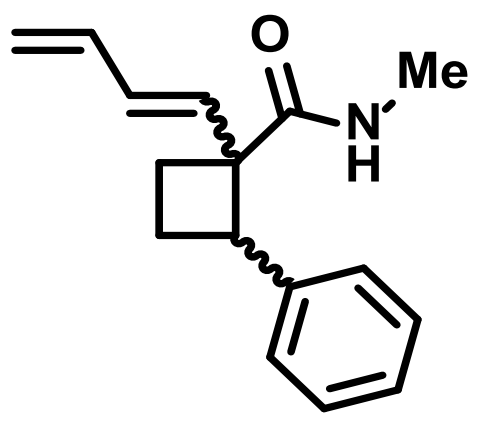

(*E*)-1-(Buta-1,3-dien-1-yl)-*N*-methyl-2-phenylcyclobutane-1-carboxamide

^{13}C NMR

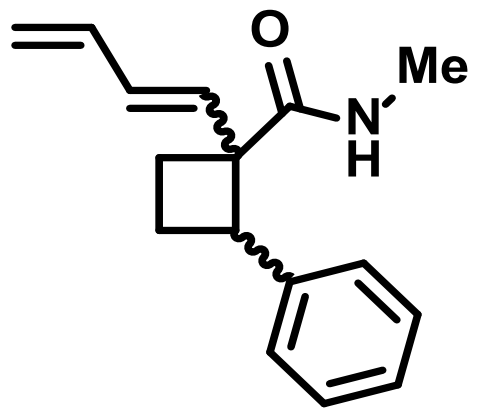

(*E*)-1-(Buta-1,3-dien-1-yl)-*N*-methyl-2-phenylcyclobutane-1-carboxamide

^{1}H NMR

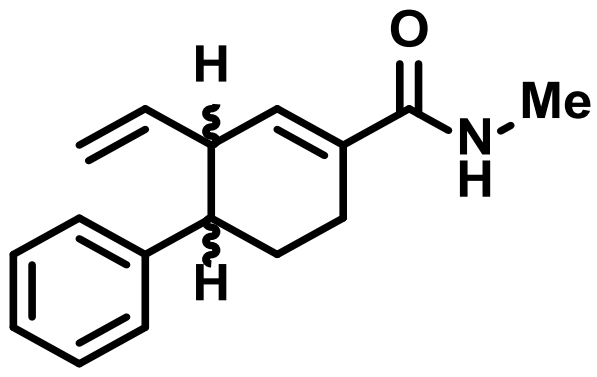

***N*-Methyl-6-vinyl-1,2,3,6-tetrahydro-[1,1’-biphenyl]-4-carboxamide**

^{13}C NMR

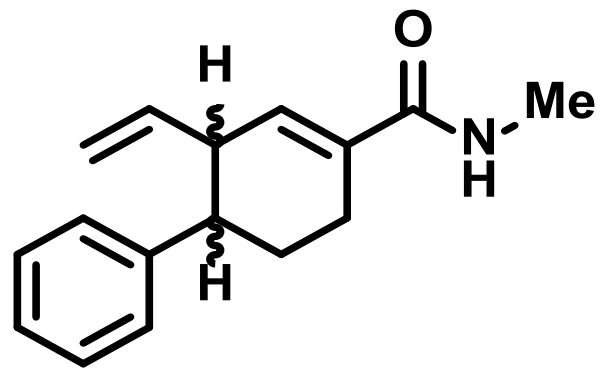

_N_-Methyl-6-vinyl-1,2,3,6-tetrahydro-[1,1'-biphenyl]-4-carboxamide

^{1}H NMR

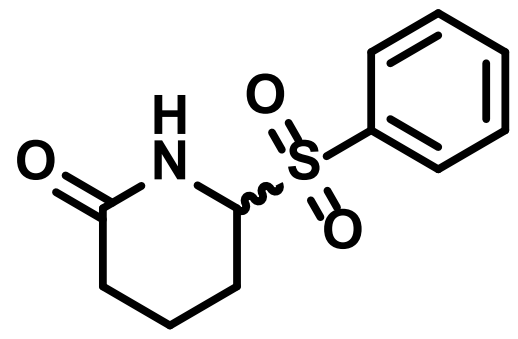

6-(Phenylsulfonyl)piperidin-2-one

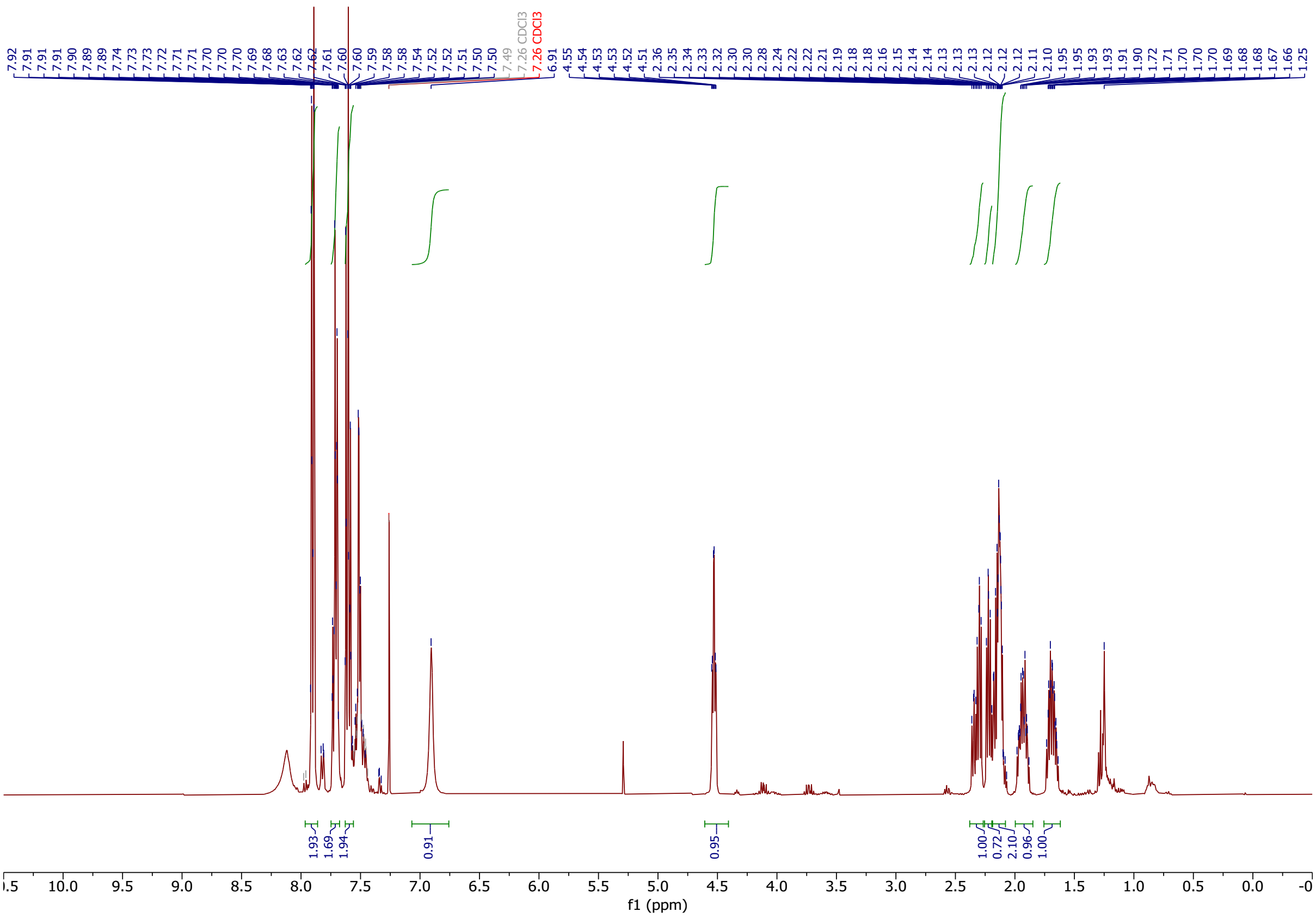

^{13}C NMR

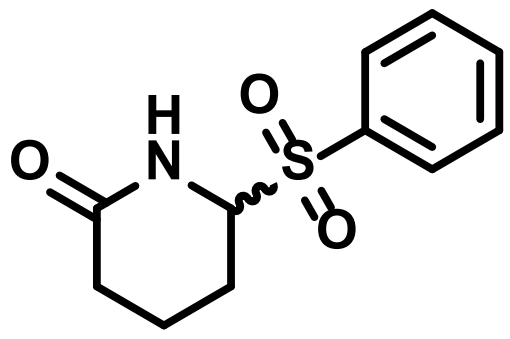

6-(Phenylsulfonyl)piperidin-2-one

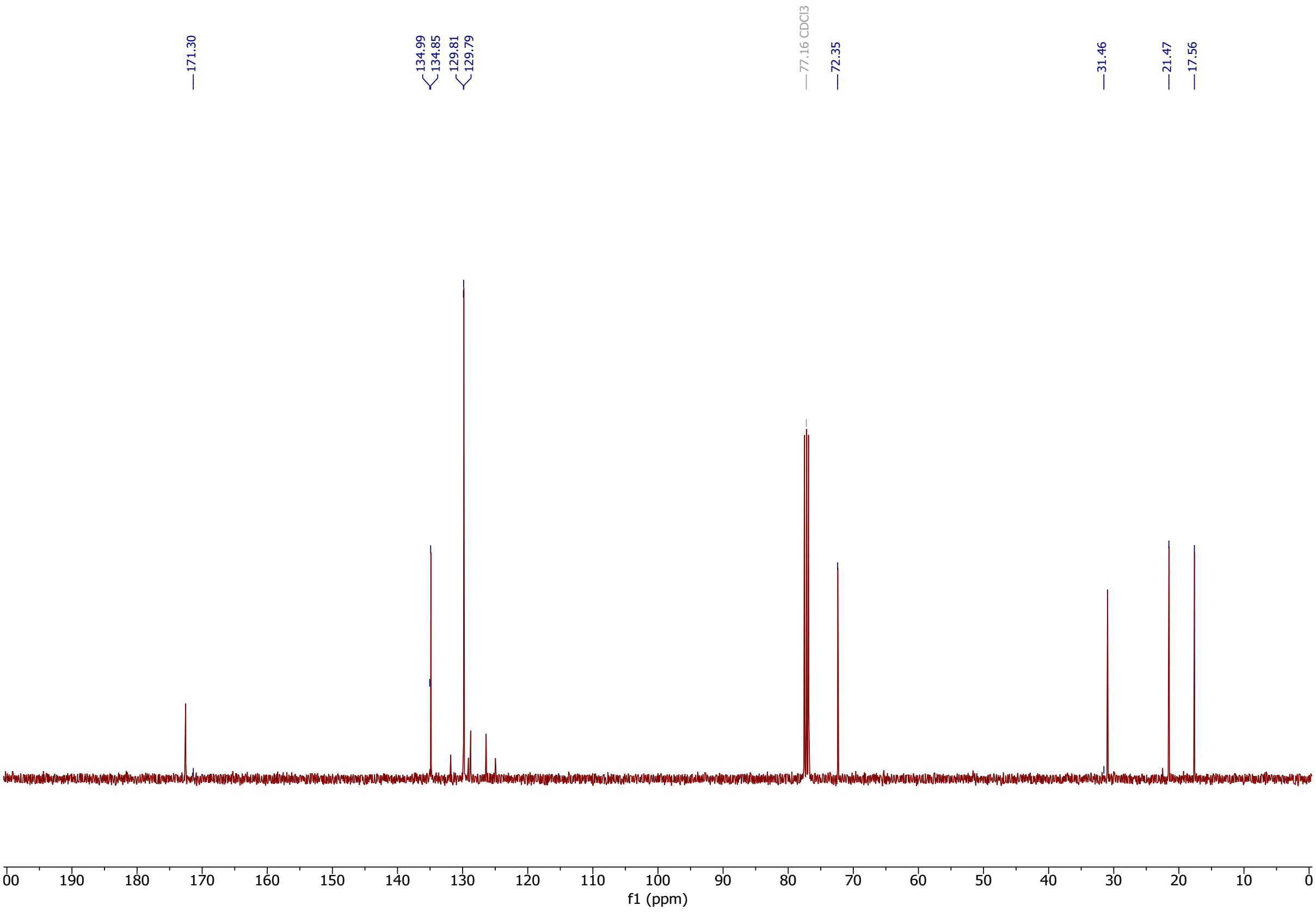

^{1}H NMR

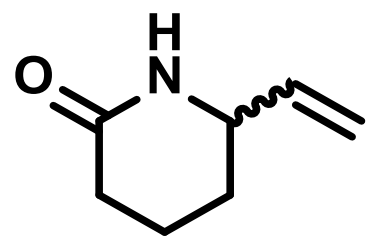

6-Vinylpiperidin-2-one

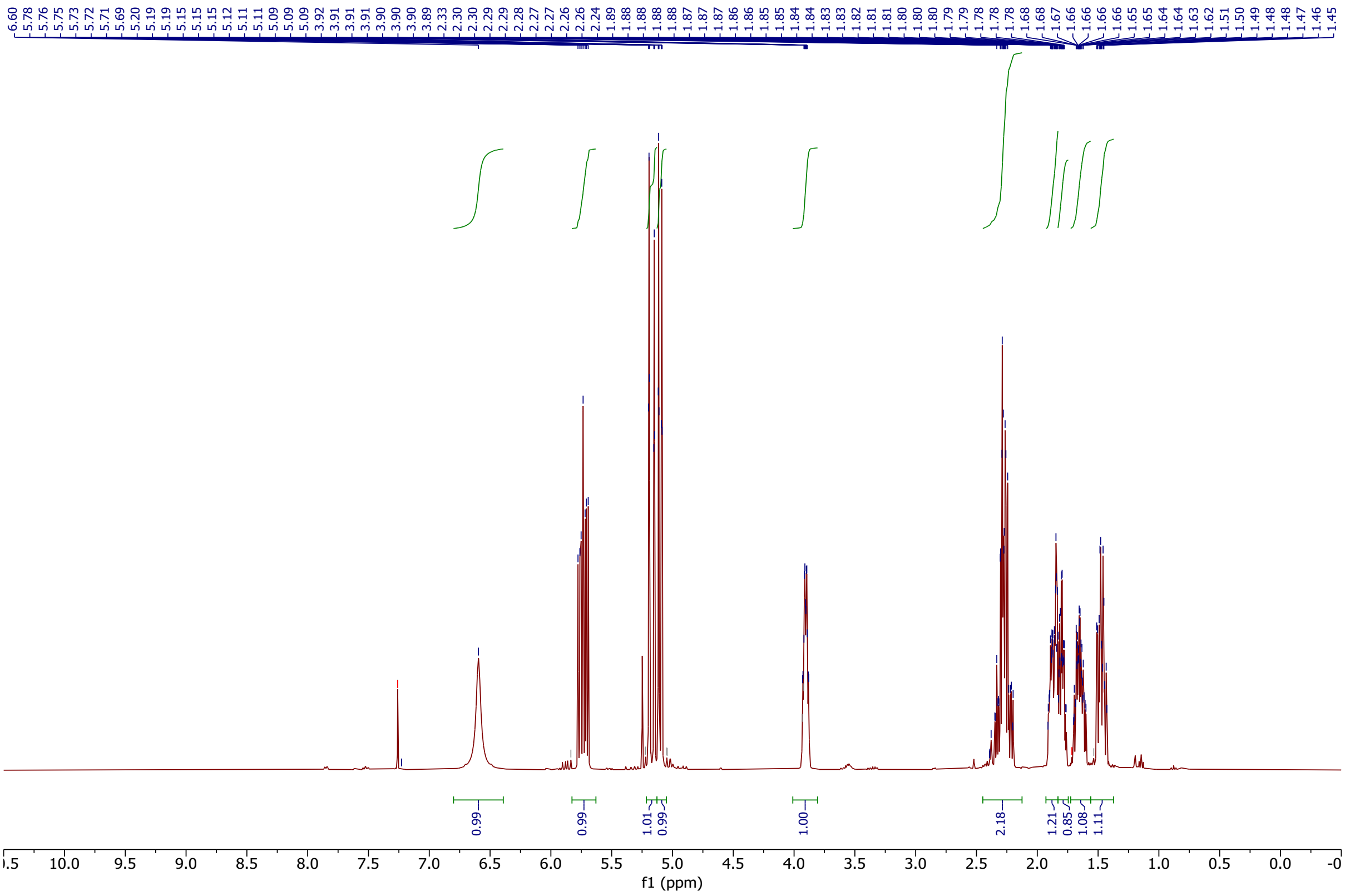

^{13}C NMR

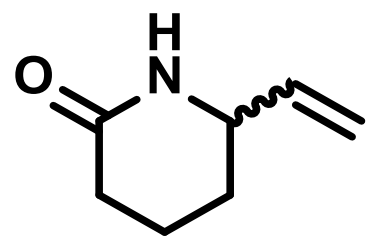

6-Vinylpiperidin-2-one

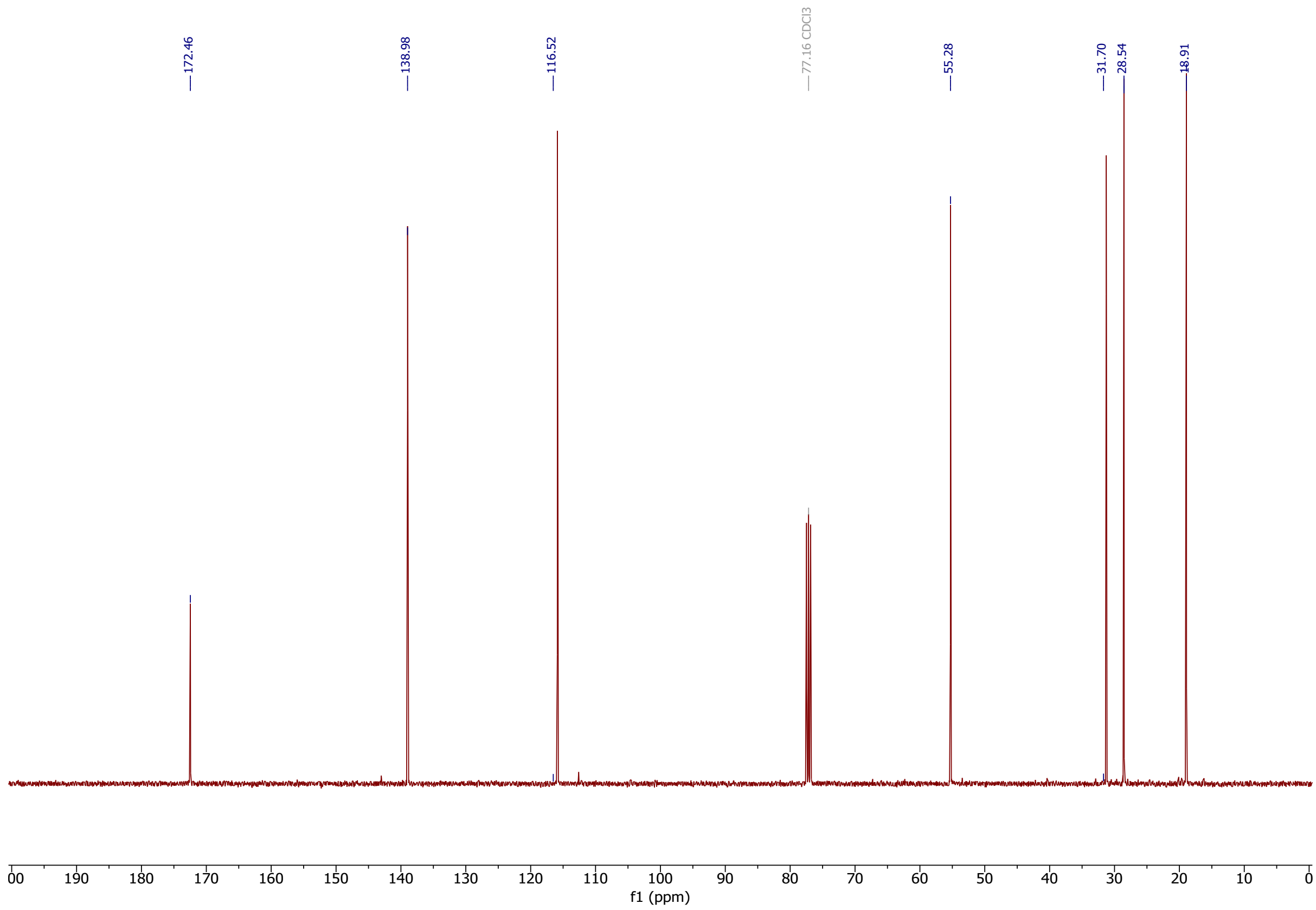

¹H NMR

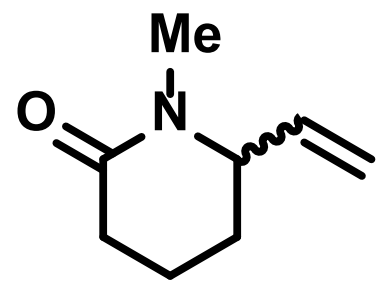

1-Methyl-6-vinylpiperidin-2-one

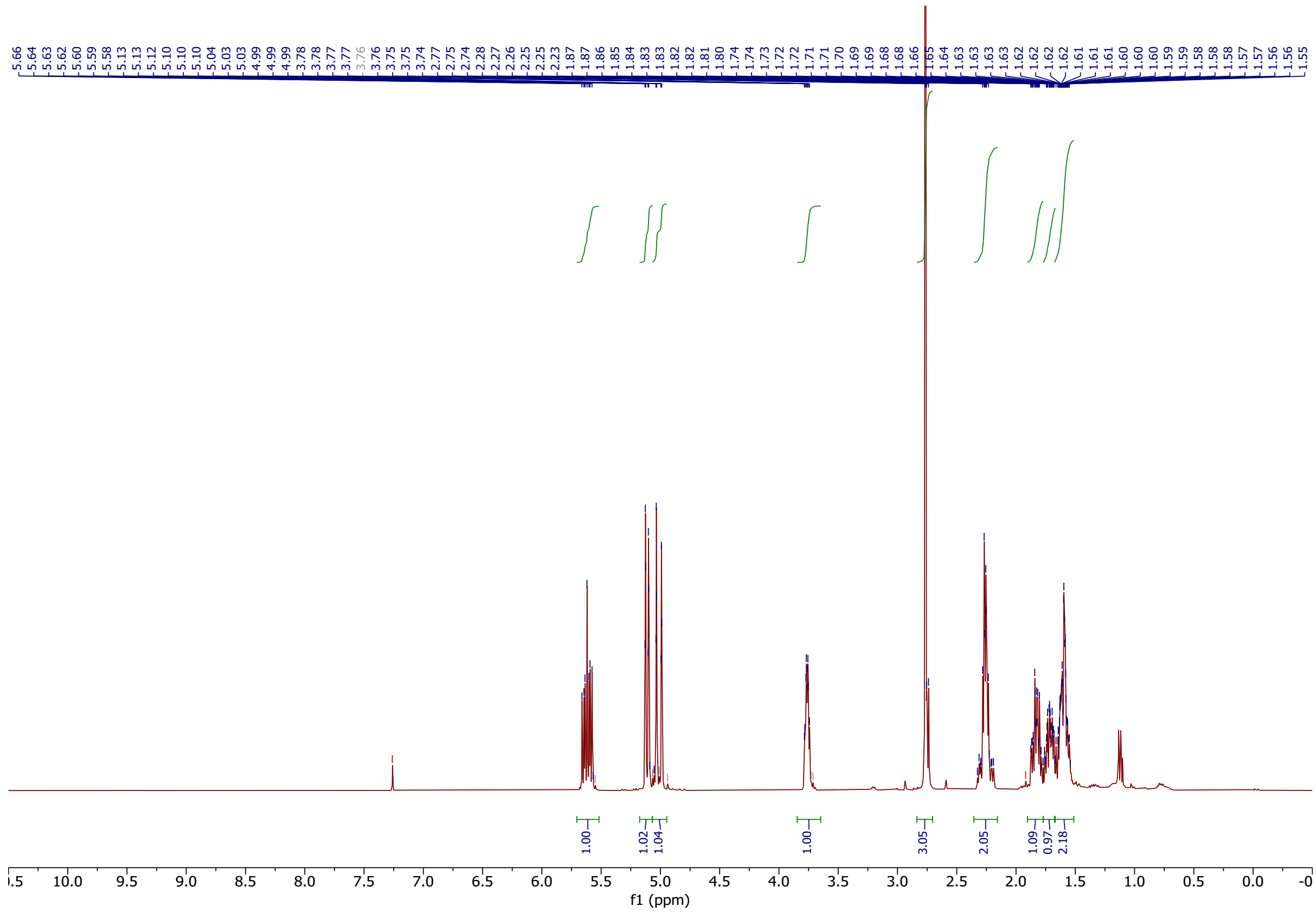

^{13}C NMR

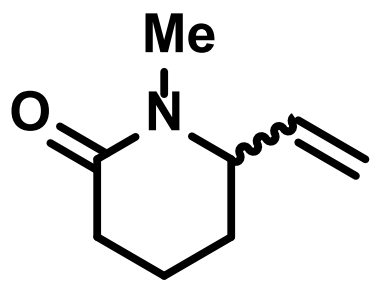

1-Methyl-6-vinylpiperidin-2-one

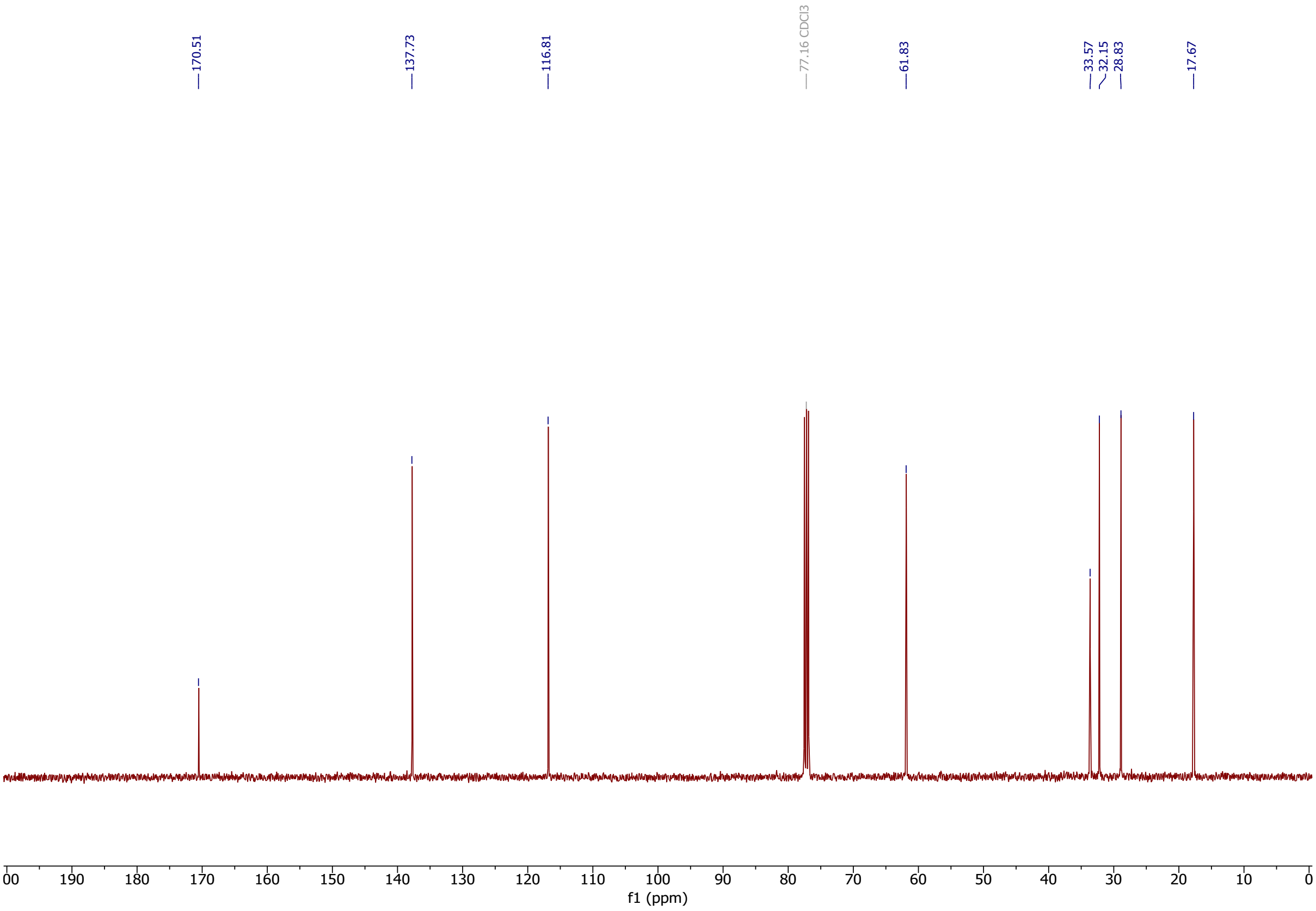

^{1}H NMR

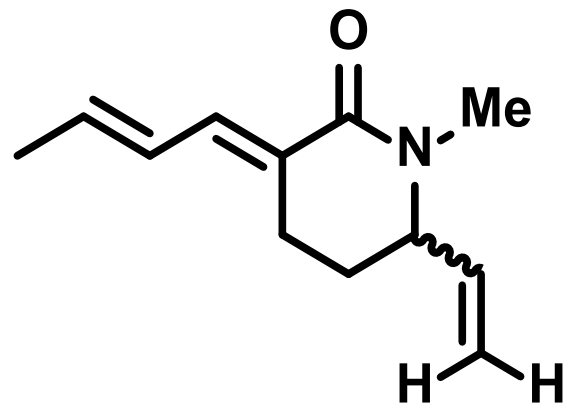

(*E*)-3-((*E*)-but-2-en-1-ylidene)-1-methyl-6-vinylpiperidin-2-one

^{13}C NMR

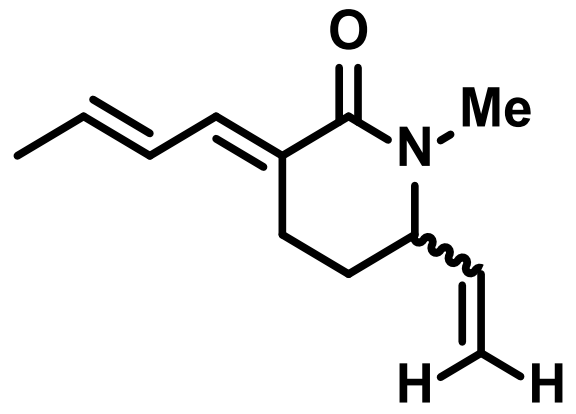

(*E*)-3-((*E*)-but-2-en-1-ylidene)-1-methyl-6-vinylpiperidin-2-one

¹H NMR

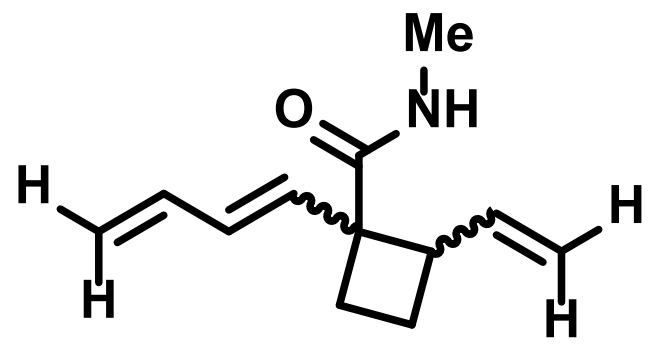

1-(Buta-1,3-dien-1-yl)-*N*-methyl-2-vinylcyclobutane-1-carboxamide

^{1}H NMR

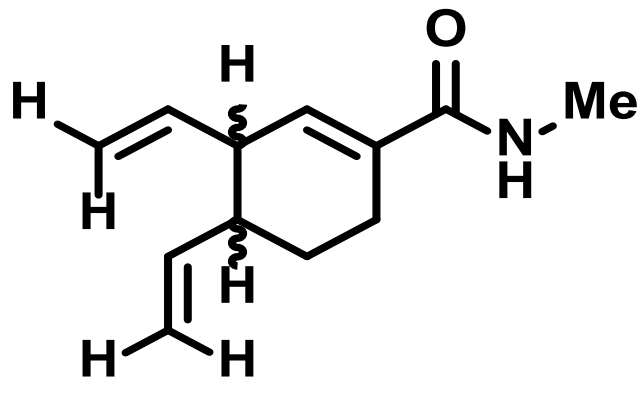

***N*-methyl-3,4-divinylcyclohex-1-ene-1-carboxamide**

^{13}C NMR

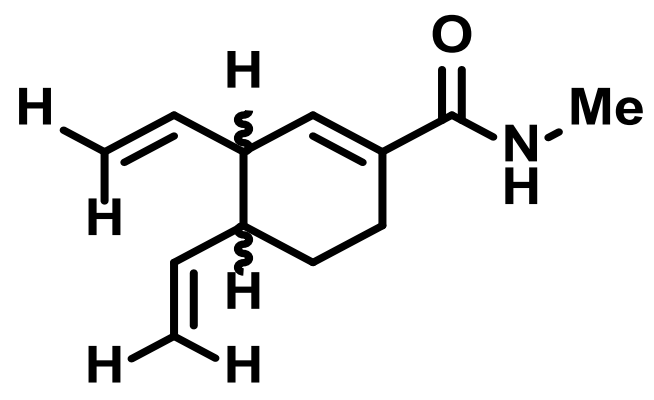

***N*-methyl-3,4-divinylcyclohex-1-ene-1-carboxamide**

3. References

1. T. H. Dunning, Jr., The Journal of Chemical Physics, 1989, **90**, 1007-1023.
2. J.-D. Chai and M. Head-Gordon, The Journal of Chemical Physics, 2008, **128**.
3. F. Neese, Wiley Interdiscip. Rev.:Comput. Mol. Sci., 2012, **2**, 73-78.
4. F. Neese, Wiley Interdiscip. Rev.:Comput. Mol. Sci., 2025, **15**, e70019.
5. Y. J. Franzke, C. Holzer, J. H. Andersen, T. Begušić, F. Bruder, S. Coriani, F. Della Sala, E. Fabiano, D. A. Fedotov, S. Fürst, S. Gillhuber, R. Grotjahn, M. Kaupp, M. Kehry, M. Krstić, F. Mack, S. Majumdar, B. D. Nguyen, S. M. Parker, F. Pauly, A. Pausch, E. Perlt, G. S. Phun, A. Rajabi, D. Rappoport, B. Samal, T. Schrader, M. Sharma, E. Tapavicza, R. S. Treß, V. Voora, A. Wodyński, J. M. Yu, B. Zerulla, F. Furche, C. Hättig, M. Sierka, D. P. Tew and F. Weigend, J. Chem. Theory Comput., 2023, **19**, 6859-6890.
6. M. Barbatti, M. Bondanza, R. Crespo-Otero, B. Demoulin, P. O. Dral, G. Granucci, F. Kossoski, H. Lischka, B. Mennucci, S. Mukherjee, M. Pederzoli, M. Persico, M. Pinheiro Jr, J. Pittner, F. Plasser, E. Sangiogo Gil and L. Stojanovic, J. Chem. Theory Comput., 2022, **18**, 6851-6865.
7. J. Bone, J. Carmona-García, D. Hollas and B. F. E. Curchod, J. Phys. Chem. A, 2025, **129**, 9355-9367.
8. X. Zhu, K. C. Thompson and T. J. Martínez, J. Chem. Phys., 2019, **150**, 164103.
9. M. J. Frisch, G. W. Trucks, H. B. Schlegel, G. E. Scuseria, M. A. Robb, J. R. Cheeseman, G. Scalmani, V. Barone, G. A. Petersson, H. Nakatsuji, X. Li, M. Caricato, A. V. Marenich, J. Bloino, B. G. Janesko, R. Gomperts, B. Mennucci, H. P. Hratchian, J. V. Ortiz, A. F. Izmaylov, J. L. Sonnenberg, Williams, F. Ding, F. Lipparini, F. Egidi, J. Goings, B. Peng, A. Petrone, T. Henderson, D. Ranasinghe, V. G. Zakrzewski, J. Gao, N. Rega, G. Zheng, W. Liang, M. Hada, M. Ehara, K. Toyota, R. Fukuda, J. Hasegawa, M. Ishida, T. Nakajima, Y. Honda, O. Kitao, H. Nakai, T. Vreven, K. Throssell, J. A. Montgomery Jr., J. E. Peralta, F. Ogliaro, M. J. Bearpark, J. J. Heyd, E. N. Brothers, K. N. Kudin, V. N. Staroverov, T. A. Keith, R. Kobayashi, J. Normand, K. Raghavachari, A. P. Rendell, J. C. Burant, S. S. Iyengar, J. Tomasi, M. Cossi, J. M. Millam, M. Klene, C. Adamo, R. Cammi, J. W. Ochterski, R. L. Martin, K. Morokuma, O. Farkas, J. B. Foresman and D. J. Fox, 2016, Gaussian 16 Rev. C.01, Wallingford, CT.
10. Y. Horbatenko, S. Sadiq, S. Lee, M. Filatov and C. H. Choi, J. Chem. Theory Comput., 2021, **17**, 848-859.
11. A. Prlj, E. Marsili, L. Hutton, D. Hollas, D. Shchepanovska, D. R. Glowacki, P. Slavíček and B. F. E. Curchod, ACS Earth Space Chem., 2022, **6**, 207-217.
12. R. Crespo-Otero and M. Barbatti, Theor. Chem. Acc., 2012, **131**, 1237.
13. R. K. Saunthwal, J. Mortimer, A. J. Orr-Ewing and J. Clayden, Chem. Sci., 2022, **13**, 2079-2085.
14. H. Li, H. Wu, H. Zhang, Y. Su, S. Yang and E. J. M. Hensen, ChemSusChem, 2019, **12**, 3778-3784.
15. L. P. S. Santos, Ronaldo A., J. Braz. Chem. Soc., 2003, **14**, 982-993.
16. P. Ballester, A. Costa, A. García-Raso, A. Gómez-Solivellas and R. Mestres, Tetrahedron Lett., 1985, **26**, 3625-3628.
17. S. E. Hampton, B. Baragaña, A. Schipani, C. Bosch-Navarrete, J. A. Musso-Buendía, E. Recio, M. Kaiser, J. L. Whittingham, S. M. Roberts, M. Shevtsov, J. A. Brannigan, P. Kahnberg, R. Brun, K. S. Wilson, D. González-Pacanowska, N. G. Johansson and I. H. Gilbert, ChemMedChem, 2011, **6**, 1816-1831.

18. J. A. Ciaccio, A. L. Drahus, R. M. Meis, C. T. Tingle, M. Smrtka and R. Geneste, Synth. Commun., 2003, **33**, 2135-2143.
19. C. E. Neipp and S. F. Martin, J. Org. Chem., 2003, **68**, 8867-8878.
